# Supplementary material for: Implementing a Holistic Review Toolkit for Faculty Recruitment and Retention
Source: MedEdPORTAL. 2024 Dec 4;20:11472. doi: 10.15766/mep_2374-8265.11472 (PMC11615027; doi:10.15766/mep_2374-8265.11472)
Supplement: Supplementary file 1 — Faculty Pilot Overview.docxOverview Equity-Minded Hiring_Step 1.docxAssess Readiness for Equity-Minded Hiring_Step 1.docxStaff Composition Inventory_Step 2.xlsxHolistic Search Committee Phases and Steps_Step 2.docxFaculty Workshop Facilitators Guide_Step 3.docxFaculty Workshop Presentation_Step 3.pptxFaculty Workshop Evaluation_Step 3.docxFaculty Workshop Activities_Step 3.docxJob Description Posting Tools and Resources_Step 4.docxInterview Questions Tools and Resources_Step 4.docxSubmission Requirements and Rating Tools_Step 4.docx360-Degree (Multisource) Reference Checking_Step 4.docxSearch Process Tools and Resources_Step 5.docxStanding Up a Search Committee_Step 5.docxMitigating Bias Resources_Step 5.docxOnboarding Tools and Resources_Step 6.docxCareer Development Discussion Guide_Step 6.docxU Colorado SOM Mentoring Resource Packet_Step 6.docxBaylor College of Medicine Exit Resources_Step 6.docxU Colorado SOM Equitable Hiring Tool_Step 7.docxHolistic Hiring and Retention Tracker_Step 8.docxEvaluation Materials Development Phase_Steps 4-6.docx [file mep_2374-8265.11472-s001.zip › Q. Onboarding Tools and Resources_Step 6.docx]

# Appendix Q: Onboarding Tools and Resources

### Department Chair Onboarding

Implementation Guidance: Use this checklist as a guide for successful department chair onboarding, from what to do before the new chair arrives through the first 30 days. You can adapt any of these steps to your unique needs. Successful onboarding of new department chairs should include building knowledge in each of the following four areas:

- Compliance: knows about basic legal and policy-related rules and regulations
- Clarification: understands the position expectations
- Culture: has a sense of organizational norms
- Connection: establishes interpersonal relationships and information networks

**The Role of the Dean Is Critical in This Process:** Ross et al.^1^ found that new chairs “lamented the passive role the dean played in their arrival.” Being active and present is a major opportunity for the dean. The dean may delegate some of the details to associate deans; however, the dean should be present in the eyes of the new chair and in the eyes of the other chairs.

Originally published in Mallon WT, Grigsby, RK. *Recruiting: Proven Search and Hiring Practices for the Best Talent*. Association of American Medical Colleges; 2017. Additional resources and information can be found on AAMC’s Hiring the Best Talent Web site.^2^

| **Before the First Day** | | |
| --- | --- | --- |
| **✓** | **Person Responsible** | **Tasks** |
|  | HR | - Finalizes start date - Confirms onboarding roles and responsibilities - Sends an email to the new chair with additional instructions |
|  | Department Administrator | - Serves as orientation navigator, ensuring the process goes smoothly - Requests technology equipment - Requests phone setup - Orders office supplies - Updates department documents and databases - Arranges for keys, badges, parking, etc. - Schedules meetings with people outside the department (dean will provide this list) |
|  | IT | - Configures and sets up equipment |
|  | Dean | - Identifies a mentor - Schedules regular meetings with the new chair - Adds the new chair to standing meetings - Schedules the first meeting with faculty - Puts together a list of people outside the department the new chair should meet within the first few weeks of the appointment (e.g., other chairs, center directors, chief medical officer, IRB chair) - Schedules a welcome call to answer any remaining questions |
|  | Search Chair | - Blocks off time to spend with the new chair |
|  | Peer Mentor | - Contacts the incoming chair; typically, a sitting chair from another department fills this role - Blocks off time to spend with the new chair to become acquainted with and socialized into the organizational culture at the medical school and health system level |
|  | Advocate or Coach | - Contacts the incoming chair; typically, an executive coach, outside mentor, or external chair fills this role - Begins work on individualized short-term and long-term plans to help the incoming chair develop leadership and management skills |
| **First 30 Days** | | |
| **✓** | **Person Responsible** | **Tasks** |
|  | HR | - Assists with completing benefits selections - Checks in with the new chair - Checks in with the dean |
|  | Department Administrator | - Helps coordinate life needs, such as childcare, pet care, banking, personal services, schools, and faith communities |
|  | IT | - Assists with phone and technology equipment setup and basic use |
|  | Dean | - Gives the new chair a tour of the campus - Makes introductions to key stakeholders - Attends the first faculty introduction meeting - Makes funds available for a department retreat - Makes funds available for professional development activities |
|  | Search Chair | - Blocks off time to spend with the new chair - Reflects on the search process and brings it to closure |
|  | Peer Mentor | - Blocks off time to spend with the new chair - Is available to answer questions, discuss concerns, and help with problem-solving, as needed |
|  | Advocate or Coach | - Blocks off time to spend with the new chair - Provides coaching and support, as well as insight and feedback in team dynamics and organizational politics - Offers guidance for developing relationships |

**References:**

1. Ross WE, Huang KHC, Jones GH. Executive onboarding: ensuring the success of the newly hired department chair. *Academic Medicine*. 2014;89(5):728-733. <https://doi.org/10.1097/ACM.0000000000000214>
2. Hiring the Best Talent. Association of American Medical Colleges. Accessed February 27, 2024. <https://www.aamc.org/career-development/leadership-development/recruiting>
