# Supplementary material for: Implementing a Holistic Review Toolkit for Faculty Recruitment and Retention
Source: MedEdPORTAL. 2024 Dec 4;20:11472. doi: 10.15766/mep_2374-8265.11472 (PMC11615027; doi:10.15766/mep_2374-8265.11472)
Supplement: Supplementary file 1 — Faculty Pilot Overview.docxOverview Equity-Minded Hiring_Step 1.docxAssess Readiness for Equity-Minded Hiring_Step 1.docxStaff Composition Inventory_Step 2.xlsxHolistic Search Committee Phases and Steps_Step 2.docxFaculty Workshop Facilitators Guide_Step 3.docxFaculty Workshop Presentation_Step 3.pptxFaculty Workshop Evaluation_Step 3.docxFaculty Workshop Activities_Step 3.docxJob Description Posting Tools and Resources_Step 4.docxInterview Questions Tools and Resources_Step 4.docxSubmission Requirements and Rating Tools_Step 4.docx360-Degree (Multisource) Reference Checking_Step 4.docxSearch Process Tools and Resources_Step 5.docxStanding Up a Search Committee_Step 5.docxMitigating Bias Resources_Step 5.docxOnboarding Tools and Resources_Step 6.docxCareer Development Discussion Guide_Step 6.docxU Colorado SOM Mentoring Resource Packet_Step 6.docxBaylor College of Medicine Exit Resources_Step 6.docxU Colorado SOM Equitable Hiring Tool_Step 7.docxHolistic Hiring and Retention Tracker_Step 8.docxEvaluation Materials Development Phase_Steps 4-6.docx [file mep_2374-8265.11472-s001.zip › S. U Colorado SOM Mentoring Resource Packet_Step 6.docx]

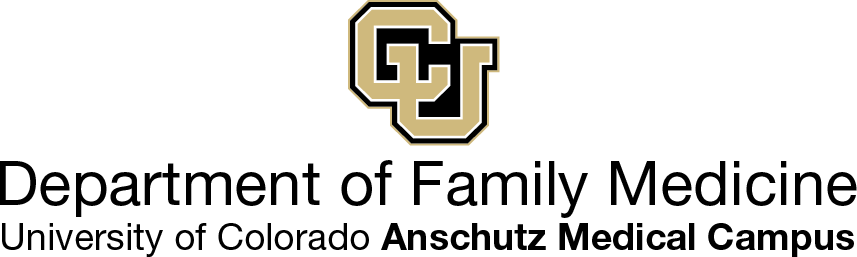


Appendix S

Mentoring Resources

**Implementation Guidance:** The following mentoring resources have been provided as examples and may be modified as necessary for your own needs.

**CONTENTS**

Mentor-Mentee Agreement 3

The First Meeting 5

Sample Meeting Agenda 6

Sample Individual Development Plan 7

Mentor Competencies 10

Goals of Mentoring 13

Communication Tips 14

Motivational Interviewing Tips 15

Coaching Tips 16

Managing Mentor Challenges 17

Harassment/Discrimination 19

Beyond Your Scope 21

Imposter Syndrome 22

Mentoring Articles 23

**UNIVERSITY OF COLORADO DEPARTMENT OF FAMILY MEDICINE MENTOR-MENTEE AGREEMENT**

We, acting as mentors and mentee, agree to enter into a team mentoring relationship based on the criteria described below, which set forth the expectations, parameters, and process for the mentoring relationship.

(mentor’s signature)

(mentor’s signature)

(mentee’s signature)

date / /

**Compact Between Early Career Research Faculty and Their Mentors**

Supporting and developing faculty is an important component of the success of the University of Colorado Department of Family Medicine (CUDFM) and the discipline of family medicine. Faculty typically join an academic department so that they can pursue their own individual part of fulfilling this mission. The faculty member may undertake clinical, scholarship, research, service, and teaching activities that together provide an experience essential for career advancement.

**Core Tenets of Faculty Development—Institutional Commitment**

Institutions that hire early career faculty must be committed to maintaining the highest standards of training and support to provide an environment sufficient to ensure, that when the mentorship is completed, the faculty member can function independently as a scientific professional. Institutional oversight must be provided for terms of appointment, salary, benefits, grievance procedures, and other matters relevant to the support of faculty. A responsible institutional official must be designated to provide this oversight, and a suitable office should be available for the administrative support.

**Importance of Mentoring**

Effective mentoring is critical for faculty development and requires that the primary mentor dedicate substantial time to ensure personal and professional development. A good mentor builds a relationship with the faculty member characterized by mutual respect and understanding. Attributes of a good mentor include being approachable, available, and willing to share their knowledge; listening effectively; providing encouragement and constructive criticism; and offering expertise and guidance.

**Commitments of Mentees**

- **I acknowledge that I have the primary responsibility for the development of my own career.** I recognize that I must take a realistic look at career opportunities and follow a path that matches my individual skills, values, and interests.
- **I will develop a mutually defined professional development plan with my mentor that includes well-defined goals and timelines.** Ideally, this plan should be outlined and agreed upon at the time of the initial appointment.
- **I will show respect for and will work collegially with my mentor, coworkers, support staff, and other individuals with whom I interact.**
- **I will seek regular feedback on my performance and ask for a formal evaluation at least annually.**
- **I recognize that I have embarked on a career requiring lifelong learning.** To meet this obligation, I must stay abreast of the latest developments in my specialized field through reading the literature, regular attendance at relevant seminar series, and attendance at scientific meetings.
- **I will actively seek opportunities outside the department (e.g., professional development seminars and workshops in oral communication, scientific writing, management, and teaching) to develop the full set of professional skills necessary to be successful in my chosen career.**

**Commitments of Mentors**

- **I will ensure that a mutually agreed-upon set of expectations and goals is in place at the outset of the mentoring period, and I will work with the mentee to create an individual career development plan.**
- **I will strive to maintain a relationship with the mentee that is based on trust and mutual respect.** I acknowledge that open communication and periodic formal performance reviews, conducted at least annually, will help ensure that the expectations of both parties are met.
- **I will ensure that the mentee has sufficient opportunities to acquire the skills necessary to become an expert in their area.**
- **I will provide the mentee with the required guidance and mentoring and will seek other faculty and departmental/institutional resources when necessary.** Although I am expected to provide guidance and education in technical areas, I recognize that I must also educate the mentee by example and by providing access to formal opportunities/programs in complementary areas necessary for a successful career.
- **I will provide a training environment suited to the individual needs of the mentee to ensure their personal and professional growth.** I will encourage a progressive increase in the level of responsibility and independence to facilitate the transition to a fully independent career.
- **I will encourage the interaction of the mentee with fellow faculty members both intra- and extramurally and encourage the mentee’s attendance at professional meetings to network and present their work.**
- **I will commit to being a supportive colleague to mentees as they transition to the next stage of their career and, to the extent possible, throughout their professional life.** I recognize that the role of a mentor continues after the formal training period.

*This compact serves as both a pledge and a reminder to mentors and their faculty member appointees that their conduct in fulfilling their commitments to one another should reflect the highest professional standards and mutual respect.*

**THE FIRST MEETING**

| **To-do List** | **Strategies for Conversation** | **Questions to Ponder** |
| --- | --- | --- |
| **1. Take time to get to know each other.** | Obtain a copy of your mentoring partner’s CV in advance of the conversation. If one is not available, create one through conversation. | What kind of information might you exchange to get to know each other better? What points of connection have you discovered in your conversation? What else do you want to learn about each other? |
| **2. Talk about mentoring.** | Share your previous mentoring experiences with your mentoring partner. | What did you like about your experiences that you each want to carry forward into this relationship? What do you want to avoid? |
| **3. Share your learning and development goals.** | Describe your career vision, hopes, and dreams, and articulate broad learning goals and the reasons why they are important. | Why do you want to engage in this relationship? What learning goals would align with your vision of the future? |
| **4. Determine relationship needs and expectations.** | Ask your mentoring partner what they want, need, and expect out of the relationship. | Are you clear about each other’s wants, needs, and expectations for this mentoring relationship? What would be a logical time/outcome to indicate the closure of the relationship? |
| **5. Candidly share your personal assumptions and limitations.** | Ask your mentoring partner about their assumptions and limitations. Discuss implications for your relationship. | What assumptions do you hold about each other and your relationship? What are you each willing to contribute and capable of contributing to the relationship? What limitations do you each bring to the relationship? |
| **6. Discuss your personal style.** | Talk about your personal styles. You may have data from instruments, such as StrengthsFinder, MBTI, DiSC, etc. | How might each other’s styles affect the learning that goes on in the mentoring relationship? |

**SAMPLE MEETING AGENDA**

“The One-Minute Mentor”

1. Assess the mentee (use active listening skills)
   - Check in
   - Assess for any urgent issues
2. What’s on the agenda?
   - Review pending items
   - Assess time available
   - Prioritize
3. Assist with ongoing projects

- Ask clarifying questions
- Set clear and measurable goals
- Give advice and suggest resources
- Agree on timeline for deliverables

1. Provide career guidance

- Review Individual Development Plan and CV
- Inquire about professional/personal balance

1. Wrap up

- Clarify expectations of mentor and mentee
- Schedule future meeting

**Source:**

Developed by Mitchell D. Feldman, MD, MPhil

Resource: Powered by CTSI at UCSF

**SAMPLE INDIVIDUAL DEVELOPMENT PLAN (IDP)**

1. **Name**
2. **Date**
3. **Academic Series and Rank**
4. **Primary Mentor**

**Additional Mentors**

1. **Identify Personal and Institutional Long-term Goals**

*Why did you decide to work at a medical school?*

*What do you personally hope to accomplish in your career?*

*List other goals discussed with your supervisor.*

1. **Areas of Focus: Definition and Distribution of Effort**

The following five areas of focus generally describe the areas where faculty direct their efforts to successfully accomplish their personal, institutional, and academic series goals.

- - **Teaching**—Excellence in Education

Teaching, student advising, continuing medical education (CME), new course development

- - **Research/Scholarship/Creative Activity—**Leadership in Innovative Research

Conducting basic science and/or clinical research, presentations, publications, application for and receipt of grant support, copyrights and patents, editing, peer review

- - **Clinical Care**—State-of-the-Art Clinical Care

Direct patient care, chart review, related clinical activities, clinical budget performance

- - **Service**—Leadership in Governance

Participation or leadership in governance, committee membership, collegial activities (suggested service priority: department, SOM, UCDHS, university, professional, and community)

- - **Self-Development**—Networking, Work-Life Balance, and Additional Mentors

Faculty development activities, leadership programs, CME training, earning advanced degrees, participation in professional academic associations or societies, developing professional contacts, consulting in one’s field, expanding network contacts, balancing work and personal life, utilizing additional mentors in specific areas of focus.

**Current Distribution of Effort**

Estimate the hours per week spent in each focus area, then list the percentage of total duties.

| **Focus Area** | **# Hours/Week** | **% of Total Duties** |
| --- | --- | --- |
| Teaching |  |  |
| Research |  |  |
| Clinical Care |  |  |
| Service |  |  |
| Self-Development |  |  |
| **Total** |  |  |

1. **Specific Goals in Focus Areas**

Complete the focus areas that specifically apply to the criteria for your academic series and will help you accomplish your personal and institutional long-term goals.

**Teaching**

Year in Review: Please list last year’s goal(s) and significant accomplishments (teaching appointments, invitations, course or program improvements, etc.). If the goals were not met, explain and identify barriers.

*Upcoming year’s teaching goal(s):*

*Identify resources, collaborators, and time commitment needed to achieve goal(s):*

*Identify barriers to achieving new goal(s):*

**Research/Creative Activities**

Year in Review: Please list last year’s goal(s) and significant accomplishments (major publications, grants, presentations, invitations, etc.). If the goals were not met, explain and identify barriers.

*Upcoming year’s research goal(s):*

*Identify resources, collaborators, and time commitment needed to achieve goal(s):*

*Identify barriers to achieving new goal(s):*

**Clinical Care**

Year in Review: Please list last year’s goal(s) and significant accomplishments (exceptional patient care, development of new techniques, clinical programs, etc.). If the goals were not met, explain and identify barriers.

*Upcoming year’s patient care goal(s):*

*Identify resources, collaborators, and time commitment needed to achieve goal(s):*

*Identify barriers to achieving new goal(s):*

**Service**

Recommended service priority: department, school, university, professional, and community.

Year in Review: Please list last year’s goal(s) and significant accomplishments. If the goals were not met, explain and identify barriers.

*Upcoming year’s administration goal(s):*

*Identify resources, collaborators, and time commitment needed to achieve goal(s):*

*Identify barriers to achieving new goal(s):*

**Self-Development** (Networking, Work-Life Balance, Additional Mentors)

Year in Review: Please list year’s goal(s) and significant accomplishments. If the goal were not met, explain and identify barriers.

*Upcoming year’s self-development goal(s):*

*Identify resources, collaborators, and time commitment needed to achieve goal(s):*

*Identify barriers to achieving new goal(s):*

1. **Optimal Distribution of Effort**

Revisit the table, “Current Distribution of Effort,” in step 6. Create a new Optimal Distribution of Effort table, taking into account your specific goals listed in step 7.

| **Focus Area** | **# Hours/Week** | **% of Total Duties** |
| --- | --- | --- |
| Teaching |  |  |
| Research |  |  |
| Clinical Care |  |  |
| Service |  |  |
| Self-Development |  |  |
| **Total** |  |  |

**Mentee Date**

**Mentor Date**

Adapted from IDP form presented by Russell G. Robertson, MD, Medical College of Wisconsin. 2004 AAMC Faculty Affairs Professional Development Conference. [www.ucdmc.ucdavis.edu/facultydev/docs/](http://www.ucdmc.ucdavis.edu/facultydev/docs/)NewCareerMntrgIDP.r

**MENTOR COMPETENCIES**

| **Thematic Areas** | **Competencies** | **Examples** |
| --- | --- | --- |
| **Cultural and Identity Awareness** | Attend to cultural diversity issues | - Understand how individual differences and cultures influence mentoring relationships - Communicate effectively across diverse dimensions, including varied backgrounds, disciplines, ethnicities, positions of power, and styles |
| **Communication and Relationship Management** | Align expectations  Address availability and accessibility  Enhance interactional skills | - Align and communicate mutually beneficial expectations for mentoring relationship - Make explicit institutional and discipline-specific norms, standards, and expectations - Demonstrate commitment to mentee/make mentee a priority (e.g., being available for meetings as needed, providing prompt feedback to emails, grants, papers) - Establish frequency of meetings and interim communication modes - Engage in active listening - Ask probing questions - Actively promote feedback - Effectively manage conflict and disagreement - Take into consideration perspectives and realities other than mentor’s own - Foster a sense of belonging and trust - Effectively negotiate dialogue across diverse backgrounds, disciplines, departments, generations, ethnicities, and positions of power |
| **Psychosocial Support** | Serve as role model  Establish capacity to reflect on and enhance relationship  Encourage peer mentoring | - Provide work-life balance guidance - Demonstrate positive leadership skills (e.g., demonstrate how to build a research team and manage a lab and how to recruit, hire, and retain good people) - Tailor amount and type of psychosocial support to the personality, circumstances, and response of the individual mentee - Acknowledge and celebrate mentee's successes - Empower mentee to seek guidance and help from other scholars at their level of training |
| **Career and Professional Development** | Prepare and guide implementation of a career development plan  Guide development of professional skills  Provide information and guidance to navigate academic/institutional environment | - Identify gaps in mentee's knowledge in order to create a realistic career development plan - Adjust mentee's responsibilities over time to foster their independence - Develop strategies for and guide mentee on professional skills and career development - Help mentee understand the fiscal realities of an academic career (e.g., negotiations with industry, working with contracts and grants, PI responsibility for fiscal management) - Actively promote mentee within institution and discipline - Help mentee navigate institutional systems |

Adapted from: Abedin Z, Biskup E, Silet K, et al. Deriving competencies for mentors of clinical and translational scholars. *J Clin Transl Sci.* 2012;5(3):273-280[.](http://onlinelibrary.wiley.com/doi/10.1111/j.1752-) <https://doi.org/10.1111/j.1752-8062.2011.00366.x> .

**Characteristics of Effective Mentors:**


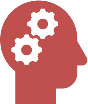

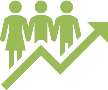

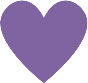


| **Competence** | **Confidence** | **Commitment** |
| --- | --- | --- |
| Professional knowledge and experience | Shared network of contacts and resources | Altruistic |
| Respect | Allows mentee to develop their own skills | Generous with time and opportunities |
| Interpersonal skills and good judgement | Demonstrates initiative | Knows limits |
|  | Shares credit |  |

| **ATTRIBUTES OF A GOOD MENTOR** | **ATTRIBUTES OF A GOOD MENTEE** |
| --- | --- |
| - Has a sincere desire and commitment to be a mentor | - Is motivated - Receives feedback effectively and graciously |
| - Is a good listener | - Is curious and creative |
| - Provides both critically positive and negative feedback (supportive and challenging) | - Is a good listener - Is responsible and responsive |
| - Is flexible and able to adapt to mentee’s individual needs | - Is able to work independently as well as in a team |
| - Is available and engaged | - Is resilient and open to multiple outcomes |
| - Stays mindful of the needs of the mentee beyond formal mentor meetings | - Takes an active role in their own learning and professional development |
| - Is empathic to the mentee’s personal and professional needs and cultural background | - Actively assesses and communicates their changing needs |
| - Shares/finds some common goals or background with the mentee | - Seeks/finds additional informal mentors - Is humble and self-reflective |
| - Serves as a role model - Feels/finds benefit in the relationship with the mentee | - Reflects on how their own cultural background and personal history influence mentoring relationships |
| - Problem-solves collaboratively |  |
| - Provides career coaching |  |
| - Is respected by peers and leadership |  |
| - Celebrates the mentee’s accomplishments |  |
| - Is humble and self-reflective |  |
| - Reflects on how their own cultural background and personal history influence mentoring relationships |  |

**Differentiating a Mentor from Other Similar Roles:**


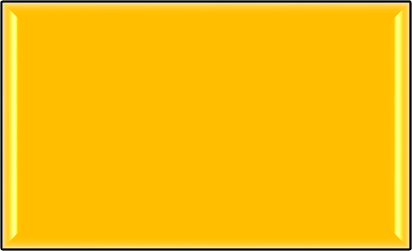


Mentor

- Longitudinal/sustained
- Broad scope
- Reciprocal


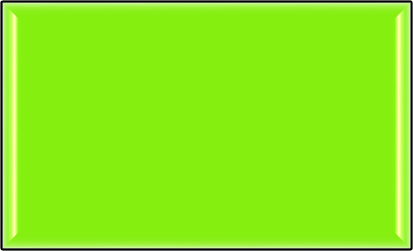


Advisor

- Less ongoing
- Gives advice on specific topic as needed


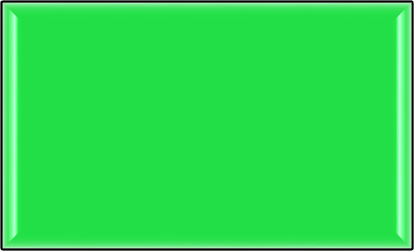


Sponsor

- Talks ABOUT you
- Recommends you for positions or awards
- Aids in obtaining opportunities


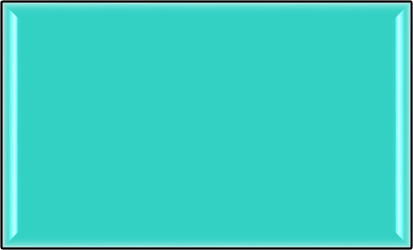


Coach

- Gets YOU to talk
- Feedback


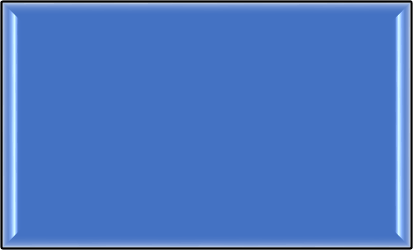


Preceptor

- Teacher/supervisor
- More directive
- Grades/assesses

**Sources:**

- The University of Wisconsin Department of Family Medicine and Community Health’s Mentorship Toolkit.
- “Becoming a Better Mentor,” presentation, Linda Montgomery, MD, FAAFP

**GOALS OF MENTORING**

The benefits of mentoring for both mentors and mentees are well documented in the literature. Effective mentoring programs also contribute to organizational goals, including:

- - Reducing employee turnover
  - Increasing engagement among employees
  - Planning for leadership succession
  - Promoting diversity and inclusion
  - Attracting and retaining top talent
  - Building up future leaders and high-potential employees

**Goals for Mentors**

- - Growing their leadership skills
  - Developing a reputation as an advisor and guide for others
  - Strengthening their emotional intelligence and communication skills
  - Gaining new perspectives
  - Transferring knowledge to others

**Goals for Mentees**

- - Skill development
  - Career planning
  - Networking
  - Learn the workplace culture
  - Gain visibility for potential promotions
  - Problem-solving skills


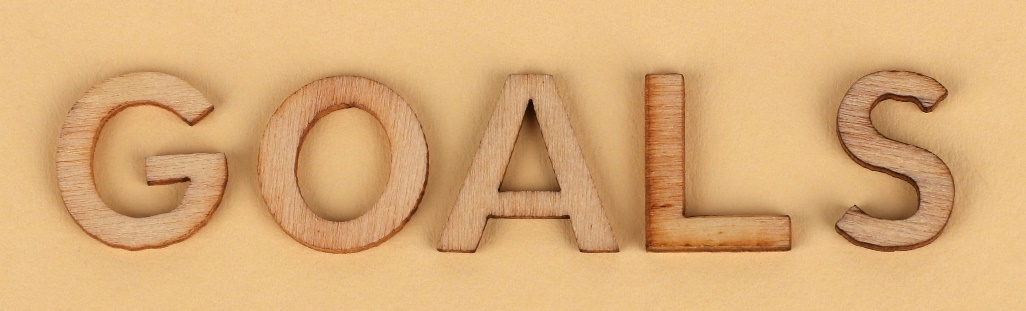


**Source:**

Reeves M. Examples of mentoring goals: for mentors, mentees, and organizations. Together. July 14, 2021. <https://www.togetherplatform.com/blog/examples-of-mentoring-program-goals>

Image by Ann H. retrieved from <https://www.pexels.com/photo/the-word-goals-from-wooden-letters-15351349/> Image is in the public domain.

**COMMUNICATION TIPS**

**Four key skills for effective communication in mentoring relationships are:**

- **Increase your awareness of yourself and others.** You are the instrument through which mentoring happens. The clearer you are about your own agenda and able to separate your own thoughts, feelings, and wants from those of your mentor/mentee, the greater the potential for intentional partnership and mutual benefit.
- **Get curious about the other person’s story.** Listening to learn something new (rather than to confirm what you already know) is essential to good mentoring. When you get curious about the other person’s story, you open up the possibility of greater connection and value for both parties.
- **Listen for passion and potential.** Effective communication in mentoring requires understanding what makes the other person tick, what has brought them to this moment in their career, and where they would like to go next.
- **Share your own crystallized experience.** One of the pleasures of mentoring is the chance to share one’s own hard-earned experience so that it might be helpful to others coming along a similar path.

**Individuals bring a wide range of different life experiences to their mentoring relationships.**

These principles can help mentors and mentees bridge potential differences to create satisfying relationships:

- **Be aware of your own assumptions.** In the same way that others may have different points of view because of differences in their life experiences, you likely have been shaped by your gender, race, social class, education, generation, geography, and a multitude of other cultural influences. Increasing your awareness of the ways you are a product of your past can help you avoid assuming that others see the world in the same way.
- **Get curious about the background and unique perspectives of colleagues who have different life experiences.** Putting yourself in other people’s shoes and seeking to understand how they may have come to their different points of view is a critical step in building a mentoring relationship.
- **Address differences openly.** Relationships in which it becomes comfortable to talk about and acknowledge differences have much greater potential value for both mentor and mentee. While it may initially feel uncomfortable to talk about topics such as race, gender, and/or socioeconomic background, the potential for increased understanding and connection makes it worth the risk.

**Communication best practices**

- Listen attentively first and avoid immediately arguing or rejecting the feedback.
- Provide relevant background information and explanation.
- Ask for clarification or specific examples if you need more information.
- Be clear about what is being said and try to avoid jumping to conclusions.
- Paraphrase the feedback to make sure you have understood the feedback before you respond to it:
  - “What I understood was .”
  - “What I hear you say is if , I __”
- Take the opportunity to check feedback with others and gain diverse perspectives.
- Ask for feedback if you are not receiving the feedback you need to help you achieve your goals.

**Sources:**

- The University of Wisconsin Department of Family Medicine and Community Health’s Mentorship Toolkit.
- Mentors: Cultivation Phase Resources. UW Institute for Clinical and Translational Research Mentoring. [https://ictr.wisc.edu/mentoring/mentors-](https://ictr.wisc.edu/mentoring/mentors-cultivation-phase-resources/) [cultivation-phase-resources/](https://ictr.wisc.edu/mentoring/mentors-cultivation-phase-resources/)

**MOTIVATIONAL INTERVIEWING TIPS**

Motivational interviewing is a brief counseling technique that can be effective at helping people overcome reluctance and make behavioral changes.

The **OARS** acronym highlights four essential aspects of motivational interviewing.

- **Ask open-ended questions instead of “yes” or “no” questions.**
- **Offer affirmations.** As you talk to the person, take joy in their successes and express empathy during tough spots. Affirmation will do more to encourage people to keep moving forward than persuasion or correction will ever do.
- **Practice reflective listening.** Reflective listening involves letting the other person express their thoughts and then, instead of telling them what to do, capturing the essence of what they have said and helping them arrive at an idea or solution.
- **Summarize the conversation.** This involves recapping what the person has said, calling attention to the salient elements of the discussion, and allowing the person to correct any misunderstandings. End the summary with an open-ended statement such as “I am wondering what you think your next step should be.”

The **RULE** acronym outlines these four guiding principles:

- **Resist** the righting reflex—you can’t shame/correct them into it.
- **Understand** the person’s own motivations—what is causing them to be stuck?
- **Listen** with empathy—kindness goes much farther than judgment.
- **Empower** the person—it’s up to them to figure it out, although you can guide them.

**Additional resources:**

- <https://psychwire.com/motivational-interviewing> (10-hour/6-week class)
- <https://www.mcgill.ca/familymed/files/familymed/motivational_counseling.pdf>
- <https://positivepsychology.com/motivational-interviewing/>
- <https://motivationalinterviewing.org/understanding-motivational-interviewing>

**Sources:**

- <https://www.aafp.org/journals/fpm/blogs/inpractice/entry/motivational_interviewing.html>
- <https://www.racgp.org.au/afp/2012/september/motivational-interviewing-techniques>

**COACHING TIPS**

Coaching and mentoring are not the same thing, but many of the same principles apply. Here are some tips:

**Ask good questions**.

Great questions lead to great answers, and great answers lead to great conversations. It is critical that as a mentor, you develop a strong relationship with your mentee. Here are some good coaching questions:

- What’s the biggest challenge you’re facing right now?
- What is your ideal outcome?
- Where are you now?
- Where do you see yourself in a year?
- What is getting in your way?
- Who could help right now?
- What are the problems with your approach?
- What might work better?

**Take a positive approach.**

Try to maintain a positive outlook on things, even in tough situations. A positive attitude toward situations will help both you and the person you’re coaching respond and react in a way that fosters positive change.

**Listen and empower.**

Coaching requires both encouragement and empowerment. Mentors must work with employees to build one-on-one relationships that result in improved performance. Your mentees are likely to have a lot of input, questions, and feedback. It’s important for them to know you care enough to listen and encourage them to share their opinions.

**Know how to guide conversations.**

This is where communication skills and emotional intelligence really come into play. Mentors must guide conversations by asking questions and listening, not by giving directives. Employees learn and grow the most when they uncover the answers themselves.

**Commit to continuous learning.**

Make a commitment to improve your own skills and competencies. If you’re not continuously learning, why should your mentee? Ask questions about where they see their career going or how they see their role evolving in the company. Even if they don't have a plan laid out yet, these questions will make them think about their career and what they want to accomplish within the organization. Show your mentees that you don’t just want them to do better so you look better but that you’re actively interested in their career, accomplishments, and success.

**Coach in the moment.**

Learning is best when things are occurring in the moment. If an employee comes to you with a question about a process or protocol, use this opportunity to teach them something new. Most people learn best by doing, so coach as you go! If you’re busy at the moment, try to schedule some time for later that day. They will appreciate that you took the time to show them how it’s done, and they can now coach others who have the same question.

**Additional Resources:**

- <https://positivepsychology.com/coaching-skills-managers-leaders/>
- <https://www.flashpointleadership.com/blog/tips-for-managers-to-become-a-better-coach>

**Sources:**

- <https://www.td.org/professional-partner-content/7-coaching-tips-for-managers-and-leaders>
- “Becoming a Better Mentor,” presentation, Linda Montgomery, MD, FAAFP

**MANAGING MENTOR CHALLENGES**


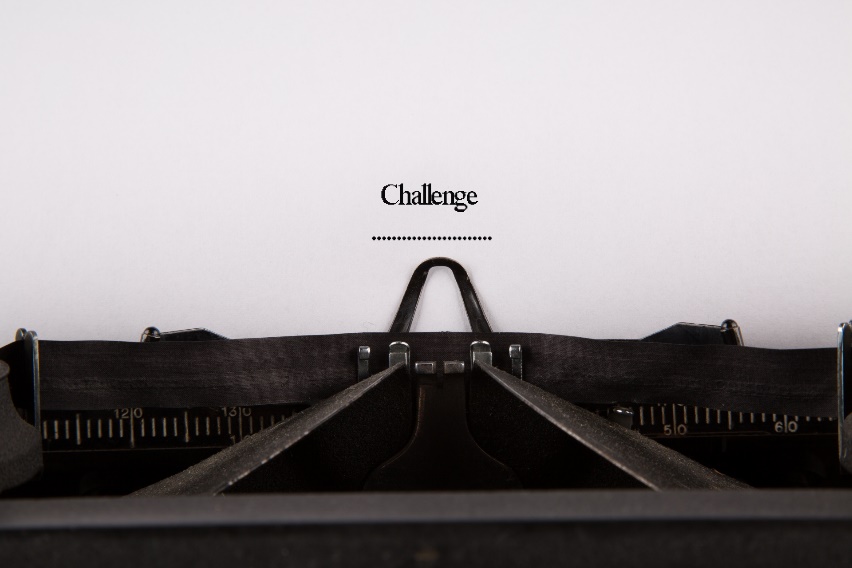
*Whenever people work together, there are bound to be times when the relationships are challenged.* *In healthy situations, the issues are discussed objectively, and each individual is empowered to state their position and feel confident that the other is genuinely listening and wanting to understand. Possible solutions are explored with open minds, and the potential effects of the solutions are considered and weighed. In this section there are some problems that mentors and mentees may encounter and suggested potential strategies for resolving each problem*.

**Mentee seen as lacking commitment.**

**Problem:** A mentor may believe that their mentee lacks the motivation and commitment to carry out the considerable work required to develop a successful career in academia. This situation is difficult for both parties because the mentee has a real chance of failing and because the mentor may believe that they have wasted a great deal of valuable time working with the mentee. At the same time, it is also possible that the mentee believes that the mentor lacks commitment to the mentee’s career. The mentee’s frustrations and lack of guidance can inhibit their movement toward independence. Because of the differential in power between the mentor and mentee, this problem is difficult to resolve while maintaining a productive relationship.

**Strategy:** If a mentee is viewed as lacking commitment, it is important for the mentor to try to discern the cause. It may be that the match is not working well, or it may be that the mentee has discovered that their career focus is no longer appealing. Individuals who choose academic careers tend to be highly motivated, so while there may be an occasional case in which there is a real lack of commitment, there is usually another underlying cause, and it is the mentor’s job to identify it and help resolve it. This could include issues outside of work.

**Mentee seen as having inappropriate attitude.**

**Problem:** Some mentees expect too much from their mentors—demanding more time and attention than they actually need. Others may expect to control their mentors.

**Strategy:** Be firm with your mentee about commitments and responsibilities. If you give your mentee an assignment or deadline, don’t accept excuses for poor work or missed deadlines (unless the excuses are beyond the mentee’s control). In terms of social etiquette, you must be supportive of your mentee and sensitive to cultural differences. For example, in some cultures, there is a preference for modesty, reserve, and control, whereas with another culture, directness or emotionally intense, dynamic, and demonstrative behavior is considered appropriate.

**Your style of mentoring may not match the needs of your mentee.**

**Problem:** Your mentoring style has a lot to do with who you are and how you work. If you are a detail-oriented person, you probably tend to give extensive directions or outline each step of an assignment. If you are a person who tends to see the big picture, you probably are more inclined to give looser, perhaps even vague directions to your mentee. Of course, noting these differences does not make one style better than the other. However, differences in styles between you and your mentee can pose an obstacle. Frustration may also occur when you don’t adapt your style to meet the developing needs or growing independence of your mentee.

**Strategy:** You need to adjust your mentoring techniques to keep in sync with your mentee’s evolution. In time, detailed directions or certain problem-solving strategies may be considered stifling by your developing mentee. Consider giving less and accepting more from your mentee. To meet your mentee’s needs, you need to periodically evaluate your mentee by considering the knowledge, skills, abilities, and traits of your mentee; the level of your mentee; and the needs of your mentee. Once you evaluate your mentee and discover the required amount of guidance, you can determine what style is appropriate for your mentee.

**Mentoring Misalignment**

Signs of mentor/mentee misalignment:

- Mentee and/or mentor dread(s) attending mentor meetings.
- Mentor does not find the time to meet as agreed upon.
- Mentor does not respond to written documents (grants, emails) in a timely manner.
- Mentee does not follow through on deadlines.
- Mentee does not feel a sense of belonging within the professional culture.
- Mentee’s work is successful, but movement toward independence is not being fostered by mentor (e.g., mentor does not give up authorship position, publicly advocate for mentee).
- A sense of shared curiosity and teamwork is not present.
- Mentor does most of the talking and direction-setting during mentoring meetings.
- Mentor or mentee find(s) themselves avoiding the other.
- Mentor and/or mentee avoid(s) eye contact during mentor meetings (can be culturally relative).

**Sources:**

- The University of Wisconsin Department of Family Medicine and Community Health’s Mentorship Toolkit.
- Mentors: Alignment Phase Resources. UW Institute for Clinical and Translational Research Mentoring. [https://ictr.wisc.edu/mentoring/mentors-](https://ictr.wisc.edu/mentoring/mentors-alignment-phase-resources/) [alignment-phase-resources/](https://ictr.wisc.edu/mentoring/mentors-alignment-phase-resources/)
- The Institute for Clinical Research Education Mentoring Resources, University of Pittsburgh. <https://www.icre.pitt.edu/WhatWeDo/mentor.html>.
- Challenging Mentor Relationships. The University of Minnesota, Swenson College of Science and Engineering. <https://scse.d.umn.edu/addressing-challenges> .
- Image by George Hodan. Retrieved from <https://www.publicdomainpictures.net/en/view-image.php?image=172066&picture=challenge> Image is in the public domain.

**HARASSMENT/DISCRIMINATION**

**NONDISCRIMINATION AND SEXUAL MISCONDUCT, INTIMATE PARTNER VIOLENCE AND STALKING**

The University of Colorado Anschutz Medical Campus and Department of Family Medicine are committed to maintaining a positive working environment free of unlawful discrimination and harassment of any kind. In doing so, the university and the department prohibit sexual misconduct, including sexual harassment and discrimination and harassment because of race, creed, color, national origin, religion, sex, age, veteran status, disability, pregnancy, marital status, sexual orientation, gender identity, gender expression, political affiliation or political philosophy, or any other legally protected status.

**Note:** These are example sites from the University of Colorado. Adapt these for your own institution.

See the university’s [nondiscrimination policy](https://www.ucdenver.edu/offices/equity/university-policies-procedures) for more information.

See the university’s [Sexual Misconduct, Intimate Partner Violence and Stalking Policy](https://www.cu.edu/ope/aps/5014) for more information.

All employees are expected to conduct themselves in a professional and businesslike manner at all times and in accordance with [Administrative Policy Statement 2027 Code of Conduct](https://www.cu.edu/ope/aps/2027), [Administrative Policy Statement 5059-](https://www.cu.edu/ope/aps/5059) [Workplace Bullying,](https://www.cu.edu/ope/aps/5059) and [Campus Administrative Policy 4005, Antiviolence Policy](https://www.ucdenver.edu/docs/librariesprovider284/default-document-library/4000-human-resources/4005---anti-violence-policy.pdf?sfvrsn=95ecf3ba_2).

Unlawful harassment includes verbal or physical conduct that is unwelcome or unreasonable and has the purpose or effect of substantially interfering with an individual’s work performance or creating an intimidating, hostile, or offensive work environment. Actions based on an individual’s legally protected status or characteristic will not be tolerated. Prohibited behavior includes, but is not limited to:

- Offensive written materials, including cartoons, emails, posters, drawings, or photographs.
- Verbal conduct, such as epithets, derogatory comments, slurs, suggestive comments, or jokes.
- Physical conduct, such as assault or blocking an individual’s movements.
- Unwelcome sexual advances, requests for sexual favors, and other verbal or physical conduct of a sexual nature.

Such conduct has the purpose or effect of substantially interfering with an individual’s work performance or creating an intimidating, hostile, or offensive work environment.

This policy applies to all employees and non-employees, such as customers, clients, vendors, consultants, etc., regardless of sex, gender, sexual orientation, gender expression, or gender identity.

**HARASSMENT COMPLAINT PROCEDURE**

If an employee is having a conflict with another employee, they should use the following complaint procedure. The Department of Family Medicine expects employees, when possible, to make a timely complaint to enable the department to promptly investigate and correct any behavior that may be in violation of university policy.

1. Employees should feel free to speak with the offending party about conduct that they find offensive and want stopped but are not required to do so. Informal discussions between employees may resolve the problem.
2. If this does not resolve the issue or if an employee doesn’t feel comfortable discussing with the other party, then employees are encouraged to report the situation to the Human Resources Program Director, the Director of Finance & Administration, or the Department Chair, who will discuss the situation with the employee, discuss available campus resources and options for resolution, or take appropriate corrective action. Complaints will be kept as confidential as practicable. The Human Resources Program Director, the Director of Finance & Administration, and the Department Chair are responsible employees under the university’s nondiscrimination and sexual misconduct policies and are obligated to report allegations of discrimination, harassment, and sexual misconduct to the Office of Equity. A report to the Office of Equity does not automatically initiate any formal action; the Office of Equity will contact the individual who is impacted to discuss both formal and informal resolution options and determine the best course of action moving forward.

**Employees may also contact the Office of Equity directly by calling 303-315-2567, via email at** [**equity@ucdenver.edu**](mailto:equity@ucdenver.edu), or by submitting an online report at [**www.ucdenver.edu/offices/equity/resolutions/make-a-report**](http://www.ucdenver.edu/offices/equity/resolutions/make-a-report)**.**

1. The department/university will conduct a prompt, thorough, impartial investigation of the complaint. The investigation will be handled as discreetly as possible.
2. If an investigation is initiated, the accused will be advised of the complaint and will be given the opportunity to provide information about what happened.
3. Other employees who may have relevant information may be contacted and are expected to cooperate with the investigation.
4. If the investigation reveals that there was a violation of policy, prompt disciplinary action up to and including termination will be taken.

**RETALIATION PROHIBITED**

Retaliation against, coercion of, or intimidation of employees who make complaints or who assist in investigations is strictly forbidden. Any employee who engages in such conduct will be subject to discipline up to and including discharge.

**BEYOND YOUR SCOPE**

**So, what do you do if a mentee stumps you or you don’t know how to help them?**

If you have a mentee seeking advice outside your area of expertise, *don’t* drift into the amateur zone and try to muddle along.

- Be transparent about your limitations. It’s OK not to know how to guide someone. You don’t have to have all the answers.
- Identify other sources of information for the mentee—your colleagues in another mission area/department/institution might be able to help. Consider HR or the Ombuds office resources as well.
- Seek other help on campus—for example, the LGBTQ+ Hub has resources for mentoring folks who may be different than you.
- It’s perfectly OK—and recommended!—for mentees to have multiple mentors with varying expertise. A mentee’s web of support could look something like this:


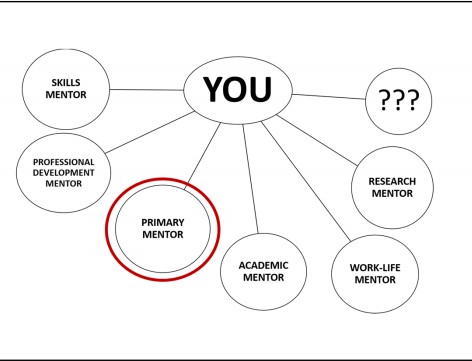


University of Colorado Anschutz Medical Campus owned Image.

**IMPOSTER SYNDROME**

Imposter syndrome is a feeling of self-doubt or incompetence and typically occurs in high achievers who perceive themselves as less competent than others see them. This gap causes feelings of inadequacy, anxiety, and the fear of being found out. This phenomenon can happen at any stage of one’s career and is quite common (up to 80% of people experience it), and research has shown that there are both internal and external reasons. Internal issues like low self-esteem or perfectionistic leanings can contribute to the situation. An example of an external influence is that women and minorities are more likely to experience the imposter phenomenon because of stereotypes and the subsequent discrimination they encounter.

Combating imposter feelings requires addressing both internal and external causes. Acknowledging one’s thoughts and feelings about self-doubt is a crucial first step. Some other tips for dealing with imposter syndrome or helping your mentee with it:

- Ask for feedback but be discerning about constructive criticism.
- Never stop learning; if there is something you want to be better at, find out how to acquire that skill.
- Avoid comparing yourself to others; we all have our individual strengths. Social media is not your friend in this case.
- Ask for help from trusted colleagues. Working in isolation isn’t good for anyone.
- Being humble is OK, even in academia. We all have things we can learn about.
- Addressing imposter syndrome will allow you to reach greater heights in your career.

**Resources:**

- *The Secret Thoughts of Successful Women*, Valerie Young
- *The Gifts of Imperfection*, Brene Brown

**Sources**

- Are You Suffering From Imposter Syndrome? <https://www.visualcapitalist.com/are-you-suffering-from-impostor-syndrome/>
- Combating Imposter Syndrome in Academia. <https://www.facultyfocus.com/articles/philosophy-of-teaching/combating-imposter-syndrome-in-academia/>
- How to Deal With Imposter Syndrome in Academia. <https://www.enago.com/academy/overcome-imposter-syndrome/>
- Imposter Syndrome TED Talk. <https://www.youtube.com/watch?v=bQZzCK5iiGg>
- Yes, Imposter Syndrome Is Real. Here’s How to Deal With It. <https://time.com/5312483/how-to-deal-with-impostor-syndrome/>

**MENTORING ARTICLES**

Gottlieb AS, Travis EL. Rationale and models for career advancement sponsorship in academic medicine: the time is here; the time is now. *Academic Medicine.* 2018;93(11):1620-1623. https://doi.org/10.1097/ACM.0000000000002342

<https://www.ncbi.nlm.nih.gov/pubmed/29979207>

Byyny RL. Mentoring and coaching in medicine. *Pharos*. 2012;75(1):1-3.

<https://www.alphaomegaalpha.org/wp-content/uploads/2021/08/2012-1-Editorial.pdf>

Cullison S. Why mentorship is important to you and to family medicine. *Fam Med.* 2014;46(8):645-646. <http://www.stfm.org/FamilyMedicine/Vol46Issue8/Cullison645>

Leadership development and diversity in academic family medicine: an emphasis on women and underrepresented in medicine. American Academy of Family Physicians.

<https://www.aafp.org/family-physician/patient-care/the-everyone-project/cafm-tool.html>

Levine RB, Ayyala MS, Skarupski KA, et al. “It’s a little different for men”—sponsorship and gender in academic medicine: a qualitative study. *J Gen Intern Med.* 2021;36(1):1-8. https://doi.org/10.1007/s11606-020-05956-2

<https://link.springer.com/article/10.1007/s11606-020-05956-2>

Osman NY, Gottlieb B. Mentoring across differences. *MedEdPORTAL.* 2018;14:10743.

<https://www.mededportal.org/doi/10.15766/mep_2374-8265.10743>

Chopra V, Edelson DP, Saint S. Mentorship malpractice. *JAMA.* 2016;315(14):1453-1454.

[https://medschool.cuanschutz.edu/docs/librariesprovider32/faculty-mentoring-and-promotion/mentorship-](https://medschool.cuanschutz.edu/docs/librariesprovider32/faculty-mentoring-and-promotion/mentorship-malpractice.pdf?sfvrsn=e4da06b9_2) [malpractice.pdf?sfvrsn=e4da06b9_2](https://medschool.cuanschutz.edu/docs/librariesprovider32/faculty-mentoring-and-promotion/mentorship-malpractice.pdf?sfvrsn=e4da06b9_2)

Chopra V, Saint S. 6 things every mentor should do. Harvard Business Review. March 29, 2017.

<https://hbr.org/2017/03/6-things-every-mentor-should-do>

Phillips-Jones L. *75 Things to Do With Your Mentees: Practical and Effective Development Ideas You Can Try.* CCC/The Mentoring Group; 2003.

<https://my.lerner.udel.edu/wp-content/uploads/75-Things-To-Do-With-Your-Mentees.pdf>

10 effective coaching strategies to drive team success. Center for Management & Organization Effectiveness.

<https://cmoe.com/blog/10-effective-coaching-strategies-help-drive-team-success/>
