# Supplementary material for: Implementing a Holistic Review Toolkit for Faculty Recruitment and Retention
Source: MedEdPORTAL. 2024 Dec 4;20:11472. doi: 10.15766/mep_2374-8265.11472 (PMC11615027; doi:10.15766/mep_2374-8265.11472)
Supplement: Supplementary file 1 — Faculty Pilot Overview.docxOverview Equity-Minded Hiring_Step 1.docxAssess Readiness for Equity-Minded Hiring_Step 1.docxStaff Composition Inventory_Step 2.xlsxHolistic Search Committee Phases and Steps_Step 2.docxFaculty Workshop Facilitators Guide_Step 3.docxFaculty Workshop Presentation_Step 3.pptxFaculty Workshop Evaluation_Step 3.docxFaculty Workshop Activities_Step 3.docxJob Description Posting Tools and Resources_Step 4.docxInterview Questions Tools and Resources_Step 4.docxSubmission Requirements and Rating Tools_Step 4.docx360-Degree (Multisource) Reference Checking_Step 4.docxSearch Process Tools and Resources_Step 5.docxStanding Up a Search Committee_Step 5.docxMitigating Bias Resources_Step 5.docxOnboarding Tools and Resources_Step 6.docxCareer Development Discussion Guide_Step 6.docxU Colorado SOM Mentoring Resource Packet_Step 6.docxBaylor College of Medicine Exit Resources_Step 6.docxU Colorado SOM Equitable Hiring Tool_Step 7.docxHolistic Hiring and Retention Tracker_Step 8.docxEvaluation Materials Development Phase_Steps 4-6.docx [file mep_2374-8265.11472-s001.zip › T. Baylor College of Medicine Exit Resources_Step 6.docx]

# Appendix T: Baylor College of Medicine Exit Resources

### Baylor College of Medicine Voluntary Exit Survey

Implementation Guidance: Use this survey to better understand who is leaving and why they are leaving. Inform participants that the survey is voluntary and confidential. Note that this resource is provided as an example and may be modified or tailored to the needs of your institution.

1. **Title**

Instructor

Assistant Professor

Associate Professor

Full Professor

1. **Tenure designation**

Tenure Track

Tenured

Non-Tenure Track

Non-Tenured

1. **What is your primary work location?**

Baylor Clinic

Main Baylor Campus

Baylor St. Luke’s Medical Center

McNair Campus

Jamail Specialty Care Clinic

Ben Taub Hospital

Texas Children’s Hospital

VA Medical Center

Menninger Clinic

Other (please specify)

1. **Department:** In what BCM department or School of Allied Health Sciences, do you hold your primary faculty appointment (e.g., Anesthesiology; Biochemistry & Molecular Biology; Dermatology; Emergency Medicine; Family & Community Medicine; Medicine; Molecular Physiology & Biophysics; Molecular Virology & Microbiology; Molecular & Cellular Biology; Molecular & Human Genetics; Neurology; Neuroscience; Neurosurgery; Obstetrics & Gynecology; Ophthalmology; Orthopedic Surgery; Otolaryngology - Head & Neck Surgery; Pathology & Immunology; Pediatrics; Pharmacology; Physical Medicine & Rehabilitation; Psychiatry & Behavioral Sciences; Radiology; Radiation Oncology; School of Allied Health; Surgery; Urology)?
2. **Years at BCM**

0-2

3-5

6-8

9-11

12-14

15-17

18-20

>20

1. **Please rank the extent to which the following factors influenced your decision to leave BCM.**

|  | Not Applicable | No Influence | Minor Influence | Important Influence | Major Influence |
| --- | --- | --- | --- | --- | --- |
| Career opportunity elsewhere |  |  |  |  |  |
| Lack of promotion opportunities |  |  |  |  |  |
| Uncertain career progression |  |  |  |  |  |
| Lack of transparency in communication and actions from leadership |  |  |  |  |  |
| Balancing work/family life issues |  |  |  |  |  |
| Lack of recognition for contributions to Baylor |  |  |  |  |  |
| Level of financial support for biomedical and/or clinical research |  |  |  |  |  |
| Department/section leadership |  |  |  |  |  |
| Personal reasons |  |  |  |  |  |
| Satisfaction with compensation/benefits |  |  |  |  |  |
| Faculty development programs |  |  |  |  |  |
| Dissatisfaction with work environment |  |  |  |  |  |
| Workload demands |  |  |  |  |  |
| Retirement |  |  |  |  |  |

1. **Please provide any comments or details explaining your reason for leaving.**
2. **Please provide any efforts that were made, or could have been made, to keep you at BCM.**
3. **If you have any concerns regarding ethical conduct at BCM, including legal or regulatory matters, that you would like to discuss, we would like to hear from you. You may leave us a contact number or submit your concerns through the BCM Integrity Hotline toll free at 855-764-7292 or** [www.bcm.ethicspoint.com](http://www.bcm.ethicspoint.com)
4. **The Ombuds Office is available to confidentially listen to any concerns affecting your work or studies at the college and help you explore options for addressing and resolving your concerns. You may leave us a contact number or present your concerns at (713) 798-5039 or** [ombudsoffice@bcm.edu](mailto:ombudsoffice@bcm.edu)

### Baylor College of Medicine Exit Interview Questions

Implementation Guidance: This framework provides an adaptable format for conducting exit interviews.

The general format for our exit interviews is as follows:

1. Introductions
2. Explanation of the interview process (receive their permission to send summary to Dr. Monroe)
3. Ask faculty member about their background and how they came to BCM
4. Ask faculty member to discuss their role within the college
5. Ask faculty member to describe the circumstances leading to their impeding departure from the college
6. Ask the faculty member for thoughts on what could have been done differently or recommendations on how to avoid similar situations in the future (as they are willing)
7. Ask the faculty member if they were asked to remain at BCM and/or if they would consider a return to BCM in the future
8. Thank the faculty member for their service

Interviews typically last 45-60 minutes.
