# Supplementary material for: Implementing a Holistic Review Toolkit for Faculty Recruitment and Retention
Source: MedEdPORTAL. 2024 Dec 4;20:11472. doi: 10.15766/mep_2374-8265.11472 (PMC11615027; doi:10.15766/mep_2374-8265.11472)
Supplement: Supplementary file 1 — Faculty Pilot Overview.docxOverview Equity-Minded Hiring_Step 1.docxAssess Readiness for Equity-Minded Hiring_Step 1.docxStaff Composition Inventory_Step 2.xlsxHolistic Search Committee Phases and Steps_Step 2.docxFaculty Workshop Facilitators Guide_Step 3.docxFaculty Workshop Presentation_Step 3.pptxFaculty Workshop Evaluation_Step 3.docxFaculty Workshop Activities_Step 3.docxJob Description Posting Tools and Resources_Step 4.docxInterview Questions Tools and Resources_Step 4.docxSubmission Requirements and Rating Tools_Step 4.docx360-Degree (Multisource) Reference Checking_Step 4.docxSearch Process Tools and Resources_Step 5.docxStanding Up a Search Committee_Step 5.docxMitigating Bias Resources_Step 5.docxOnboarding Tools and Resources_Step 6.docxCareer Development Discussion Guide_Step 6.docxU Colorado SOM Mentoring Resource Packet_Step 6.docxBaylor College of Medicine Exit Resources_Step 6.docxU Colorado SOM Equitable Hiring Tool_Step 7.docxHolistic Hiring and Retention Tracker_Step 8.docxEvaluation Materials Development Phase_Steps 4-6.docx [file mep_2374-8265.11472-s001.zip › U. U Colorado SOM Equitable Hiring Tool_Step 7.docx]

# Appendix U: Equitable Hiring Tool

# Department of Family Medicine University of Colorado Anschutz Medical Campus

**Implementation Guidance:** Before utilizing this tool, your institution should review federal and local laws to ensure it aligns with organizational policies and procedures. Note that this resource is provided as an example and may be modified or tailored to the needs of your institution.

**A. GENERAL INFORMATION**

Hiring Manager:       Date:

Name:

Job Title Being Reviewed:

**Search Committee**

Does your panel include:

Gender diversity?

Racial diversity?

Organizational diversity (people with different work experience)?

| NAME | JOB TITLE OR GROUP BEING REPRESENTED |
| --- | --- |
|  |  |
|  |  |
|  |  |
|  |  |
|  |  |
|  |  |

**Information to Review Before Using This Tool**

**Note:** These are example sites from the University of Colorado. Adapt these for your own institution.

Review department’s [DFM Diversity and Health Equity Resources](https://medschool.cuanschutz.edu/family-medicine/Diversity-and-Health-Equity#:~:text=The%20University%20of%20Colorado%27s%20Department%20of%20Family%20Medicine,inclusive%20environment%20-%20treating%20every%20human%20with%20respect.)

Review university’s [Equitable Workforce Plan](https://www1.ucdenver.edu/offices/equity/affirmative-action/executive-plan-summaries)

Review Denver Metro [demographics](https://statisticalatlas.com/metro-area/Colorado/Denver/Race-and-Ethnicity)

Review department demographics (available from HR)

Review the DFM Hiring Process FAQ (provided with this tool) <https://www.cityofmadison.com/employeenet/civil-rights/hiring>

Record information you considered from above here:

|  |
| --- |

Did you complete all the tasks in this section?  Yes  No

If not, why not, and what is your plan to address these?

|  |
| --- |

**B. POSITION DESCRIPTION UPDATING**

You should work on updating the position description as soon as you get notice that the incumbent is leaving or on a regular basis. Before looking at the current job description (JD), complete this portion of the tool so that you aren’t overly influenced by what has been in the JD in the past.

**Basic Skills**

|  |
| --- |

**Transferable Skills**

Often when we want to fill a position, we look for someone who had that position or a very similar position with another employer. However, candidates could have gained the skills we are looking for from different positions and, in addition, bring new perspectives to our department. For example, if you are hiring a call center supervisor, someone who supervised employees providing frontline customer service in a retail environment, but not in a call center, might also be a potential fit for the position.

What are some transferable skills that would qualify a candidate for this job even if they haven’t worked in this field or position before?

|  |
| --- |

**Minimum Qualifications**

Based on the listed skills, are there any minimum qualifications?  Yes  No

If so, what are they?

|  |
| --- |

Could any of these be learned on the job?*  Yes  No

Please list.

|  |
| --- |

*If yes, this should be a preferred qualification, not a minimum one.

**Educational Requirements**

Based on the listed skills, are there any minimum education requirements?  Yes  No

If so, what are they?

|  |
| --- |

Are there any potential unintended effects caused by these education requirements? What will you do to mitigate them?

|  |
| --- |

**Experience Requirements**

Based on the listed skills, are there any minimum experience requirements?  Yes  No

If so, what are they?

|  |
| --- |

Are there any potential unintended effects caused by these experience requirements, and what you will do to mitigate them?

|  |
| --- |

**Physical Requirements**

List the physical requirements, including examples of the work performed that justify these requirements.

|  |
| --- |

Can these requirements be accommodated? Does everyone working in the position need to meet these requirements?

|  |
| --- |

Are there any potential unintended effects caused by these physical requirements, and what you will do to mitigate them?

|  |
| --- |

**Travel Requirements**

Does this position require a person to travel? If so, does the person need to have a driver’s license to do this, or do they have the ability to arrange their travel through other means? Remember that requiring a driver’s license has been shown to have an increased negative impact on people of color.

|  |
| --- |

Are there any potential unintended effects caused by this requirement, and what you will do to mitigate them?

|  |
| --- |

**Updating the Position Description**

Does the job description clearly reflect our department's mission, vision, and values? Yes  No

If not, what is your plan to address this?

|  |
| --- |

Does the job description accurately reflect the job you wish the candidate to perform? Yes  No

If not, why not, and what is your plan to address these?

|  |
| --- |

On what date was this position description last updated?

|  |
| --- |

Has it ever been updated using this Equitable Hiring Tool?  Yes  No

If yes, on what date?

Update the existing position description using the information you have listed in Section B. Include language about working with multicultural communities.

Did you complete all the tasks in this section?  Yes  No

If not, why not, and what is your plan to address these?

|  |
| --- |

**C. RECRUITMENT AND ADVERTISING**

**Posting Internal/External**

Should this position be posted internally to university employees only, or should it be open and competitive? What is the rationale behind this decision, and does it minimize negative impacts and unintended consequences for marginalized and underrepresented groups?

|  |
| --- |

If posting the job in this way leads to unintended outcomes for a group that is already underrepresented in the department, discuss why you want to post it this way, and consider your decision.

|  |
| --- |

**Advertising the Position**

How do you plan to target outreach to underrepresented groups with this job posting?

|  |
| --- |

Where do you plan to post the advertisement of this position (in addition to standard locations used for all university job postings)?

|  |
| --- |

Please consult with the HR Team or the Diversity, Equity, and Inclusion Leadership for sources to advertise to underrepresented demographics. Remember that authentic relationships are the best form of advertising.

Did you complete all the tasks in this section?  Yes  No

If not, why not, and what is your plan to address these?

|  |
| --- |

**D. INTERVIEW QUESTIONS DEVELOPMENT**

**Interview Questions**

In developing your interview questions, consider what skills you are looking for that you didn’t find out about through the paper application (CV/resume/cover letter). Make sure you are using behavioral interview questions. Human Resources will provide you with a starter set of questions that may be adapted for this position.

Choose at least one of the DEI questions to assure that all new employees demonstrate their capacity and willingness toward working with multicultural communities and workplace teams. These questions should be benchmarked and scored at the same level of importance as the other questions being asked. What interview question(s) will you use to accomplish this?

|  |
| --- |

**E. CONDUCTING INTERVIEWS AND MAKING A SELECTION**

**Interview Panels**

What strategies or steps will your team take to minimize bias during the interview process?*

|  |
| --- |

*Please contact HR or the Vice Chair of Diversity, Equity, and Inclusion if you need tips and tools to help with this.

Did you complete all the tasks in this section?  Yes  No

If not, why not, and what is your plan to address these?

|  |
| --- |
