# Supplementary material for: Implementing a Holistic Review Toolkit for Faculty Recruitment and Retention
Source: MedEdPORTAL. 2024 Dec 4;20:11472. doi: 10.15766/mep_2374-8265.11472 (PMC11615027; doi:10.15766/mep_2374-8265.11472)
Supplement: Supplementary file 1 — Faculty Pilot Overview.docxOverview Equity-Minded Hiring_Step 1.docxAssess Readiness for Equity-Minded Hiring_Step 1.docxStaff Composition Inventory_Step 2.xlsxHolistic Search Committee Phases and Steps_Step 2.docxFaculty Workshop Facilitators Guide_Step 3.docxFaculty Workshop Presentation_Step 3.pptxFaculty Workshop Evaluation_Step 3.docxFaculty Workshop Activities_Step 3.docxJob Description Posting Tools and Resources_Step 4.docxInterview Questions Tools and Resources_Step 4.docxSubmission Requirements and Rating Tools_Step 4.docx360-Degree (Multisource) Reference Checking_Step 4.docxSearch Process Tools and Resources_Step 5.docxStanding Up a Search Committee_Step 5.docxMitigating Bias Resources_Step 5.docxOnboarding Tools and Resources_Step 6.docxCareer Development Discussion Guide_Step 6.docxU Colorado SOM Mentoring Resource Packet_Step 6.docxBaylor College of Medicine Exit Resources_Step 6.docxU Colorado SOM Equitable Hiring Tool_Step 7.docxHolistic Hiring and Retention Tracker_Step 8.docxEvaluation Materials Development Phase_Steps 4-6.docx [file mep_2374-8265.11472-s001.zip › A. Faculty Pilot Overview.docx]

Appendix A: Holistic Approach to Faculty Recruitment and Retention Overview

**Implementation Guidance**

Before implementing the recommendations in this initiative, your institution should review federal and local laws to ensure they align with organizational policies and procedures. The development of a holistic recruiting and retention process is divided into three phases and eight steps. Please work through the steps in sequence to achieve the best institutional results. This appendix lays out each phase and step of the initiative. You may want to refer to this appendix for guidance as you work through the program.

**Program Overview**

The tools, resources, and instruction provided in this program will guide your institution to:

- Assess your current hiring practices.
- Determine the desired competencies and mission-critical experiences, attributes, competencies, and scholarly metrics (EACMs) an ideal candidate would have.
- Develop interview questions.
- Develop a candidate evaluation form and score sheet.
- Assess your current recruitment and retention metrics for analysis.

**Assemble Your Implementation Team**

Your implementation team should include stakeholders with a deep understanding of institutional context and culture. You will need team members with experience in facilitation, data analysis, project management, and diversity strategic planning. Also, make sure you have representatives who can drive change.

Key stakeholders include, but are not limited to, academic and administrative leaders (viz., deans, chairs), faculty, administrators, human resources, and staff. Support staff will be pivotal to this process for scheduling and record keeping.

The implementation team is needed to perform these key functions:

- Administer holistic review workshop and unconscious bias training.
- Modify current interview questions and candidate evaluation forms.
- Create a process for standardized and coordinated interviews.
- Monitor your progress.
- Set a planning action cycle for monitoring continuous holistic recruitment and retention progress.

**Determine Your Scope**

How many departments or units do you want to review and revise their recruiting and retention policies and practices at your institution or organization?

The Implementation team will draw on their expert knowledge of your institution to determine what the key stakeholder groups will be for each department or unit.

**Develop Your Timeline**

To achieve the goals of this tool kit, you and your team will need to determine a reasonable timeline. Use the following suggestions to guide the development of your timeline.

**Phase I: Education and Investigation**

The assessment and evaluation you will conduct in steps 1 and 2 lay the groundwork for successfully tailoring and delivering the Holistic Principles Workshop (step 3).

**Prework: Getting Ready**

- Assemble your implementation team.
- Select and orient workshop facilitator(s).
- Determine the scope of this project.
- Develop your timeline.

**Step 1: Assess Organizational Climate**

In this step, you will assess your unit’s, department’s, or institution’s climate for inclusivity. This assessment will help you to identify areas in need of attention by looking at the domains where you score three or below on a 5-point scale.

***Organizational Assessment Tools and Resources***

- Appendix B: Overview of Equity-Minded Hiring
- Appendix C: Assessing Readiness for Equity-Minded Hiring

**Step 2: Current Recruitment and Retention Process Evaluation**

In this step, you will take an inventory of your staff composition and review your current search practices against the templates and tools provided. Identify any gaps in your current process and **update your search plan based on holistic principles.**

***Process Evaluation Tools and Resources***

- Appendix D: Staff Composition Inventory
- Appendix E: Holistic Search Committee Phases and Steps

**Step 3: Deliver Holistic Principles Workshop**

In this step, you will need to select people to serve on future search committees and to schedule blocks of time to deliver the workshop to each group of stakeholders. It is important that members of the recruiting group deliver each workshop so that they can define the EACMs appropriate for their group.

***Workshop Tools and Resources***

- Appendix F: Faculty Workshop Facilitators Guide
- Appendix G: Faculty Workshop Presentation
- Appendix H: Faculty Workshop Evaluation
- Appendix I: Faculty Workshop Activities
  - Activity 1 - Identifying Priority Applicant Criteria
  - Activity 2 - Defining Priority Applicant Criteria
  - Activity 3 - Action Plan and Next Steps

**Phase II: Recruitment, Selection, and Retention Materials and Process Updates**

While steps 1-3 in the education and investigation phase were linear, material and process updates can be a circular process. As you begin to rewrite your job descriptions and your interview questions, you may then move to looking at where you are posting your job openings and how you are evaluating candidates. Steps 4-6 are highly interrelated; treat this development phase as a flexible, rather than rigid, process.

**Step 4: Rewrite Job Descriptions, Interview Questions, and Applicant Evaluation Materials**

In this step, the implementation team should meet regularly to review the EACMs identified by each group. Using this information, you will update your job descriptions and interview questions.

***Job Description and Posting Tools and Resources* (Appendix J)**

- BCM Faculty Job Description Guidelines
- BCM Wellness Director Job Description
- UMass Job Posting Sites

***Interview Question Tools and Resources* (Appendix K)**

- BCM Holistic Interview Questions
- Interview Questions to Assess Background in Diversity, Equity, and Inclusion
- UMass Employment Interview FAQs

***Interview and Reference Rating Tools and Resources***

- Appendix L: Submission Requirements and Interview Rating Tools
  - BCM Applicant Submission Requirements and Evaluation Tool
  - BCM Wellness Director Candidate Rating Form
- Appendix M: 360-Degree (Multisource) Reference Checking

**Step 5: Update Search Plan**

In this step, the implementation team should use the EACMs identified by each group to develop and adapt search materials for your recruiting efforts. You will also analyze your hiring packages for equity across similar positions.

***Search Process Tools and Resources* (Appendix N)**

- Ten Characteristics of a Good Search
- Concierge-level Service for the Application Process
- Candidate Feedback Questionnaire Template

***Search Committee Tools and Resources***

- Appendix O: Standing Up a Search Committee
  - Choosing the Search Committee
  - Dean’s Charge for the Search Committee: Preparation Guide
  - Search Committee Code of Conduct
- Appendix P: Mitigating Bias Resources

***Hiring Package Analysis****:*Conduct internal and external, multi-source analyses to determine salary ranges and conduct routine pay equity analyses.

**Step 6: Review and Update Retention Materials**

We recognize that multiple factors contribute to the retention of faculty, many of which are beyond the scope of this pilot. However, we have provided some materials for your review and use, as desired, in the areas of onboarding, career development, and the collection of exit information.

- Appendix Q: Department Chair Onboarding

***Career Development Tools and Resources***

- Appendix R: Career Development Discussion Guide
  - Faculty and Staff Template
  - Department Chair Template
- Appendix S: UCSOM Mentoring Resource Packet
- Appendix T: BCM Exit Resources

**Implementation Phase**

Now it is time to apply all the investigation and development work your team has done. Steps 7 and 8 provide tools, resources, and guidance for ongoing monitoring and continuously improving your processes and outcomes.

**Step 7: Search Committee Selection and Training**

In this step, you will introduce and socialize the new processes, forms, and interview questions aimed at recruiting and selecting candidates with the desired and mission-critical EACMs.

- Appendix U: UCSOM Equitable Hiring Tool

**Step 8: Monitor Progress and Continuous Improvement**

In this step, you will monitor your progress in recruiting and retaining faculty. We recommend meeting on a quarterly basis, but your team can determine what works best for your institution. In this step, you will use your tracker and exit interviews to review and assess your progress. We recommend that you meet annually to determine what, if any, changes need to be made to your recruitment process and what, if any, policy changes need to be made to better retain faculty after recruitment. Communicate your progress broadly across the institution.

- Appendix V: Holistic Hiring and Retention Practices Tracker
