# Supplementary material for: Implementing a Holistic Review Toolkit for Faculty Recruitment and Retention
Source: MedEdPORTAL. 2024 Dec 4;20:11472. doi: 10.15766/mep_2374-8265.11472 (PMC11615027; doi:10.15766/mep_2374-8265.11472)
Supplement: Supplementary file 1 — Faculty Pilot Overview.docxOverview Equity-Minded Hiring_Step 1.docxAssess Readiness for Equity-Minded Hiring_Step 1.docxStaff Composition Inventory_Step 2.xlsxHolistic Search Committee Phases and Steps_Step 2.docxFaculty Workshop Facilitators Guide_Step 3.docxFaculty Workshop Presentation_Step 3.pptxFaculty Workshop Evaluation_Step 3.docxFaculty Workshop Activities_Step 3.docxJob Description Posting Tools and Resources_Step 4.docxInterview Questions Tools and Resources_Step 4.docxSubmission Requirements and Rating Tools_Step 4.docx360-Degree (Multisource) Reference Checking_Step 4.docxSearch Process Tools and Resources_Step 5.docxStanding Up a Search Committee_Step 5.docxMitigating Bias Resources_Step 5.docxOnboarding Tools and Resources_Step 6.docxCareer Development Discussion Guide_Step 6.docxU Colorado SOM Mentoring Resource Packet_Step 6.docxBaylor College of Medicine Exit Resources_Step 6.docxU Colorado SOM Equitable Hiring Tool_Step 7.docxHolistic Hiring and Retention Tracker_Step 8.docxEvaluation Materials Development Phase_Steps 4-6.docx [file mep_2374-8265.11472-s001.zip › B. Overview Equity-Minded Hiring_Step 1.docx]

Appendix B:

Overview of Holistic Review and Equity-Minded Hiring and Retention Practices to Assist in Diversifying the Faculty Workforce

#### David Acosta, MD, AAMC Chief Diversity and Inclusion Officer

Implementation Guidance: Before implementing the recommendations in this document, your institution should review federal and local laws to ensure they align with organizational policies and procedures.

This tool would be helpful for the implementation team to review and discuss early in the process of implementing the Faculty Holistic Review (FHR) program. Using the Five E’s model to address resistance, the implementation team can address barriers and pave the path for successful implementation of the FHR program. This tool could also be a helpful pre-read for participants before their involvement in the FHR workshop in Step 3, as it defines common terms and introduces the FHR model.

A note about framing: This document was last updated in 2019. At that time, the AAMC defined Underrepresented in Medicine (URiM) more narrowly, focusing primarily on race and ethnicity. For the FHR program, we have adopted a more expansive definition of URiM, aligned with the National Institutes of Health's (NIH) definition of underrepresented populations in research.^1^ Consequently, while this document primarily addresses racial and ethnic minority faculty, it offers evidence-based strategies and suggestions that can be broadly applied to all URiM faculty.

## Definition of holistic review^2^ for faculty

- Holistic review is a flexible, individualized way of assessing an applicant’s capabilities by which balanced consideration is given to experiences, attributes, competencies, and metrics (EACMs) and, when considered in combination, how the individual might contribute value as a faculty member.
- Core principles:
  - Selection criteria are linked and aligned with departmental mission, values, and goals.
  - A balance of EACMs (see Appendix 1, p. 6) is:
    - Used to assess applicants with the intent of diversifying the pool,
    - Applied equitably across all applicants, and
    - Grounded in data that provide the evidence supporting the use of selection criteria beyond number of publications, grants, and other metrics that usually influence decision-making.
  - Committee members give individualized consideration to how each applicant may contribute to the department and institution.
  - Race and ethnicity may be considered as factors when consideration is narrowly tailored to achieve mission-related goals.

## Address institutional, departmental, and individual resistance

- “…the first step to successfully diversifying faculty and staff is naming and understanding the nature of institutional and individual resistance to diverse hiring in predominantly White colleges and universities.”^3^
- Studies demonstrate that the best way to manage resistance is to plan for it.
  - All sources of resistance need to be acknowledged and people’s emotions attached to the resistance validated.

## The 12 Most Common Reasons for Resistance^4^

| - Misunderstanding the need to change - Fear of the unknown - Lack of competence - Connected to the old way - Low trust - Temporary fad | - Not being consulted - Poor communication - Changes to the routine - Exhaustion/saturation - Change in the status quo - Benefits and rewards inadequate |
| --- | --- |

- **5-E’s to Address Resistance** (see Appendix 2, p. 7)—one approach to managing resistance.

“Admittedly, addressing resistance to diversity by institutions and individuals is more complex and difficult than inventing short-term fixes, projects, and strategies, but failing to do so will result in only temporary and cosmetic changes in diverse hiring statistics and not in real, long-term diversification of faculty.”^3^

Recruitment must be inextricably linked to retention and attrition in order to actualize the benefits that diversity brings to the organization.

- “...any initiatives to diversify the faculty and staff that do not address hostile institutional and faculty/staff cultures will end up fueling the ‘revolving door’ so common for faculty and staff of color.”^3^
- Shine a flashlight on your **institutional culture and climate**, e.g., climate assessment surveys.
  - Before looking outward towards the available talent pool, it is critical to first look inward, to understand the values, philosophies, and underlying assumptions that create the foundation of your department.
  - You must first know who you are before you can begin the process of communicating your image to the public.
  - Apply the *Foundational Principles of Inclusion Excellence Departmental Assessment Toolkit* (AAMC product) to assess the “lived experiences” within the department.
- What does a faculty profile of success look like for your department? What does it look like for a faculty of color?
- How do minority faculty perceive the brand of your department? Successful organizations have a well-defined brand.
- Targeted recruitment practices are the norm for those institutions successful in diversifying their faculty workforce. They are deliberate and intentional.
- What institutions are you targeting to recruit minority candidates? Myth: “We’d hire more minority faculty, but they’re just not there.”^5^
- Explore attrition rates and exit interview results from underrepresented in medicine (URiM) faculty or URiM faculty that were offered acceptances but withdrew their candidacy—what can be learned?

Leverage the minority faculty you already have.

- Get into the practice of “stay interviews.” (“You are valued. What can I and the department do to keep you here?”).
- What worked in recruiting these faculty members to your academic institution? What promising practices did they encounter when looking for a job?
- What keeps these faculty members at your academic institution? What promising practices are they aware of at other institutions? Remember, URiM faculty from different institutions talk to each other and keep each other informed.
- Consider forming an advisory council made up of URiM faculty to inform department chairs of promising and effective practices.

## Reexamine search committee processes.

#### Search committee chair:

- - How are search committee chairs appointed, e.g., are there a set of qualifications that have been established by the institution that are adhered to?
  - Do you consider performance history on past search committees they have participated in?
  - Do they possess other important skill sets? For example:
    - Do they practice conscious inclusion?
    - Do they practice equity-mindedness?
    - Do they understand implicit biases and their impact on search process?^6^
    - Do they understand the impact of fixed versus growth mindsets?^7^
  - Do you provide **training** for committee chairs (use of case-based scenarios to test their responses)?
  - Do you provide **coaches** for committee chairs to work through difficult situations encountered?

#### Committee members:

- How are search committee members selected? Are there any special criteria they must meet to serve on the committee?
- Is there **balanced representation** on the committee?
- What training is provided for the committee members?
- Are the responsibilities and **expectations** of the department chair and/or the dean clearly stated and documented?
- Is the **charge** to the search committee clear?
- Is there a set of **cultural norms** that the committee is expected to follow during the process? Are they visible at every meeting as a reminder?

#### Equity advisors:

- Have you considered appointing a faculty equity advisor?^8^ By definition, an equity advisor:
- Ensures diversity balance in membership.
- Offers unconscious bias training to all members of the committee.
- Provides recommendations for targeted outreach.
- Monitors applicant pool to ensure a diverse pool of candidates.
- Reviews applicant pool, examines its diversity, and is given the authority by the department chair or dean to make the decision either to move forward with the pool of candidates or to reopen the search.
- Ensures holistic approach to screening applications and letters of recommendation; and
- Monitors for cognitive errors during proceedings, e.g., discussion of screened applicants, interview results, nomination, and selection.

#### Interview panels:

- Is there **balanced representation**?
- Are there **structured interview questions** asked equitably of all applicants?
- Be sure to include **diversity and inclusion questions**, such as:
- “How will you contribute to the diversity of our department? The institution?”
- “Provide some examples on how you have demonstrated this at other institutions.”

#### Diversity statements^9^

- Be explicit and provide directions for the applicant writing the statement regarding what content and topic areas they should cover.
  - “Describe your past efforts, as well as future plans to advance diversity, equity and inclusion.”
  - “For all past activities and experiences, please be specific about the context, your role, scope or level of effort, and the impact that you think you made.”
  - “Describe how you plan to contribute to diversity at this institution, including activities you would pursue and how they would fit into your research area, department, campus, or national context.”
  - Example: See University of California, San Diego, *Guidelines for Applicants Writing Statement.*^10^
- Develop a standardized scoring system for reviewers to use when reviewing these written statements. Don’t assume all reviewers know what to look for.
  - Example: See University of California, Irvine, *Diversity Statement - Example of Ad Language and Evaluation of Statements,* Diversity Statement Evaluation Grid.^11^


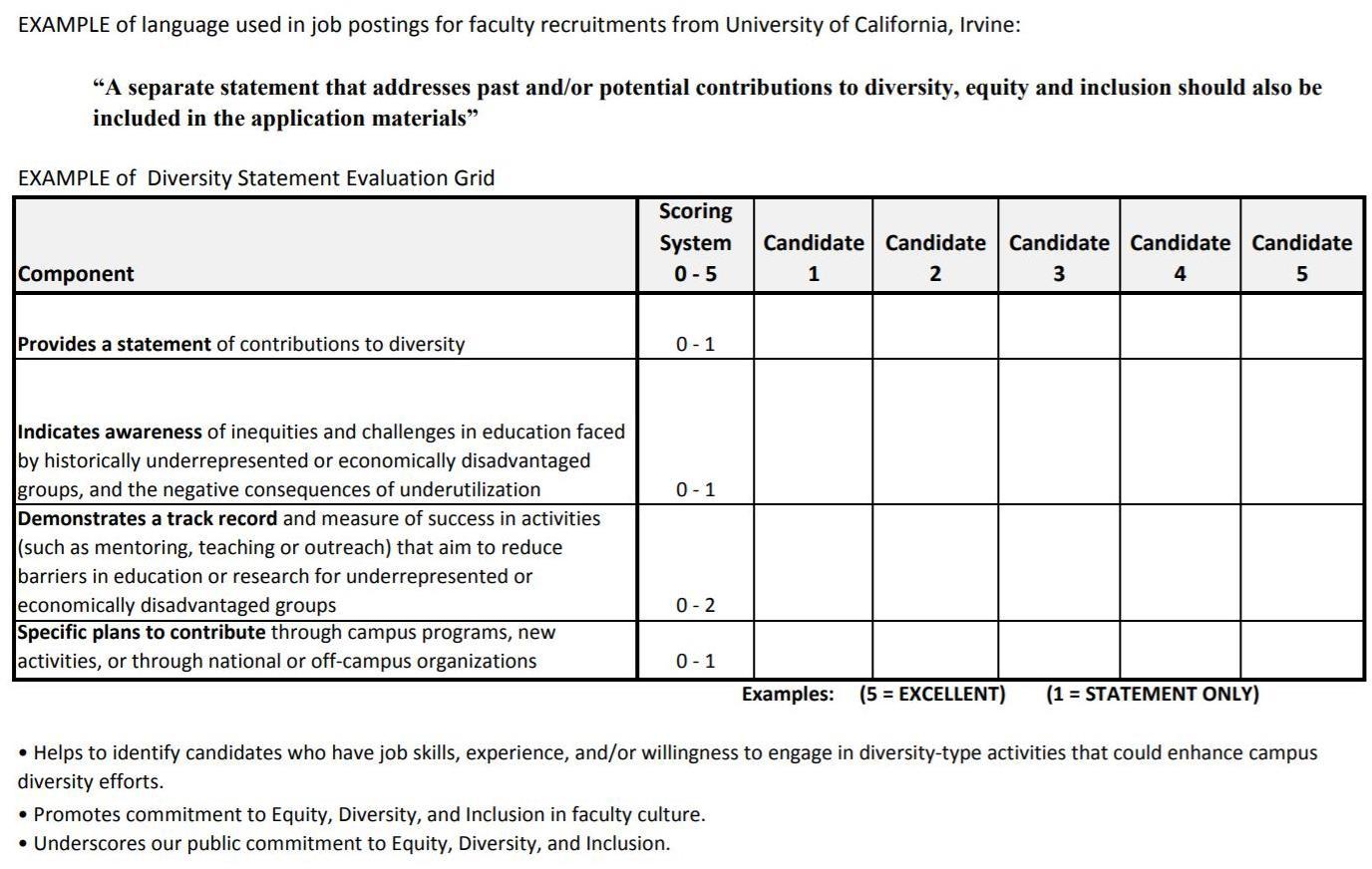


**REFERENCES**

1. Notice of NIH's Interest in Diversity. National Institutes of Health Office of Extramural Research. November 22, 2019. Accessed July 23, 2024. <https://grants.nih.gov/grants/guide/notice-files/NOT-OD-20-031.html>
2. Advancing holistic review. Association of American Medical Colleges. Accessed November 13, 2017. <https://www.aamc.org/initiatives/holisticreview/about/>
3. Kayes PE. New paradigms for diversifying faculty and staff in higher education: uncovering cultural biases in the search and hiring processes. *Multicult Educ.* 2006;14(2):65-69.
4. Torbin R. Top 12 reasons why people resist change. Change Management. May 23, 2011. Accessed November 13, 2017. <https://www.torbenrick.eu/blog/change-management/12-reasons-why-people-resist-change/>
5. Moody J. *Faculty Diversity: Removing the Barriers.* 2nd ed. Routledge; 2012.
6. Kirwan Institute for the Study of Race and Ethnicity. *State of the Science: Implicit Bias Review 2017 Edition.* Kirwan Institute for the Study of Race and Ethnicity; 2017. Accessed April 26, 2019. <http://kirwaninstitute.osu.edu/wp-content/uploads/2107/11/2017-SOTS-final-draft-02.pdf>
7. Dweck CS. *Mindset: The New Psychology of Success.* Random House; 2016.
8. Faculty equity advisors. University of California, San Francisco Office of Diversity and Outreach. Accessed November 13, 2017. <https://diversity.ucsf.edu/faculty-equity-advisor>
9. Golash-Boza T. The effective diversity statement. Inside Higher Ed. June 10, 2016. Accessed November 13, 2017. <https://www.insidehighered.com/advice/2016/06/10/how-write-effective-diversity-statement-essay>
10. University of California, San Diego Center for Faculty Diversity and Inclusion. *Guidelines for Applicants Writing Statement.* Center for Faculty Diversity and Inclusion; date unknown. Accessed March 18, 2018. <https://facultydiversity.ucsd.edu/_files/c2d-guidelines.pdf>
11. University of California, Irvine. *Diversity Statement - Example of Ad Language and Evaluation of Statements.* Columbia University Faculty of Arts and Sciences; no date. Accessed March 18, 2018. <https://fas.columbia.edu/files/fas/content/Example%20of%20Diversity%20Statement%20Ad%20Language%20and%20Evaluation%20Grid.pdf>

**Appendix 1: The AAMC’s Holistic Review Framework’s Experiences, Attributes, and Metrics (EAM) Model**

**Adapted for Faculty Hiring Practices (EACM)**


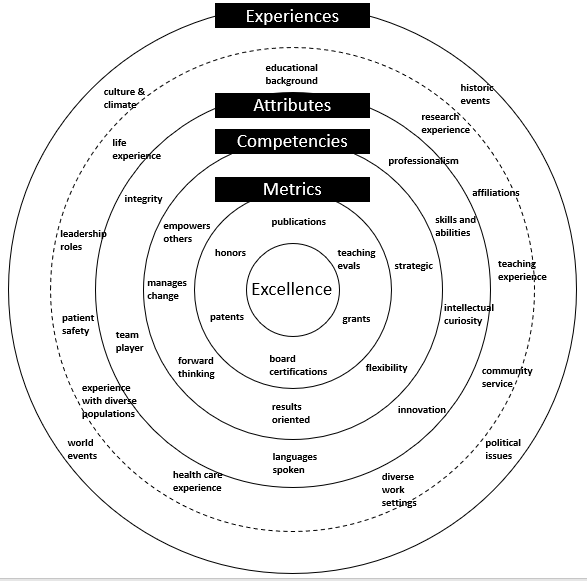


Adapted from Loden M, Rosener JB, *Workforce America: Managing Employee Diversity as a Vital Resource,* McGraw Hill Publishing; 1990.

**Appendix 2:**


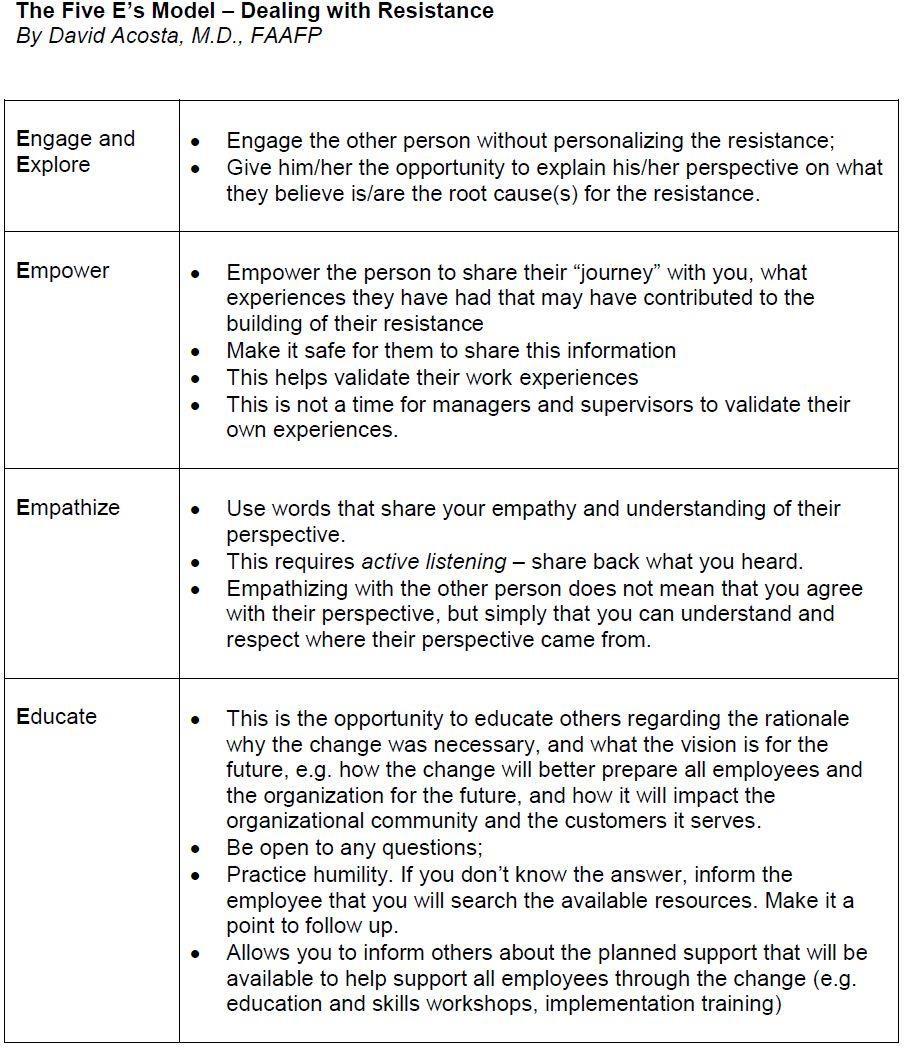


Updated on April 24, 2019.
