# Supplementary material for: Implementing a Holistic Review Toolkit for Faculty Recruitment and Retention
Source: MedEdPORTAL. 2024 Dec 4;20:11472. doi: 10.15766/mep_2374-8265.11472 (PMC11615027; doi:10.15766/mep_2374-8265.11472)
Supplement: Supplementary file 1 — Faculty Pilot Overview.docxOverview Equity-Minded Hiring_Step 1.docxAssess Readiness for Equity-Minded Hiring_Step 1.docxStaff Composition Inventory_Step 2.xlsxHolistic Search Committee Phases and Steps_Step 2.docxFaculty Workshop Facilitators Guide_Step 3.docxFaculty Workshop Presentation_Step 3.pptxFaculty Workshop Evaluation_Step 3.docxFaculty Workshop Activities_Step 3.docxJob Description Posting Tools and Resources_Step 4.docxInterview Questions Tools and Resources_Step 4.docxSubmission Requirements and Rating Tools_Step 4.docx360-Degree (Multisource) Reference Checking_Step 4.docxSearch Process Tools and Resources_Step 5.docxStanding Up a Search Committee_Step 5.docxMitigating Bias Resources_Step 5.docxOnboarding Tools and Resources_Step 6.docxCareer Development Discussion Guide_Step 6.docxU Colorado SOM Mentoring Resource Packet_Step 6.docxBaylor College of Medicine Exit Resources_Step 6.docxU Colorado SOM Equitable Hiring Tool_Step 7.docxHolistic Hiring and Retention Tracker_Step 8.docxEvaluation Materials Development Phase_Steps 4-6.docx [file mep_2374-8265.11472-s001.zip › C. Assess Readiness for Equity-Minded Hiring_Step 1.docx]

Appendix C:

Assessing Department/Unit Readiness for Holistic Review and Equity-Minded

Hiring and Retention Practices to Diversify Faculty Workforce

**Implementation Guidance:** Before implementing the recommendations in this assessment, your institution should review federal and local laws to ensure they align with organizational policies and procedures.

This tool can be used by departments or units prior to implementing holistic faculty hiring and retention processes to assess a baseline. It can then be used at regular intervals (e.g., every year) to assess the extent to which the department or units are implementing more inclusive and equitable recruitment and retention practices. Leaders could also use the tool to set goals on the amount of improvement expected per year and to evaluate progress made.

Instructions: This readiness assessment should be compiled by one individual with input from the team members involved in implementing the changes desired. Complete one readiness assessment per selected school, discipline, or department.

Select the appropriate box for each element using the following Likert scale:

**1** = *Not at all* | **2** = *Starting to think about it* | **3** = *In the beginning stages of development* |

**4** = *Developed but have not yet implemented fully* | **5** = *Completely developed and implemented*

Because this is an institutional change initiative, we recommend focusing your initial rollout on a small number of departments or disciplines.

## Steps:

1. Schedule a one-hour meeting for all team members per selected school, discipline, or department.
2. Designate one person to compile the assessment results.
3. Send the assessment to all team members to be completed before the meeting.
4. In the meeting, discuss each item on the assessment and come to a consensus on the score.
5. Identify areas in need of attention by looking at the domains where you score three or below.

Instructions: Rate your department or unit with respect to each of the following components. Select the appropriate box for each element using the Likert scale below:

**1** *= Not at all* | **2** *= Starting to think about it* | **3** *= In the beginning stages of development* | **4** *= Developed but have not yet implemented fully* | **5** *= Completely developed and implemented*

| **Culture/Climate** | **1** | **2** | **3** | **4** | **5** |
| --- | --- | --- | --- | --- | --- |
| A climate assessment survey of the department has been completed to assess how faculty and staff perceive the department’s culture and climate. |  |  |  |  |  |
| The department has a well-defined brand. |  |  |  |  |  |
| The department has a mission statement that includes how the department values diversity and inclusion (D&I). |  |  |  |  |  |
| The department has a values statement that includes the value of diversity, equity, and inclusion. |  |  |  |  |  |
| The department has a diversity liaison that directs the D&I agenda for the department. |  |  |  |  |  |
| The department has a D&I committee that addresses and monitors all D&I efforts. |  |  |  |  |  |
| **Recruitment** | **1** | **2** | **3** | **4** | **5** |
| The department has developed a faculty profile of success using the holistic review EACM (experiences, attributes, competencies, metrics) approach, aligning with the department's mission and values. |  |  |  |  |  |
| Targeted recruitment practices that include institutions with a high percentage of underrepresented in medicine (URiM) residents, fellows, and faculty are the norm for all department searches, e.g., includes historically Black colleges or universities, minority-serving institutions, Hispanic-serving institutions, centers of excellence. |  |  |  |  |  |
| The department actively studies attrition rates of URiM faculty. |  |  |  |  |  |
| The department routinely performs exit interviews of URiM faculty. |  |  |  |  |  |
| The department has an advisory committee made up of URiM faculty presently in the department to advise the chair and division chiefs on recruitment and retention best practices. |  |  |  |  |  |
| The department has developed and implemented a URiM faculty recruitment and retention toolkit to assist the chair and division chiefs in their recruitment and retention planning. |  |  |  |  |  |
| **Retention** | **1** | **2** | **3** | **4** | **5** |
| The department chair and/or division chiefs perform stay interviews with URiM faculty. |  |  |  |  |  |
| The department has a structured mentoring academy for URiM faculty. |  |  |  |  |  |
| The department leadership regularly discuss potential sponsorship opportunities for their URiM faculty. |  |  |  |  |  |
| The department demonstrates investment in its URiM faculty by promoting and providing professional and leadership development opportunities to enhance their careers, e.g., encourages attending AAMC Minority Faculty Leadership Seminars: Early-Career and Mid-Career; AAMC Grant Writing Workshops; and/or AAMC Group on Diversity and Inclusion (GDI) spring meeting. |  |  |  |  |  |
| **Retention (continued)** | **1** | **2** | **3** | **4** | **5** |
| The department has a designated representative that belongs to the AAMC GDI with the goal of providing the department with information about promising/effective practices in D&I matters and opportunities. |  |  |  |  |  |
| **Search Process** | **1** | **2** | **3** | **4** | **5** |
| The department has a designated equity advisor that is involved in all searches and advises search committees. |  |  |  |  |  |
| Balanced representation of membership on every search committee is the norm. |  |  |  |  |  |
| All members of departmental search committees must participate in required unconscious bias training to serve. |  |  |  |  |  |
| The department has documented specific criteria for the screening of applications describing the necessary characteristics and attributes that are in alignment with the department’s mission and faculty profile of success (EACM). |  |  |  |  |  |
| All applicants are expected to submit a diversity statement that has equal weight with their personal statement, teaching statement, and research statement. |  |  |  |  |  |
| The department chair has granted responsibility and authority to the equity advisor, diversity liaison, or the institution’s chief diversity officer (vice dean of D&I) to review and approve all applicant pools before moving forward with the search and selection process to ensure that there is an appropriate pool of diverse candidates being considered. |  |  |  |  |  |
| Balanced representation of participants on all interview panels is the norm. |  |  |  |  |  |
| All interviews have standardized interview questions that are asked of all interviewees. |  |  |  |  |  |
| Standardized interview questions include questions on D&I, e.g., “How will you contribute to the diversity of our department?” |  |  |  |  |  |
| All search committee members are evaluated by the equity advisor regarding their performance, noted trends in screening, and scoring, and these evaluations are submitted to the department chair (this performance evaluation will be considered in the future regarding whether or not a member will be asked to serve on future search committees). |  |  |  |  |  |
| **All searches track the following information:** | **1** | **2** | **3** | **4** | **5** |
| - Number of URiM applications |  |  |  |  |  |
| - Number of URiM applications that make it into the selected pool |  |  |  |  |  |
| - Number of URiM applicants that are offered an interview |  |  |  |  |  |
| - Number of URiM applicants that complete an interview |  |  |  |  |  |
| - Number of URiM applicants that withdraw their application |  |  |  |  |  |
| - Number of URiM applicants that are offered a position |  |  |  |  |  |
| - Details of the hiring package offered to URiM applicants |  |  |  |  |  |
| - Number of URiM applicants that accept the offer |  |  |  |  |  |
| - Number of URiM applicants that turn down the offer |  |  |  |  |  |
| *Developed by David Acosta, MD, March 22, 2019.* | | | | | |

# Assessing Department Readiness for Holistic and Equity-Minded Hiring and Retention Practices to Diversify Faculty: Interpreting the Results of Your Scoring

| **5** | If most of your responses are in the five range, you are well on your way to a successful and sustainable initiative to diversify your faculty workforce and provide significant attention to their success. It will be of utmost importance to maintain the spirit and the momentum of this initiative, ensure that the individuals leading this effort have the support they need to fully sustain their efforts, and publicly recognize their work. It is also important to share the successful outcomes that this initiative has generated with other departments with the hope of catalyzing their interest in replicating your efforts. This generates the transformational change needed to transform the institutional culture. |
| --- | --- |
| **4** | If most of your responses are in the four range, you probably have several strategies and processes in place that support efforts to diversify your faculty. However, your department may have hit a snag, or there may be a lack of momentum that is prohibiting progression to the next phase of growth. It will be important to pause, reflect, reassess your processes, and identify what promising practices have emerged and what challenges continue to hinder progress moving forward. Do not forget to celebrate any small wins that your team has achieved and be sure to bring these to the attention of your department chair. |
| **3** | If most of your responses are in the three range, you are most likely in the beginning phases of a potentially strong initiative. It will be crucial to develop a strong and sustainable strategic plan aligned with both the departmental and institutional mission and strategic goals and to build a diversity advisory committee/council or a task force appointed by the department chair that has the commitment, has been granted the authority, and has been empowered to implement the plan. |
| **2** | If most of your responses are in the two range, it signifies that there has been or is currently some attempt to support faculty diversity in the department, yet the leadership and/or the majority of the institution are not yet fully engaged, have not fully provided their buy-in, and/or possibly are having difficulty grasping the significance and/or value of having a diverse faculty workforce and connecting it to the department’s potential success. Of concern is the lack of demonstrated commitment to diversifying the faculty. This may mean that the stated commitment to diversity is simply rhetoric and not genuine. At this stage, it is critical to remind the leadership about LCME Standard 3.0, Element 3.3, and the new ACGME core requirement I.C (p. 5 of 52, ACGME Core Requirement on Diversity and Inclusion) stating that “programs must focus on mission-driven, ongoing, systematic recruitment and retention of a diverse and inclusive workforce of…faculty members.” |
| **1** | If most of your responses are in the one range, it is of dire importance to begin building your case for diversifying the faculty to gain departmental and institutional buy-in. Consider assessing the institutional climate for D&I and identifying the major challenges and resistance to an initiative to diversify the department faculty. This starts with gathering the necessary data to begin the conversation, establishing a sense of urgency (think compliance with both the LCME accreditation standards and the ACGME core requirements), and creating the vision to achieve a diverse faculty workforce within the department. This is the time to engage the department chair to consider establishing a task force within the department to begin addressing the need. Encourage the department chair to create a charge for the task force to catalyze the efforts and provide direction. |

*Developed by David Acosta, MD, April 21, 2019.*
