# Supplementary material for: Implementing a Holistic Review Toolkit for Faculty Recruitment and Retention
Source: MedEdPORTAL. 2024 Dec 4;20:11472. doi: 10.15766/mep_2374-8265.11472 (PMC11615027; doi:10.15766/mep_2374-8265.11472)
Supplement: Supplementary file 1 — Faculty Pilot Overview.docxOverview Equity-Minded Hiring_Step 1.docxAssess Readiness for Equity-Minded Hiring_Step 1.docxStaff Composition Inventory_Step 2.xlsxHolistic Search Committee Phases and Steps_Step 2.docxFaculty Workshop Facilitators Guide_Step 3.docxFaculty Workshop Presentation_Step 3.pptxFaculty Workshop Evaluation_Step 3.docxFaculty Workshop Activities_Step 3.docxJob Description Posting Tools and Resources_Step 4.docxInterview Questions Tools and Resources_Step 4.docxSubmission Requirements and Rating Tools_Step 4.docx360-Degree (Multisource) Reference Checking_Step 4.docxSearch Process Tools and Resources_Step 5.docxStanding Up a Search Committee_Step 5.docxMitigating Bias Resources_Step 5.docxOnboarding Tools and Resources_Step 6.docxCareer Development Discussion Guide_Step 6.docxU Colorado SOM Mentoring Resource Packet_Step 6.docxBaylor College of Medicine Exit Resources_Step 6.docxU Colorado SOM Equitable Hiring Tool_Step 7.docxHolistic Hiring and Retention Tracker_Step 8.docxEvaluation Materials Development Phase_Steps 4-6.docx [file mep_2374-8265.11472-s001.zip › E. Holistic Search Committee Phases and Steps_Step 2.docx]

# Appendix E: Holistic Search Committee Phases and Steps

Implementation Guidance: Before implementing the recommendations in this checklist, your institution should review federal and local laws to ensure they align with organizational policies and procedures.

This checklist can be used by the implementation team to identify any gaps in your current process. It can also be used by the search committee chair and coordinator after you have developed your holistic hiring process to guide the search committee in their work.

| **Phase** | **✔** | **Evaluation Criteria** |
| --- | --- | --- |
| Workforce and Position Assessment |  | Complete the Department Readiness for Holistic Review and Equity-Minded Hiring and Retention Practices to Diversify Faculty Assessment. |
|  |  | Take an inventory of the composition of your workforce using the Staff Composition Inventory or another tool of your choice for baseline comparison. |
|  |  | Assess the position: Is the position in alignment with organizational goals? Does it need to be updated, or should it be closed? |
| Selection and Training of Search Committee Members |  | Search committee chair is selected by the dean, CEO, or hiring authority. |
|  |  | Members are identified and appointed to the search committee. Working within your school’s parameters, ensure that you include representation from members of institutional mission-appropriate diversity groups and from the diversity and inclusion office. |
|  |  | Appropriate administrative support has been arranged for the search process. |
|  |  | Search committee has received the Holistic Principles for Faculty Recruitment Training in advance of the search process and defines the experiences, attributes, competencies, and metrics (EACMs) desired. |
|  |  | Search committee has received unconscious bias training in advance of the search process. |
| Develop a Search Plan and Search Tools |  | Outreach: The relevant job boards, journals, publications, and websites for this position have been identified. |
|  |  | Job description is based on the EACMs. |
|  |  | Job posting uses language that is inclusive and reflective of the school’s mission and diversity goals. |
|  |  | Application includes a short-answer essay prompt for applicant’s EACMs. |
|  |  | Interview methods have been selected (e.g., behavioral-based, situational, core values, mission-based, and/or traditional). |
|  |  | Candidate evaluation form and score sheet have been developed. |
|  |  | Interview questions informed by the identified EACMs, and legal FAQs have been written. |
| Post Position |  | Position has been posted and broadly advertised to expand applicant pool. |
|  |  | Search announcement has been sent to identified individuals. |
|  |  | Nominations have been requested (viz., passive candidates). |
| Review Applications |  | Candidate screening tool used to review applications is based upon identified EACMs and job description. |
| Develop Short List |  | Identify 3-5 candidates that represent the EACMs. |
|  |  | Invite candidates who reflect position EACMs to interview. |
|  |  | Thank candidates who were not selected for their interest in the position. |
|  |  | It is recommended (Johnson, Hekman, and Chan, 2016) that you have two or more candidates from the school’s mission-appropriate diversity groups (or provide an explanation why not). |
| Schedule On-campus (or Virtual) Interview |  | Design interview questions and candidate evaluation rubrics based on the position’s EACMs. |
|  |  | Assign interview questions to each search committee member. |
|  |  | Conduct virtual or in person interviews and write evaluations using an objective rubric to evaluate candidate responses to interview questions. |
|  |  | Interview visit may also include meetings with identified stakeholders that may not participate in candidate evaluations (e.g., Office of Faculty Development, Office of Diversity, Inclusion, and Equity). |
|  |  | Request anonymous feedback on the recruitment process from all candidates at the end of the day. |
| Second-Round Interviews and Finalist Selection |  | Conduct second-round interviews based on the position’s EACMs and write evaluations using an objective rubric to evaluate candidate responses as needed for the position. |
|  |  | Make reference calls using standard behavioral questions that reflect your desired EACMs. |
|  |  | Create and present to the dean, CEO, or hiring authority a list of finalists including EACM candidate evaluation forms completed by search committee/interviewers. |
| Negotiation of Employment and Close Search |  | Develop a mutual understanding of the position with the leading candidate and prepare an offer. |
|  |  | Communicate with unsuccessful candidates once the leading candidate has accepted the offer. |
|  |  | Announce the search outcome to the community and thank the search committee publicly. |
|  |  | Plan onboarding activities. |

Reference: Johnson SK, Hekman DR, Chan ET. If there is only one woman in your candidate pool, there is statistically no chance she will be hired. Harvard Business Review. April 26, 2016. Accessed October 18, 2019. <https://hbr.org/2016/04/if-theres-only-one-woman-in-your-candidate-pool-theres-statistically-no-chance-shell-be-hired?referral=00060>
