# Supplementary material for: Implementing a Holistic Review Toolkit for Faculty Recruitment and Retention
Source: MedEdPORTAL. 2024 Dec 4;20:11472. doi: 10.15766/mep_2374-8265.11472 (PMC11615027; doi:10.15766/mep_2374-8265.11472)
Supplement: Supplementary file 1 — Faculty Pilot Overview.docxOverview Equity-Minded Hiring_Step 1.docxAssess Readiness for Equity-Minded Hiring_Step 1.docxStaff Composition Inventory_Step 2.xlsxHolistic Search Committee Phases and Steps_Step 2.docxFaculty Workshop Facilitators Guide_Step 3.docxFaculty Workshop Presentation_Step 3.pptxFaculty Workshop Evaluation_Step 3.docxFaculty Workshop Activities_Step 3.docxJob Description Posting Tools and Resources_Step 4.docxInterview Questions Tools and Resources_Step 4.docxSubmission Requirements and Rating Tools_Step 4.docx360-Degree (Multisource) Reference Checking_Step 4.docxSearch Process Tools and Resources_Step 5.docxStanding Up a Search Committee_Step 5.docxMitigating Bias Resources_Step 5.docxOnboarding Tools and Resources_Step 6.docxCareer Development Discussion Guide_Step 6.docxU Colorado SOM Mentoring Resource Packet_Step 6.docxBaylor College of Medicine Exit Resources_Step 6.docxU Colorado SOM Equitable Hiring Tool_Step 7.docxHolistic Hiring and Retention Tracker_Step 8.docxEvaluation Materials Development Phase_Steps 4-6.docx [file mep_2374-8265.11472-s001.zip › F. Faculty Workshop Facilitators Guide_Step 3.docx]

Appendix F:

Holistic Principles for Faculty Recruitment

and Retention Workshop

Facilitator’s Guide

Implementation Guidance: The following workshop specifications and agenda have been provided as examples and may be modified as necessary for your own needs.

Workshop Setup and Materials Needed

| **Duration** | ~3 hours per stakeholder group. The workshop can also be broken into two 1.5-hour segments. |
| --- | --- |
| **Materials** | 1 flip chart for the Parking Lot, Post-it notes and pens at each table for capturing Parking Lot items, name tents (as needed) |
| **Handouts** | - Activities 1-3 (1 worksheet per participant, or collaborative Google or SharePoint documents 1 per small breakout group) - Holistic Search Committee Phases and Steps document (1 copy per participant, or collaborative Google or SharePoint documents 1 per small breakout group) - **Optional:** Printout of the slides as note pages |
| **Recruiting Materials** | Have all participants **bring materials relevant to their recruiting efforts;** examples include but are not limited to:   - Job postings and announcements, - Interview questions, - Application screening rubrics, and - Interview scoring rubrics. |
| **Instructions for Setup** | Content review and preparation   - Gather your school/institution/unit/department data on race and ethnicity by rank to add to slide 12. - Gather your school/institution/unit/department faculty data on gender by rank to add to slide 15. - Review the facilitation notes and modify them for relevance to your institutional and departmental context. - Review slide 38 on implicit and structural bias. Make changes to or eliminate the slide per your local laws and organizational policies.   In-person room set   - Place 1 flip chart labeled Parking Lot near the front of the room and make sure it is visible to all participants. - Tables should be in rounds of ~6-8 with all participants able to see the presentation.   **Note:** If the room is large and/or the group is over 30, make sure that you have mics available for the discussion and sharing parts of the workshop.  Table set   - Set of Post-it notepads—these will be used to write notes on for the Parking Lot, e.g., questions, statements, etc. - Pens - Name tents (as needed)   Virtual room set-up   - Determine if small groups will be assigned randomly or preset. - Have shared activity document links ready (1 per group). - Encourage participants to be on camera (especially for small-group work). |

Workshop Outline (3 hours or two 1.5-hour sessions)

| **Suggested Time** | **Topic** | **Slides & Materials** |
| --- | --- | --- |
| 5 minutes | Introduction   - Objectives - Any housekeeping notes | Slides 1-2 |
| 10 minutes | Holistic Review in Admissions—A System Perspective   - What holistic review is designed to achieve - Definition and core principles***** | Slides 3-11 |
| 10 minutes | The Current State of Faculty Diversity   - Present national statistics on faculty diversity - Compare these to your own school or department-level data | Slides 12-17 |
| 55 - 60 minutes | Holistic Principles in Faculty Recruitment and Retention   - Adaptations to the definition and the core principles - The holistic faculty selection model - Activity 1 and discussion | Slides 18-29  Activity 1 |
| 5 - 10 minutes | **Break or End Day 1** | Slide 30 |
| 60 minutes | Applying Holistic Principles to Faculty Recruitment and Retention   - What you can control and influence - Activity 2 and discussion | Slides 31-38  Activity 2 |
| 30 minutes | Best Practices for Applying Holistic Principles to Faculty Selection   - Training and interviewing - Impact/effort matrix - Action planning - Next steps and workshop close | Slides 39-45  Activity 3 |

***Note:** The Holistic Review Framework has been updated since the implementation of this pilot to conform with the 2024 Students for Fair Admissions v Harvard and UNC Supreme Court ruling on the use of race and ethnicity in college admissions. The PowerPoint deck includes a note about this change. The most recent version of the framework can be found on the [Holistic Review Website](https://www.aamc.org/services/member-capacity-building/holistic-review).
