# Supplementary material for: Implementing a Holistic Review Toolkit for Faculty Recruitment and Retention
Source: MedEdPORTAL. 2024 Dec 4;20:11472. doi: 10.15766/mep_2374-8265.11472 (PMC11615027; doi:10.15766/mep_2374-8265.11472)
Supplement: Supplementary file 1 — Faculty Pilot Overview.docxOverview Equity-Minded Hiring_Step 1.docxAssess Readiness for Equity-Minded Hiring_Step 1.docxStaff Composition Inventory_Step 2.xlsxHolistic Search Committee Phases and Steps_Step 2.docxFaculty Workshop Facilitators Guide_Step 3.docxFaculty Workshop Presentation_Step 3.pptxFaculty Workshop Evaluation_Step 3.docxFaculty Workshop Activities_Step 3.docxJob Description Posting Tools and Resources_Step 4.docxInterview Questions Tools and Resources_Step 4.docxSubmission Requirements and Rating Tools_Step 4.docx360-Degree (Multisource) Reference Checking_Step 4.docxSearch Process Tools and Resources_Step 5.docxStanding Up a Search Committee_Step 5.docxMitigating Bias Resources_Step 5.docxOnboarding Tools and Resources_Step 6.docxCareer Development Discussion Guide_Step 6.docxU Colorado SOM Mentoring Resource Packet_Step 6.docxBaylor College of Medicine Exit Resources_Step 6.docxU Colorado SOM Equitable Hiring Tool_Step 7.docxHolistic Hiring and Retention Tracker_Step 8.docxEvaluation Materials Development Phase_Steps 4-6.docx [file mep_2374-8265.11472-s001.zip › G. Faculty Workshop Presentation_Step 3.pptx]

## Slide 1
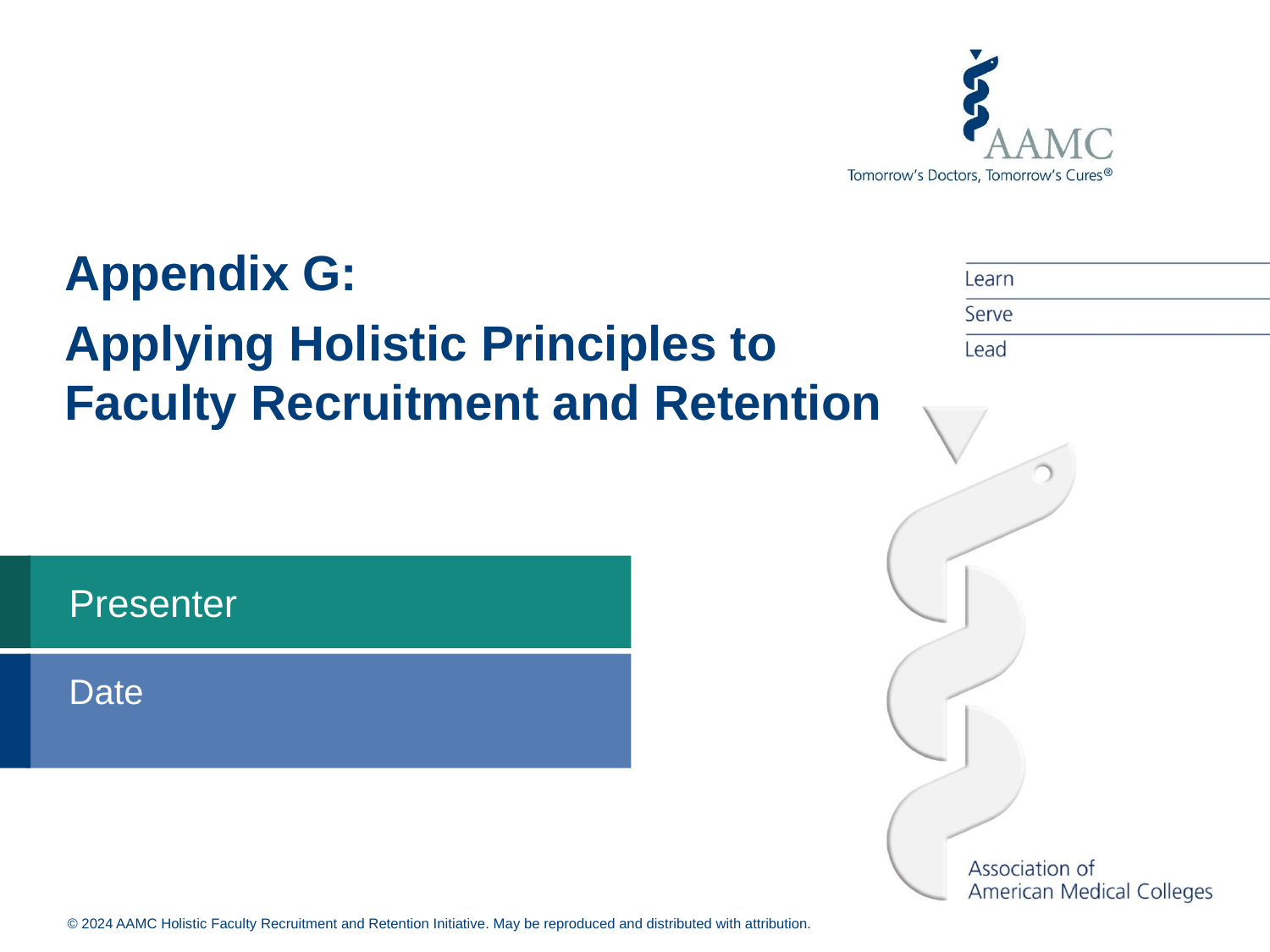

Appendix G:
Applying Holistic Principles to Faculty Recruitment and Retention
Presenter
Date

## Slide 2
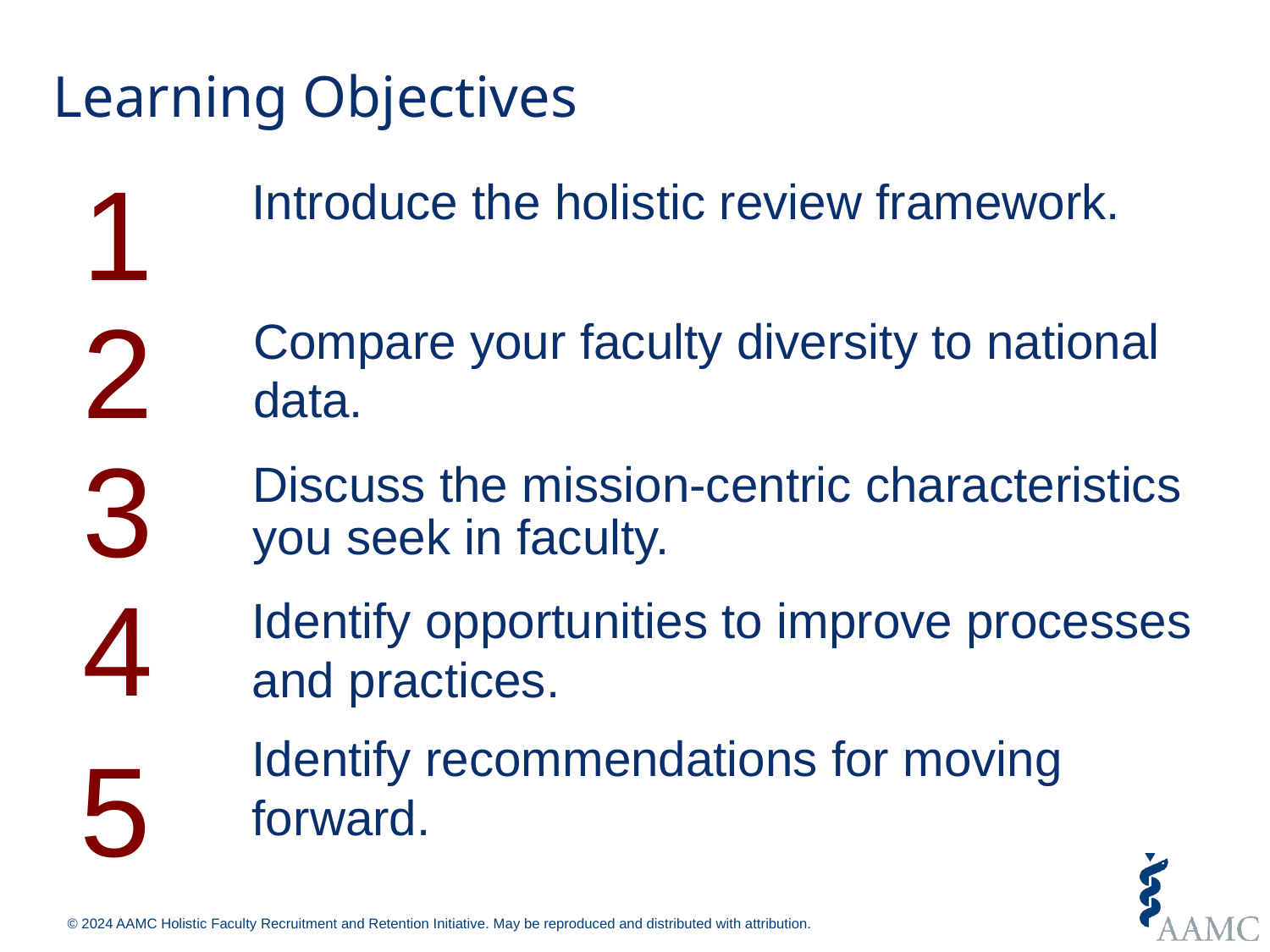

Learning Objectives
1
Introduce the holistic review framework.
2
Compare your faculty diversity to national data.
3
Discuss the mission-centric characteristics you seek in faculty.
4
Identify opportunities to improve processes and practices.
Identify recommendations for moving forward.
5

## Slide 3
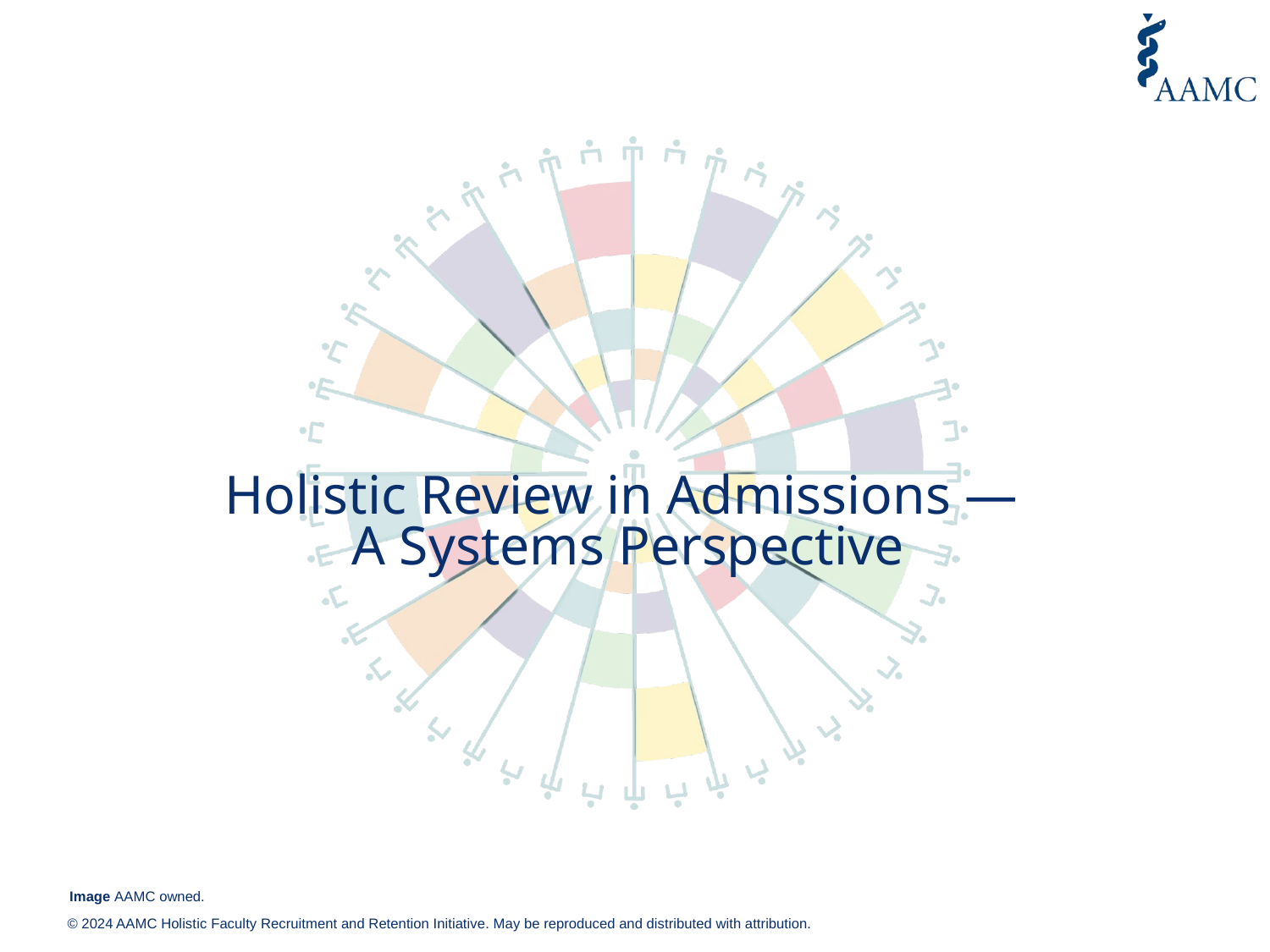

# Holistic Review in Admissions — A Systems Perspective
Image AAMC owned.

## Slide 4
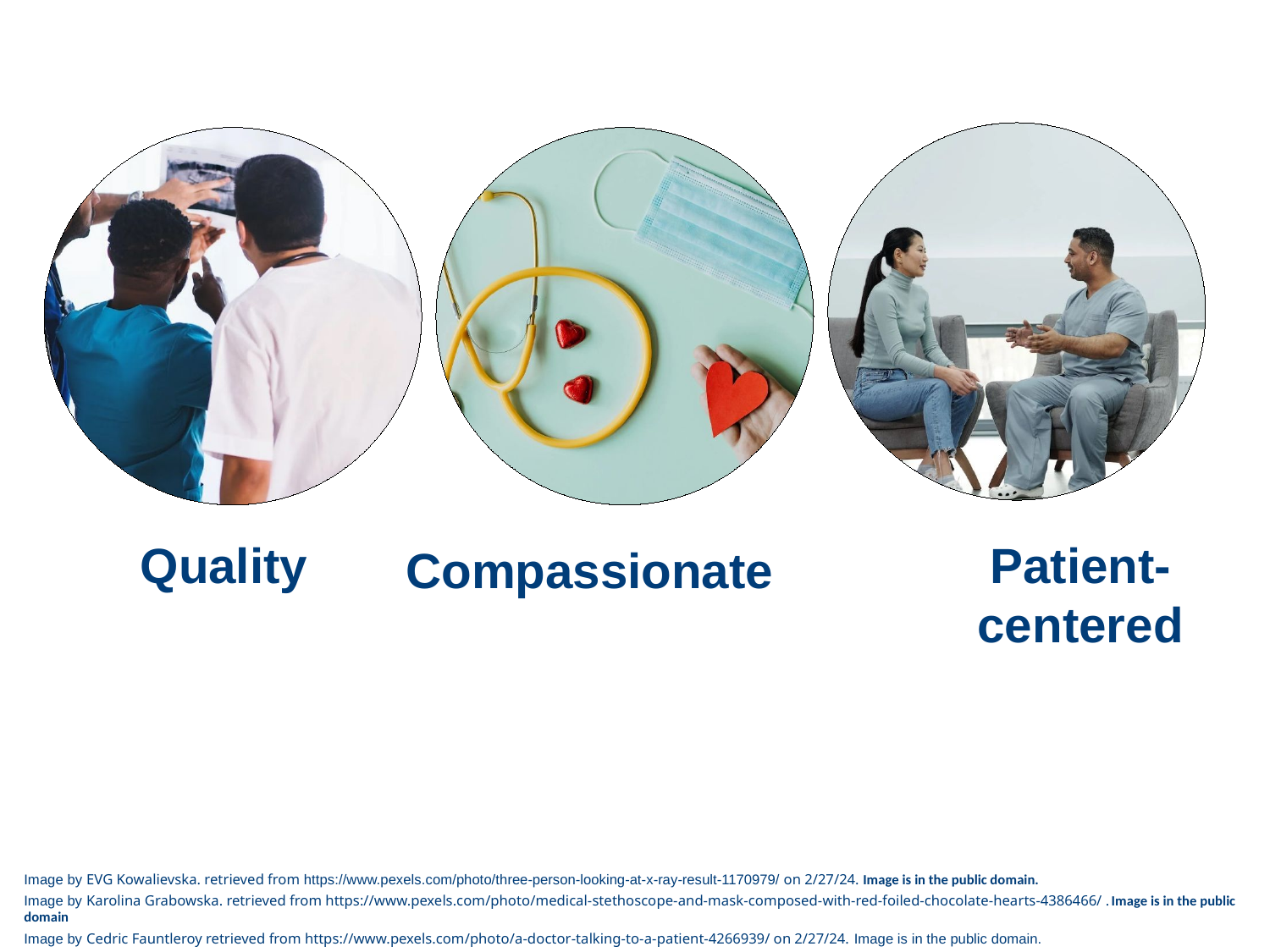

Quality
Patient-centered
Compassionate
Image by EVG Kowalievska. retrieved from https://www.pexels.com/photo/three-person-looking-at-x-ray-result-1170979/ on 2/27/24. Image is in the public domain.
Image by Karolina Grabowska. retrieved from https://www.pexels.com/photo/medical-stethoscope-and-mask-composed-with-red-foiled-chocolate-hearts-4386466/ .Image is in the public domain
Image by Cedric Fauntleroy retrieved from https://www.pexels.com/photo/a-doctor-talking-to-a-patient-4266939/ on 2/27/24. Image is in the public domain.

## Slide 5
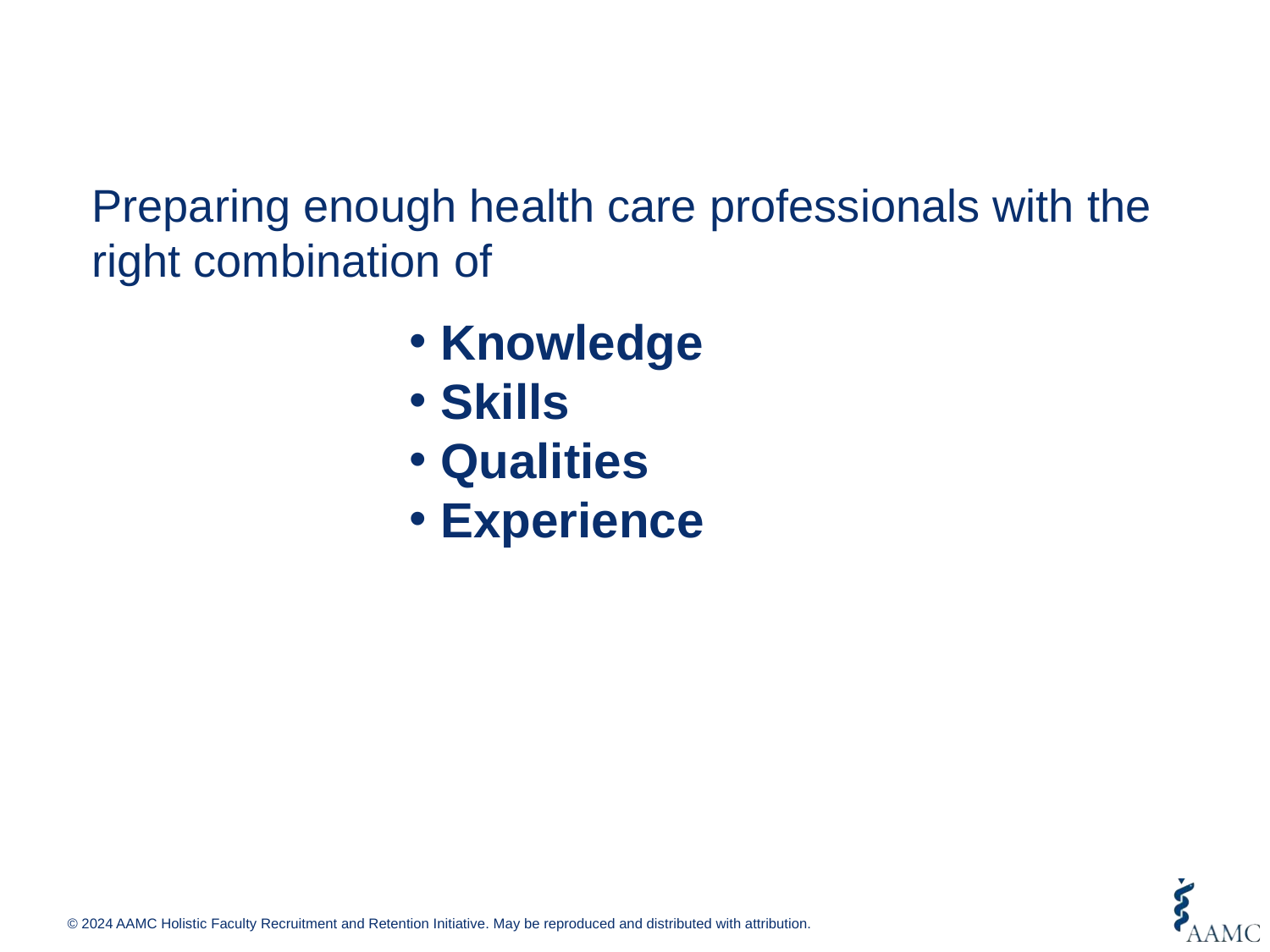

Preparing enough health care professionals with the right combination of
Knowledge
Skills
Qualities
Experience

## Slide 6
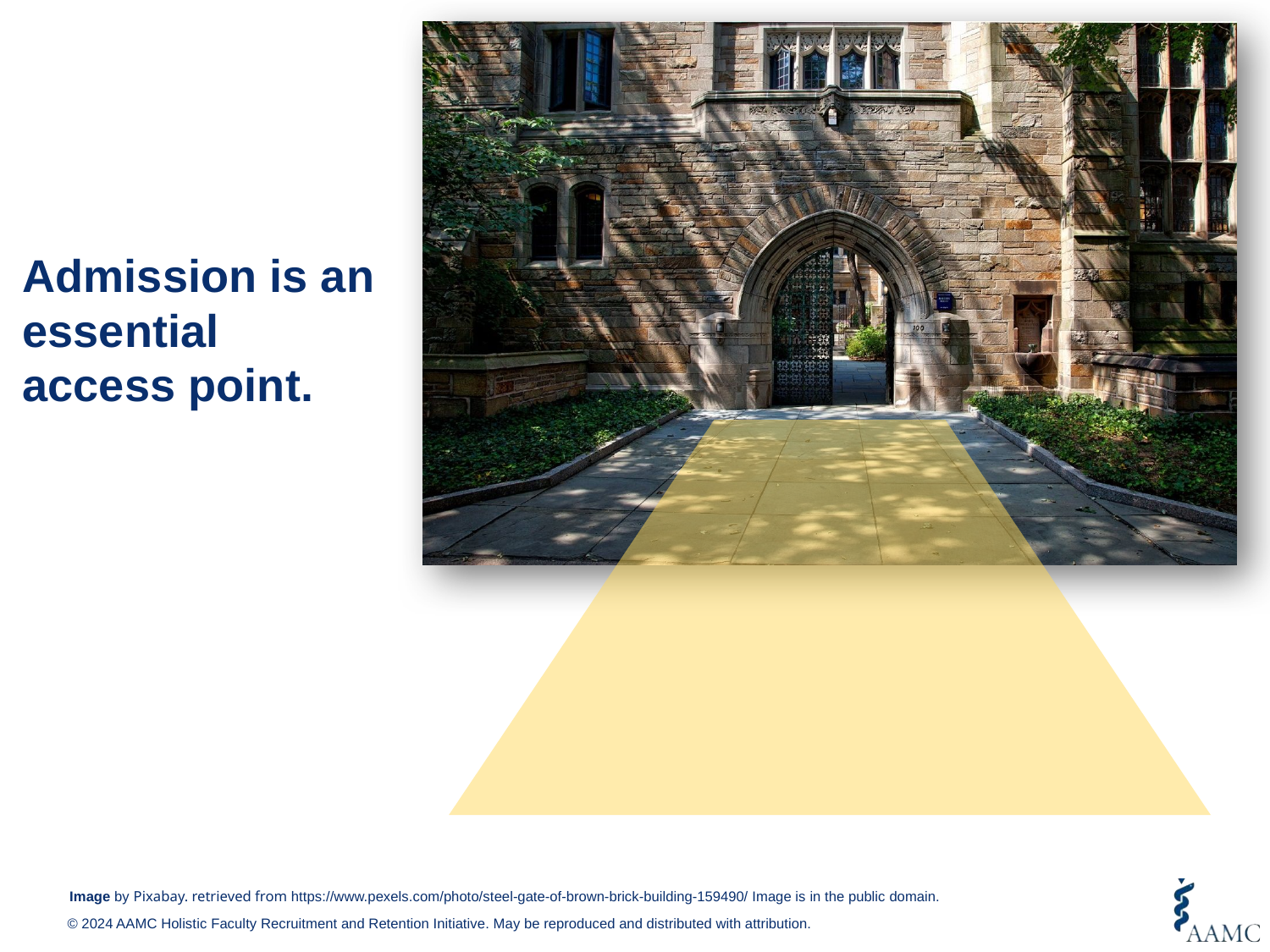

Admission is an essential access point.
Image by Pixabay. retrieved from https://www.pexels.com/photo/steel-gate-of-brown-brick-building-159490/ Image is in the public domain.

## Slide 7
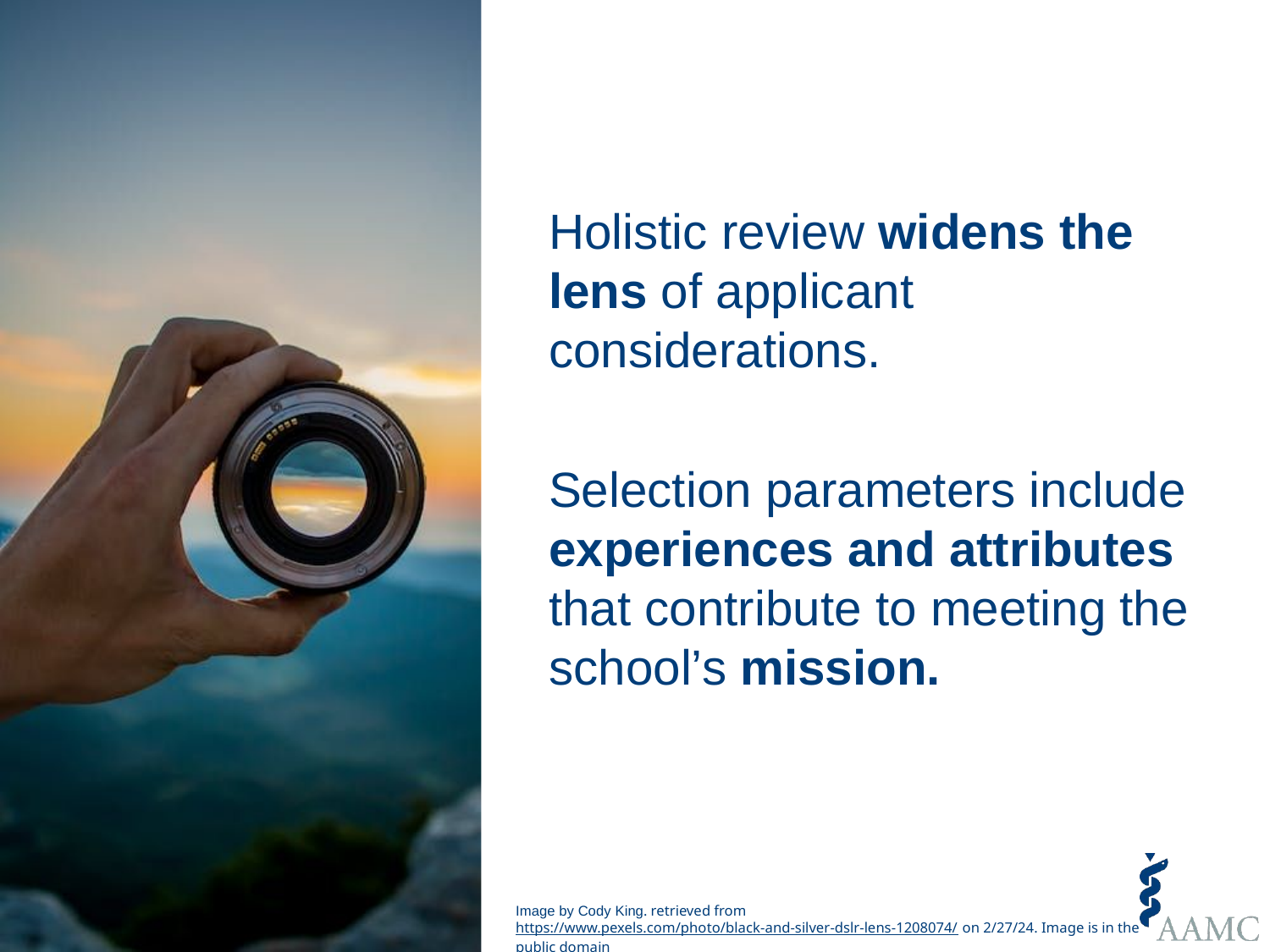

Holistic review widens the lens of applicant considerations.
Selection parameters include experiences and attributes that contribute to meeting the school’s mission.
Image by Cody King. retrieved from https://www.pexels.com/photo/black-and-silver-dslr-lens-1208074/ on 2/27/24. Image is in the public domain

## Slide 8
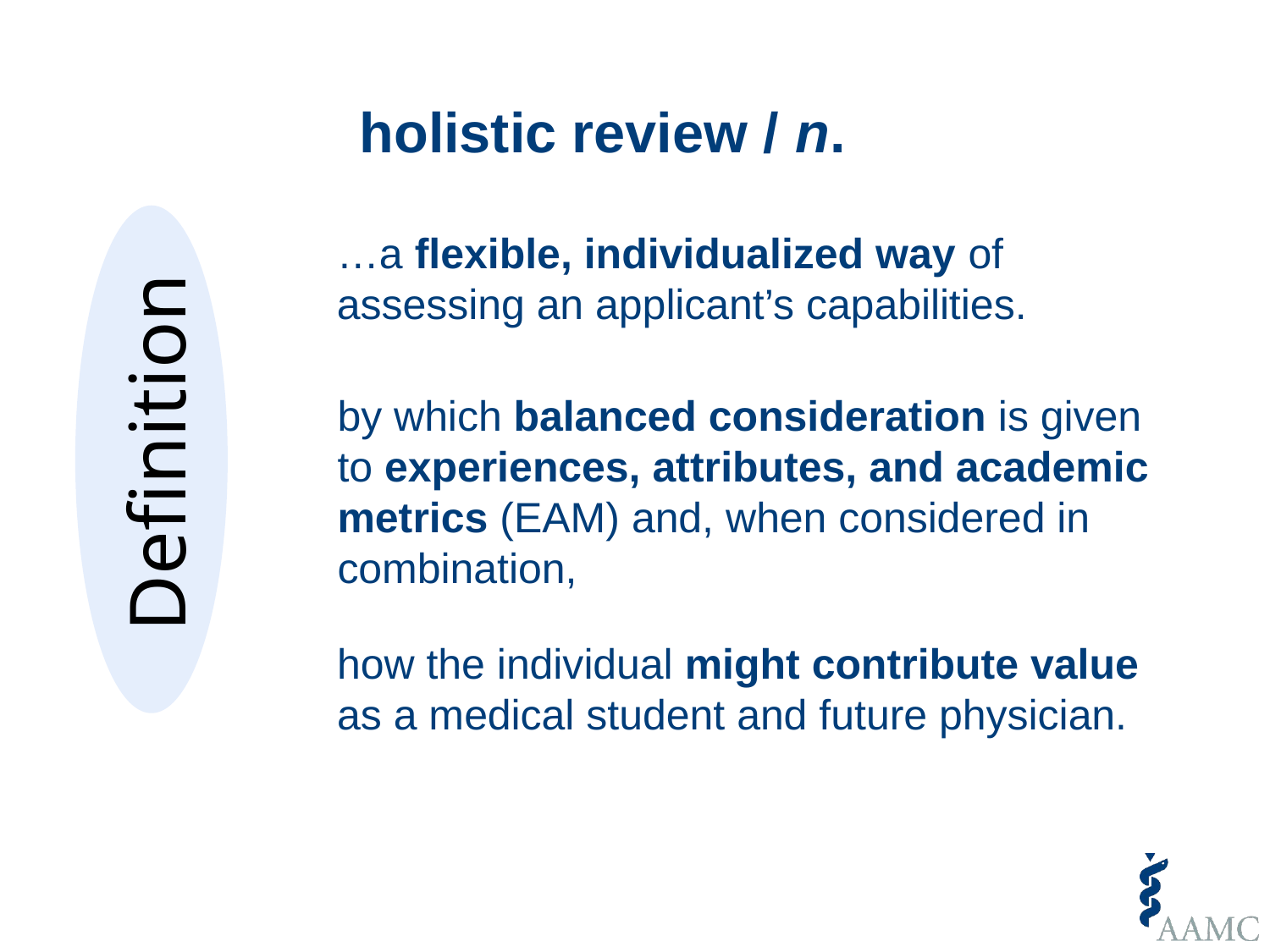

holistic review / n.
…a flexible, individualized way of assessing an applicant’s capabilities.
by which balanced consideration is given to experiences, attributes, and academic metrics (EAM) and, when considered in combination,
Definition
how the individual might contribute value as a medical student and future physician.

## Slide 9
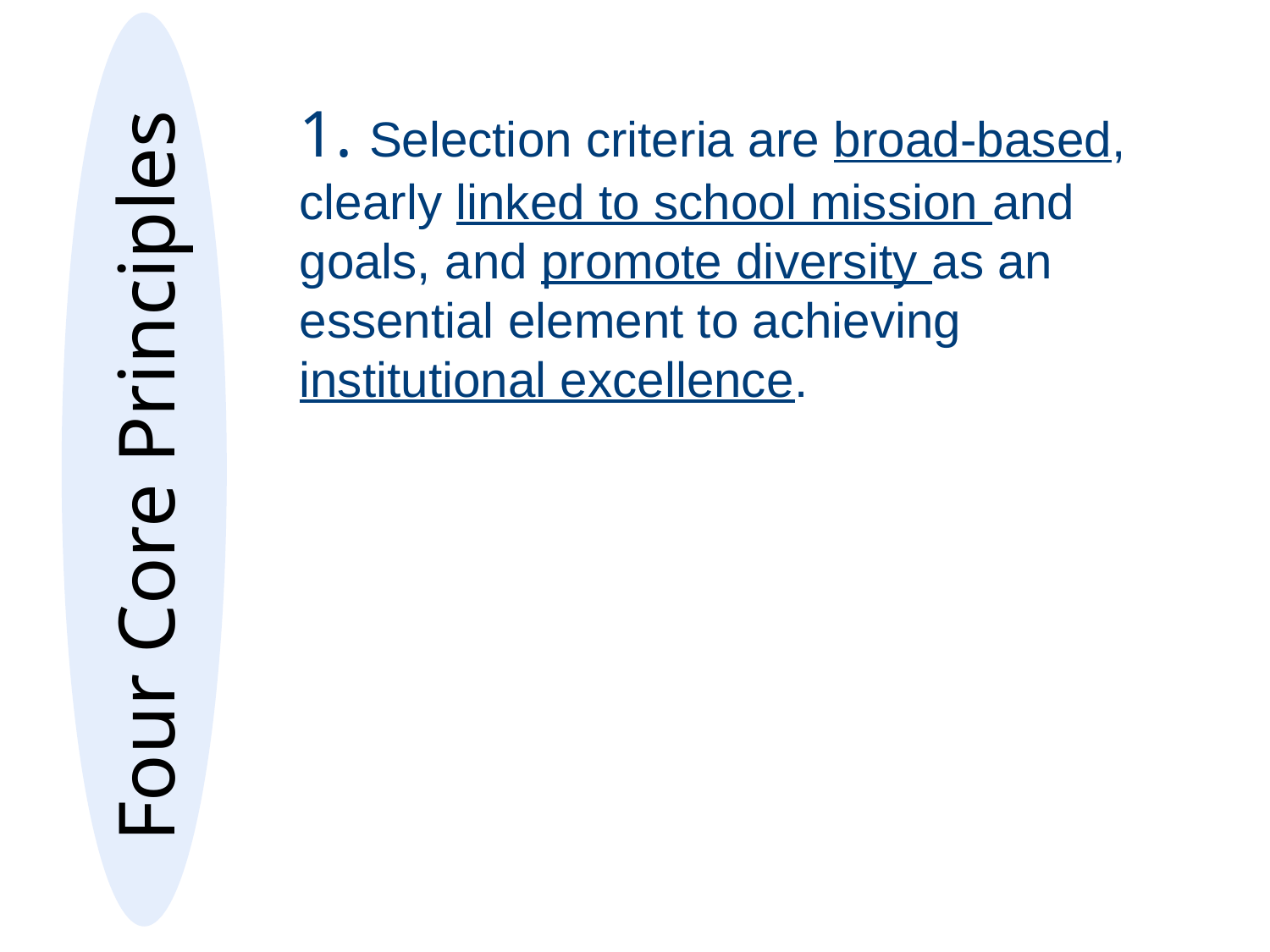

1. Selection criteria are broad-based, clearly linked to school mission and goals, and promote diversity as an essential element to achieving institutional excellence.
Four Core Principles

## Slide 10
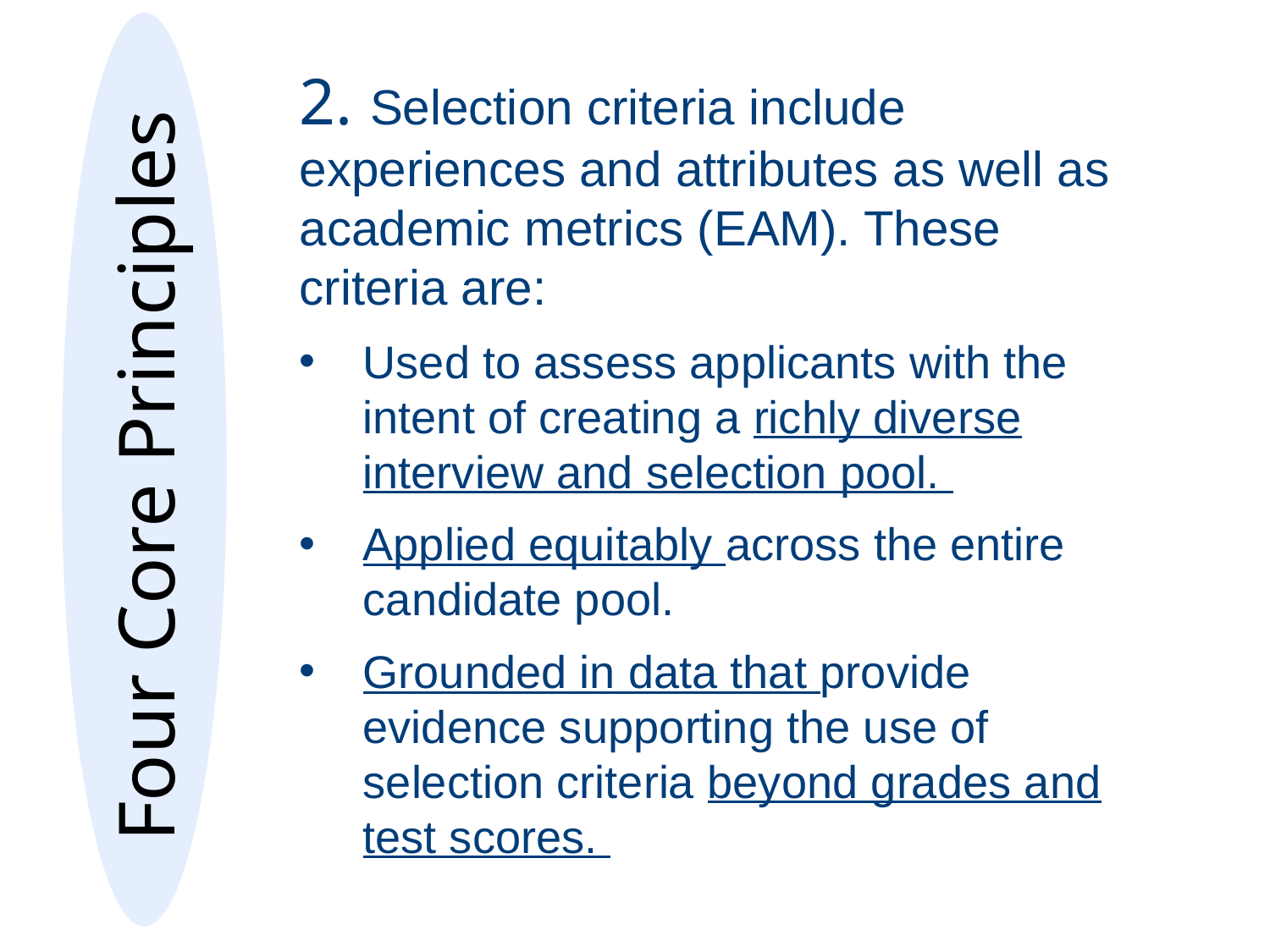

2. Selection criteria include experiences and attributes as well as academic metrics (EAM). These criteria are:
Used to assess applicants with the intent of creating a richly diverse interview and selection pool.
Applied equitably across the entire candidate pool.
Grounded in data that provide evidence supporting the use of selection criteria beyond grades and test scores.
Four Core Principles

## Slide 11
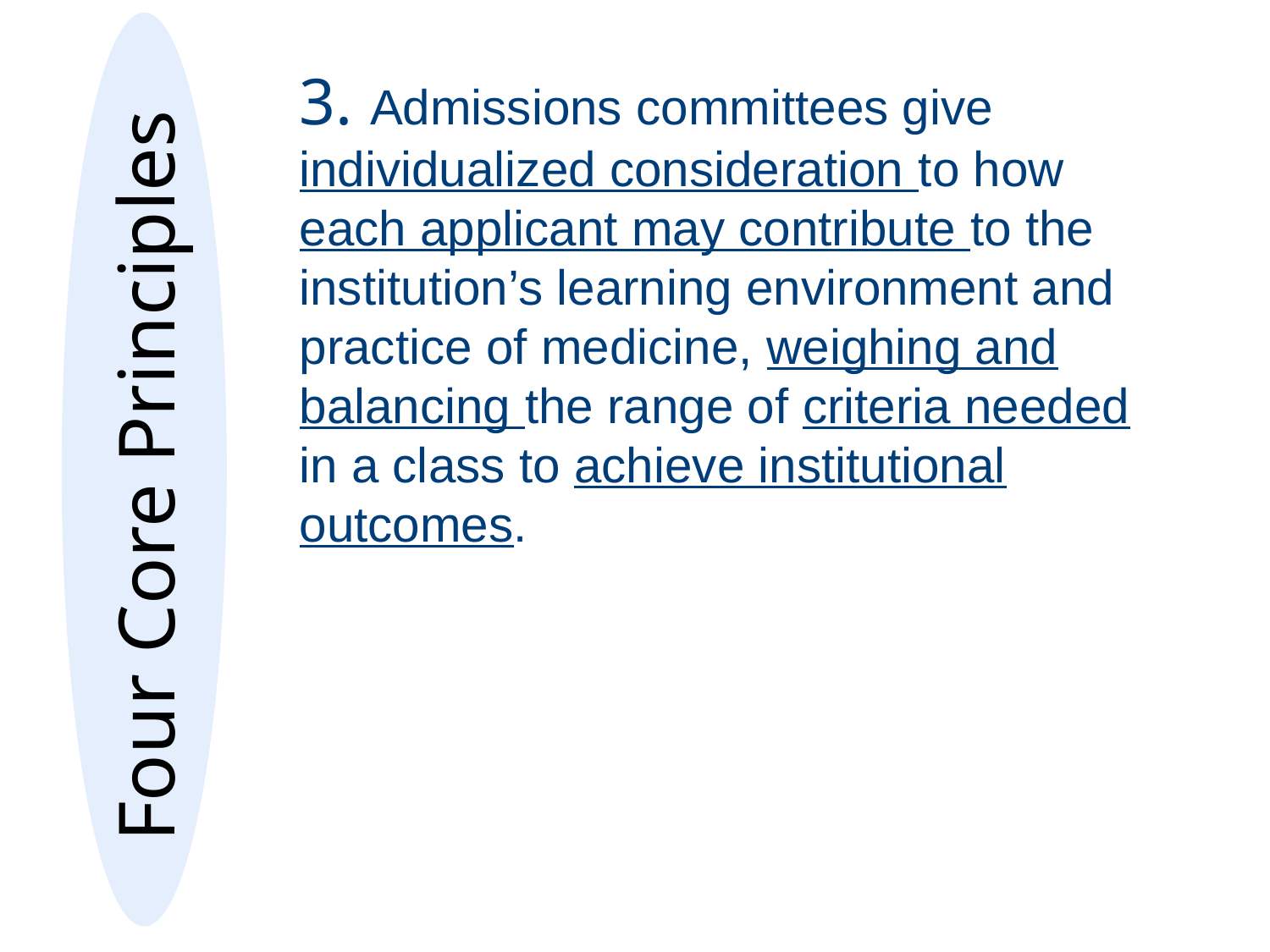

3. Admissions committees give individualized consideration to how each applicant may contribute to the institution’s learning environment and practice of medicine, weighing and balancing the range of criteria needed in a class to achieve institutional outcomes.
Four Core Principles

## Slide 12
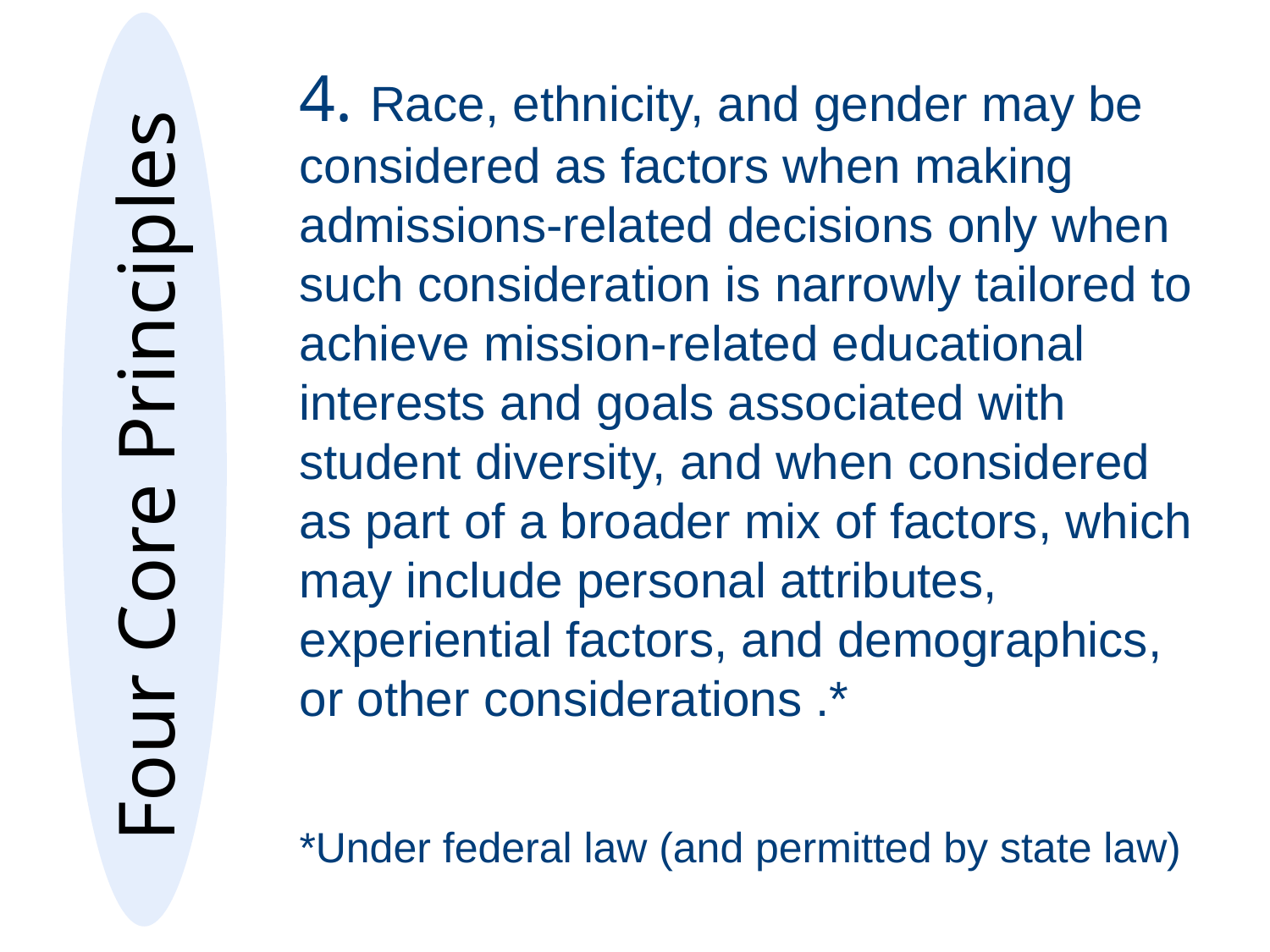

4. Race, ethnicity, and gender may be considered as factors when making admissions-related decisions only when such consideration is narrowly tailored to achieve mission-related educational interests and goals associated with student diversity, and when considered as part of a broader mix of factors, which may include personal attributes, experiential factors, and demographics, or other considerations .*
*Under federal law (and permitted by state law)
Four Core Principles

## Slide 13
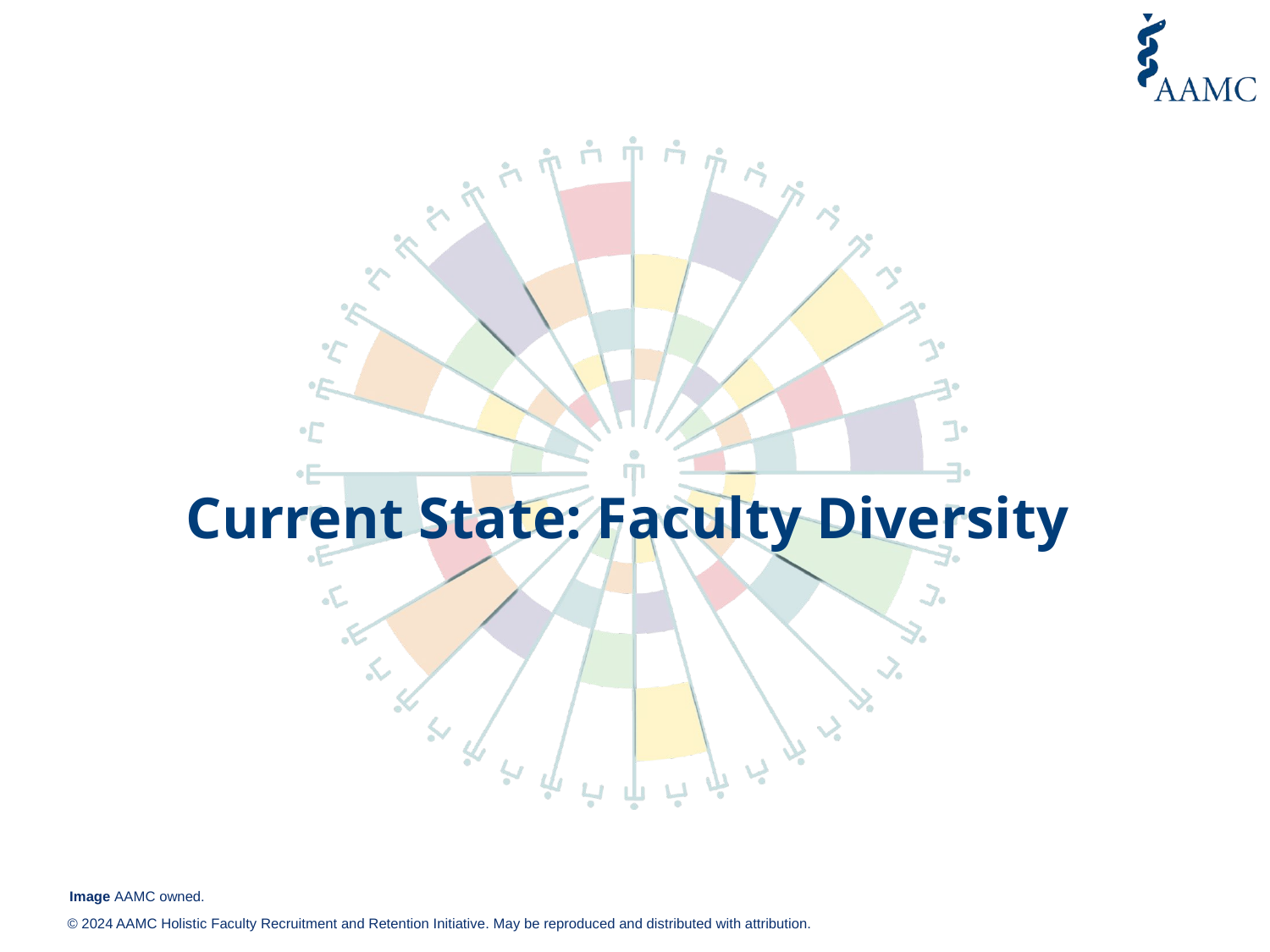

# Current State: Faculty Diversity
Image AAMC owned.

## Slide 14
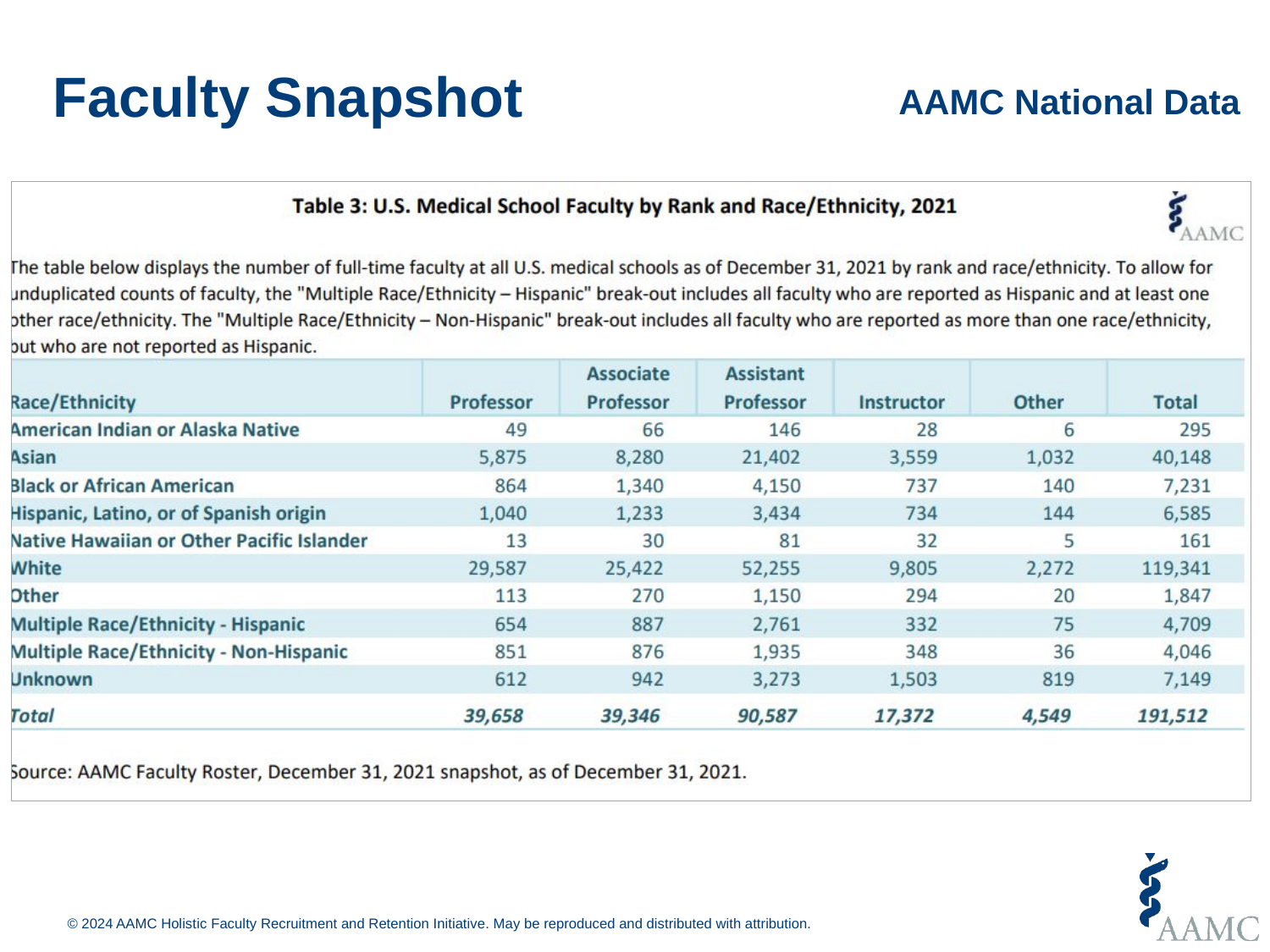

# Faculty Snapshot
AAMC National Data

## Slide 15
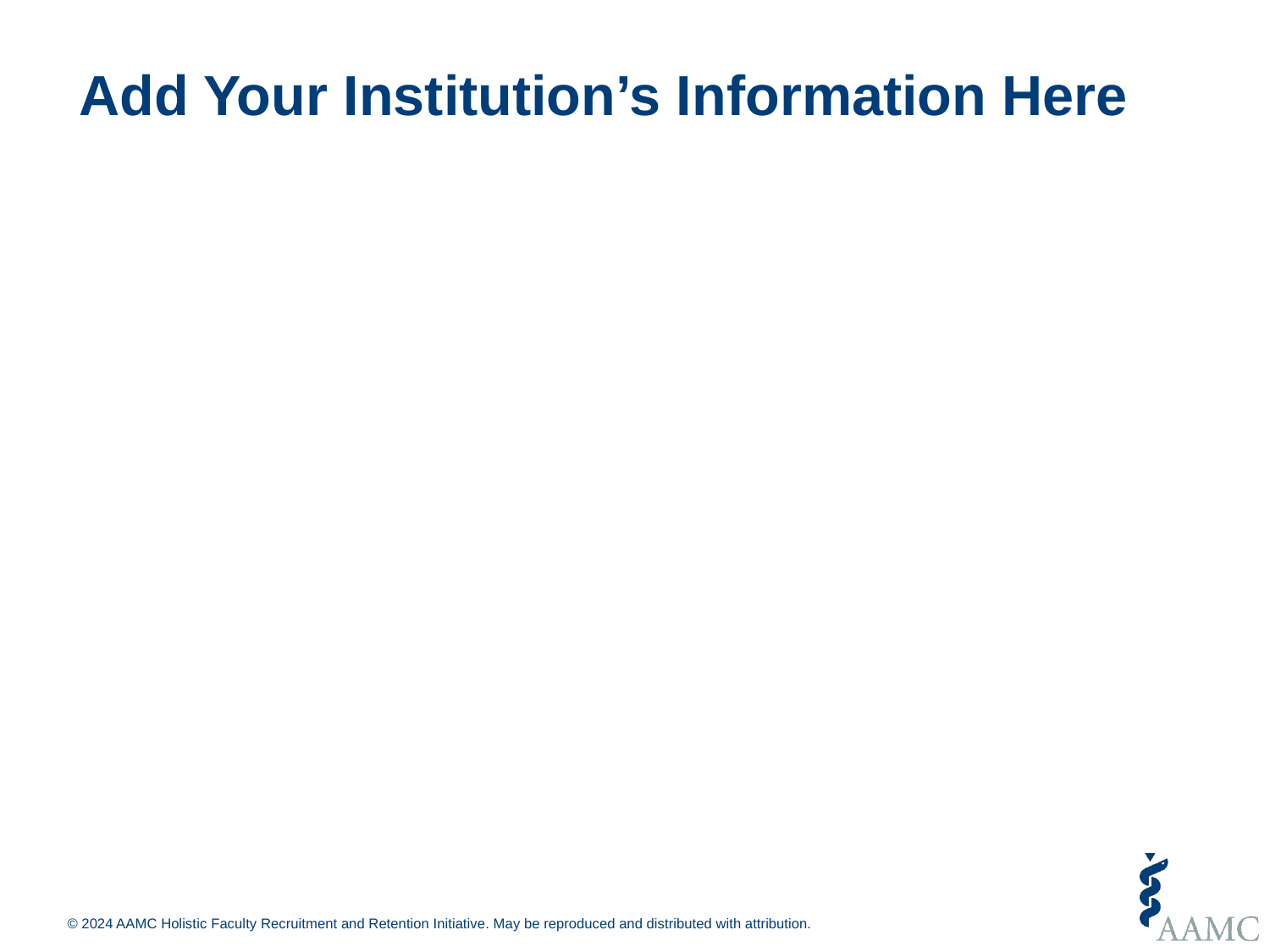

# Add Your Institution’s Information Here

## Slide 16
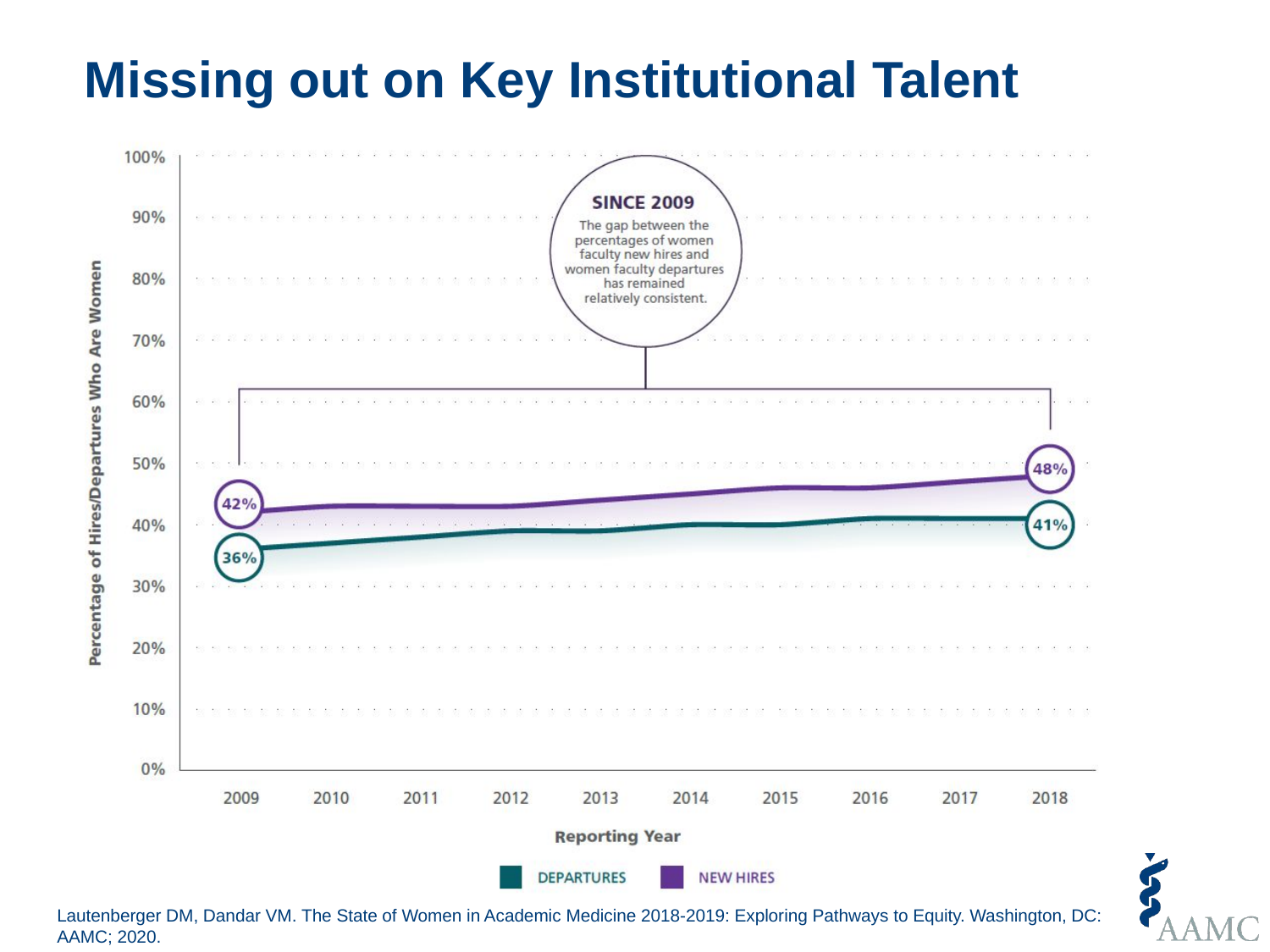

# Missing out on Key Institutional Talent
Lautenberger DM, Dandar VM. The State of Women in Academic Medicine 2018-2019: Exploring Pathways to Equity. Washington, DC: AAMC; 2020.

## Slide 17
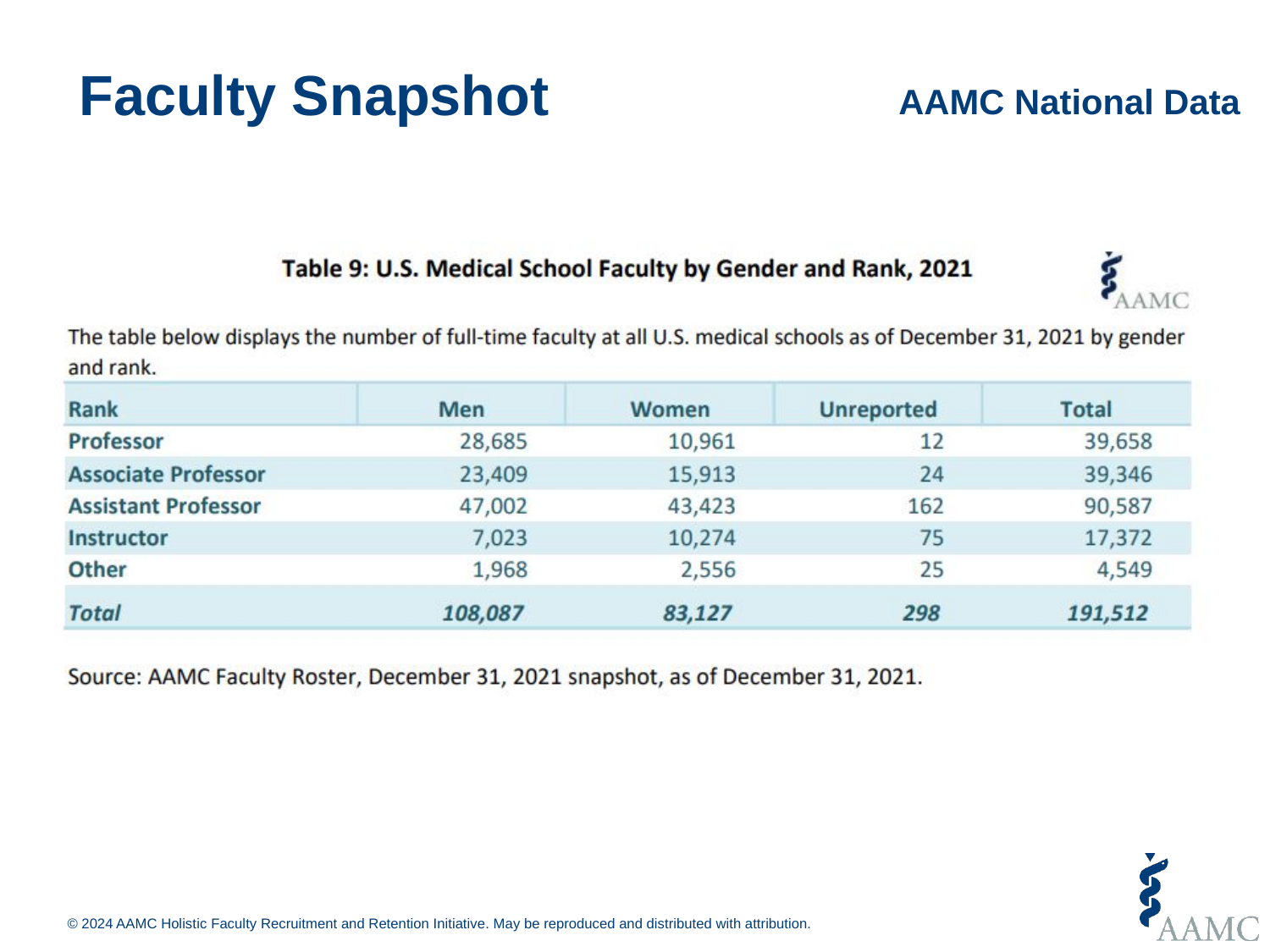

# Faculty Snapshot
AAMC National Data

## Slide 18
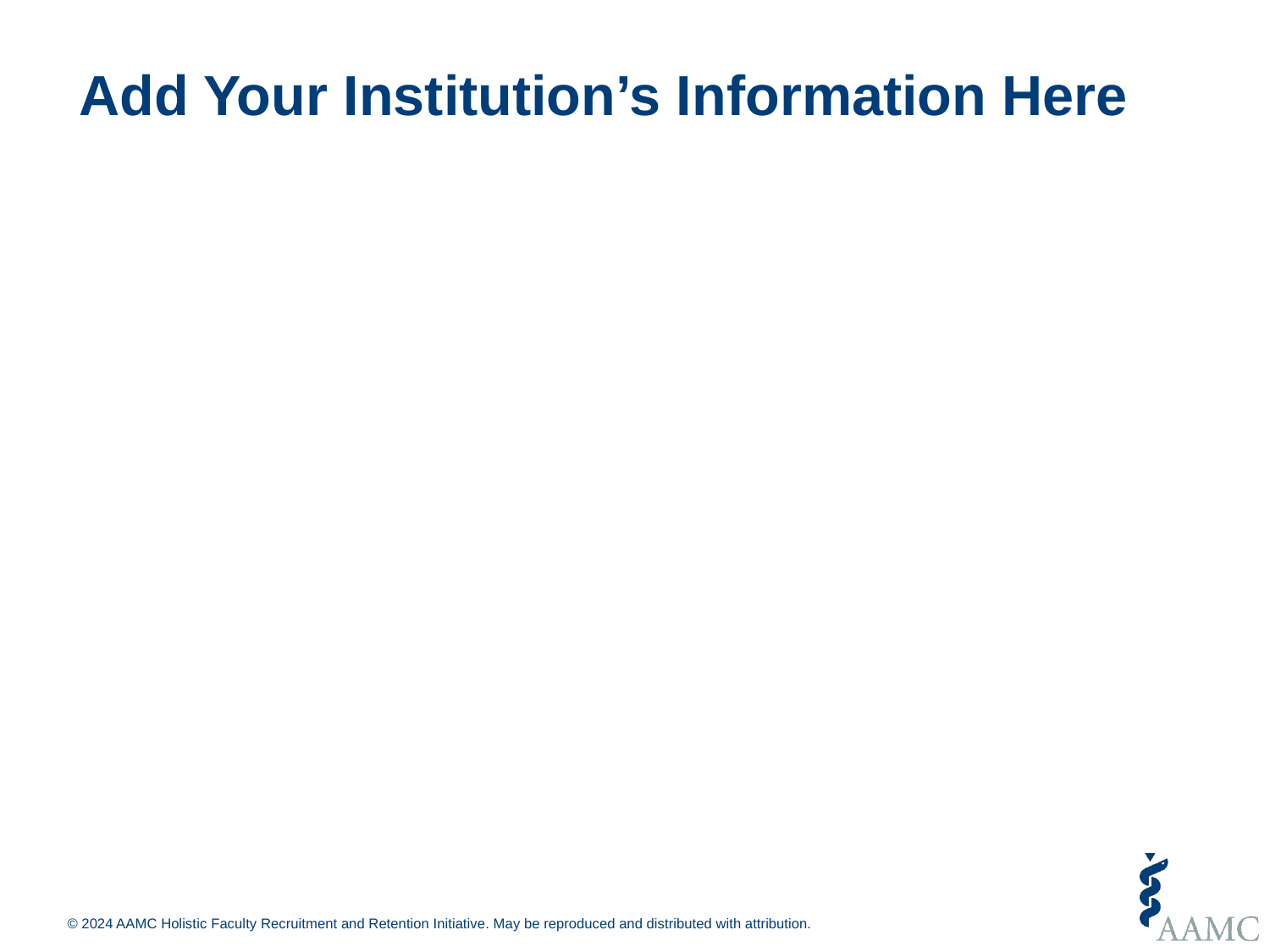

# Add Your Institution’s Information Here

## Slide 19
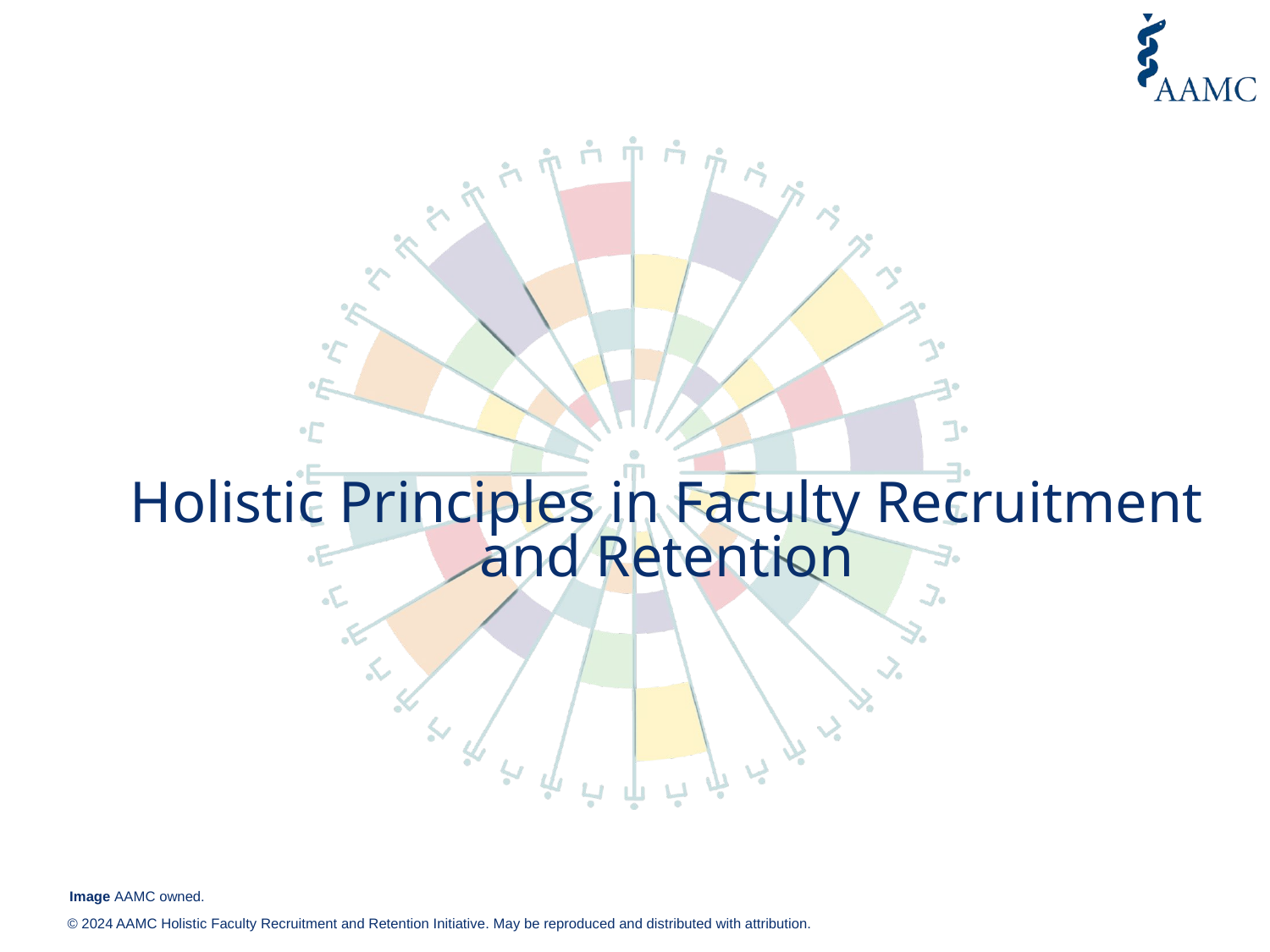

# Holistic Principles in Faculty Recruitment and Retention
Image AAMC owned.

## Slide 20
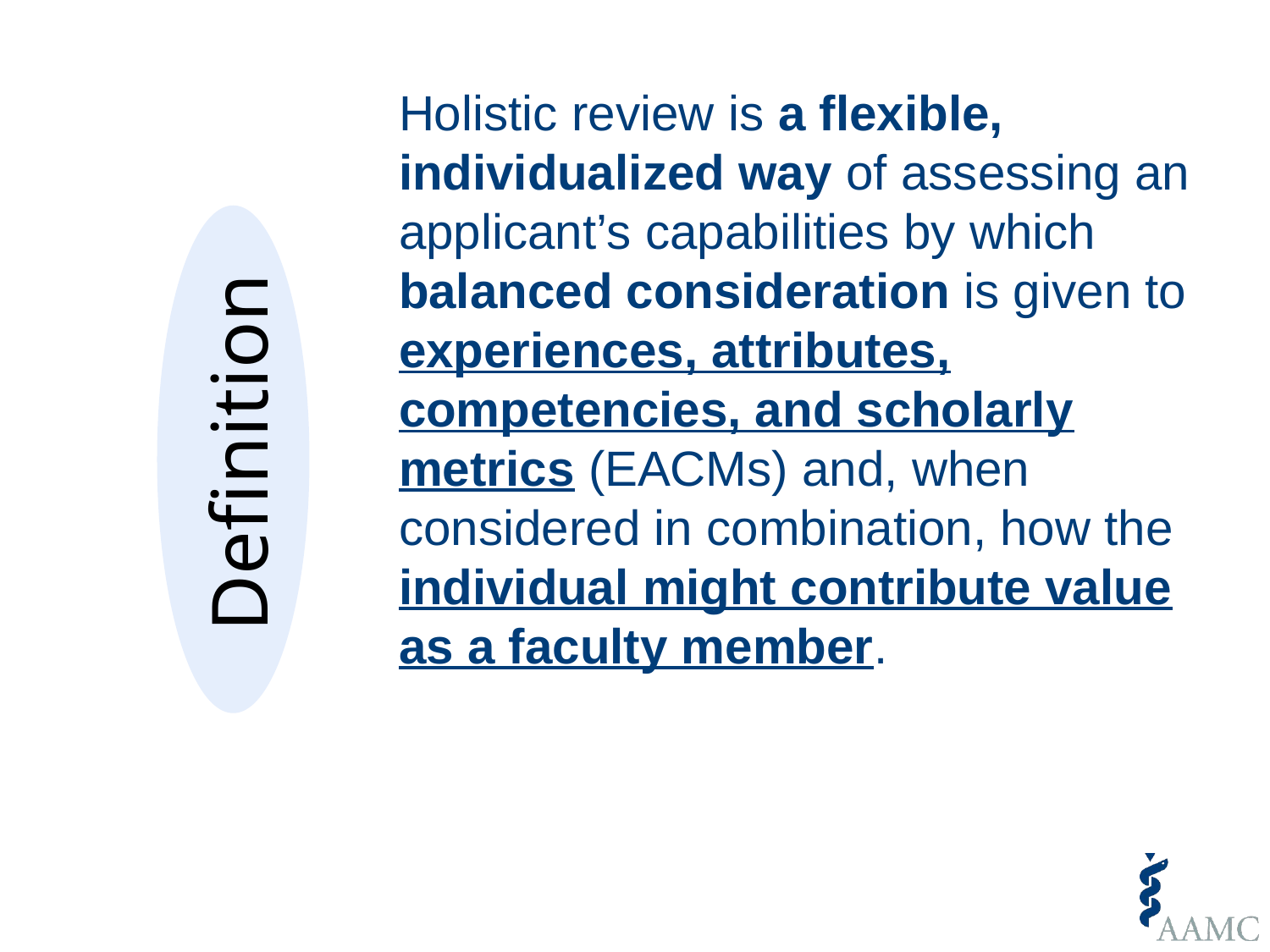

Holistic review is a flexible, individualized way of assessing an applicant’s capabilities by which balanced consideration is given to experiences, attributes, competencies, and scholarly metrics (EACMs) and, when considered in combination, how the individual might contribute value as a faculty member.
Definition

## Slide 21
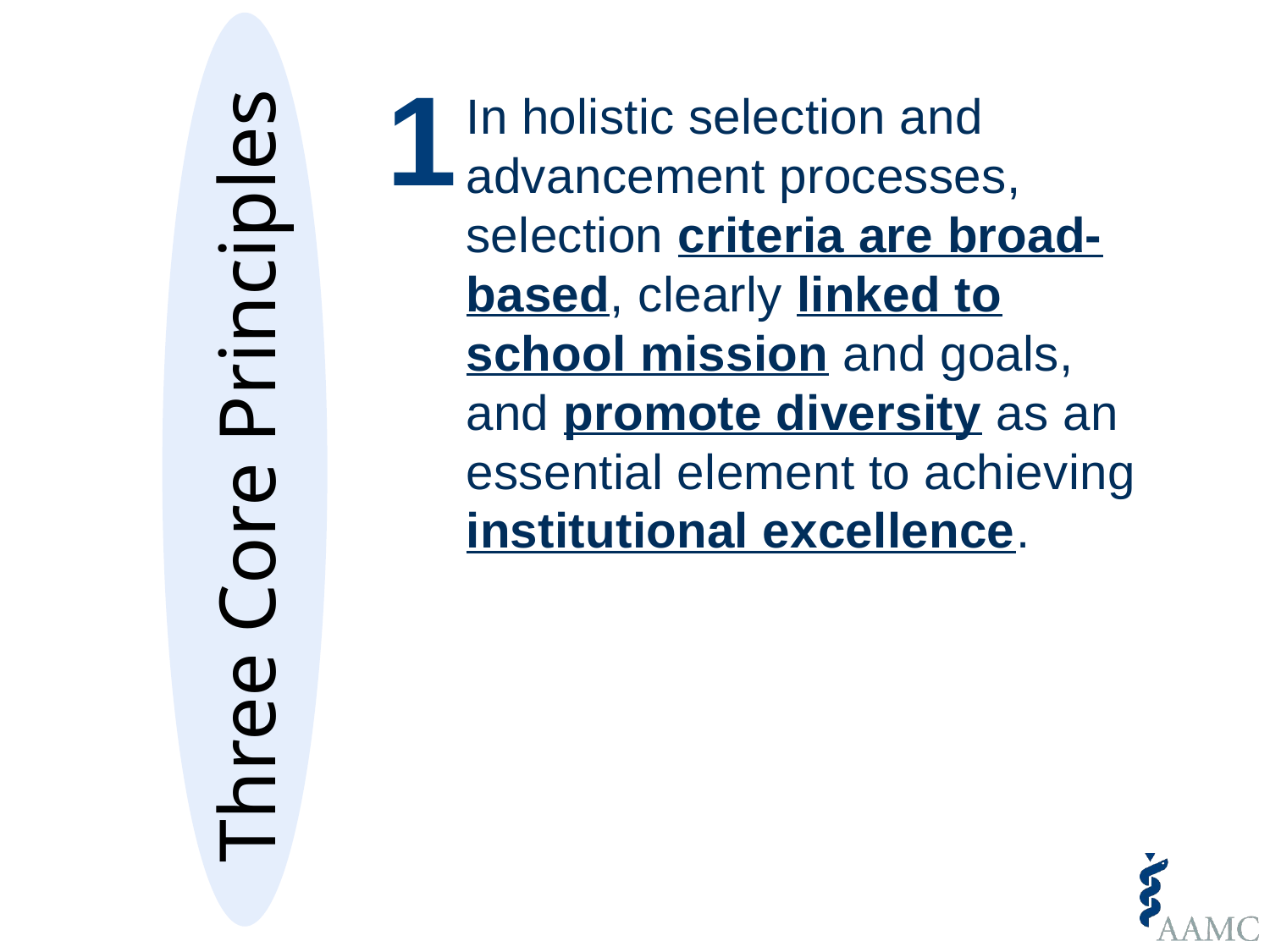

Three Core Principles
1
In holistic selection and advancement processes, selection criteria are broad-based, clearly linked to school mission and goals, and promote diversity as an essential element to achieving institutional excellence.

## Slide 22
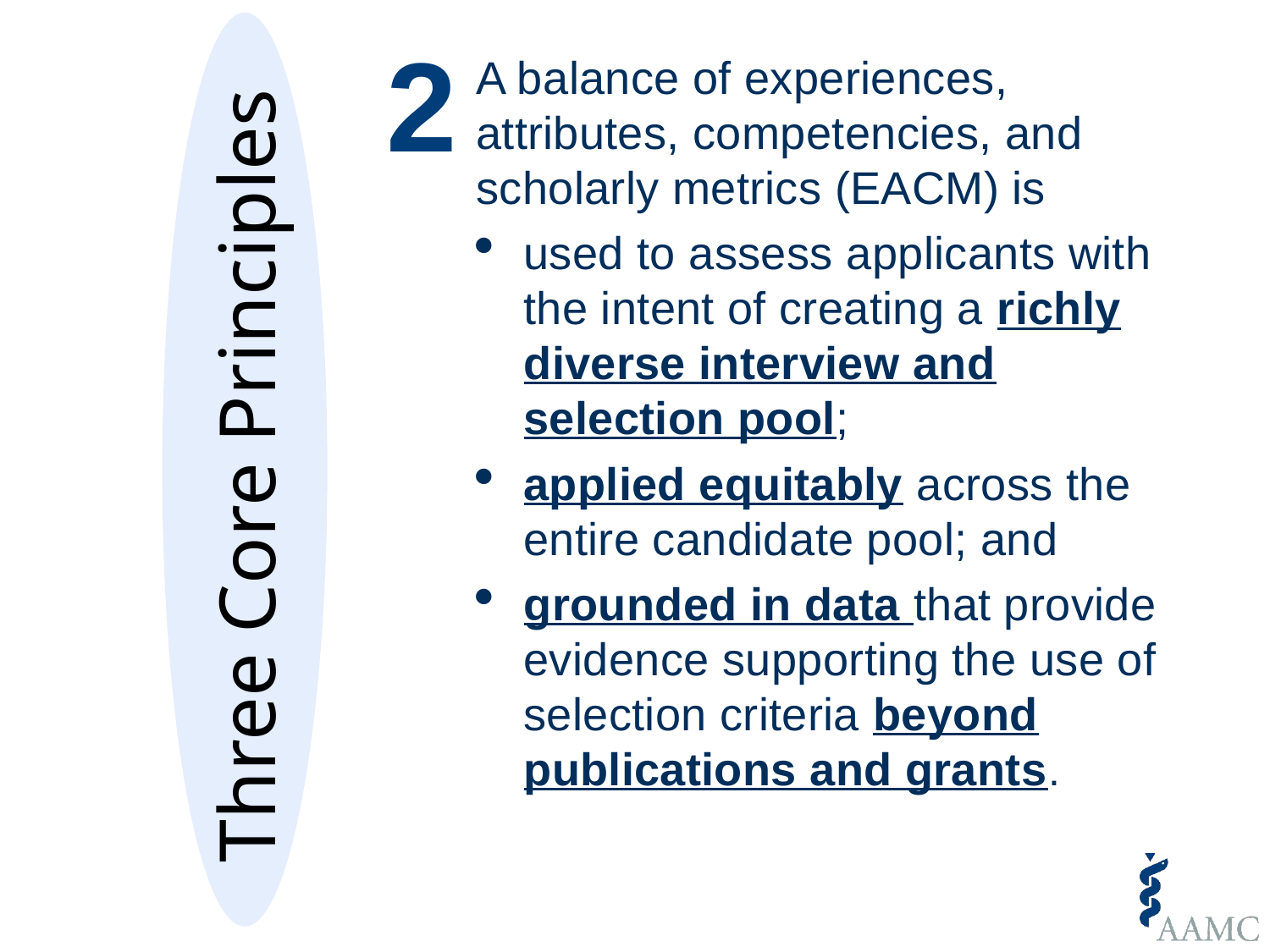

Three Core Principles
A balance of experiences, attributes, competencies, and scholarly metrics (EACM) is
used to assess applicants with the intent of creating a richly diverse interview and selection pool;
applied equitably across the entire candidate pool; and
grounded in data that provide evidence supporting the use of selection criteria beyond publications and grants.
2

## Slide 23
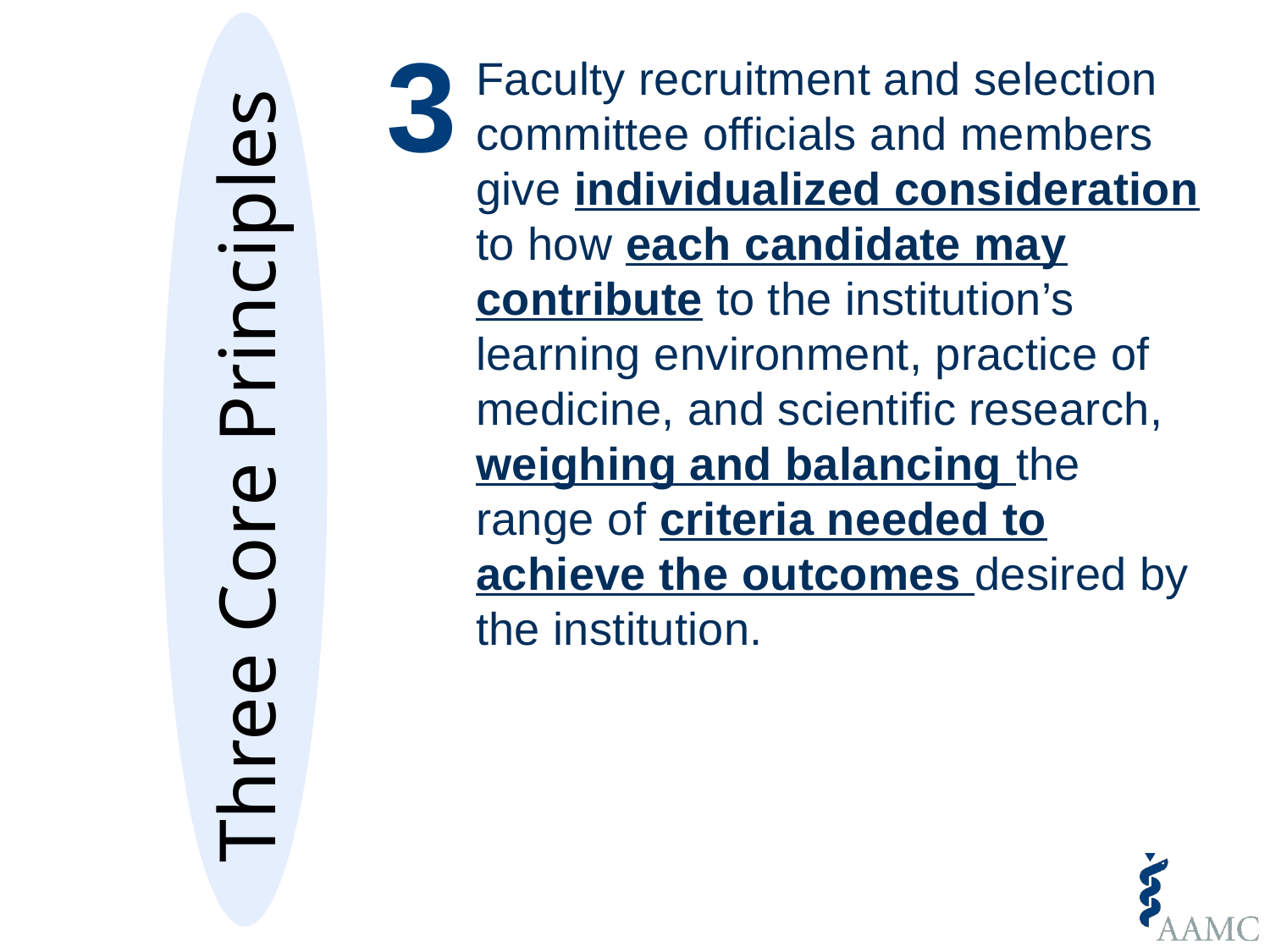

Three Core Principles
Faculty recruitment and selection committee officials and members give individualized consideration to how each candidate may contribute to the institution’s learning environment, practice of medicine, and scientific research, weighing and balancing the range of criteria needed to achieve the outcomes desired by the institution.  relevant relevant in this context. Maybe in a ‘cohort’? or maybe, ‘balancing the range of criteria desired in a faculty and/or staff body to achieve the outcomes desired by the institution.’
3

## Slide 24
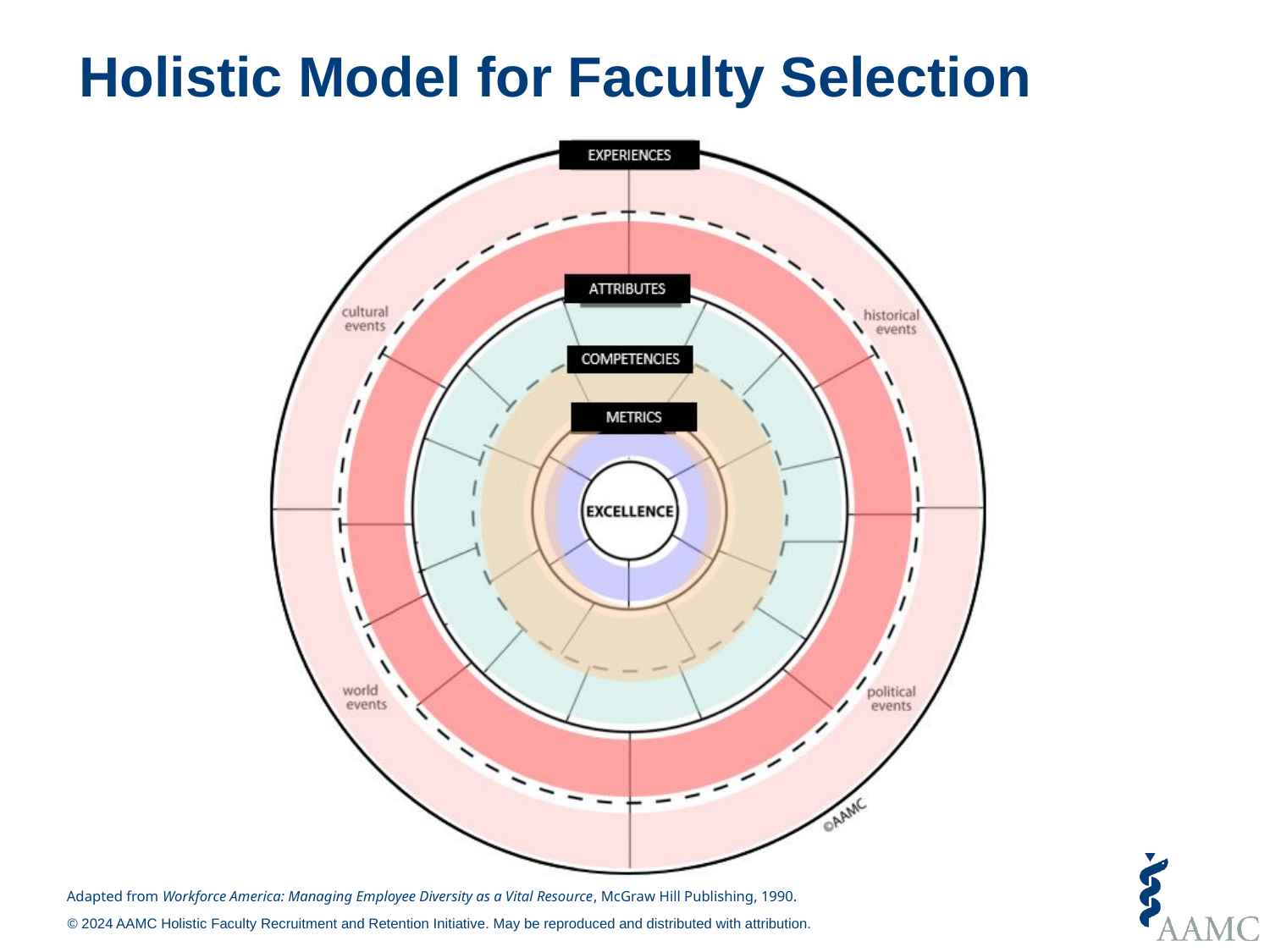

# Holistic Model for Faculty Selection
Adapted from Workforce America: Managing Employee Diversity as a Vital Resource, McGraw Hill Publishing, 1990.

## Slide 25
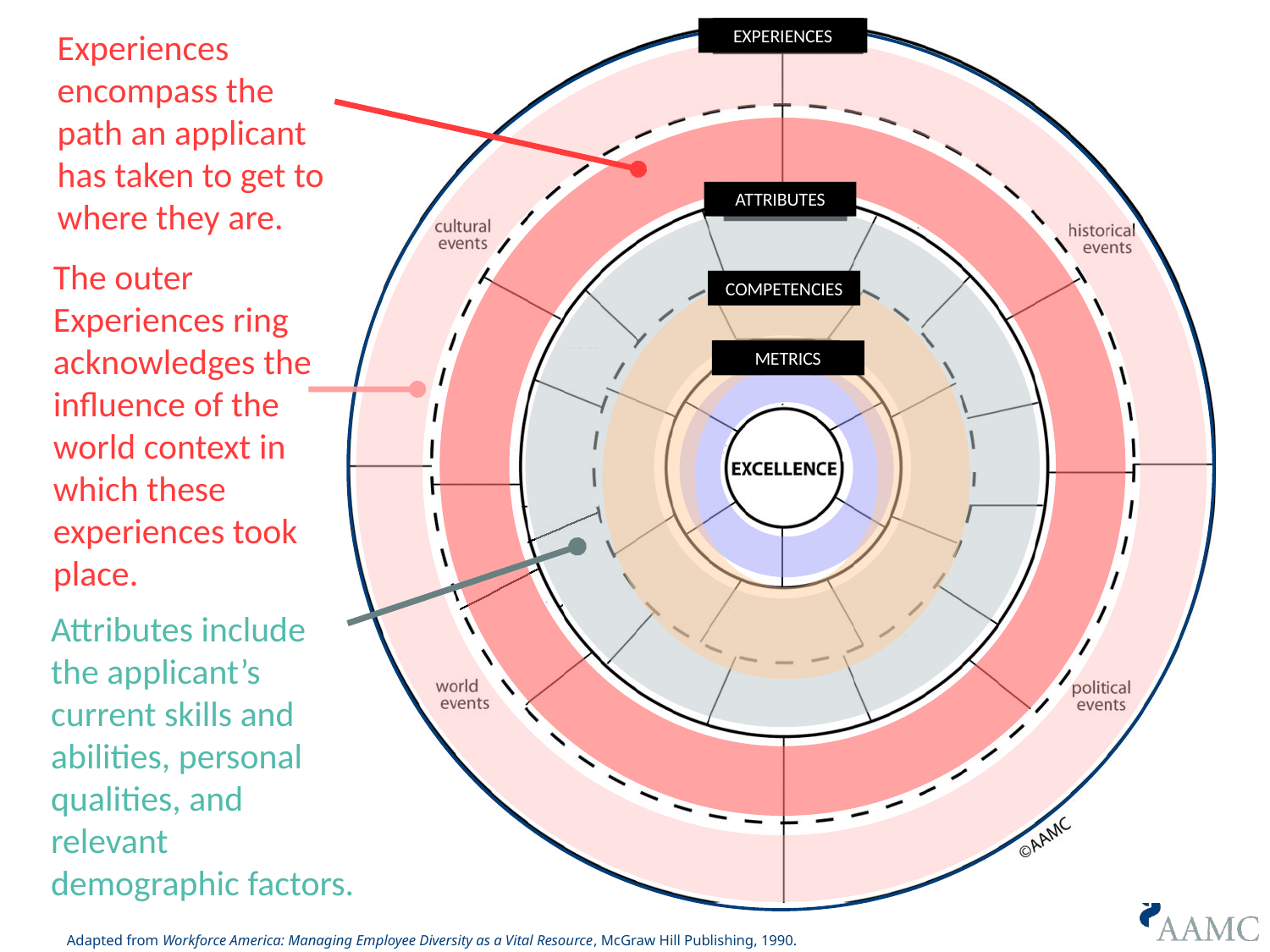

EXPERIENCES
Experiences encompass the path an applicant has taken to get to where they are.
ATTRIBUTES
The outer Experiences ring acknowledges the influence of the world context in which these experiences took place.
METRICS
Attributes include the applicant’s current skills and abilities, personal qualities, and relevant demographic factors.
COMPETENCIES
METRICS
Adapted from Workforce America: Managing Employee Diversity as a Vital Resource, McGraw Hill Publishing, 1990.

## Slide 26
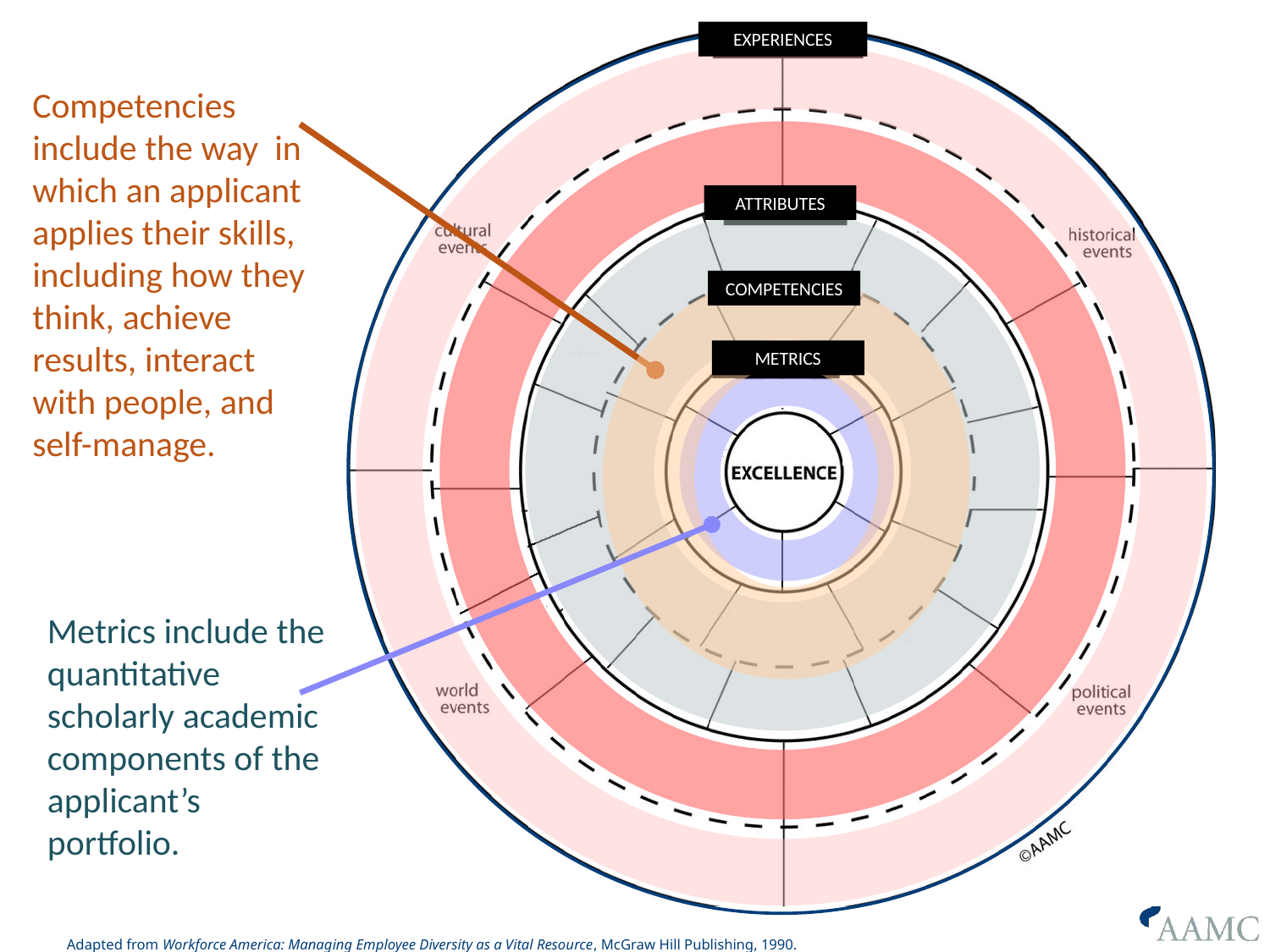

EXPERIENCES
Competencies include the way in which an applicant applies their skills, including how they think, achieve results, interact with people, and self-manage.
ATTRIBUTES
METRICS
COMPETENCIES
METRICS
Metrics include the quantitative scholarly academic components of the
applicant’s portfolio.
Adapted from Workforce America: Managing Employee Diversity as a Vital Resource, McGraw Hill Publishing, 1990.

## Slide 27
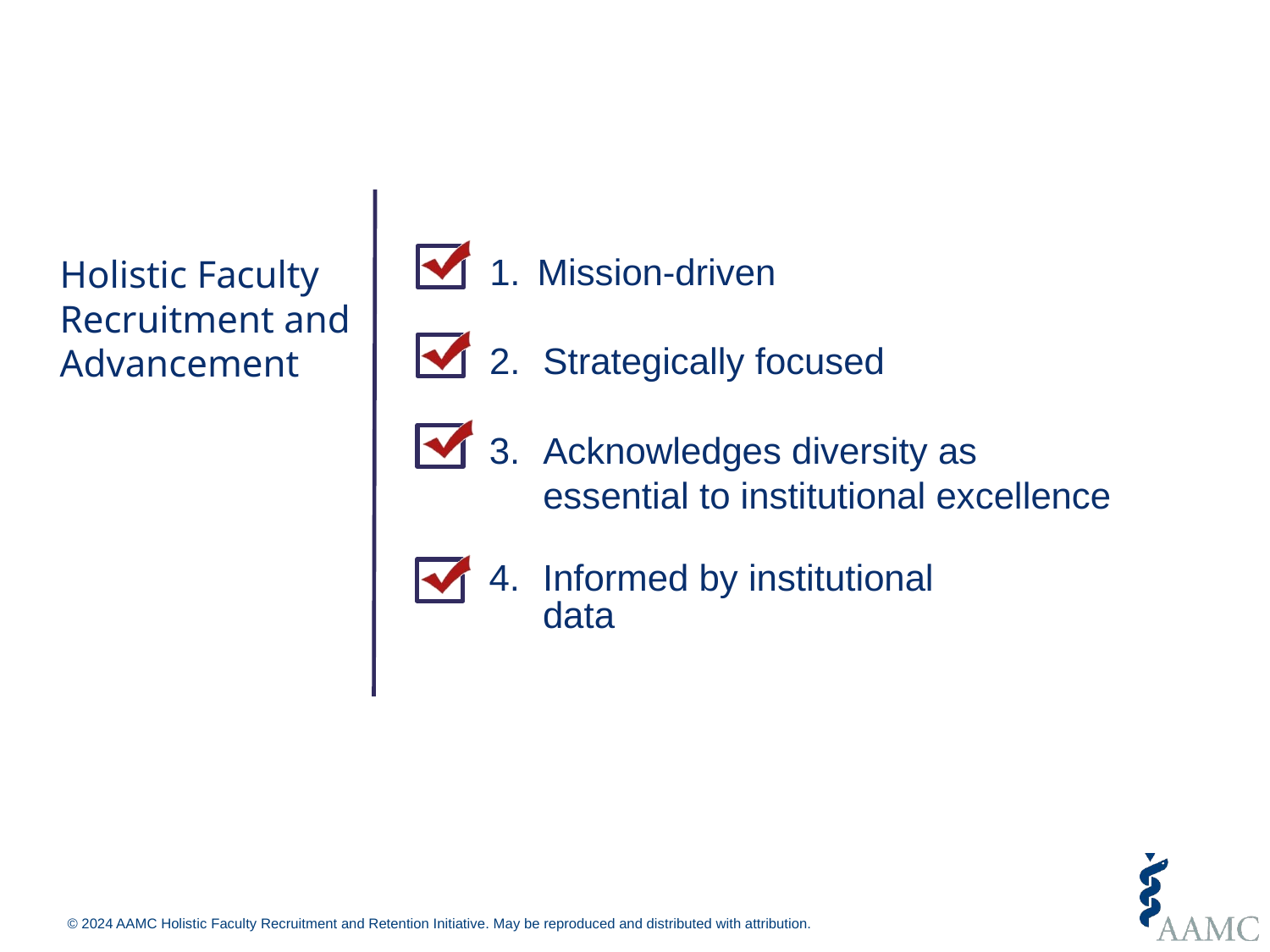

Mission-driven
Holistic Faculty Recruitment and Advancement
Strategically focused
Acknowledges diversity as essential to institutional excellence
Informed by institutional data

## Slide 28
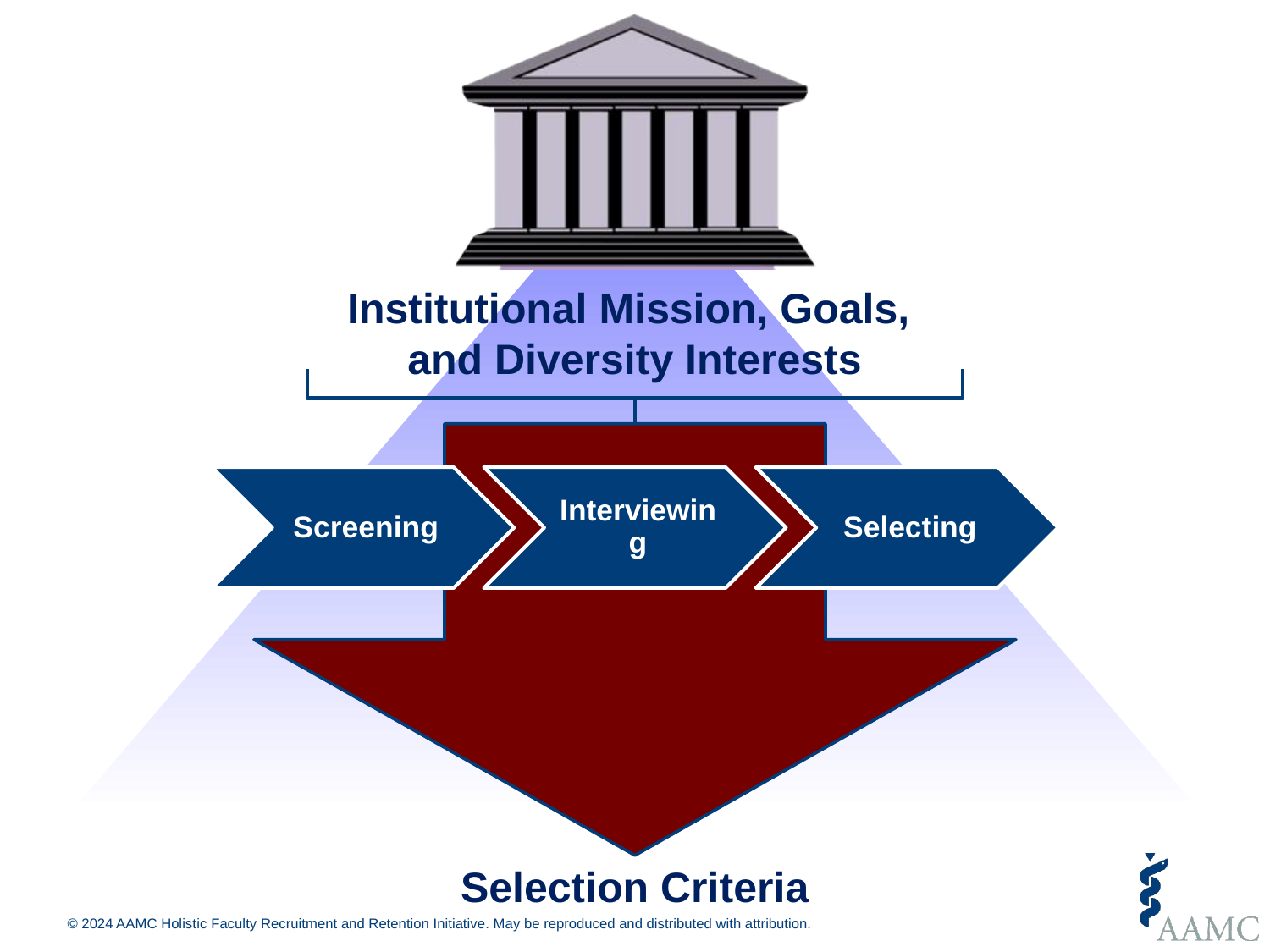

Institutional Mission, Goals,
and Diversity Interests
Selection Criteria

## Slide 29
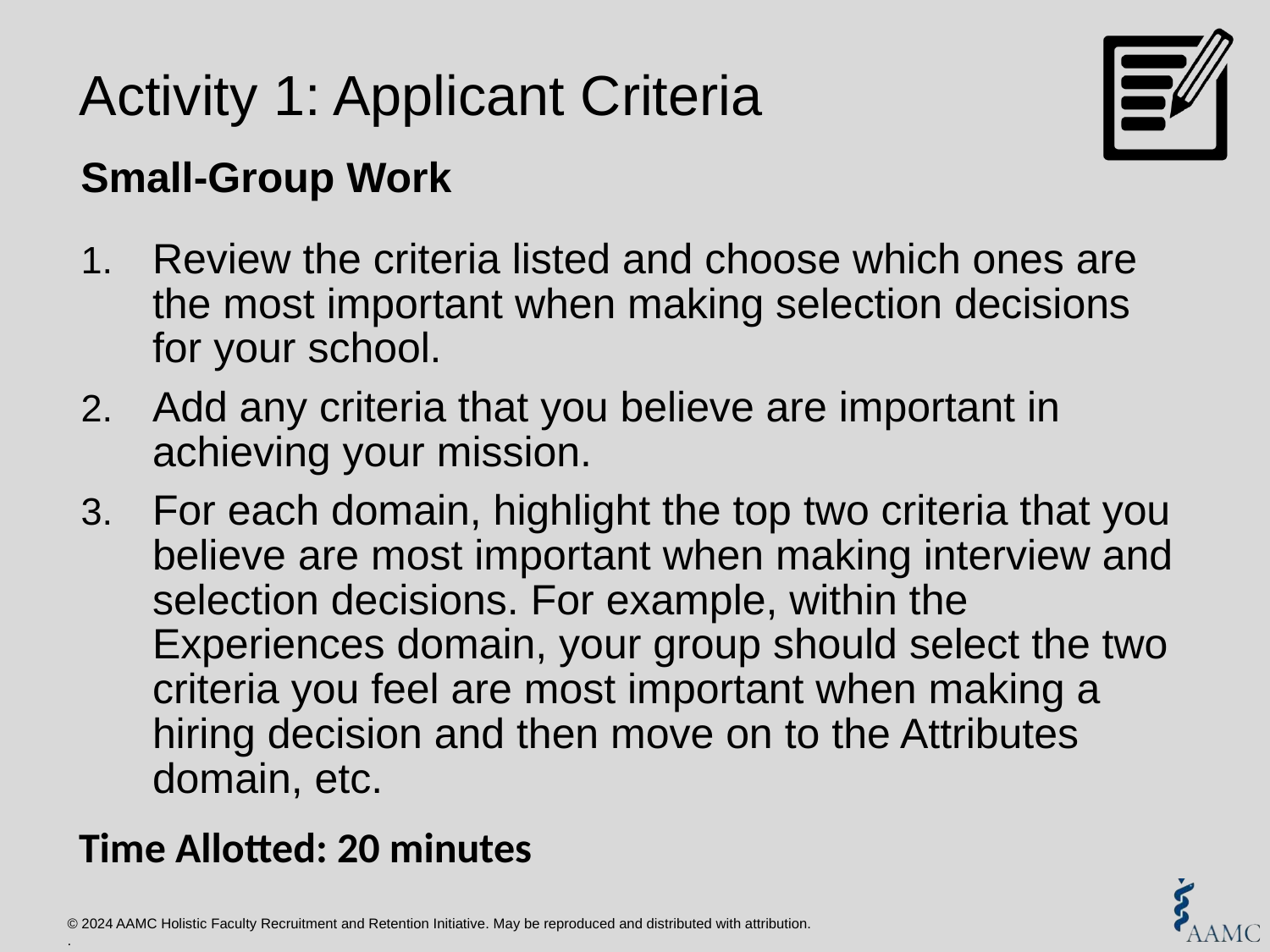

# Activity 1: Applicant Criteria
Small-Group Work
Review the criteria listed and choose which ones are the most important when making selection decisions for your school.
Add any criteria that you believe are important in achieving your mission.
For each domain, highlight the top two criteria that you believe are most important when making interview and selection decisions. For example, within the Experiences domain, your group should select the two criteria you feel are most important when making a hiring decision and then move on to the Attributes domain, etc.
Time Allotted: 20 minutes

## Slide 30
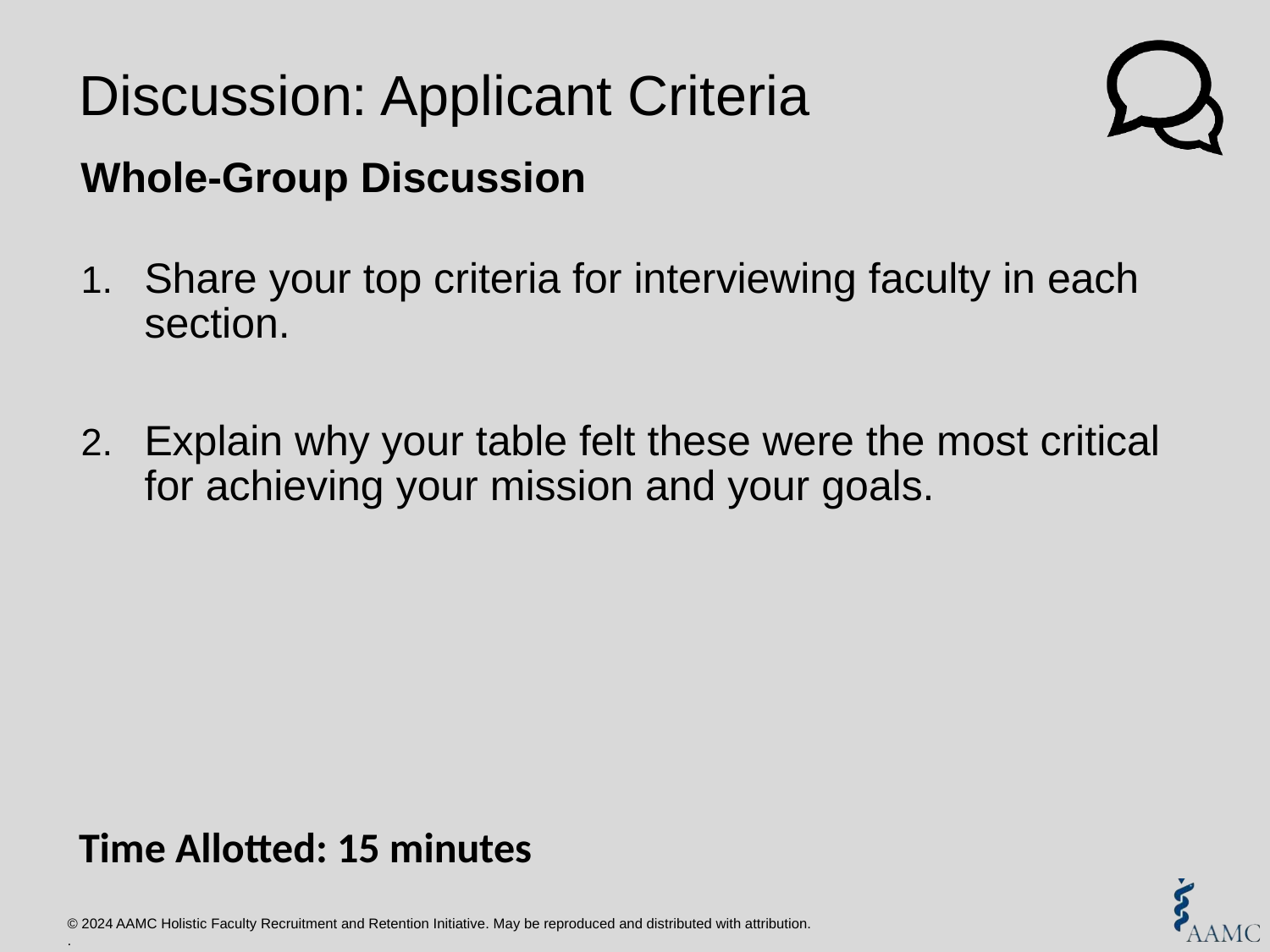

# Discussion: Applicant Criteria
Whole-Group Discussion
Share your top criteria for interviewing faculty in each section.
Explain why your table felt these were the most critical for achieving your mission and your goals.
Time Allotted: 15 minutes

## Slide 31
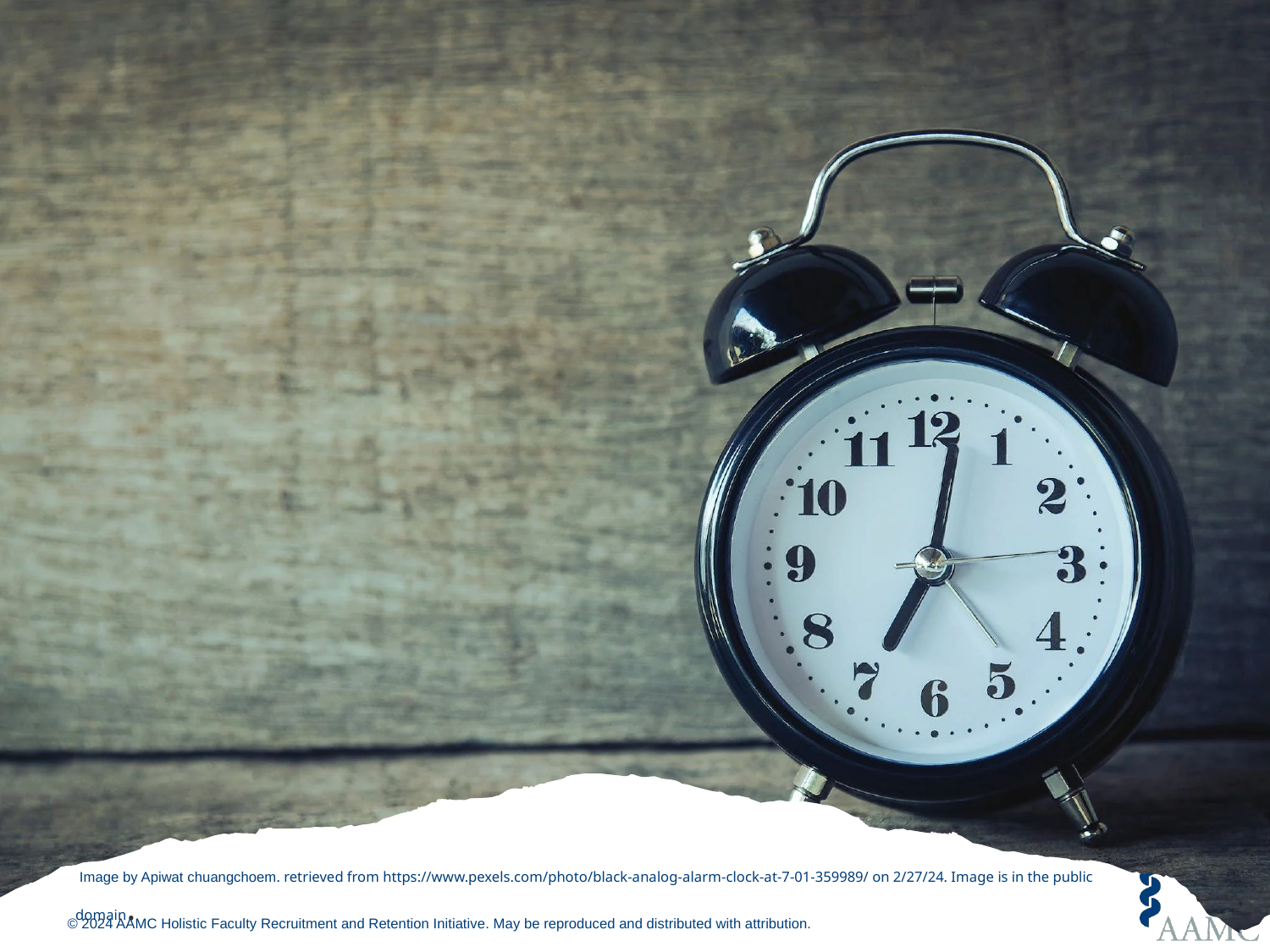

Image by Apiwat chuangchoem. retrieved from https://www.pexels.com/photo/black-analog-alarm-clock-at-7-01-359989/ on 2/27/24. Image is in the public domain.

## Slide 32
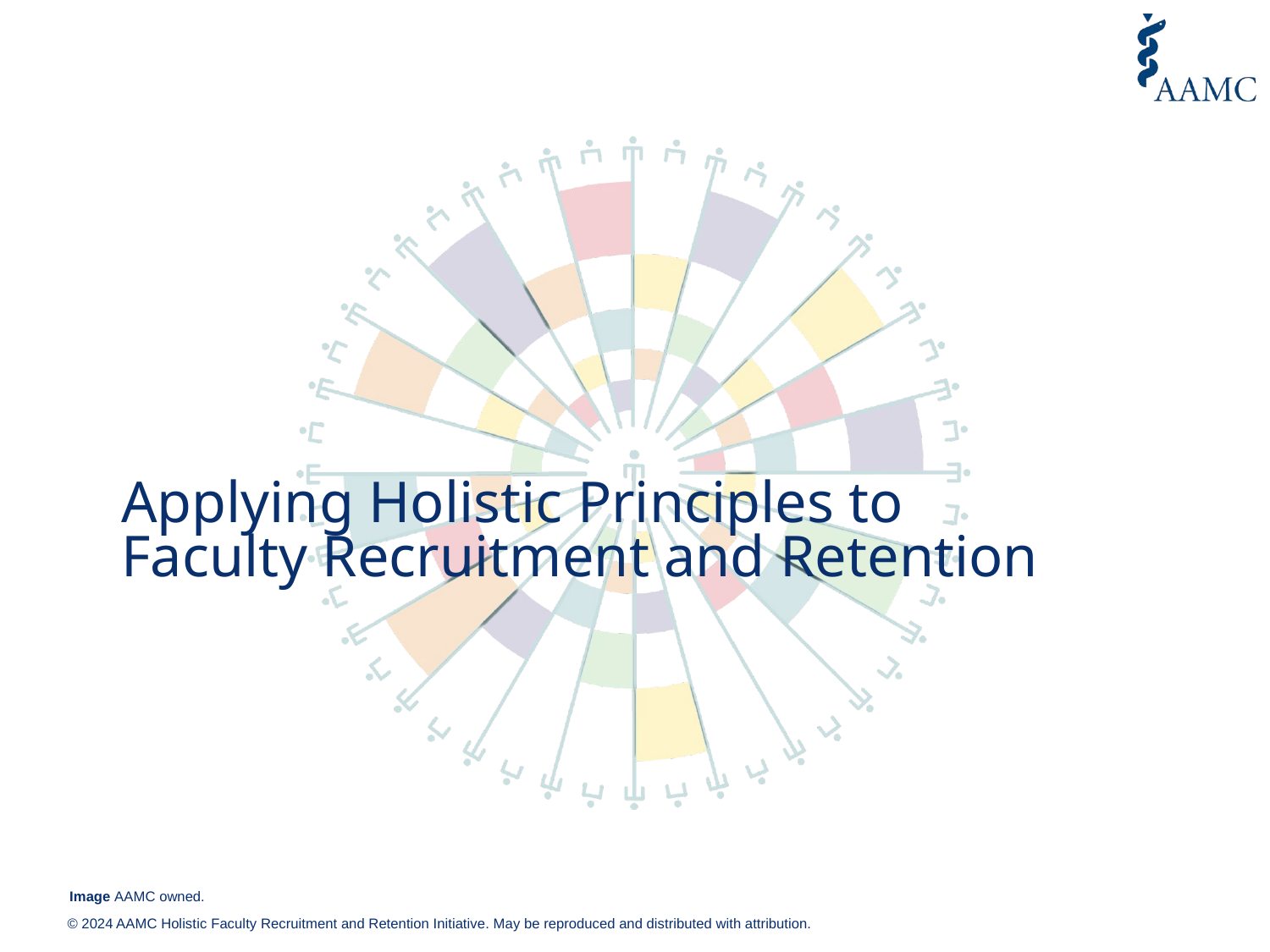

# Applying Holistic Principles to Faculty Recruitment and Retention
Image AAMC owned.

## Slide 33
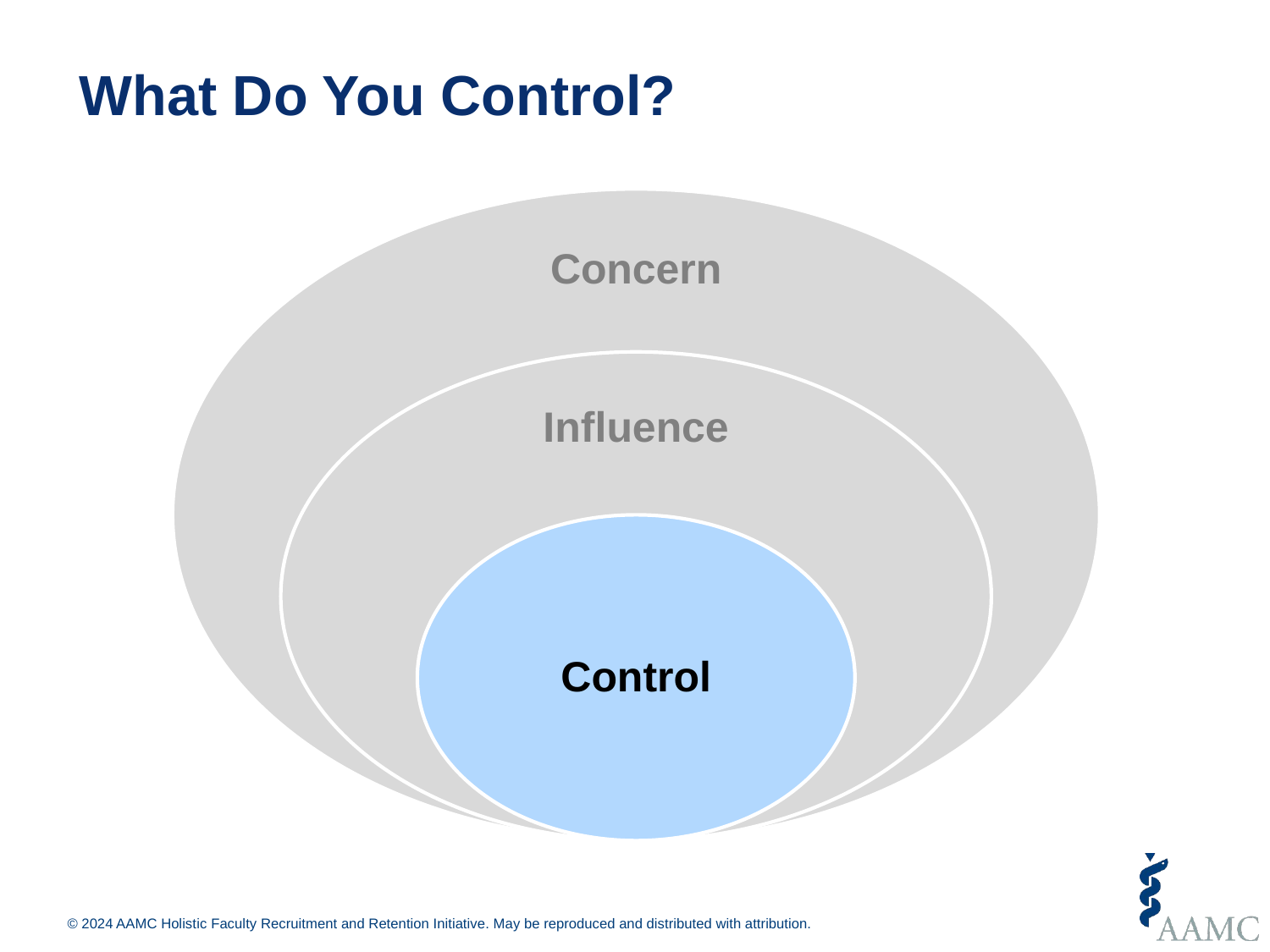

# What Do You Control?

## Slide 34
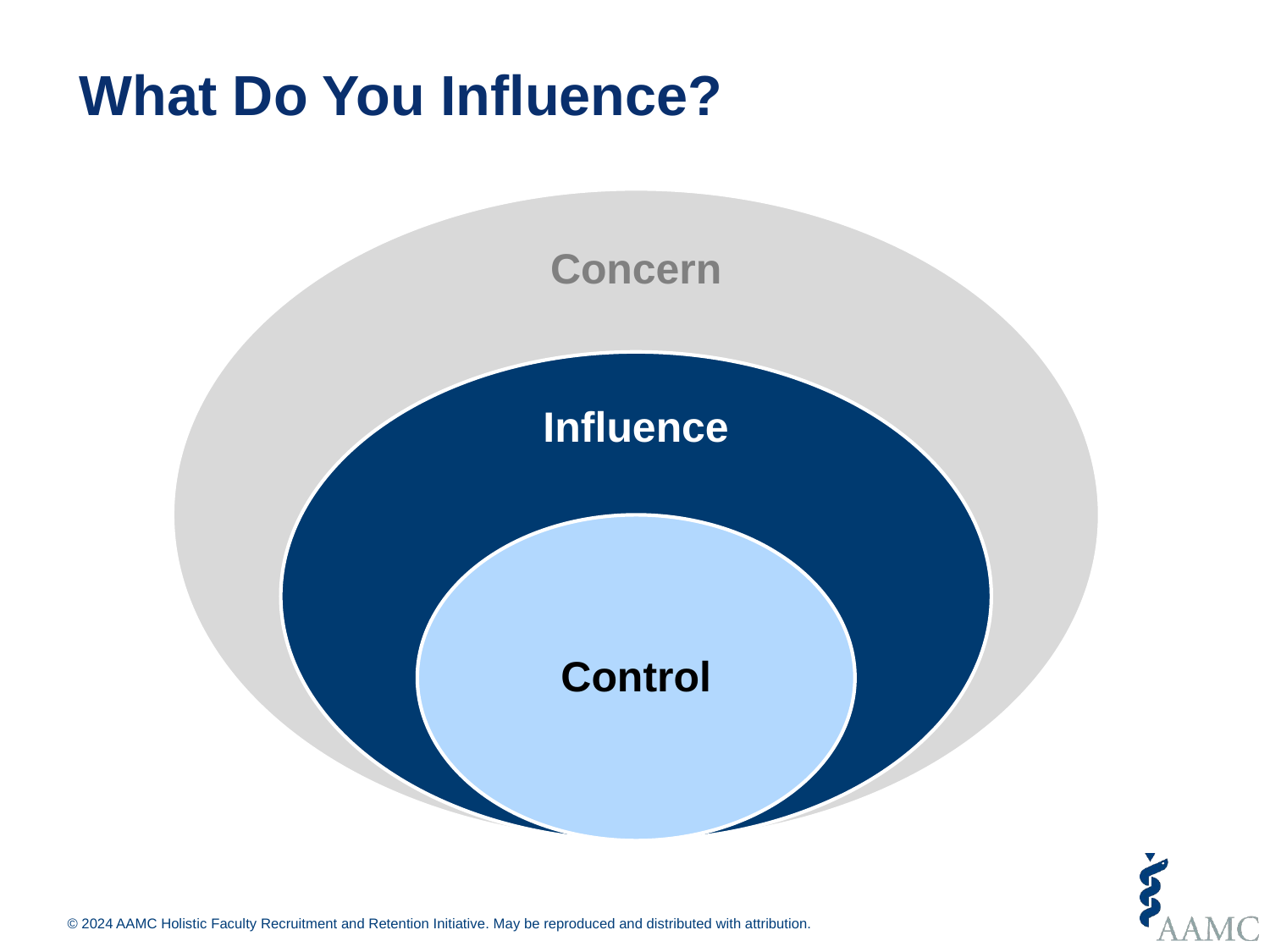

# What Do You Influence?

## Slide 35
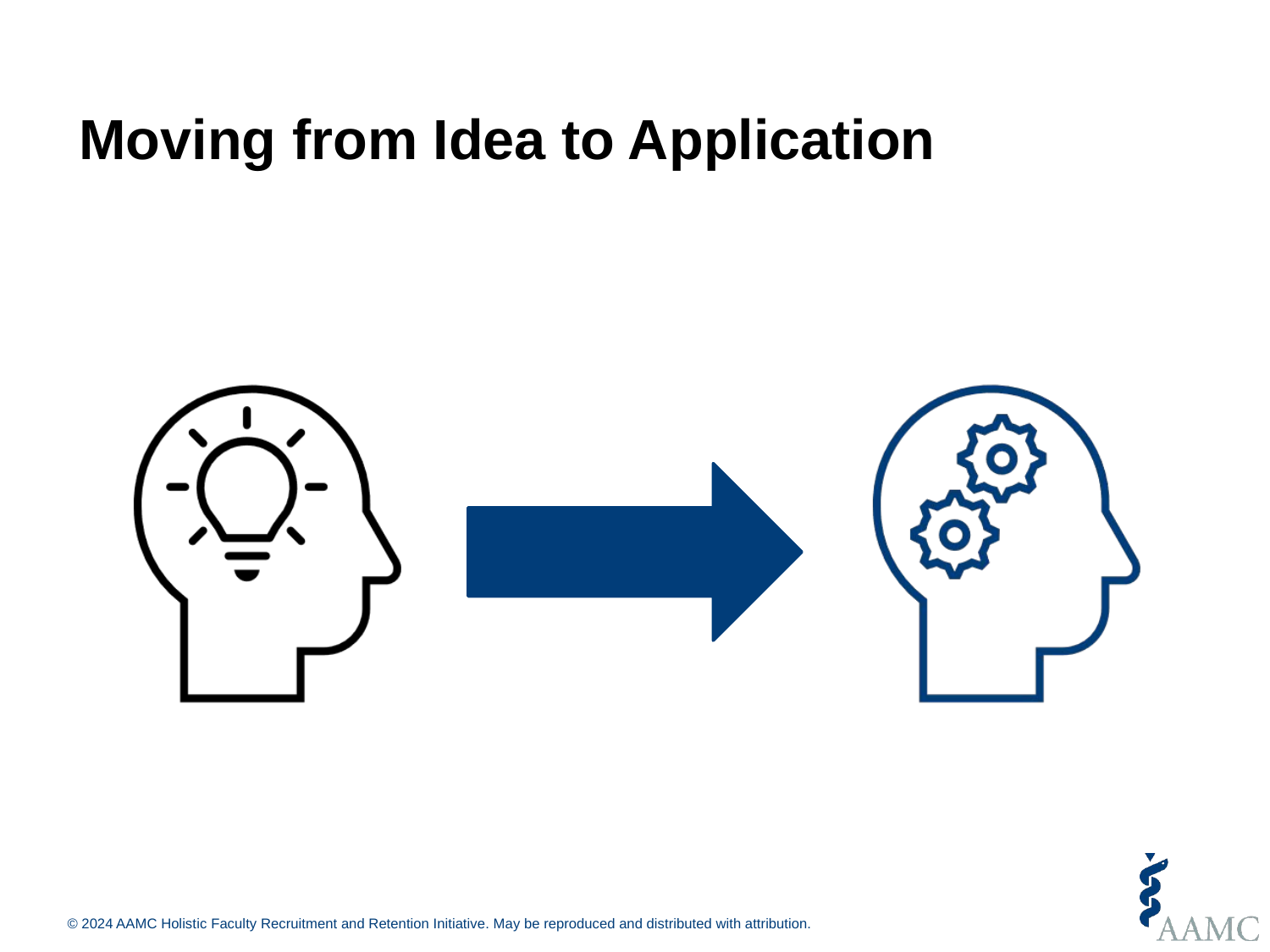

# Moving from Idea to Application

## Slide 36
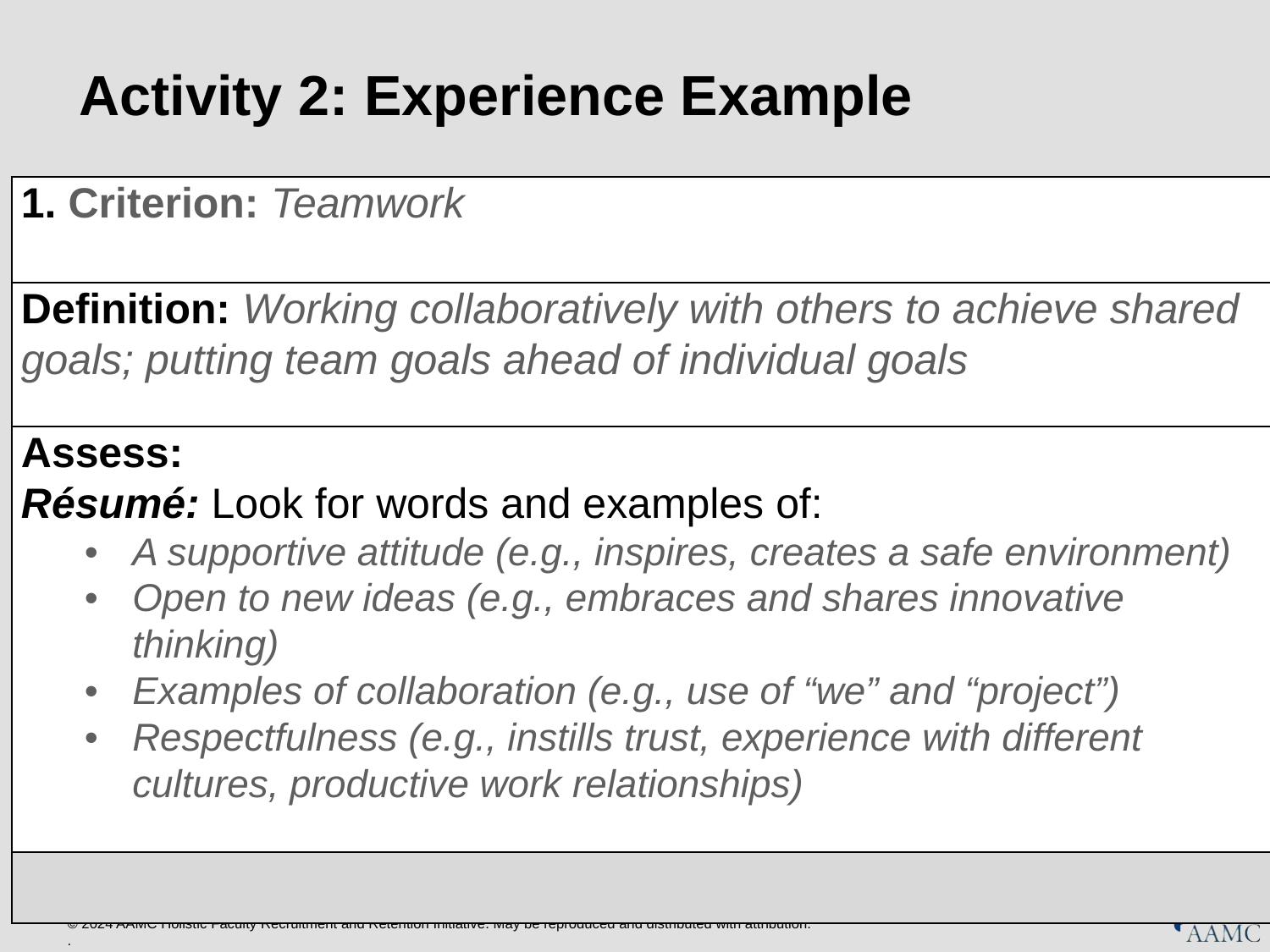

# Activity 2: Experience Example
| 1. Criterion: Teamwork |
| --- |
| Definition: Working collaboratively with others to achieve shared goals; putting team goals ahead of individual goals |
| Assess: Résumé: Look for words and examples of: A supportive attitude (e.g., inspires, creates a safe environment) Open to new ideas (e.g., embraces and shares innovative thinking) Examples of collaboration (e.g., use of “we” and “project”) Respectfulness (e.g., instills trust, experience with different cultures, productive work relationships) |
| |

## Slide 37
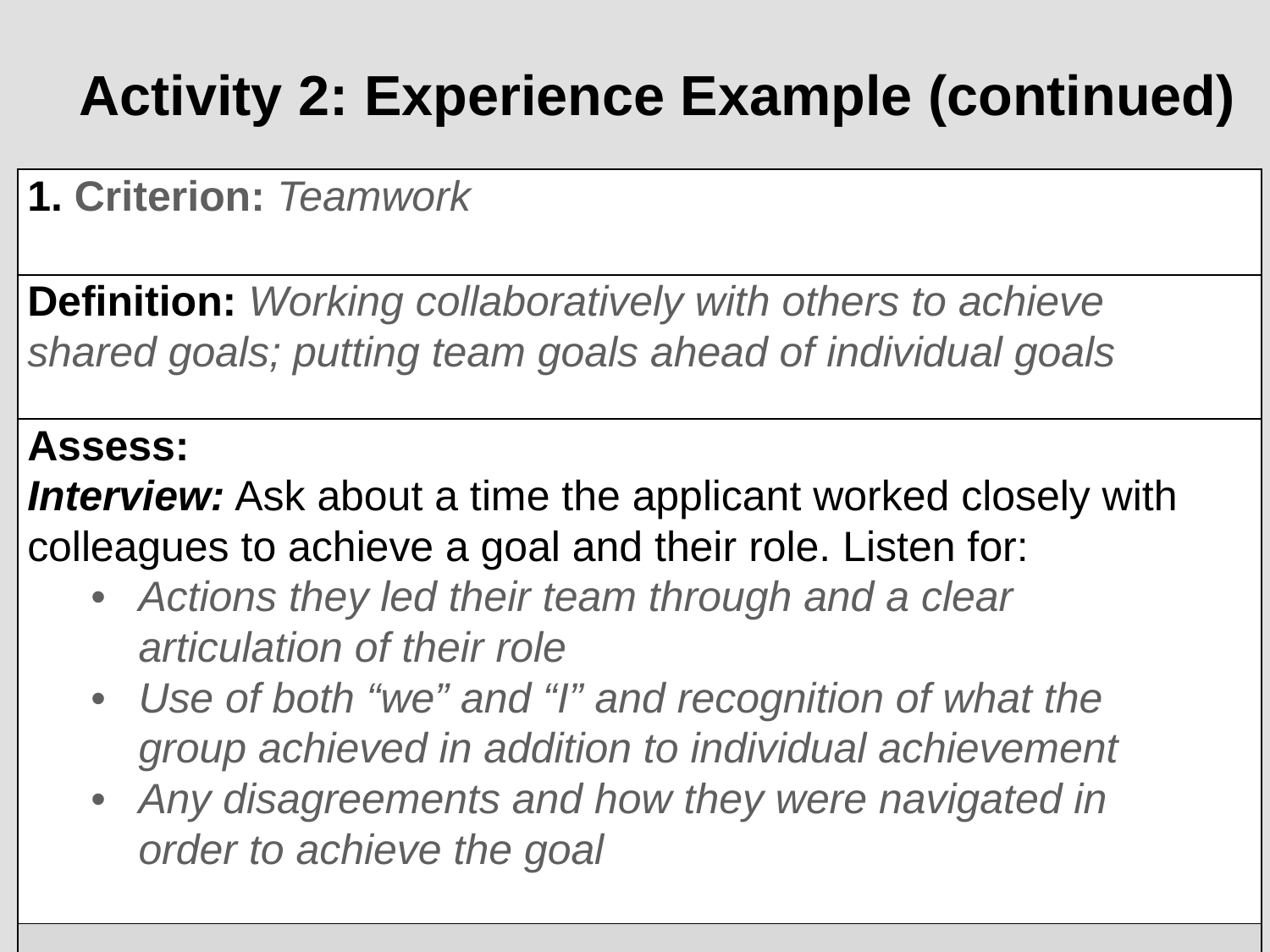

# Activity 2: Experience Example (continued)
| 1. Criterion: Teamwork |
| --- |
| Definition: Working collaboratively with others to achieve shared goals; putting team goals ahead of individual goals |
| Assess: Interview: Ask about a time the applicant worked closely with colleagues to achieve a goal and their role. Listen for: Actions they led their team through and a clear articulation of their role Use of both “we” and “I” and recognition of what the group achieved in addition to individual achievement Any disagreements and how they were navigated in order to achieve the goal |
| |

## Slide 38
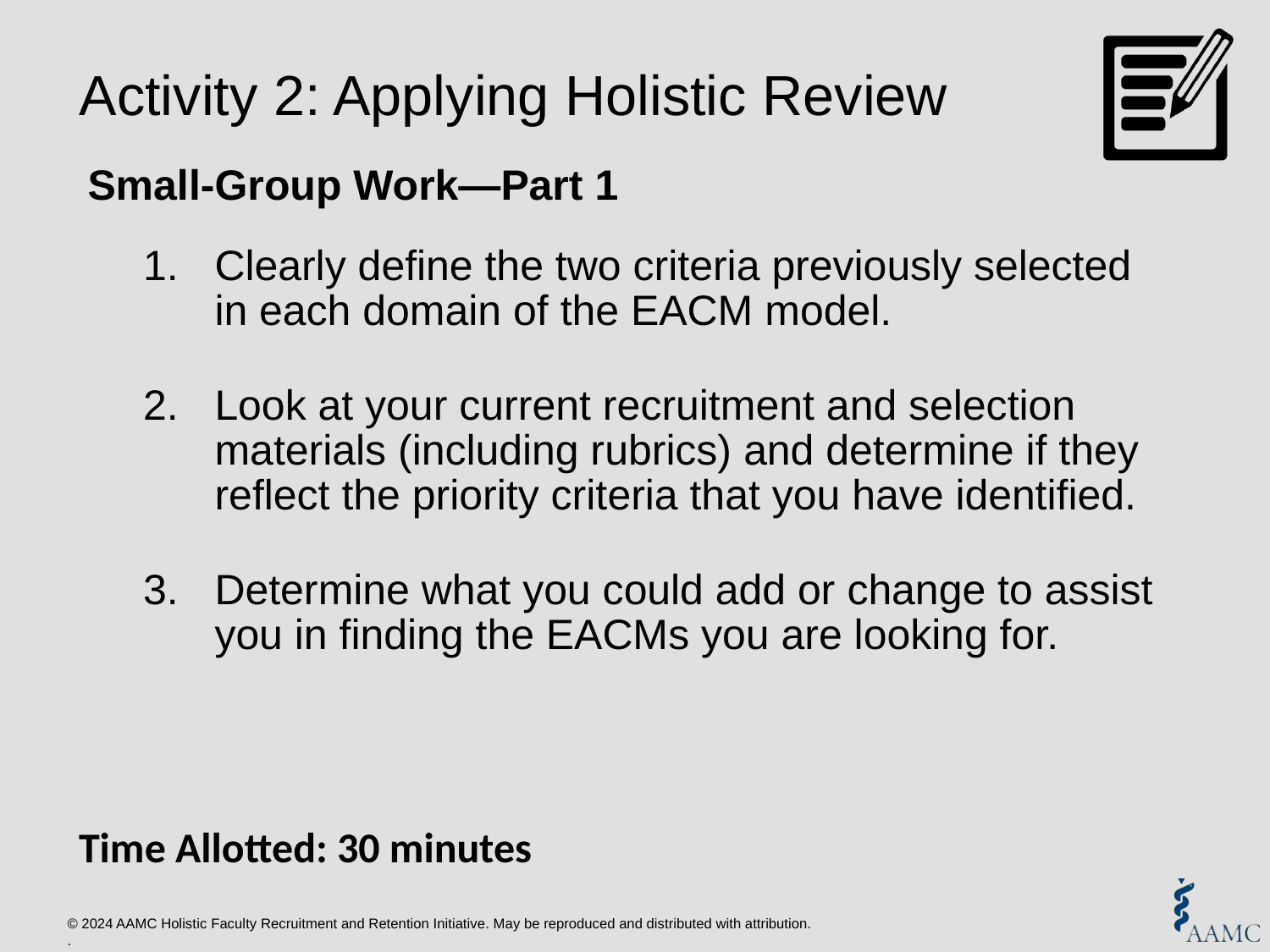

# Activity 2: Applying Holistic Review
Small-Group Work—Part 1
Clearly define the two criteria previously selected in each domain of the EACM model.
Look at your current recruitment and selection materials (including rubrics) and determine if they reflect the priority criteria that you have identified.
Determine what you could add or change to assist you in finding the EACMs you are looking for.
Time Allotted: 30 minutes

## Slide 39
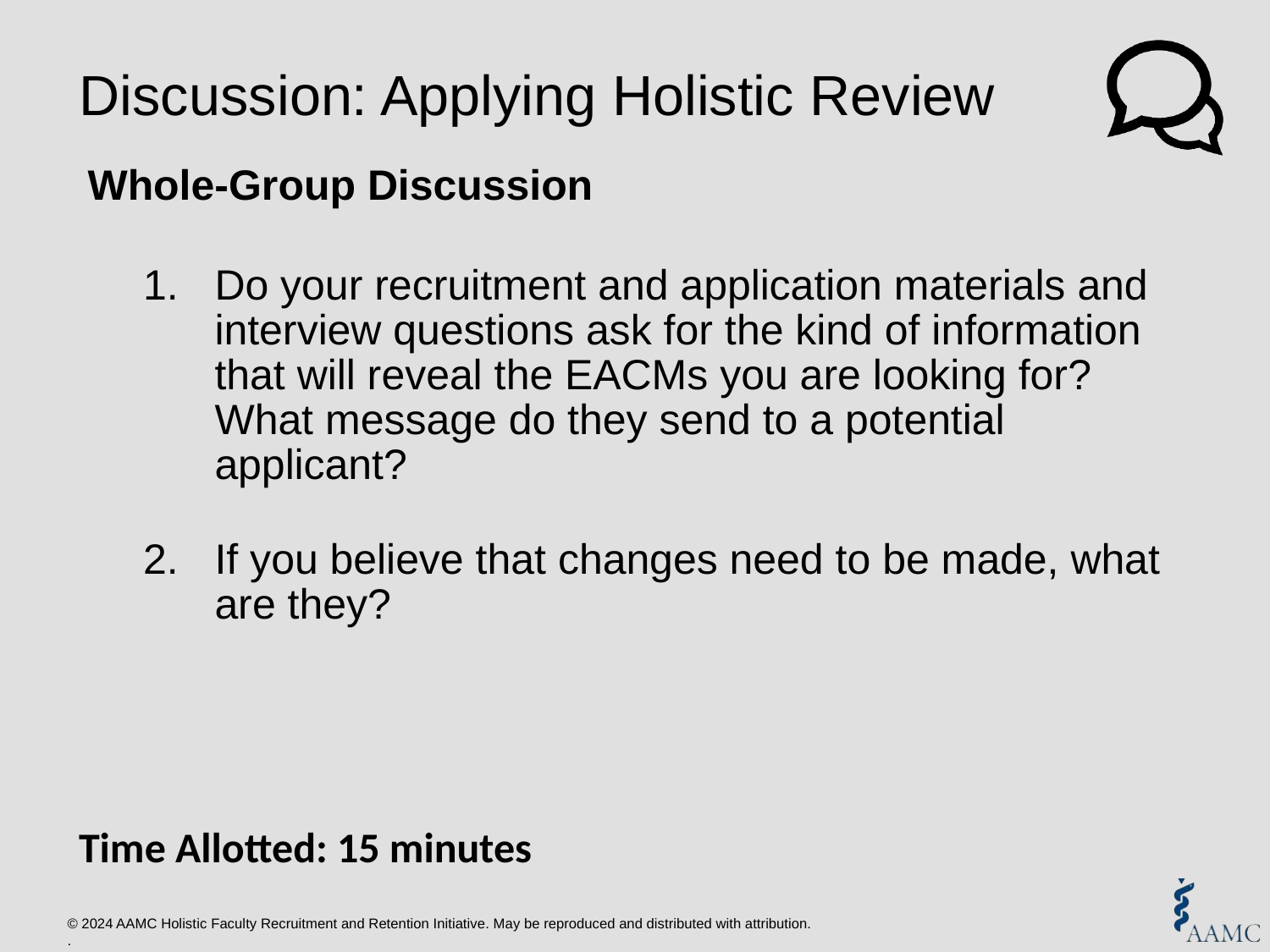

# Discussion: Applying Holistic Review
Whole-Group Discussion
Do your recruitment and application materials and interview questions ask for the kind of information that will reveal the EACMs you are looking for? What message do they send to a potential applicant?
If you believe that changes need to be made, what are they?
Time Allotted: 15 minutes

## Slide 40
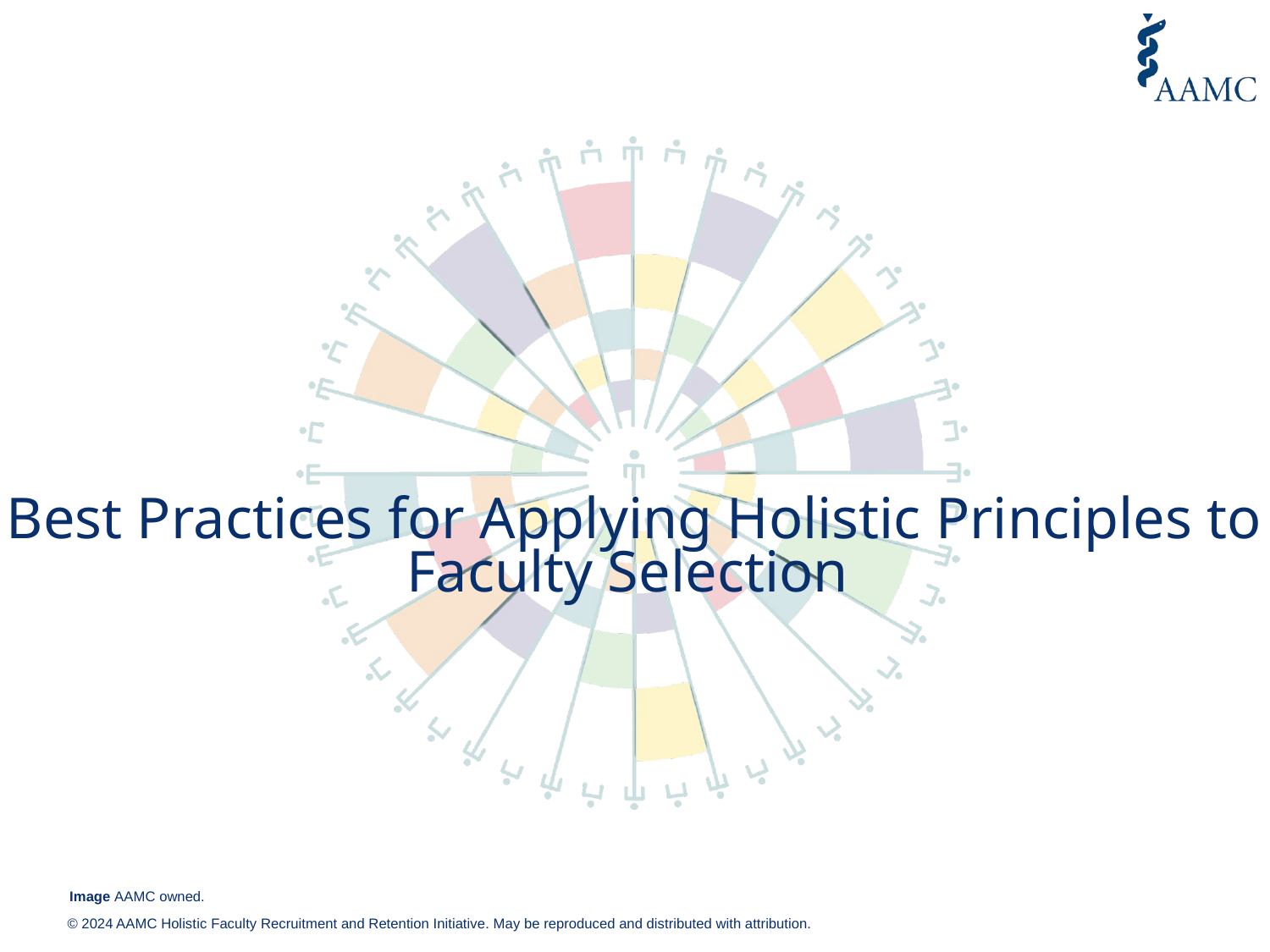

# Best Practices for Applying Holistic Principles to Faculty Selection
Image AAMC owned.

## Slide 41
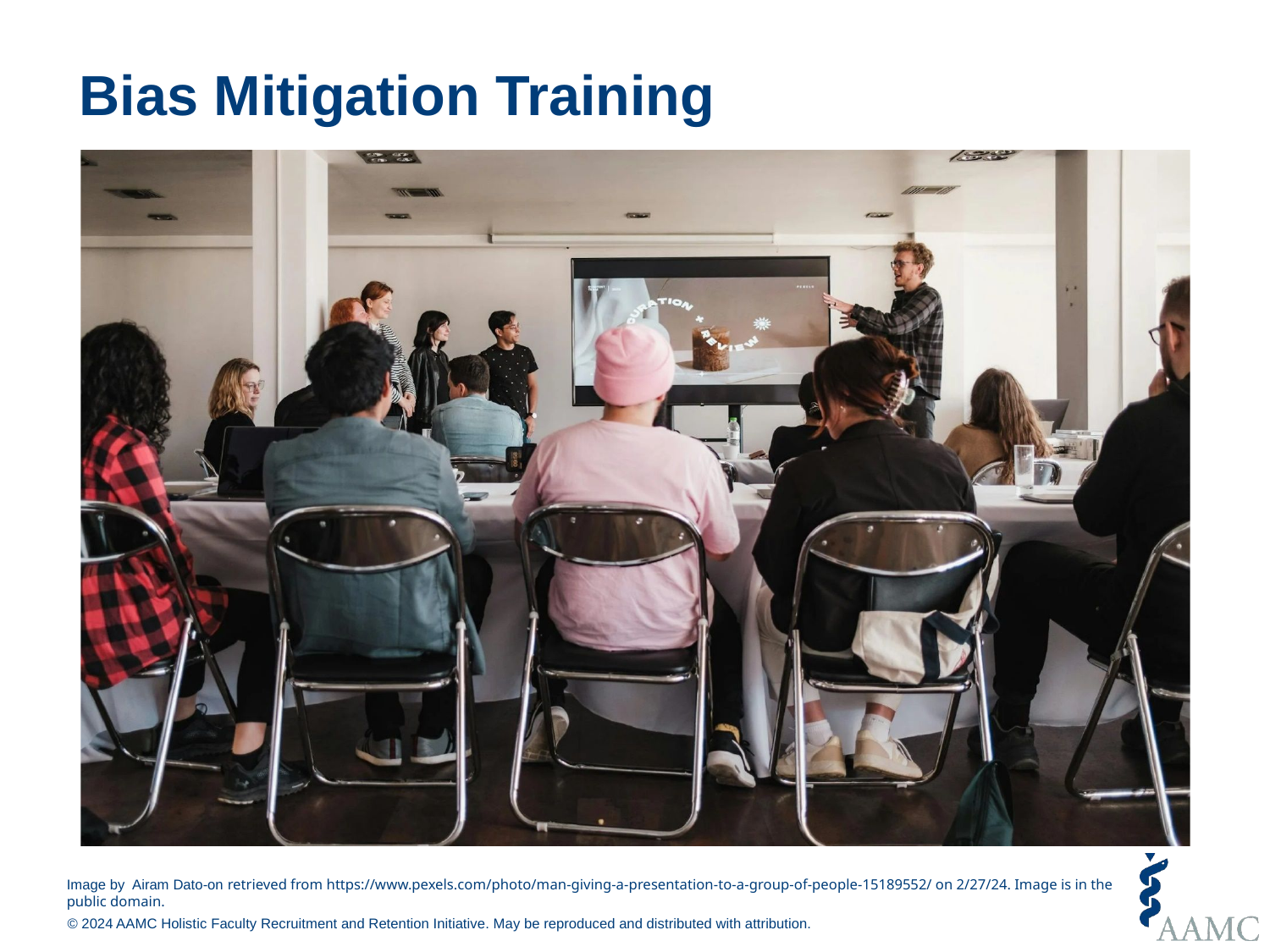

# Bias Mitigation Training
Image by Airam Dato-on retrieved from https://www.pexels.com/photo/man-giving-a-presentation-to-a-group-of-people-15189552/ on 2/27/24. Image is in the public domain.

## Slide 42
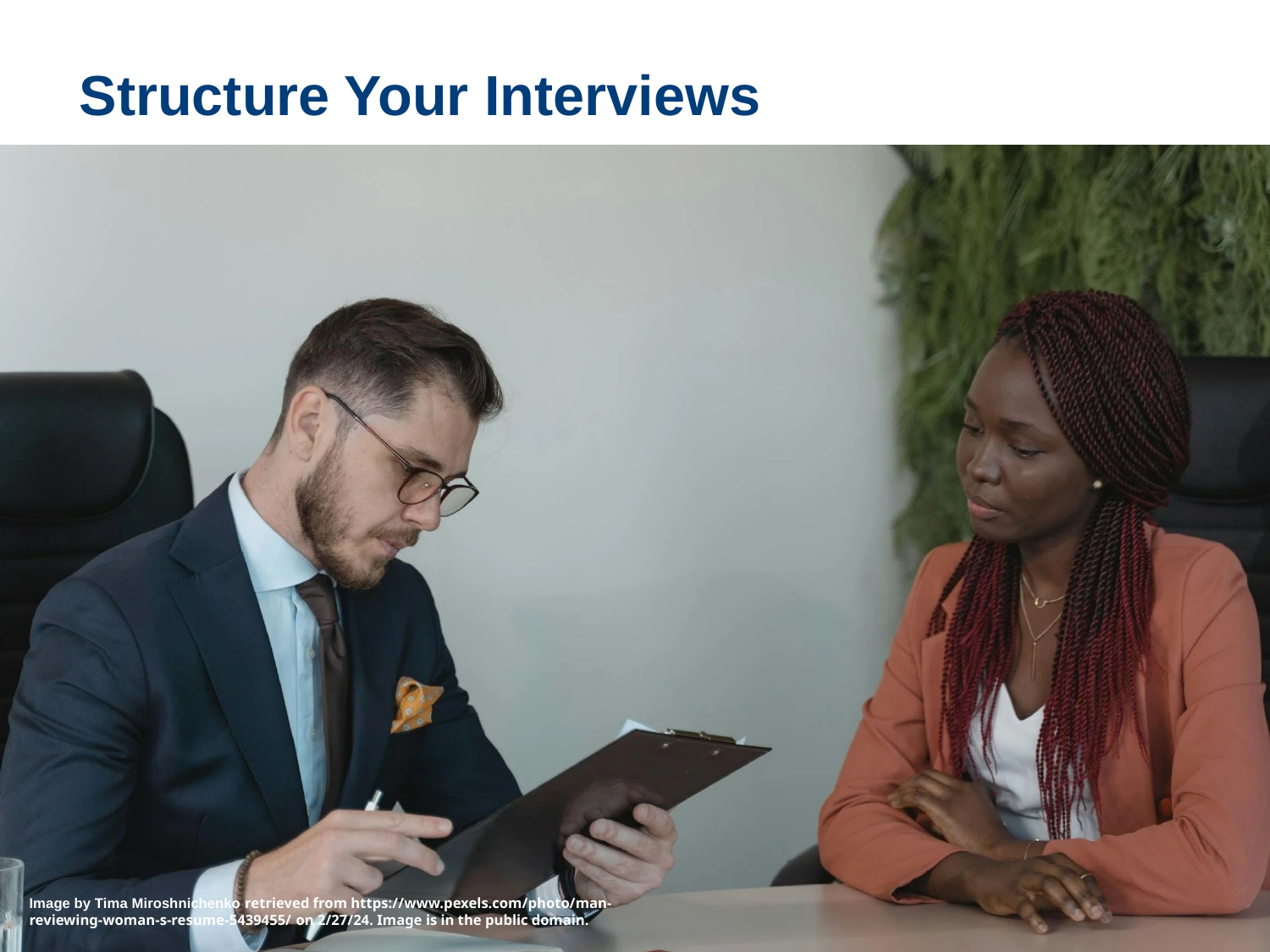

# Structure Your Interviews
Image by Tima Miroshnichenko retrieved from https://www.pexels.com/photo/man-reviewing-woman-s-resume-5439455/ on 2/27/24. Image is in the public domain.

## Slide 43
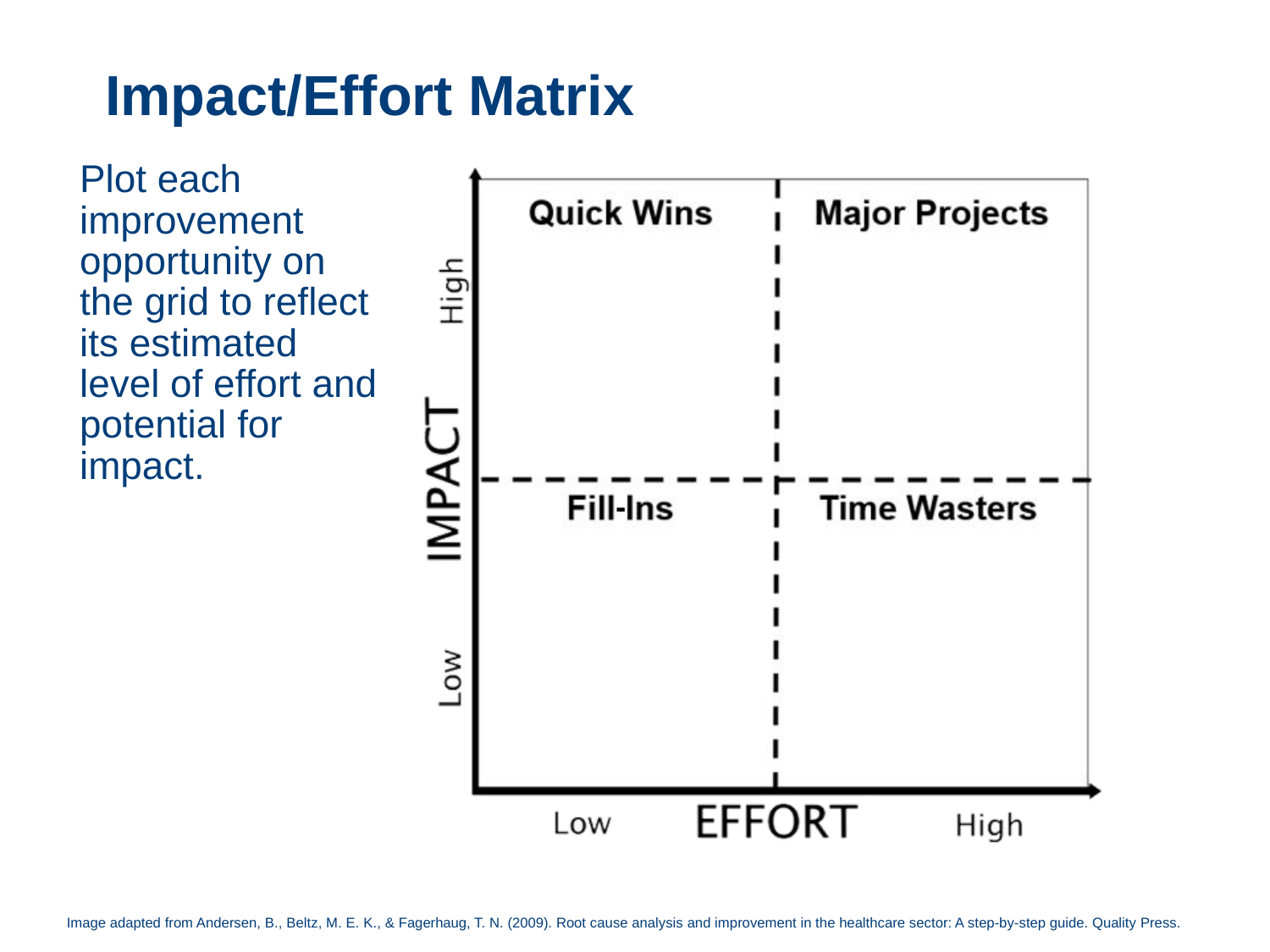

Impact/Effort Matrix
Plot each improvement opportunity on the grid to reflect its estimated level of effort and potential for impact.
Image adapted from Andersen, B., Beltz, M. E. K., & Fagerhaug, T. N. (2009). Root cause analysis and improvement in the healthcare sector: A step-by-step guide. Quality Press.

## Slide 44
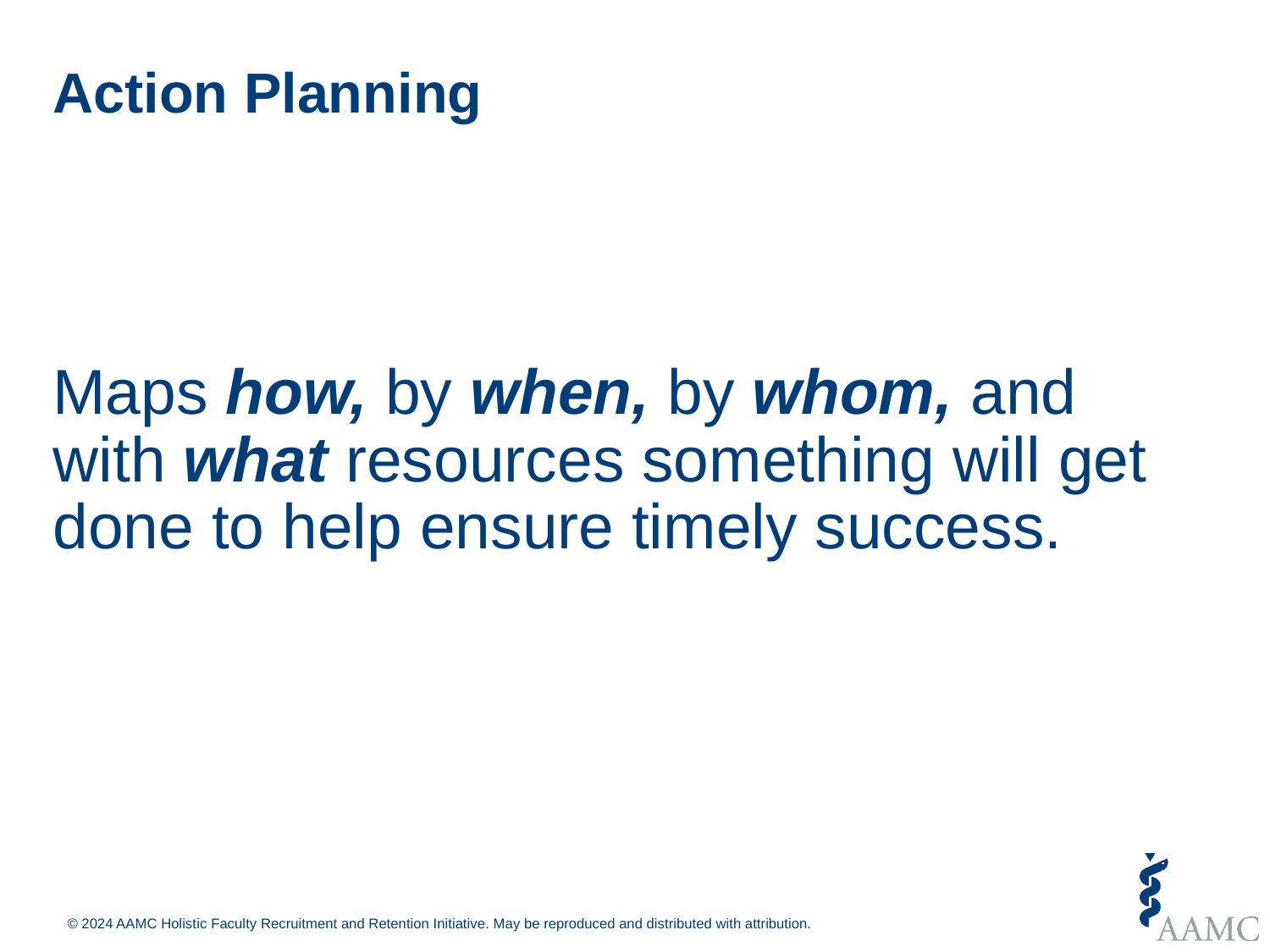

# Action Planning
Maps how, by when, by whom, and with what resources something will get done to help ensure timely success.

## Slide 45
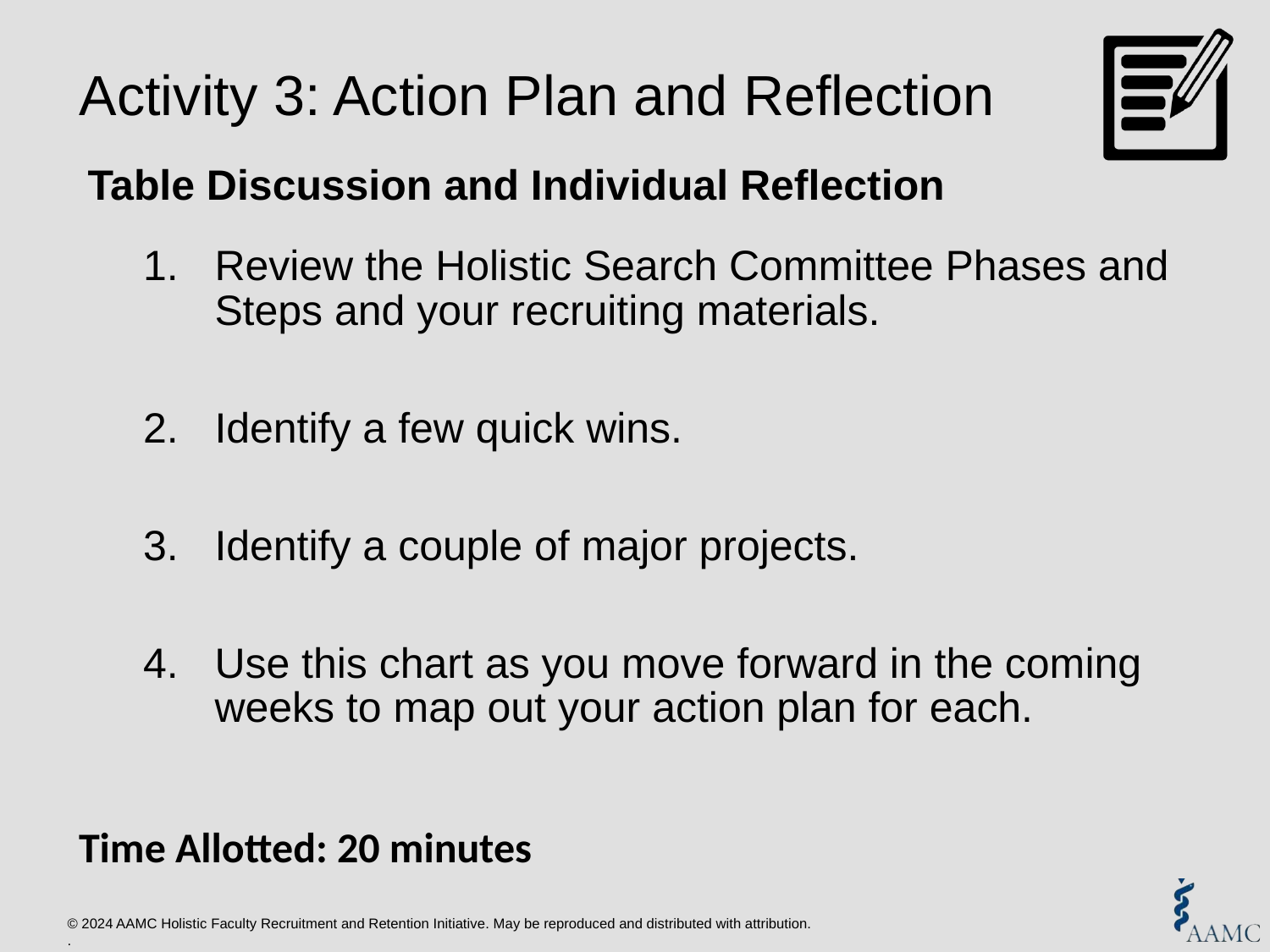

# Activity 3: Action Plan and Reflection
Table Discussion and Individual Reflection
Review the Holistic Search Committee Phases and Steps and your recruiting materials.
Identify a few quick wins.
Identify a couple of major projects.
Use this chart as you move forward in the coming weeks to map out your action plan for each.
Time Allotted: 20 minutes

## Slide 46
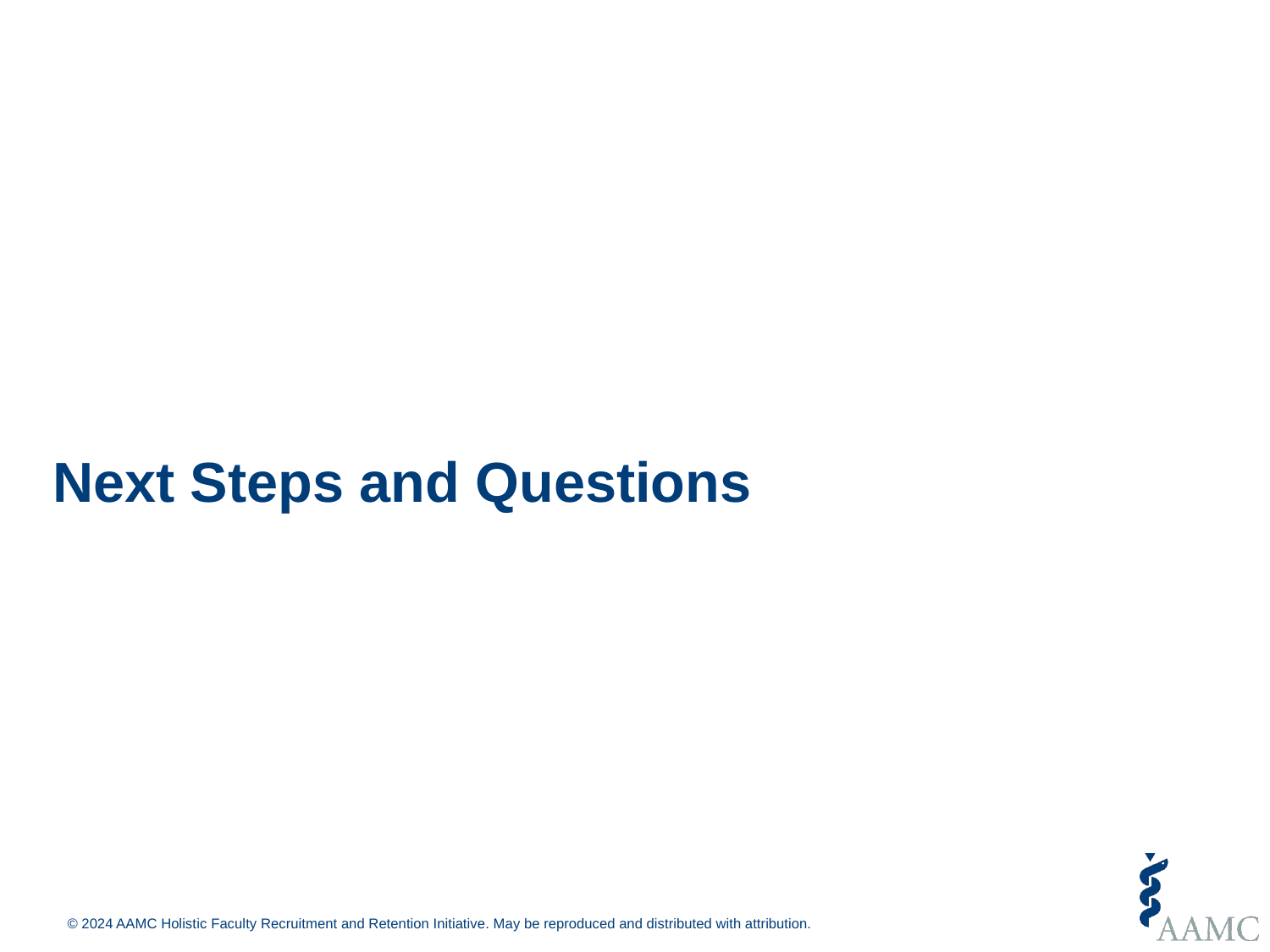

# Next Steps and Questions
