# Supplementary material for: Implementing a Holistic Review Toolkit for Faculty Recruitment and Retention
Source: MedEdPORTAL. 2024 Dec 4;20:11472. doi: 10.15766/mep_2374-8265.11472 (PMC11615027; doi:10.15766/mep_2374-8265.11472)
Supplement: Supplementary file 1 — Faculty Pilot Overview.docxOverview Equity-Minded Hiring_Step 1.docxAssess Readiness for Equity-Minded Hiring_Step 1.docxStaff Composition Inventory_Step 2.xlsxHolistic Search Committee Phases and Steps_Step 2.docxFaculty Workshop Facilitators Guide_Step 3.docxFaculty Workshop Presentation_Step 3.pptxFaculty Workshop Evaluation_Step 3.docxFaculty Workshop Activities_Step 3.docxJob Description Posting Tools and Resources_Step 4.docxInterview Questions Tools and Resources_Step 4.docxSubmission Requirements and Rating Tools_Step 4.docx360-Degree (Multisource) Reference Checking_Step 4.docxSearch Process Tools and Resources_Step 5.docxStanding Up a Search Committee_Step 5.docxMitigating Bias Resources_Step 5.docxOnboarding Tools and Resources_Step 6.docxCareer Development Discussion Guide_Step 6.docxU Colorado SOM Mentoring Resource Packet_Step 6.docxBaylor College of Medicine Exit Resources_Step 6.docxU Colorado SOM Equitable Hiring Tool_Step 7.docxHolistic Hiring and Retention Tracker_Step 8.docxEvaluation Materials Development Phase_Steps 4-6.docx [file mep_2374-8265.11472-s001.zip › H. Faculty Workshop Evaluation_Step 3.docx]

# Appendix H: Holistic Principles for Faculty Recruitment and Retention

# Workshop Evaluation

Implementation Guidance: The following sample questions reflect the workshop objectives and may be modified as necessary for your own workshop evaluation.

As a result of this workshop, please rate how confident you are currently in conducting the following where 5=extremely confident, 4=very confident, 3=somewhat confident, 2=not very confident, 1=not at all confident

| Question | 5 | 4 | 3 | 2 | 1 |
| --- | --- | --- | --- | --- | --- |
| Identifying how the composition and rank (i.e., gender, race/ethnicity) of your faculty compare at the national level. |  |  |  |  |  |
| Explaining how criteria selected to recruit, screen, interview, and select applicants support my institution’s mission and goals. |  |  |  |  |  |
| Acting on identified opportunities to change our recruitment and hiring processes, practices, and policies to achieve our desired mission-based outcomes. |  |  |  |  |  |
