# Supplementary material for: Implementing a Holistic Review Toolkit for Faculty Recruitment and Retention
Source: MedEdPORTAL. 2024 Dec 4;20:11472. doi: 10.15766/mep_2374-8265.11472 (PMC11615027; doi:10.15766/mep_2374-8265.11472)
Supplement: Supplementary file 1 — Faculty Pilot Overview.docxOverview Equity-Minded Hiring_Step 1.docxAssess Readiness for Equity-Minded Hiring_Step 1.docxStaff Composition Inventory_Step 2.xlsxHolistic Search Committee Phases and Steps_Step 2.docxFaculty Workshop Facilitators Guide_Step 3.docxFaculty Workshop Presentation_Step 3.pptxFaculty Workshop Evaluation_Step 3.docxFaculty Workshop Activities_Step 3.docxJob Description Posting Tools and Resources_Step 4.docxInterview Questions Tools and Resources_Step 4.docxSubmission Requirements and Rating Tools_Step 4.docx360-Degree (Multisource) Reference Checking_Step 4.docxSearch Process Tools and Resources_Step 5.docxStanding Up a Search Committee_Step 5.docxMitigating Bias Resources_Step 5.docxOnboarding Tools and Resources_Step 6.docxCareer Development Discussion Guide_Step 6.docxU Colorado SOM Mentoring Resource Packet_Step 6.docxBaylor College of Medicine Exit Resources_Step 6.docxU Colorado SOM Equitable Hiring Tool_Step 7.docxHolistic Hiring and Retention Tracker_Step 8.docxEvaluation Materials Development Phase_Steps 4-6.docx [file mep_2374-8265.11472-s001.zip › I. Faculty Workshop Activities_Step 3.docx]

# Appendix I: Workshop Activities

**Implementation Guidance:** This activity will help you to identify criteria that are broadly important in achieving your mission and departmental goals, irrespective of position. It is important to note that when considering a specific position, the competencies required and the EAMs that support those competencies will need to be identified for that position and search.

##### **Activity 1 - Identifying Priority Applicant Criteria**

## Directions:

#### Time allotted: 20 minutes

1. Decide who will serve in the following roles:
   1. Facilitator: Will facilitate the conversation and report-out for your breakout group
   2. Scribe: Will complete the document through screen-share if virtual or through a shared document if in person
   3. Reporter for the after-activity debrief
2. Choose the two most important criteria in each EACM domain by doing the following:
   1. Review the criteria listed (which come from the Baylor College of Medicine) and discuss which ones are the most important when making hiring decisions within your group (e.g., department, team, institution).
   2. You can add any criteria that you believe are important in achieving your mission and departmental goals.
   3. For each domain, **highlight the top two criteria** that your group believes are *most important when hiring* within your group. For example, within the Experiences domain, your group should select the two criteria you feel are most important when making a hiring decision and then move on to the Attributes domain, etc.

| Sample Criteria for Experiences:  - Educational background - Teaching experience - Professional associations - Exposure to diverse work settings - Community service - Health care experience - Leadership roles - Experience with diverse populations - Research experience - Life experiences | Sample Criteria for Attributes:  - Professionalism - Skills and abilities - Professional stature - Cultural humility - Political savvy - Integrity - Intellectual curiosity - Innovation - Languages spoken - Team-minded/team player - Leadership - Values and beliefs - Individual interests |
| --- | --- |

### Sample Criteria for Competencies

- Dealing with people
  - Establishing focus
  - Providing motivational support
  - Fostering teamwork
  - Empowering others
  - Managing change
  - Developing others
  - Managing performance
  - Oral communication
  - Written communication
  - Interpersonal awareness
  - Influencing others
  - Building collaborative relationships
- Preventing and solving problems
  - Diagnostic information gathering
  - Analytical thinking
  - Forward thinking
  - Conceptual thinking
  - Strategic thinking
  - Technical expertise
- Achieving results
  - Initiative
  - Fostering innovation
  - Results orientation
  - Thoroughness
  - Decisiveness
- Self-management
  - Self-confidence
  - Stress management
  - Personal credibility
  - Flexibility

### Sample Criteria for Metrics:

- Publications
- Teaching evaluations
- Grants
- Board certifications
- Patents
- Honors and awards

Sample Experiences, Attributes, and Metrics were developed at Baylor College of Medicine.

Sample Competencies were adapted from: Cripe EJ. 31 Core Competencies Explained. Workforce.com. September 3, 2002. Accessed April 4, 2024. <https://workforce.com/news/31-core-competencies-explained>

**Implementation Guidance:** This activity will help you to define criteria that are broadly important in achieving your mission and departmental goals, irrespective of position. It is important to note that when considering a specific position, the competencies required and the EAMs that support those competencies will need to be defined for that position and search.

##### **Activity 2 - Defining Priority Applicant Criteria**

## Directions:

#### Time allotted: 30 minutes

For each prioritized applicant criterion:

1. Determine who will report out for your table.
2. Select two criteria in each domain in the EACM model. Clearly define each of those criteria.
3. Look at your current recruitment and interview materials and determine if they reflect the priority criteria that you have identified.
4. Determine what you could add or change to assist you in finding the Experiences, Attributes, Competencies, and Metrics you are looking for.

### Faculty Selection Criteria

| **EXPERIENCES** | **1.** *Criterion:* |
| --- | --- |
|  | **Definition:** *How do you define it?* |
|  | **Assess:** *What evidence will satisfy this requirement? Do my current recruitment and selection materials allow me to assess this criterion? What, if any, changes are needed?* |
|  |  |
|  | **2.** *Criterion:* |
|  | **Definition:** *How do you define it?* |
|  | **Assess:** *What evidence will satisfy this requirement? Do my current recruitment and selection materials allow me to assess this criterion? What, if any, changes are needed?* |

### Faculty Selection Criteria

| **ATTRIBUTES** | **1.** *Criterion:* |
| --- | --- |
|  | **Definition:** *How do you define it?* |
|  | **Assess:** *What evidence will satisfy this requirement? Do my current recruitment and selection materials allow me to assess this criterion? What, if any, changes are needed?* |
|  |  |
|  | **2.** *Criterion:* |
|  | **Definition:** *How do you define it?* |
|  | **Assess:** *What evidence will satisfy this requirement? Do my current recruitment and selection materials allow me to assess this criterion? What, if any, changes are needed?* |

**Notes:**

### Faculty Selection Criteria

| **COMPETENCIES** | **1.** *Criterion:* |
| --- | --- |
|  | **Definition:** *How do you define it?* |
|  | **Assess:** *What evidence will satisfy this requirement? Do my current recruitment and selection materials allow me to assess this criterion? What, if any, changes are needed?* |
|  |  |
|  | **2.** *Criterion:* |
|  | **Definition:** *How do you define it?* |
|  | **Assess:** *What evidence will satisfy this requirement? Do my current recruitment and selection materials allow me to assess this criterion? What, if any, changes are needed?* |

**Notes:**

### Faculty Selection Criteria

| **METRICS** | **1.** *Criterion:* |
| --- | --- |
|  | **Definition:** *How do you define it?* |
|  | **Assess:** *What evidence will satisfy this requirement? Do my current recruitment and selection materials allow me to assess this criterion? What, if any, changes are needed?* |
|  |  |
|  | **2.** *Criterion:* |
|  | **Definition:** *How do you define it?* |
|  | **Assess:** *What evidence will satisfy this requirement? Do my current recruitment and selection materials allow me to assess this criterion? What, if any, changes are needed?* |

**Notes:**

**Implementation Guidance:** This activity will help you to review your recruiting materials against recommended practices to identify opportunities to improve your processes.

##### **Activity 3 - Action Plan and Next Steps**

## Directions:

#### Time allotted: 20 minutes

1. Review the Holistic Search Committee Phases and Steps (Appendix E) and your recruiting materials.
2. Identify a few quick wins.
3. Identify a couple of major projects.
4. Use this chart as you move forward in the coming weeks to map out your action plan for each.

|  | **Quick Wins** | **Steps Required** | **Timeline for Completing Action** | **Resources Required to Address Action** |
| --- | --- | --- | --- | --- |
| **1.** |  |  |  |  |
| **2.** |  |  |  |  |
| **3.** |  |  |  |  |

|  | **Major Projects** | **Steps Required** | **Timeline for Completing Action** | **Resources Required to Address Action** |
| --- | --- | --- | --- | --- |
| **1.** |  |  |  |  |
| **2.** |  |  |  |  |
