# Supplementary material for: Implementing a Holistic Review Toolkit for Faculty Recruitment and Retention
Source: MedEdPORTAL. 2024 Dec 4;20:11472. doi: 10.15766/mep_2374-8265.11472 (PMC11615027; doi:10.15766/mep_2374-8265.11472)
Supplement: Supplementary file 1 — Faculty Pilot Overview.docxOverview Equity-Minded Hiring_Step 1.docxAssess Readiness for Equity-Minded Hiring_Step 1.docxStaff Composition Inventory_Step 2.xlsxHolistic Search Committee Phases and Steps_Step 2.docxFaculty Workshop Facilitators Guide_Step 3.docxFaculty Workshop Presentation_Step 3.pptxFaculty Workshop Evaluation_Step 3.docxFaculty Workshop Activities_Step 3.docxJob Description Posting Tools and Resources_Step 4.docxInterview Questions Tools and Resources_Step 4.docxSubmission Requirements and Rating Tools_Step 4.docx360-Degree (Multisource) Reference Checking_Step 4.docxSearch Process Tools and Resources_Step 5.docxStanding Up a Search Committee_Step 5.docxMitigating Bias Resources_Step 5.docxOnboarding Tools and Resources_Step 6.docxCareer Development Discussion Guide_Step 6.docxU Colorado SOM Mentoring Resource Packet_Step 6.docxBaylor College of Medicine Exit Resources_Step 6.docxU Colorado SOM Equitable Hiring Tool_Step 7.docxHolistic Hiring and Retention Tracker_Step 8.docxEvaluation Materials Development Phase_Steps 4-6.docx [file mep_2374-8265.11472-s001.zip › J. Job Description Posting Tools and Resources_Step 4.docx]

Appendix J: Job Description and Posting Tools and Resources

**Baylor College of Medicine Job Description Guidelines**

Implementation Guidance: Before implementing the recommendations in this document, your institution should review federal and local laws to ensure they align with organizational policies and procedures.

These sample guidelines will help you to write a job description that will clearly communicate what the position requires (EACMs) and for what the applicant will be responsible.

**Background:** Job descriptions are an essential tool for recruiting qualified and diverse faculty members to Baylor College of Medicine (Baylor). These descriptions must clearly delineate the responsibilities of the position, basic qualifications (experiences, attributes, competencies, and academic metrics) required, where the person hired will work, and to whom she/he/they will report. Below are guidelines for announcing job openings and recruiting new faculty members to fill them.

**Position Title:** This is the first statement a potential faculty applicant will read. Include the faculty rank/title associated with the position and indicate whether these are negotiable. Identify the hiring department, include a closing date for receipt of applications (*a minimum of ten business days of posting the position is required*) and a proposed start date for the position, and state whether the job is full or part time.

**Baylor College of Medicine and Department Summary:** Include a brief overview of Baylor and the hiring department and provide website links for both. The sample text below may provide a template:

Baylor ([www.bcm.edu](http://www.bcm.edu)) is recognized as one of the nation’s premier academic health science centers and is known for excellence in education, research, and health care and community service. Located in the heart of the world’s largest medical center ([Texas Medical Center](https://en.wikipedia.org/wiki/Texas_Medical_Center)), Baylor is affiliated with multiple educational, health care and research affiliates ([Baylor Affiliates](https://www.bcm.edu/about-us/affiliates)).

The Department of ABC is . . . (2-3 sentences about the hiring department).

**Position Summary:** The Department of ABC is currently seeking to identify a faculty member with the following experiences, attributes, competencies, and academic metrics for this position.

**Dos and Don’ts for Writing a Position Summary:**

- Avoid gendered words (i.e., “competitive,” “supportive”) in job advertisements, which can repel certain genders from applying and limit the applicant pool.
  - Source: Harvard Kennedy School. Evidence that gendered wording in job advertisements exists and sustains gender inequality. Accessed April 12, 2024. <https://gap.hks.harvard.edu/evidence-gendered-wording-job-advertisements-exists-and-sustains-gender-inequality>
- Use gender neutral language instead of gendered language in job advertisements and avoid stating preferred qualifications as required qualifications because it is known that women and minority applicants are less likely to apply for a job unless they feel they meet all the qualifications.
  - Source: Collier D, Zhang C. Can we reduce bias in the recruiting process and diversify pools of candidates by using different types of words in job descriptions? Cornell University Library. Accessed April 12, 2024. <https://hdl.handle.net/1813/74363>
- Consider putting your job advertisement through a gender decoder (example below).
  - Katmanfield. Gender decoder. Accessed April 12, 2024. <http://gender-decoder.katmatfield.com/>

**Position Responsibilities:** Clearly describe the position so candidates have a clear understanding of the type of work they will be expected to perform and the environment in which they will work.

- Identify the setting in which the faculty member will work (e.g., hospitals, clinics, labs) and the individual(s) to whom he/she/they will report.
- Articulate clinical, research, and/or teaching responsibilities of the position.
- Delineate supervisory responsibilities (if any) for staff, students, trainees, and/or other faculty members.
- Add any specific functions required/desired by the department.
- Include specific measurable and attainable performance goals for the position.

**Position Qualifications:** List the minimum requirements (experiences, attributes, competencies, and metrics) needed to qualify for the position.

- Experiences: Previous clinical, research, teaching, patient safety, diverse employment settings, community engagement, and/or service
- Attributes: Languages spoken, cultural competence, team-player (Baylor values)
- Competencies: Working with people, preventing and solving problems, achieving results, self-management.
- Metrics:
  - Education: required or desired terminal degree
  - Post-terminal degree education (e.g., residency, postdoctoral training/fellowships)
  - Certifications/registrations/licensure required for the position
  - Other desired academic metrics (e.g., publications, grants, awards, patents, etc.)

**Diversity and Inclusion Policy (2013, revised 2014):** Baylor College of Medicine fosters diversity among its students, trainees, faculty, and staff as a prerequisite to accomplishing our institutional mission and setting standards for excellence in training health care providers and biomedical scientists, promoting scientific innovation, and providing patient-centered care.

Diversity, respect, and inclusiveness create an environment that is conducive to academic excellence and strengthen our institution by increasing talent, encouraging creativity, and ensuring a broader perspective.

Diversity helps position Baylor to reduce disparities in health and health care access and to better address the needs of the community we serve.

Baylor is committed to recruiting and retaining outstanding students, trainees, faculty, and staff from diverse backgrounds by providing a welcoming, supportive learning environment for all members of the Baylor community.

**Baylor College of Medicine Wellness Director Job Description**

**Implementation Guidance:** This sample job description illustrates the application of job position requirements (EACMs) and job responsibilities and can serve as a model to help you write a clear job description.

The Wellness Director in the Office of the Provost will partner with key stakeholders to plan, strategize, leverage current resources, and implement new programs to promote wellness and to enhance resilience among trainees and faculty. The Wellness Director will coordinate assessments of well-being and track effectiveness of institutional interventions. This faculty position will also provide psychological services for trainees within the Student and House Staff Mental Health Service. The Wellness Director will report to the Associate Provost of Institutional Diversity, Inclusion, and Equity & Student Services.

- Experience in designing, leading, and promoting inclusion and wellness programs
- Demonstrated ability to lead through influence, have a bias towards action, and high emotional intelligence
- Established record of collecting and using data to assess the effectiveness of wellness initiatives and to achieve measurable results
- Direct patient care services provided include evaluation, short-term psychotherapy, crisis intervention, wellness coaching, and assistance with referrals to the community when warranted for trainee
- Serve as a resource for departments by providing information and educational wellness presentations/workshops
- Perform other job-related duties as assigned during work hours and must have availability for flexible scheduling of appointments and events

Required:

- Doctorate (PhD) in Psychology, Counseling, or related field
- Five years of directly related experience
- Experience in wellness, mental health and/or health sciences institution administration, policies, and procedures
- Understanding of governmental regulations, laws, programs, and agencies affecting trainee and faculty mental health, including licensure

**University of Massachusetts Medical School Job Posting Sites**

**Implementation Guidance:** Posting your positions to the following sites may assist you in recruiting a more diverse candidate pool. Posting to these sites is optional, may incur a cost, and at the discretion of your organization. When posting positions, consider including salary ranges to increase equity and equality of pay.^1^

**Job Posting Sites**

- Academic Careers. Accessed February 28, 2024. <https://academiccareers.com/>
- Academic Diversity Search. Accessed February 28, 2024. <https://www.academicdiversitysearch.com/>
- INSIGHT Into Diversity. Accessed February 28, 2024. <https://www.insightintodiversity.com/>
- National Medical Association. Accessed February 28, 2024. <http://www.nmanet.org/>
- Diverse Jobs. Accessed February 28, 2024. <https://diversejobs.net/>
- Equal Opportunity Publications Career Center. Accessed February 28, 2024. <https://www.eop.com/career.php>
- AAUW Careers. Accessed February 28, 2024. <https://www.aauw.org/about/careers/>
- Diversity Employers. Accessed February 28, 2024. <https://www.diversityemployers.com/>
- Association for Women in Science. Accessed February 28, 2024. <https://awis.org/>
- Society of Women Engineers. Accessed February 28, 2024. <https://swe.org/>
- Women In Higher Education. Accessed February 28, 2024. <https://employer.wihe.com/>
- National Society of Black Engineers. Accessed February 28, 2024. <https://www.nsbe.org/>
- Society for the Advancement of Chicanos and Native Americans in Science. Accessed February 28, 2024. <https://www.sacnas.org/>

**Publications**

- The Journal of Blacks in Higher Education. Accessed February 28, 2024. <https://jbhe.com/>
- Chronicle of Higher Education. Accessed February 28, 2024. <https://www.chronicle.com/>
- Hispanic Outlook on Education Magazine. Accessed February 28, 2024. <https://www.hispanicoutlook.com/>

**Reference:**

^1^Obloj, T., Zenger, T. The influence of pay transparency on (gender) inequity, inequality and the performance basis of pay. *Nat Hum Behav* 6, 646–655 (2022). https://doi.org/10.1038/s41562-022-01288-9
