# Supplementary material for: Implementing a Holistic Review Toolkit for Faculty Recruitment and Retention
Source: MedEdPORTAL. 2024 Dec 4;20:11472. doi: 10.15766/mep_2374-8265.11472 (PMC11615027; doi:10.15766/mep_2374-8265.11472)
Supplement: Supplementary file 1 — Faculty Pilot Overview.docxOverview Equity-Minded Hiring_Step 1.docxAssess Readiness for Equity-Minded Hiring_Step 1.docxStaff Composition Inventory_Step 2.xlsxHolistic Search Committee Phases and Steps_Step 2.docxFaculty Workshop Facilitators Guide_Step 3.docxFaculty Workshop Presentation_Step 3.pptxFaculty Workshop Evaluation_Step 3.docxFaculty Workshop Activities_Step 3.docxJob Description Posting Tools and Resources_Step 4.docxInterview Questions Tools and Resources_Step 4.docxSubmission Requirements and Rating Tools_Step 4.docx360-Degree (Multisource) Reference Checking_Step 4.docxSearch Process Tools and Resources_Step 5.docxStanding Up a Search Committee_Step 5.docxMitigating Bias Resources_Step 5.docxOnboarding Tools and Resources_Step 6.docxCareer Development Discussion Guide_Step 6.docxU Colorado SOM Mentoring Resource Packet_Step 6.docxBaylor College of Medicine Exit Resources_Step 6.docxU Colorado SOM Equitable Hiring Tool_Step 7.docxHolistic Hiring and Retention Tracker_Step 8.docxEvaluation Materials Development Phase_Steps 4-6.docx [file mep_2374-8265.11472-s001.zip › K. Interview Questions Tools and Resources_Step 4.docx]

Appendix K: Interview Questions Tools and Resources

**Baylor College of Medicine Holistic Interview Questions**

This document was created by Baylor’s Office of Institutional Diversity, Inclusion, and Equity, in collaboration with Human Resources, to assist search committees in hiring faculty and leadership positions within departments, academic centers, and schools. It includes content adapted from a behavioral interview guide by the Link Executive Search Firm.

**Implementation Guidance**

The following questions can be utilized for the recruitment of individuals across discipline and position (e.g., senior leadership, mid-career faculty, early career faculty, etc.). Use these sample questions, categorized by specific factors, to help you write relevant interview questions for the position you are seeking to fill.

**Interviewing: Questions to ask to identify experiences and attributes that are aligned with departmental and institutional mission(s).**

*Respect*

- How do you currently encourage people on your team to express their ideas and share a diversity of opinions? Can you give an example?
- Describe how you have helped to cultivate a respectful and inclusive learning and workplace environment in your current or previous jobs.
- How do you handle disagreement? Give me an example.
- What do you think creates and contributes to respect in the workplace?
- Tell me about an experience where you had to confront someone or give candid feedback. How did you do it? What did you say?

*Integrity*

- Tell me about a time when you were asked to do something that you didn’t think was right.
- How would you respond if you witnessed discrimination in the learning or workplace environment?
- Tell me what you do to ensure that you meet the work commitments you make to others.

*Innovation*

- What is one of the most imaginative or innovative projects you have developed in your present position?
- How have you leveraged talent from individuals who are from diverse backgrounds and/or multiple disciplines to achieve a goal or complete a project?
- Tell me about an innovation that you’ve introduced in your work area. What have you done to introduce change or redefine the way work gets done in your area?

*Teamwork*

- When working on a team project, have you ever had an experience where there was strong disagreement or interpersonal conflict among team members that you suspect was based upon the individuals’ backgrounds? How did you handle the situation?
- What are some of the things you are doing to ensure the effectiveness of the team you are leading?
- Have you been on a team that was ineffective? What did you do as a team member or team leader to address the problems?
- Tell me about a time where you disagreed with the objectives or direction of a team you were on. How did you handle this?

*(Note: Not all positions at Baylor College of Medicine work actively with teams. Hiring managers should consider what working with a team means in their department—if the employee mainly works on projects alone, how is teamwork fostered? Is collaboration encouraged? If so, discuss this as well.)*

*Excellence*

- In your last job, what problems did you identify that had previously been overlooked? Were changes made? Who supported the changes as a result of your ideas?
- As a leader, how have you promoted excellence and equity in your team?
- Give me an example of an important goal that you had set in the past and tell me about your success in reaching it.

*Good introductory questions*

1. How did you hear about this position?
2. What motivated you to apply for this position?
3. Why did you decide to seek a position in this field?
4. Tell me what you know about our institution.
5. Why did you decide to seek a position in this institution?

*Questions for recent graduates/those at the beginning of careers*

1. How has your college/training experience prepared you for a career in __________?
2. Please describe the ideal job for you following graduation.
3. What influenced you to choose this career?
4. At what point did you choose this career?
5. If you could do so, how would you plan your college career differently?
6. Are you more energized by working with data or by collaborating with other individuals?
7. What were your reasons for selecting your college or university?
8. If you could change or improve anything about your college, what would it be?
9. How will the academic program and coursework you’ve taken benefit your career and/or this position?
10. Which college classes or subjects did you like best (or least)? Why?
11. Are you the type of student for whom conducting independent research has been a positive experience? Why or why not?
12. Describe the type of professor that has created the most beneficial learning experience for you. Do you think that your grades are an indication of your academic achievement? Why or why not?
13. What plans do you have for continued study? An advanced degree?
14. Describe a situation in which you had to use reference materials to write a research paper. What was the topic? What journals did you read? Describe your research method.
15. How was your transition from high school to college? Did you face any particular problems?
16. What are your standards of success in school? What have you done to meet these standards?
17. How have you differed from your professors in evaluating your performance? How did you handle the situation?
18. Why did you select your college or university?
19. What led you to choose your major or field of study?
20. What have you learned from participation in extracurricular activities?

*Goals*

1. What specific goals, including those related to your occupation, have you established for your life?
2. What specific goals have you established for your career? How do you plan to achieve them?
3. What will it take to attain your goals, and what steps have you taken toward attaining them?
4. Would you describe yourself as goal-driven? Please describe a recent goal that you set and how you achieved it or are working toward achieving it.
5. Describe what you’ve accomplished toward reaching a recent goal for yourself.
6. What short-term goals and objectives have you established for yourself?
7. Can you describe your long-range goals and objectives? What do you expect to be doing in five years? Ten years?
8. Give me an example of an important goal that you had set in the past and tell me about your success in reaching it.

*Team-orientation/tact and diplomacy/cooperating with others to achieve a common goal/professional presence*

1. How would you describe yourself in terms of your ability to work as a member of a team? Describe the types of teams you’ve been involved with. What were your roles?
2. Describe a team experience you found rewarding.
3. Describe a team experience you found disappointing. What would you have done to prevent this?
4. Can you tell me about an occasion where you needed to work with a group to get a job done?
5. Describe when you or a group that you were a part of were in danger of missing a deadline. What did you do?
6. Tell me about a time when you worked with a person who did things very differently from you. How did you get the job done?
7. Describe a time when you got coworkers or classmates who disliked each other to work together. How did you accomplish this? What was the outcome?

*Motivation/perseverance/accomplishments/success*

1. What motivates you to put forth your greatest effort?
2. Give an example of a time when you went above and beyond the call of duty.
3. Tell me about an important goal that you set in the past. Were you successful? Why?
4. Describe a situation when you were able to have a positive influence on the actions of others.
5. How would you define “success” for someone in your chosen career?
6. Give examples of your experiences at school or in a job that were satisfying. Give examples of your experiences that were dissatisfying.
7. How have you motivated yourself to complete an assignment or task that you did not want to do?
8. Describe a time you felt you were the most resourceful in solving a problem or coming up with an improvement.
9. Tell me about the last time you went “the extra mile” to do something because it needed to be done, even though it wasn’t your responsibility.
10. Tell me about some times when you weren’t very pleased with your performance. What did you do about it? (Work Standards)
11. All jobs have their frustrations and problems. Describe some examples of specific job conditions, tasks, or assignments that have been dissatisfying to you. Exactly why were they dissatisfying?
12. Give an example of when your persistence had the biggest payoff.
13. What do you think it takes to be successful in this career?
14. How do you determine or evaluate success? Give me an example of one of your successful accomplishments.
15. Do you have the qualifications and personal characteristics necessary for success in your chosen career?
16. What has been your most rewarding accomplishment?
17. What quality or attribute do you feel will most contribute to your career success?
18. What challenges to your success do you expect to encounter in this position?

*Initiative*

1. Give me an example of when you had to go above and beyond the call of duty in order to get a job done.
2. Give me examples of projects/tasks you started on your own.
3. Give some instances in which you anticipated problems and were able to influence a new direction.
4. How have you demonstrated initiative?
5. Have you generated any new ideas or suggestions while at school or at work?
6. Give me a specific example of a time when you sold your supervisor or professor on an idea or concept. How did you proceed? What was the result?

*Conflict resolution*

1. How would you evaluate your ability to deal with conflict?
2. Have you ever had difficulty with a supervisor or instructor? How did you resolve the conflict?
3. Tell me about a major problem you recently handled. Were you successful in resolving it?
4. Would you say that you can easily deal with high-pressure situations?
5. What personal weakness has caused you the greatest difficulty in school or on the job?
6. Tell of a time when you worked with a colleague who was not completing his or her share of the work. Who, if anyone, did you tell or talk to about it? Did the manager take any steps to correct your colleague? Did you agree or disagree with the manager’s actions?

*Decision-making*

1. Give an example of a time in which you had to be relatively quick in coming to a decision.
2. What steps do you follow to study a problem before making a decision?
3. Give me an example of a time you had to make an important decision. How did you make the decision? How does it affect you today?
4. What was your most difficult decision in the last 6 months? What made it difficult?
5. What kind of decisions do you make rapidly? What kind takes more time? Give examples.
6. Tell me about a time when you had to make a decision but didn’t have all the information you needed.
7. Tell me about the riskiest decision that you have made.

*Problem-solving/learning ability/problem analysis and judgment*

1. Give me a specific example of a time when you used good judgment and logic in solving a problem.
2. We can sometimes identify a small problem and fix it before it becomes a major problem. Give an example(s) of how you have done this.
3. Describe a situation in which you effectively developed a solution to a problem by combining different perspectives or approaches.
4. Describe an instance when you had to think on your feet to extricate yourself from a difficult situation.
5. Describe a time when you were faced with problems or stresses that tested your coping skills.
6. Describe a specific problem you solved for your employer or professor. How did you approach the problem? What role did others play? What was the outcome?
7. Describe the system you use for keeping track of multiple projects. How do you track your progress so that you can meet deadlines? How do you stay focused?
8. Tell me about a time when you came up with an innovative solution to a challenge your company/class/organization was facing. What was the challenge? What role did others play?
9. Can you tell me about a complex problem that you solved? Describe the process you utilized.
10. Think about a complex project or assignment that you have been assigned. What approach did you take to complete it?
11. Tell me about a challenge that you successfully met.
12. Walk me through a situation where you had to do research and analyze the results for one of your classes.
13. Describe the last situation in which you were required to “think on your feet.” What did you decide to do? How satisfied were you with the results? What would you change if you could?
14. Tell me about the last time you made a decision or solved a problem that required a lot of hard thinking and careful analysis on your part.
15. Can you think of a situation you had to handle in which old solutions didn’t work? What did you do to handle it?
16. Describe the last problem you were involved with that was solved in a highly imaginative manner. What part did you play?

*Communication*

1. Discuss how you have effectively communicated across diverse groups and disciplines.
2. Tell me about a situation when you had to speak up (be assertive) in order to get a point across that was important to you.
3. Describe the most significant written document, report, or presentation that you had to complete.
4. Have you had to “sell” an idea to your coworkers, classmates, or group? How did you do it? Did they “buy” it?
5. Describe a situation in which you were able to use persuasion to successfully convince someone to see things your way.
6. Describe a time when you had to use your written communication skills to get an important point across.
7. Describe the most significant or creative presentation that you have had to complete.
8. What has been your experience in giving presentations? What has been your most successful experience in speechmaking?
9. Give me an example of a time when you were able to successfully communicate with another person even when that individual may not have personally liked you (or vice versa).
10. Tell of a time when your active listening skills really paid off for you—maybe a time when other people missed the key idea being expressed.

*Interpersonal skills*

1. Give an example of when you had to work with someone who was difficult to get along with. Why was this person difficult? How did you handle that person?
2. Describe a situation where you found yourself dealing with someone who didn’t like you. How did you handle it?
3. By providing examples, convince me that you can adapt to a wide variety of people, situations, and environments.
4. Describe a situation in which you had to arrive at a compromise or guide others to a compromise.
5. Describe a situation where others you were working with on a project disagreed with your ideas. What did you do?
6. Describe a situation in which you were able to effectively “read” another person and guide your actions by your understanding of their needs and values.
7. What have you done in past situations to contribute toward a teamwork environment?
8. Describe a recent unpopular decision you made and what the result was.
9. Tell me about the most difficult or frustrating individual that you’ve ever had to work with and how you managed to work with them.
10. Tell me about a time when you had to deal with a difficult person. How did you handle the situation?
11. Tell me about the last time you went out of your way to get to know someone. What was the situation? What motivated you to get to know this person? What specifically did you do? How did this person respond?
12. Give an example of dealing with a person when you pushed too hard. Why? How did you determine when you should back off? Give an example.

*Time management/organization/prioritizing/deadlines*

1. How do you determine priorities in scheduling your time? Give examples.
2. Describe a situation that required a number of things to be done at the same time. How did you handle it? What was the result?
3. Have you found any ways to make school or a job easier or more rewarding or to make yourself more effective?
4. Tell me about a time you had to handle multiple responsibilities. How did you organize the work you needed to do?
5. Describe a situation where class assignments and work or personal activities conflicted. How did you prioritize? How did you manage your time? What was the outcome?
6. Tell me about a time when you failed to meet a deadline. What things did you fail to do? What were the repercussions? What did you learn?
7. What deadlines are you working toward currently that you have imposed on yourself? Tell me how you set that deadline and planned to meet it. How far ahead or behind schedule are you?
8. Tell me about the time when your workload was most hectic. What competing demands did you have to deal with? What did you do to handle the situation? How did you schedule your time? How did it turn out?
9. What has been your biggest challenge in making effective use of your time? Give me an example of a time when that was a problem for you. What have you done to deal with that problem?
10. Tell me about a time when you decided to say “No” when you were asked to help with a project. Why did you decide to say “No”? How did you handle the situation?
11. How do you decide what gets top priority when scheduling your time?
12. What do you do when your schedule is suddenly interrupted? Give an example.
13. What have you done in order to be effective with your organization and planning?
14. How do you schedule your time? Set priorities? How do you handle doing twenty things at once?
15. What do you do when your time schedule or project plan is upset by unforeseen circumstances? Give an example.
16. Tell me about a time when you got caught up in the details of the project.

*Leadership abilities/management style—delegating to, motivating, and influencing others/coaching others*

1. What leadership positions have you held? Describe your leadership style.
2. Have you ever worked in a position (or on a school project or a recreational/sports event) where you acted as a team leader? How did you organize the other people’s activities? How did you work with people who did not do what you wanted them to do (give me an example)?
3. Describe the situation that best demonstrates your ability to get things done through others from diverse backgrounds.
4. Describe the most significant situation in which you demonstrated leadership by getting others to believe in a goal and energizing them to achieve it.
5. Tell me about the time you met the most resistance when proposing a plan of action. How did you handle the situation?
6. How would you describe your leadership and/or management style?
7. How would others describe your leadership and/or management style? Why do you feel this is accurate/inaccurate?
8. In a supervisory or group leader role, have you ever had to discipline or counsel an employee or group member? What was the nature of the discipline? What steps did you take? How did that make you feel? How did you prepare yourself?
9. Give an example of your ability to build motivation in your coworkers, classmates, and even if on a volunteer committee.
10. What is the toughest group that you have had to get cooperation from? Describe how you handled it. What was the outcome?
11. Have you ever been a member of a group where two of the members did not work well together? What did you do to get them to do so?
12. Give me a specific example of something you did that helped build enthusiasm in others.
13. Tell me about a time when you were able to provide a coworker with recognition for the work they performed.
14. What steps do you take to ensure that the work you delegate is successful?

*Strategic thinking*

1. Tell us about a specific decision that you made within your organization that had unexpected consequences outside your organization. How did you deal with those consequences?
2. Describe a change you are responsible for that improved the performance of your work area or organization. How did: 1) you come up with the idea for the change, 2) you go about implementing the change, 3) staff respond to the change, and 4) you measure the outcome of the change? Looking back, what things would you do differently?

*Customer service*

1. Tell of the most difficult customer service experience that you have ever had to handle—perhaps an angry or irate customer. Be specific and tell what you did and what the outcome was.
2. Tell me about a time when you had to go above and beyond the call of duty in order to get a job done.

*Project management/projects*

1. Recall a time when you were assigned what you considered to be a complex project. Specifically, what steps did you take to prepare for and finish the project? Were you happy with the outcome? What one step would you have done differently if given the chance?
2. What was the most complex assignment you have had? What was your role?
3. Describe some projects or ideas (not necessarily your own) that were implemented or carried out successfully primarily because of your efforts.
4. Describe how you develop a project team’s goals and project plan.

*Performance*

1. Compare and contrast the times when you did work that was above the standard with times your work was below the standard.
2. Describe a time when you were not very satisfied or pleased with your performance. What did you do about it?
3. What is the most significant contribution you made to the company during a past job or internship?
4. What is the biggest mistake you’ve made?
5. Describe your three greatest accomplishments to date.
6. Tell me about a time when you did not live up to your full potential.
7. Describe for me a time when you failed at something and how you responded.
8. Tell me about a time when you missed an obvious solution to a problem.

*Adjustment—flexibility, stress management/integrity/maturity and self-awareness/dealing with criticism/dealing with change*

1. Tell me about a situation when you had to learn something new in a short time. How did you proceed? Describe a situation that you initially thought you could easily handle alone and soon realized you were over your head. What did you do?
2. All of us become “edgy” at times. Tell me about a time you felt that way. What did you do about it?
3. What were some of your biggest disappointments at a job/school/organization? How did you cope with them?
4. Give me a specific occasion when you conformed to a policy with which you did not agree.
5. Sometimes it’s easy to get in “over your head.” Describe a situation where you had to request help or assistance on a project or assignment.
6. Recall a time from your work experience when your manager or supervisor was unavailable and a problem arose. What was the nature of the problem? How did you handle that situation? How did that make you feel?
7. Tell me of some situations in which you have had to adjust quickly to changes over which you had no control. What was the impact of the change on you?
8. Give me a specific example of a time when a coworker or classmate criticized your work in front of others. How did you respond? How has that event shaped the way you communicate with others?
9. Describe a time when you put your needs aside to help a coworker or classmate understand a task. How did you assist him or her? What was the result?
10. How have you most constructively dealt with disappointment and turned it into a learning experience?
11. Give an example of how you applied knowledge from previous coursework to a project in another class.
12. Describe a situation in which you found that your results were not up to your professor’s or supervisor’s expectations. What happened? What action did you take?
13. Tell me about a time when you had to adjust to changes over which you had no control.
14. Tell me about a time when you had to adjust to a classmate or coworker’s working style in order to complete a project or achieve your objectives.

*Integrity/honesty*

1. Tell me about a time when you experienced a loss for doing what was right.
2. Give me a specific example of a policy you conformed to with which you did not agree.
3. Tell me about a specific time when you had to handle a tough problem that challenged fairness or ethical issues.
4. Tell me about a time when you challenged the status quo to do what you felt was right.

*Technical skills/knowledge*

1. In what areas do you have special expertise?
2. How did you develop that expertise? How have you utilized that expertise?
3. What do you keep up to date in your field?
4. Give me some examples of the most complex (assignments, projects, etc.) you had. What was your role? How did it work out?

*Other*

1. Describe the characteristics of a successful manager.
2. What kind of supervisor do you work best for? Provide examples.
3. What suggestions do you have for our organization?
4. Before you can make a productive contribution to the company, what degree of training do you feel you will require?
5. Given the investment our company will make in hiring and training you, can you give us a reason to hire you?

*Relocation-related*

1. Do you have a geographic preference?
2. Why do you think you might like to live in the community in which our company is located?
3. Would it be a problem for you to relocate?
4. To what extent would you be willing to travel for the job?

*Compensation-related*

1. Which is more important to you, the job itself or your salary? Why?
2. What level of compensation would it take to make you happy?
3. Tell me about the salary range you’re seeking.

*Final questions—concluding the interview*

1. Is there anything that we might have left out that you would like us to clarify?
2. What else should we know about you?
3. Do you have any questions you would like to ask us?

*Probing questions—can be used with any of the questions above to solicit more detail*

- Why?
- Where?
- When?
- How?
- What?
- How did you prepare for that?
- What was your reasoning?
- Who else was involved?
- What was your role?
- What obstacles did you face?
- What were you thinking at that point?
- Lead me through your decision process.
- Tell me more about your interaction with that person.
- Where were you when this happened?
- Who do you think was at fault?
- What was your logic?
- Why did you decide to do that?
- How did you resolve that?
- What did you learn from that?
- What was the outcome of that?
- How do you feel about that?
- What was your reaction?
- How did you handle that?
- What happened after that?
- How did he/she react?
- What did you say?
- Were you happy with that outcome/result?
- What did you wish you had done differently?

**Interview Questions to Assess Background in Diversity, Equity, and Inclusion**

**Implementation Guidance:** Before incorporating questions from this list, your institution should review federal and local laws to ensure they align with organizational policies and procedures. Use the following sample questions to help you write relevant interview questions for the position you are seeking to fill.

1. Please tell us about a time when you gave feedback to a colleague who was not accepting of another person, based on some aspect of that person’s identity, such as race or ethnicity. What factors did you consider in approaching them, and how did you do it?
2. Please tell us about a time when you realized you had said or done something that may have been offensive to a colleague, given their cultural identity? How did you respond to the realization, and what was the outcome?
3. Describe the climate for DEI at your present institution. What impact have you had on that climate, and how have you created that impact?
4. What measures have you taken to make someone from a background that is underrepresented in medicine feel comfortable, especially if others seem uncomfortable with their presence?
5. Please tell us about how you have improved the learning environment to better meet the needs of learners, staff, or faculty from marginalized backgrounds (such as Native Americans, African Americans, Latinos, Asians, and other communities).
6. Describe your experience or explain how you have been educated to understand the history of African Americans, Latinos, Asians, Native Americans, and other historically marginalized communities in America.
7. In previous work experiences, what has been the greatest obstacle in developing culturally humble and competent staff or learners? How have you worked to overcome these obstacles?
8. Suppose that in working with a university unit you discover a pervasive belief that diversity and excellence are somehow in conflict. How do you conceptualize the relationship between diversity and excellence with respect to patient care, teaching, and scholarship? What kinds of leadership efforts are needed to encourage a commitment to excellence through diversity?
9. What programs or initiatives have you developed in the area of DEI or anti-racism?
10. Has diversity played a role in shaping your teaching, advising, patient care, and/or leadership styles?
11. What have you done to further your knowledge/understanding about DEI? How have you demonstrated or applied your learning?
12. If you were to be offered this position, how would you use it to advance DEI and/or anti-racism in your new role?
13. For clinical positions: How do you demonstrate cultural humility when working with patients?
14. For clinical positions: Please give an example of an encounter with a culturally different patient that went well and one that didn’t go so well. What did you learn from these experiences, and how have you applied those lessons to subsequent patient interactions?
15. For research positions: How have you advanced DEI in your scholarship? How would you continue to advance DEI in the position for which you are applying?
16. For teaching positions: How have you incorporated anti-racism in your teaching and curricula? How would you continue to do so in this position?
17. How would you respond if a student made a comment in a lecture setting that you interpreted as a microaggression?

**Employment Interviews FAQs**

**Implementation Guidance: f**ederal, state, and local laws provide protections based on certain characteristics. Depending on your locality, some or all the questions below may pose legal risk to your organization. Check with your legal counsel on which laws apply to your organization.

So long as you do not use the information for a discriminatory purpose, it’s **OKAY** to ask all applicants whether they are able to perform the job for which they are interviewing. For example:

- Are you authorized to work in the United States?
- Are you able to perform the essential functions of this position with or without reasonable accommodations?
- Will you be able to meet the licensing requirements for this position?

As a general matter, it is a best practice to **AVOID** questions about:

| RACE | 🗷 “What is your race, color, or ethnicity?”  🗷 “Where are you/your parents from?” |
| --- | --- |
| RELIGION | 🗷 “What is your religion?”  🗷 “Do you need breaks to pray?”  🗷 “Do you need time off for religious holidays?” |
| SEX | 🗷 “Are you male or female?”  🗷 “What is your sexual orientation?”*  🗷 “What is your gender identity?”* |
| AGE | 🗷 “How old are you?”  🗷 “What’s your date of birth?” |
| CITIZENSHIP or NATIONAL ORIGIN | 🗷 “Are you a U.S. citizen?”  🗷 “What is your citizenship status?”  🗷 “What is your country of origin?” |
| DISABILITY | 🗷 “Are you disabled?”  🗷 “Do you use drugs or alcohol?”  🗷 “Do you have any health problems?”  🗷 “How serious are your disabilities/health problems?” |
| CRIMINAL BACKGROUND* | 🗷 “Have you ever been arrested?”  🗷 “Have you ever been convicted of a crime?” |

(*Protections vary by location.)
