# Supplementary material for: Implementing a Holistic Review Toolkit for Faculty Recruitment and Retention
Source: MedEdPORTAL. 2024 Dec 4;20:11472. doi: 10.15766/mep_2374-8265.11472 (PMC11615027; doi:10.15766/mep_2374-8265.11472)
Supplement: Supplementary file 1 — Faculty Pilot Overview.docxOverview Equity-Minded Hiring_Step 1.docxAssess Readiness for Equity-Minded Hiring_Step 1.docxStaff Composition Inventory_Step 2.xlsxHolistic Search Committee Phases and Steps_Step 2.docxFaculty Workshop Facilitators Guide_Step 3.docxFaculty Workshop Presentation_Step 3.pptxFaculty Workshop Evaluation_Step 3.docxFaculty Workshop Activities_Step 3.docxJob Description Posting Tools and Resources_Step 4.docxInterview Questions Tools and Resources_Step 4.docxSubmission Requirements and Rating Tools_Step 4.docx360-Degree (Multisource) Reference Checking_Step 4.docxSearch Process Tools and Resources_Step 5.docxStanding Up a Search Committee_Step 5.docxMitigating Bias Resources_Step 5.docxOnboarding Tools and Resources_Step 6.docxCareer Development Discussion Guide_Step 6.docxU Colorado SOM Mentoring Resource Packet_Step 6.docxBaylor College of Medicine Exit Resources_Step 6.docxU Colorado SOM Equitable Hiring Tool_Step 7.docxHolistic Hiring and Retention Tracker_Step 8.docxEvaluation Materials Development Phase_Steps 4-6.docx [file mep_2374-8265.11472-s001.zip › L. Submission Requirements and Rating Tools_Step 4.docx]

# Appendix L: Submission Requirements and Interview Rating Tools

### Baylor College of Medicine Applicant Submission Requirements and Evaluation Tool

**Implementation Guidance:** Before implementing the recommendations in this document, your institution should review federal and local laws to ensure they align with organizational policies and procedures. The following submission requirements and evaluation tools have been provided as examples and may be modified as necessary for your own needs.

### Submission Requirements

## Online Application System

- Decide if you want to collect statements that support your identified experiences, attributes, and competencies (from applicants or references).

## Reference Letter or Screen Components

- Determine what specific experiences, attributes, and competencies you would like reference letters to address or screeners to ask references about.

## Applicant Questions

Describe the experiences, attributes, and competencies you have that will help us achieve our mission and that make you uniquely suited to be successful in this position.

Describe how you have contributed and could contribute to our inclusive environment.

### Holistic Applicant Evaluation Tool

**Implementation Guidance:** The following offers a method for department faculty to provide evaluations of job applicants. It is meant to be a template for departments that they can modify as necessary for their own uses. The proposed questions are designed for junior faculty candidates; however, alternate language is suggested in parenthesis for senior faculty candidates.

Applicant’s name:

Please indicate which of the following are true for you (check all that apply):

| □ | Read applicant’s letters of recommendation |
| --- | --- |
| □ | Read applicant’s CV for evidence of alignment with our four mission areas (education, research, health care, and service) |
| □ | Read statement for evidence of alignment with Baylor College of Medicine (BCM) values/mission (respect, integrity, innovation, teamwork, excellence) |
| □ | Read applicant’s application for alignment of their experiences, attributes, and metrics with the position description |

| Please rate the applicant on each of the following: | Excellent | Good | Neutral | Fair | Poor | Unable to Evaluate |
| --- | --- | --- | --- | --- | --- | --- |
| Evidence of identified experiences relevant to the position and mission area(s) |  |  |  |  |  |  |
| Evidence of educational contributions and productivity |  |  |  |  |  |  |
| Evidence of research productivity |  |  |  |  |  |  |
| Evidence of quality in health care |  |  |  |  |  |  |
| Evidence of participation in service (local/regional/national/global) |  |  |  |  |  |  |
| Evidence of attributes relevant to the position and mission area(s) |  |  |  |  |  |  |
| Evidence of alignment with BCM values (respect, integrity, innovation, teamwork, excellence) |  |  |  |  |  |  |
| Evidence of identified metrics relevant to the position and mission area(s) |  |  |  |  |  |  |
| Potential to contribute to the institutional mission/diversity goals* |  |  |  |  |  |  |
| Potential for scholarly impact (tenure/non-tenure) |  |  |  |  |  |  |

Additional Comments:

Adapted from UC Irvine’s National Science Foundation ADVANCE PROGRAM

UC Irvine ADVANCE Program. Accessed March 18, 2018. <http://advance.uci.edu>.


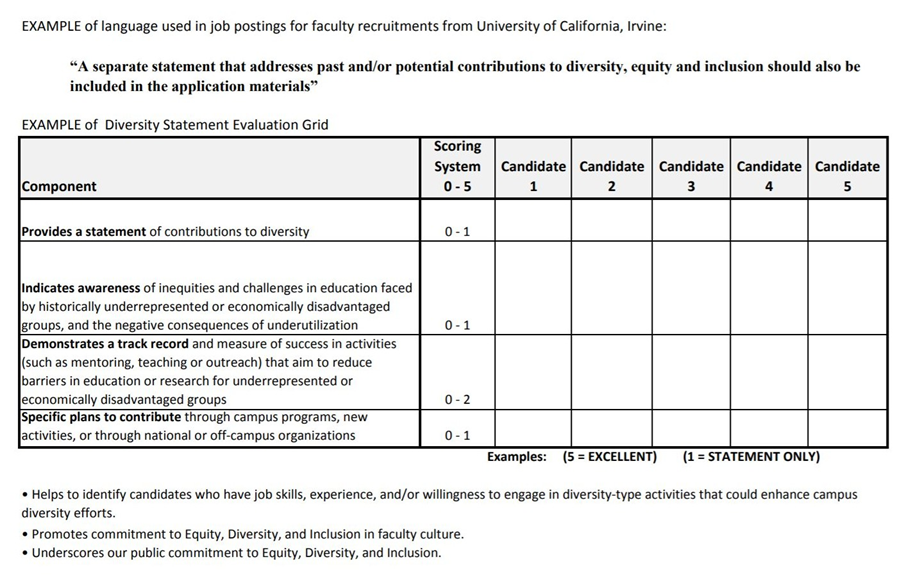


Diversity Statement Evaluation Grid adapted from University of California, Irvine. *Evaluations of Faculty Job Candidates’ Diversity Statement Evaluation* and Columbia University *Evaluating Faculty Candidates Diversity, Equity, and Inclusion Statements*.

*UCI Faculty Job Candidates’ Diversity Statement Evaluation.* Office of Faculty Development & Diversity, UCI School of Social Sciences. Accessed April 15, 2024. <https://www.equity.socsci.uci.edu/files/docs/faculty-diversity-statements.pdf>

*Evaluating Faculty Candidates Diversity, Equity, and Inclusion Statements*. Columbia University Equal Opportunity and Affirmative Action. Accessed April 15, 2024 <https://eoaa.columbia.edu/sites/default/files/content/docs/Rubric_to_Assess_Faculty_Candidate_Contributions_to_D_E_I.pdf>

### Sample Form: Baylor College of Medicine Wellness Director Candidate Rating Form

**Implementation Guidance:** The following sample form is meant to serve as an example illustrating how Baylor College of Medicine integrated their priority competencies, experiences, attributes, and metrics into a rating form for a specific position.

| **Candidate Name:** | | | | |
| --- | --- | --- | --- | --- |
| **Interview Date:** | | | | |
| **Interviewer Name:** | | | | |
| ***To what degree has the candidate demonstrated the capability?*** ***(1 - not at all/very little; 2 - moderate; 3 - completely demonstrated)*** | | | | |
| **Desired Competency, Experience, Attribute, or Metric** | **1** | **2** | **3** | **N/A** |
| **Employment Experiences:**   \| - Experience in designing, leading, and promoting inclusion and wellness programs \|  \|  \|  \|  \| \| --- \| --- \| --- \| --- \| --- \| \| - Established record of collecting and using data to assess the effectiveness of wellness initiatives and to achieve measurable results \|  \|  \|  \|  \| \| - Leading institutional and/or departmental wellness or well-being committees \|  \|  \|  \|  \| \| - Works to ensure appropriate record keeping (e.g., meeting minutes) and information sharing procedures (e.g., in accordance with HIPAA and FERPA). \|  \|  \|  \|  \| \| - Establishes meeting agendas and facilitates meeting discussion(s). \|  \|  \|  \|  \| \| - Direct patient care services provided include evaluation, short-term psychotherapy, crisis intervention, and wellness coaching \|  \|  \|  \|  \| \| - Assists with community referrals for health and wellness services \|  \|  \|  \|  \| \| - Provides information regarding wellness and mental health resources for trainees, faculty, and staff \|  \|  \|  \|  \| \| - Experience in wellness, mental health, and/or health sciences institution administration, policies, and procedures \|  \|  \|  \|  \| \| - Understanding of governmental regulations, laws, programs, and agencies affecting trainee and faculty mental health, including licensure \|  \|  \|  \|  \| | | | | |

### Sample Form: Baylor College of Medicine Wellness Director Candidate Rating Form (continued)

| **Attributes:** | **1** | **2** | **3** | **N/A** |
| --- | --- | --- | --- | --- |
| \| - Embodies the BCM value Respect (diversity, inclusion, equity) \|  \|  \|  \|  \| \| --- \| --- \| --- \| --- \| --- \| \| - Exemplifies the BCM value Integrity (honesty, ethics, professionalism) \|  \|  \|  \|  \| \| - Illustrates the BCM value Innovation (imagination, creativity) \|  \|  \|  \|  \| \| - Personifies the BCM value Teamwork \|  \|  \|  \|  \| \| - Embodies the BCM value Excellence (quality, continuous quality improvement) \|  \|  \|  \|  \| \| - Demonstrates a commitment to diversity and inclusiveness \|  \|  \|  \|  \| \| - Displays outstanding interpersonal skills \|  \|  \|  \|  \| \| - Commitment to service \|  \|  \|  \|  \| | | | | |
| \| **Metric(s)/Qualifications:** \| **1** \| **2** \| **3** \| **N/A** \| \| --- \| --- \| --- \| --- \| --- \| \| - Doctorate (PhD) in Psychology, Counseling, or related field \|  \|  \|  \|  \| \| \| - Five years of directly related experience \|  \|  \|  \|  \| \| \| - Licensure in a counseling or related field by the Texas State Board of Examiners \|  \|  \|  \|  \| \| | | | | |
| **Recommendation:**  **As an overall assessment of the suitability of this candidate for this position, I would (please check one option):**  **____ strongly recommend this individual to be considered for the Wellness Director role.**  **____ recommend this individual to be considered for the Wellness Director role.**  **____ recommend with reservations that this individual be considered for the Wellness Director role.**  **____ not recommend this individual to be further considered for the Wellness Director role.**  **Comments:** | | | | |
