# Supplementary material for: Implementing a Holistic Review Toolkit for Faculty Recruitment and Retention
Source: MedEdPORTAL. 2024 Dec 4;20:11472. doi: 10.15766/mep_2374-8265.11472 (PMC11615027; doi:10.15766/mep_2374-8265.11472)
Supplement: Supplementary file 1 — Faculty Pilot Overview.docxOverview Equity-Minded Hiring_Step 1.docxAssess Readiness for Equity-Minded Hiring_Step 1.docxStaff Composition Inventory_Step 2.xlsxHolistic Search Committee Phases and Steps_Step 2.docxFaculty Workshop Facilitators Guide_Step 3.docxFaculty Workshop Presentation_Step 3.pptxFaculty Workshop Evaluation_Step 3.docxFaculty Workshop Activities_Step 3.docxJob Description Posting Tools and Resources_Step 4.docxInterview Questions Tools and Resources_Step 4.docxSubmission Requirements and Rating Tools_Step 4.docx360-Degree (Multisource) Reference Checking_Step 4.docxSearch Process Tools and Resources_Step 5.docxStanding Up a Search Committee_Step 5.docxMitigating Bias Resources_Step 5.docxOnboarding Tools and Resources_Step 6.docxCareer Development Discussion Guide_Step 6.docxU Colorado SOM Mentoring Resource Packet_Step 6.docxBaylor College of Medicine Exit Resources_Step 6.docxU Colorado SOM Equitable Hiring Tool_Step 7.docxHolistic Hiring and Retention Tracker_Step 8.docxEvaluation Materials Development Phase_Steps 4-6.docx [file mep_2374-8265.11472-s001.zip › M. 360-Degree (Multisource) Reference Checking_Step 4.docx]

# Appendix M: 360-Degree (Multisource) Reference Checking

Implementation Guidance: A 360-degree (multisource) reference check helps form a more objective picture of the possible candidate. Use the best practices outlined below for your reference checks.

Originally published in Mallon WT, Grigsby, RK. *Recruiting: Proven Search and Hiring Practices for the Best Talent*. Association of American Medical Colleges; 2017. Additional resources and information can be found on AAMC’s Hiring the Best Talent Web site.^1^

Best Practices

- **Assess behavioral competencies that are critical to work success.** The following questions, adapted from Bennis and O’Toole,^2^ may be useful.

| - Can you provide an example of how the candidate inspired followers to trust them? - In what ways does the candidate energize others? Can you provide an example? - Can you tell me how you have witnessed the candidate developing others? - In what ways does the candidate demonstrate respect for followers? Have you witnessed situations where they did not? - In what ways does the candidate demonstrate that they listen? - How does the candidate hold people accountable for their performance and promises? - Have you seen the candidate delegating important tasks to others? How so? |
| --- |

- **Strive for consistency.** Reference calls should be made by one person, either the dean or the search committee chair, so that each reviewer is asked the same questions. Calls by search committee members other than the chair should not be allowed.
- **Gather multiple perspectives on the candidate.** Call the references the candidate has provided and go off list by calling peers, collaborators, colleagues, and, most importantly, workers who report to the candidate, such as administrative assistants, nurses, students, and residents.

Offer the candidate the opportunity to identify individuals whom they believe would be biased against them. Honoring requests not to contact these people is recommended in the spirit of open communication with the candidate.

- **Document your process and results.** Keep a log of individuals you contacted for references, those with whom you spoke, and notes on the conversations. This documentation is not shared with anyone outside the search committee.

## References:

1. Hiring the Best Talent. Association of American Medical Colleges. Accessed February 27, 2024. <https://www.aamc.org/career-development/leadership-development/recruiting>
2. Bennis WG, O’Toole J. Don’t hire the wrong CEO. *Harv Bus Rev*. 2000;78(3):170-176,218.
