# Supplementary material for: Implementing a Holistic Review Toolkit for Faculty Recruitment and Retention
Source: MedEdPORTAL. 2024 Dec 4;20:11472. doi: 10.15766/mep_2374-8265.11472 (PMC11615027; doi:10.15766/mep_2374-8265.11472)
Supplement: Supplementary file 1 — Faculty Pilot Overview.docxOverview Equity-Minded Hiring_Step 1.docxAssess Readiness for Equity-Minded Hiring_Step 1.docxStaff Composition Inventory_Step 2.xlsxHolistic Search Committee Phases and Steps_Step 2.docxFaculty Workshop Facilitators Guide_Step 3.docxFaculty Workshop Presentation_Step 3.pptxFaculty Workshop Evaluation_Step 3.docxFaculty Workshop Activities_Step 3.docxJob Description Posting Tools and Resources_Step 4.docxInterview Questions Tools and Resources_Step 4.docxSubmission Requirements and Rating Tools_Step 4.docx360-Degree (Multisource) Reference Checking_Step 4.docxSearch Process Tools and Resources_Step 5.docxStanding Up a Search Committee_Step 5.docxMitigating Bias Resources_Step 5.docxOnboarding Tools and Resources_Step 6.docxCareer Development Discussion Guide_Step 6.docxU Colorado SOM Mentoring Resource Packet_Step 6.docxBaylor College of Medicine Exit Resources_Step 6.docxU Colorado SOM Equitable Hiring Tool_Step 7.docxHolistic Hiring and Retention Tracker_Step 8.docxEvaluation Materials Development Phase_Steps 4-6.docx [file mep_2374-8265.11472-s001.zip › N. Search Process Tools and Resources_Step 5.docx]

# Appendix N: Search Process Tools and Resources

### The 10 Characteristics of a Good Search

Implementation Guidance: Use this checklist to ensure that your organization has attended to all 10 components of a good search.

Originally published in Mallon WT, Grigsby, RK. *Recruiting: Proven Search and Hiring Practices for the Best Talent.* Association of American Medical Colleges; 2017. Additional resources and information can be found on AAMC’s Hiring the Best Talent Web site.^1^

| ✔ | Search Characteristic |
| --- | --- |
| ☐ | Continuity: Has your institution adopted a systemwide approach to searches? Here are some points to consider:   - Are searches run with a common framework and approach? - Are all searches staffed in the same manner? - Is there an institutional point person or team that ensures consistency and quality control in the mechanics of the search? |
| ☐ | Communication: Are mechanisms in place to ensure open and timely communication with everyone involved in the search? Consider communication between:   - Dean or CEO and the search committee - Dean or CEO and staff - Search chair and search committee - Search committee and candidates - Search committee and interview panels |
| ☐ | Charge: Have the desired experiences, attributes, competencies, and skills been articulated and defined in outcome terms? |
| ☐ | Culture: Have search committee members articulated what a good fit would be between the organizational culture and values and the leader’s style and preferences? |
| ☐ | Candidates (and Their Competence): Has the committee developed standards or indicators for the level of performance and behavior that meets the demands of the institution’s plan for the new leader and satisfies the dean’s charge? |
| ☐ | Chair: Has the search committee chair been appointed thoughtfully? Does the chair have:   - Adequate administrative support? - Appropriate training? - Accountability for the professional quality of the search? |

### The 10 Characteristics of a Good Search (continued)

| ✔ | Search Characteristic |
| --- | --- |
| ☐ | Composition: When choosing search committee members, ask:   - Do the search committee and interview panels reflect broad and deep diversity of perspectives, backgrounds, and levels within the organization? - Is each member able to respectfully—and strongly—present differing opinions while building consensus? |
| ☐ | Conduct: With respect to the committee and the search process:   - Do search committee members function as a team? - Is the search process being conducted with transparency, confidentiality, and integrity? |
| ☐ | Confidentiality: Does everyone involved in the search—committee members, interviewers, senior leaders—understand the importance of confidentiality for the integrity of the search? With respect to confidentiality:   - Do search committee members agree to keep private all information about search committee proceedings, the identity of potential candidates and candidates, and other search-related discussions? - Is the search committee chair the only person authorized to speak on behalf of the committee? |
| ☐ | Closure: After the committee forwards the list of finalists to the dean:   - Has someone communicated with the candidates who were not chosen? - Have search committee members strategized and prepared to support the new leadership and to help accelerate acceptance and adoption of change? |

### Concierge-Level Service

Directions: Use this checklist as a good practice guide for the application process, including previsit preparation and the actual visit to your institution. You can adapt any of these steps to your unique needs.

| Application: Create a good first impression with applicants and those who may become applicants. Consider the following: | |
| --- | --- |
|  | Are inquiries and applications treated confidentially and acknowledged promptly? |
|  | Are candidates who will not be considered further notified as soon as possible? |
| Previsit: Help candidates make the best use of their time while on campus by providing institutional and departmental dossiers before a campus visit. Consider including the following: | |
|  | Institutional and departmental strategic plans |
|  | Institutional and departmental financial summaries |
|  | Institutional and departmental histories and fact sheets |
|  | Institutional and departmental summaries about education, research, and clinical care |
|  | Organizational charts |
|  | Selected summaries or excerpts of departmental reviews |
|  | Details on important strategic initiatives, new programs, etc. |
|  | Brief biosketches of all individuals with whom the candidate will meet |
|  | Brief biosketches of other key institutional or departmental leaders |
|  | Details about the local community and region |

### Concierge-Level Service (continued)

| Visit: Provide concierge-level service to all candidates to let them know that the institution is serious about attracting top-quality talent and is a great place to work. Consider the following: | |
| --- | --- |
|  | Are candidates cared for the entire time by one recruitment coordinator? |
|  | Does someone meet the candidate at the airport? |
|  | Does the interview schedule address the complete candidate—such as personal interests, the community, schools, real estate, and spousal and family needs? Does the committee even know what the candidate’s needs and personal interests are? |
|  | Does the interview schedule allow for a few moments of personal time during the day? |

### Candidate Feedback Questionnaire Template

**Implementation Guidance:** Use this template as a guide for writing your own feedback questionnaire on the overall search experience and the candidate’s on-campus experience. Distribution of the feedback request can be after the qualifying interview or at the end of the application process. This will help you to capture the applicant’s experience while it’s still fresh in their mind. You will need to determine how to distribute the questionnaire, how to collect responses, and whether responses should be anonymous.

Dear [Candidate Name]:

Thank you for visiting our campus. Applying for the position of [position title here] requires an investment on the part of the candidate as well as the medical school. We appreciate your time and energy in the recruitment and interview process and hope your experience has been positive.

As we strive to give candidates the best experience our organization has to offer, we would appreciate your helping us learn more about your experience in this process.

Please respond to this brief questionnaire about your overall experience in the process. Thank you for your help!

**Please indicate your level of agreement or disagreement with each statement:**

| **Your Overall Experience with the Search** | **Strongly Agree** | **Agree** | **Neutral** | **Disagree** | **Strongly Disagree** | **Not Applicable** |
| --- | --- | --- | --- | --- | --- | --- |
| The search process was well organized. |  |  |  |  |  |  |
| The search process was efficient. |  |  |  |  |  |  |
| I was treated with respect throughout the process. |  |  |  |  |  |  |
| I gained a thorough understanding of the position, the department, and the institution. |  |  |  |  |  |  |
| My questions were answered in a timely and responsive manner. |  |  |  |  |  |  |
| I have a positive impression of the department and the institution because of the search process. |  |  |  |  |  |  |

### Candidate Feedback Questionnaire Template (continued)

**Please indicate your level of agreement or disagreement with each statement:**

| **Your On-Campus Experience** | **Strongly Agree** | **Agree** | **Neutral** | **Disagree** | **Strongly Disagree** | **Not Applicable** |
| --- | --- | --- | --- | --- | --- | --- |
| Travel and accommodations were taken care of in a timely manner. |  |  |  |  |  |  |
| I felt welcomed during my visit. |  |  |  |  |  |  |
| The length of appointments was appropriate. |  |  |  |  |  |  |
| The number of appointments was appropriate. |  |  |  |  |  |  |
| Interviewers were prepared for their meetings with me. |  |  |  |  |  |  |
| Sufficient attention was given to my personal needs and circumstances. |  |  |  |  |  |  |

Using the space below, please describe how your experience as a candidate could have been improved:

## References:

1. Hiring the Best Talent. Association of American Medical Colleges. Accessed February 27, 2024. <https://www.aamc.org/career-development/leadership-development/recruiting>
