# Supplementary material for: Implementing a Holistic Review Toolkit for Faculty Recruitment and Retention
Source: MedEdPORTAL. 2024 Dec 4;20:11472. doi: 10.15766/mep_2374-8265.11472 (PMC11615027; doi:10.15766/mep_2374-8265.11472)
Supplement: Supplementary file 1 — Faculty Pilot Overview.docxOverview Equity-Minded Hiring_Step 1.docxAssess Readiness for Equity-Minded Hiring_Step 1.docxStaff Composition Inventory_Step 2.xlsxHolistic Search Committee Phases and Steps_Step 2.docxFaculty Workshop Facilitators Guide_Step 3.docxFaculty Workshop Presentation_Step 3.pptxFaculty Workshop Evaluation_Step 3.docxFaculty Workshop Activities_Step 3.docxJob Description Posting Tools and Resources_Step 4.docxInterview Questions Tools and Resources_Step 4.docxSubmission Requirements and Rating Tools_Step 4.docx360-Degree (Multisource) Reference Checking_Step 4.docxSearch Process Tools and Resources_Step 5.docxStanding Up a Search Committee_Step 5.docxMitigating Bias Resources_Step 5.docxOnboarding Tools and Resources_Step 6.docxCareer Development Discussion Guide_Step 6.docxU Colorado SOM Mentoring Resource Packet_Step 6.docxBaylor College of Medicine Exit Resources_Step 6.docxU Colorado SOM Equitable Hiring Tool_Step 7.docxHolistic Hiring and Retention Tracker_Step 8.docxEvaluation Materials Development Phase_Steps 4-6.docx [file mep_2374-8265.11472-s001.zip › O. Standing Up a Search Committee_Step 5.docx]

# Appendix O: Standing Up a Search Committee

### Choosing the Search Committee

Implementation Guidance: Use the Overview below and the Search Committee Analysis that follows to select an effective search committee.

Originally published in Mallon WT, Grigsby, RK. *Recruiting: Proven Search and Hiring Practices for the Best Talent.* Association of American Medical Colleges; 2017. Additional resources and information can be found on AAMC’s Hiring the Best Talent Web site.^1^

## Overview

Your goal is to assemble a diverse set of committee members who have a good understanding of the special nature of the institution and the department and who understand the attributes and skill sets required to be successful as a committee member.

Search committees are evolving from representative-based to competency-based. Members of the search committee should be selected based on their talents and skills for identifying the right candidates, not on the constituency each committee member represents. When institutions review a list of individuals to consider as potential committee members, they should consider a broad range of approaches to capture their diverse views. For example, if some individuals are prohibited from serving on search committees by institutional policies, they could meet with the search committee early in the process to share their perspectives or they could participate in interviews of candidates.

**Consider the following competencies** (adapted from the University of Mississippi Medical Center search committee model) to help determine who should be included:

- Can be objective.
- Can be candid.
- Can set aside political agendas.
- Can handle conflict like an adult.
- Can keep confidences.
- Can adopt an institutional orientation.
- Is a good judge of people.
- Is a good listener and communicator.
- Is a skilled interviewer.
- Can be an institutional ambassador.
- Is a good recruiter as well as selector.
- Can compromise and build consensus.

**For department chair searches, consider the following roles** to help ensure a variety of perspectives are included on the search committee:

- Other department chairs
- Faculty members
- Hospital administrators
- Nurses
- Allied health professionals
- Medical residents and fellows
- Medical students
- Postdocs and graduate students
- Community members (both community physicians and laypeople)
- Other internal and external “customers” of the department
- Community physicians and system leaders

## Search Committee Analysis

Directions: Once you have potential committee members in mind, use the chart below to help you analyze the strength of the committee and to choose the people to invite to serve on it. Take a look at the diversity, the contributions that each member could make, and the overall balance of the team.

| **Committee Member** | **Role** | **Perspective** | **Conflicts of Interest**  **(Real or Perceived)** |
| --- | --- | --- | --- |
|  |  |  |  |
|  |  |  |  |
|  |  |  |  |
|  |  |  |  |
|  |  |  |  |
|  |  |  |  |
|  |  |  |  |
|  |  |  |  |
|  |  |  |  |
|  |  |  |  |
|  |  |  |  |

### Dean’s Charge for the Search Committee-Preparation Guide

Implementation Guidance: The dean will fill in the 10 questions in this guide to organize the points to be covered with the search committee BEFORE the committee begins its recruitment and selection work.

Position Title: Click here to enter text.

Position Number**:** Click here to enter text.

Search Committee Members (also identify who will serve as the search chair)**:**

Click here to enter text.

1. Describe the **type of department desired** and its anticipated focus. Provide a summary of the most recent departmental review or access to the entire report.

Click here to enter text.

1. Define the **breadth of the search** as internal, external, statewide, regional, national, or international.

Click here to enter text.

1. Describe the **responsibilities, attributes, and competencies** of the leader being sought.

Click here to enter text.

1. Identify the **essential leadership characteristics** being sought. Set the expectation that committee members will focus on the evaluation of leadership skills and potential rather than traditional academic curriculum vitae and bibliography content.

Click here to enter text.

1. Give precise instructions about the **number of finalists** to be identified and ask that they be submitted unranked in alphabetical order. Outline expectations for diversity in this group. Clarify that no candidate will be considered whose name has not been generated through the committee nomination and evaluation process.

Click here to enter text.

1. Provide guidelines for the creation of the **position description and for the active prospecting** that the search committee and institution should undertake to generate the candidate pool, including active steps to generate a diverse list of candidates.

Click here to enter text.

1. Provide **communication guidelines** for announcing the position. Include deans, chairs, teaching hospital CEOs, key leaders in the field, and national societies, among others.

Click here to enter text.

1. Provide clear instructions about the **confidentiality** of the entire search and screening process. Ask search committee members to adhere to the search committee code of conduct.

Click here to enter text.

### Dean’s Charge for the Search Committee-Preparation Guide (continued)

1. Define expectations for **tasks to be handled** **only by the search committee chair** (e.g., making public statements about the status of the search, checking references).

Click here to enter text.

1. Outline the **timetable** for the search, including expected milestones and the date by which the short list will be given to the dean.

Click here to enter text.

### A Model Search Committee Code of Conduct

Implementation Guidance: Use this model to develop your own committee code of conduct.

## Preamble

You have been asked to serve on this search committee because our organization has a high degree of trust in you. As a search committee member, you will have access to information that is otherwise confidential, and you will assume an important role in the process to select new leaders for the organization.

Therefore, it is imperative that you use the highest standards of ethical and professional conduct to protect the integrity of the process and the integrity of each and every candidate. Accepting the appointment to the search committee attests to your understanding of and commitment to maintaining the highest standards of ethical and professional conduct.

## Standards

As a member of this search committee, I agree to uphold the following principles:

**Honesty:** I agree to fully disclose current and former relationships with candidates. I will fully reveal any real or potential conflicts of interest in the relationships of committee members and candidates. I agree to correct any inaccuracies of commission or omission for which I am responsible.

**Respect for all persons:** I will respect other search committee members and all applicants and candidates. I agree to maintain civility in all transactions with other committee members, search professionals, search-support personnel, potential candidates, and candidates. Furthermore, I agree to attend all meetings unless excused. I pledge to arrive on time and participate fully in the committee activities. I will respect all candidates and participants in the search process across the wide range of diverse backgrounds, temperaments, generations, and orientations.

**Privacy:** I agree to maintain appropriate boundaries and will not go outside the formal process to obtain information about candidates, intrude into private lives of candidates, or participate in gossip about the process or about candidates or others involved in the search process.

**Confidentiality:** I will keep private all information about search committee proceedings, identity of potential candidates or candidates, origin of candidates, and all other search-related discussions even after the search is completed. I acknowledge that the search committee chair is the only person authorized to speak on behalf of the committee.

In addition to adhering to the principles stated above, I agree to participate in and responsibly discharge all the assignments requested of me as a committee member. I will put the best interests of the organization ahead of my personal interests, and I will not allow those personal interests to interfere with my duties as a committee member.

**References:**

1. Hiring the Best Talent. Association of American Medical Colleges. Accessed February 27, 2024. <https://www.aamc.org/career-development/leadership-development/recruiting>
