# Supplementary material for: Implementing a Holistic Review Toolkit for Faculty Recruitment and Retention
Source: MedEdPORTAL. 2024 Dec 4;20:11472. doi: 10.15766/mep_2374-8265.11472 (PMC11615027; doi:10.15766/mep_2374-8265.11472)
Supplement: Supplementary file 1 — Faculty Pilot Overview.docxOverview Equity-Minded Hiring_Step 1.docxAssess Readiness for Equity-Minded Hiring_Step 1.docxStaff Composition Inventory_Step 2.xlsxHolistic Search Committee Phases and Steps_Step 2.docxFaculty Workshop Facilitators Guide_Step 3.docxFaculty Workshop Presentation_Step 3.pptxFaculty Workshop Evaluation_Step 3.docxFaculty Workshop Activities_Step 3.docxJob Description Posting Tools and Resources_Step 4.docxInterview Questions Tools and Resources_Step 4.docxSubmission Requirements and Rating Tools_Step 4.docx360-Degree (Multisource) Reference Checking_Step 4.docxSearch Process Tools and Resources_Step 5.docxStanding Up a Search Committee_Step 5.docxMitigating Bias Resources_Step 5.docxOnboarding Tools and Resources_Step 6.docxCareer Development Discussion Guide_Step 6.docxU Colorado SOM Mentoring Resource Packet_Step 6.docxBaylor College of Medicine Exit Resources_Step 6.docxU Colorado SOM Equitable Hiring Tool_Step 7.docxHolistic Hiring and Retention Tracker_Step 8.docxEvaluation Materials Development Phase_Steps 4-6.docx [file mep_2374-8265.11472-s001.zip › P. Mitigating Bias Resources_Step 5.docx]

# Appendix P: Mitigating Bias Resources

**Implementation Guidance:** These resources can be used to mitigate bias throughout the search, recruitment, and hiring processes. Before utilizing these resources, your institution should review federal and local laws to ensure they align with organizational policies and procedures.

**Unconscious Bias Resources for Health Professionals**^1^

## Content:

- Addressing Implicit Bias in Virtual Interviews
- Online Seminar: The Science of Unconscious Bias and What To Do About it in the Search and Recruitment Process
- Proceedings of the Diversity and Inclusion Forum: Unconscious Bias in Academic Medicine Report
- Video Interview with Howard Ross, author of *Reinventing Diversity: Transforming Organizational Community to Strengthen People, Purpose, and Performance*

**Bias Breakers: Continuous Practice for Admissions and Selection Committees**^2^

## Educational Objectives:

1. Identify the types of bias present in cognition and decision-making.
2. Identify manifestations of bias in committee processes, policies, discussions, and decisions.
3. Apply strategies and skills to mitigate bias in committee processes, policies, discussions, and decisions.
4. Build skills to establish a culture of bias recognition for practitioners and committee members.
5. Achieve more diverse, equitable, and inclusive outcomes by recognizing and mitigating bias during selection processes.

**References:**

1. AAMC. Accessed April 15, 2024. <https://www.aamc.org/about-us/equity-diversity-inclusion/unconscious-bias-training>
2. Nakae S, Palermo AG, Sun M, Byakod R, La T. Bias Breakers: Continuous Practice for Admissions and Selection Committees. *MedEdPORTAL*. 2022;18:11285. <https://doi.org/10.15766/mep_2374-8265.11285>
