# Supplementary material for: Implementing a Holistic Review Toolkit for Faculty Recruitment and Retention
Source: MedEdPORTAL. 2024 Dec 4;20:11472. doi: 10.15766/mep_2374-8265.11472 (PMC11615027; doi:10.15766/mep_2374-8265.11472)
Supplement: Supplementary file 1 — Faculty Pilot Overview.docxOverview Equity-Minded Hiring_Step 1.docxAssess Readiness for Equity-Minded Hiring_Step 1.docxStaff Composition Inventory_Step 2.xlsxHolistic Search Committee Phases and Steps_Step 2.docxFaculty Workshop Facilitators Guide_Step 3.docxFaculty Workshop Presentation_Step 3.pptxFaculty Workshop Evaluation_Step 3.docxFaculty Workshop Activities_Step 3.docxJob Description Posting Tools and Resources_Step 4.docxInterview Questions Tools and Resources_Step 4.docxSubmission Requirements and Rating Tools_Step 4.docx360-Degree (Multisource) Reference Checking_Step 4.docxSearch Process Tools and Resources_Step 5.docxStanding Up a Search Committee_Step 5.docxMitigating Bias Resources_Step 5.docxOnboarding Tools and Resources_Step 6.docxCareer Development Discussion Guide_Step 6.docxU Colorado SOM Mentoring Resource Packet_Step 6.docxBaylor College of Medicine Exit Resources_Step 6.docxU Colorado SOM Equitable Hiring Tool_Step 7.docxHolistic Hiring and Retention Tracker_Step 8.docxEvaluation Materials Development Phase_Steps 4-6.docx [file mep_2374-8265.11472-s001.zip › R. Career Development Discussion Guide_Step 6.docx]

# Appendix R: Career Development Discussion Guide

### Faculty and Staff Template

Implementation Guidance: Give this template to the faculty or staff member at least one week before their scheduled development conversation. Ask them to enter answers to the questions in the white space on this template. Let the faculty or staff member know that their answers will help make the conversation productive.

Originally published in Mallon WT, Grigsby, RK. *Leading: Top Skills, Attributes, and Behaviors Critical for Success.* Association of American Medical Colleges; 2016. Additional resources and information can be found on AAMC’s Hiring the Best Talent Web site.^1^

| Section 1: Where Am I Now? (Performance) |
| --- |
| **Current performance**  Analyze your current performance against position description, goals/targets, and behaviors.  Ask yourself:   - Am I performing as well as I could? - If not, in what areas do I need to improve? - What constructive feedback have I received over the last 12 months or so, and how have I acted on it? What has been the result? |
|  |

| **Development needs**   - What additional skills, knowledge, or expertise might you need to improve your performance? - What were the development needs identified at your last appraisal (if applicable), and how have you progressed in meeting them? - How have you implemented new knowledge, skills, and abilities from learning opportunities, such as development or training programs, and what has been the result? - In what way have you demonstrated commitment to your self-development over the last year or so? |
| --- |
|  |

| **Contribution and achievements**   - What have you contributed to departmental or organizational objectives in the last 12-18 months? - What has been your biggest achievement(s) this year? |
| --- |
|  |

| **Strengths**  What have you done really well? What do you perceive to be your key skills/strengths? |
| --- |
|  |

| Section 2: Where Do I Want to Be? (Potential) |
| --- |
| **Environment**   - What type of environment do you like to work in? - How do you like to employ your skills? - What makes you feel satisfied and fulfilled at work? - What does a great day look like? |
|  |

| **Key skills, knowledge, and behaviors**  Consider how your strengths could be more effectively used in your current role.   - What are your key strengths? Include professional expertise, as well as skills and behaviors. - What are some skills you may not be using currently, such as those from previous roles, outside interests, or languages? |
| --- |
|  |

| **Matching skills to development opportunities**   - What would you like to do *more* of or *less* of in your work? - What would you most want to take with you to another position, and what would you most like to leave behind? - What type of position or role might combine your strengths, desires, and career aspirations? |
| --- |
|  |

| Section 3: How Will I Get There? (Opportunity) |
| --- |
| **Development gaps**  Identify the skills and knowledge gaps between where you are now and where you want to be. Think about what learning experiences could provide these skills and knowledge. |
|  |

| **Helpers and hinderers**  Consider the factors that may affect your career, either positively or negatively. This will help to increase your awareness of them and decide which, if any, factor you can or want to change. Positive factors are generally referred to as “helpers,” and negative ones are known as “hinderers” or obstacles. These factors may be within you (internal), such as feelings, thoughts, and skills, or they may be in your environment (external), such as departmental or organizational priorities, home life, life events, and ability to travel. Always try to think about those factors you can influence rather than those you cannot. |
| --- |
|  |

| **Investment of effort**  Think about how you will commit time and effort to your development; this is important to consider as you will be the owner and driver of your development. (You could talk to like-minded individuals and inquire about how they approached their development.) Decide your preference for career planning. Would you like to draw up a plan spanning several years, or do you prefer to think in stages, perhaps one year or one development need or career step at a time? |
| --- |
|  |

| **Network**  You should always try to have a network of people who will support your development. This may be a mixture of people with whom and from whom you can learn, create ideas, exchange best practices, gain exposure to new things, raise your visibility, etc. Identify which individuals to include in your network and discuss them with your chair. |
| --- |
|  |

### Department Chair Template

Implementation Guidance: To prepare for the conversation, leaders should complete the tasks and answer the questions in the white space on this template. Be prepared to share this information with the faculty or staff member during the conversation.

| Section 1: Where Is This Individual Now? (Performance) |
| --- |
| **Identify strengths in current performance**   - Review job description, current goals/targets, and behaviors against expectations. - Reflect on the faculty/staff member’s main contributions and achievements over the last 12-18 months. (Include previous roles if less than 6 months in role.) |
|  |

| **Identify gaps between current performance and job requirements**   - Review job description, current goals/targets, and behaviors against expectations. - Reflect on the faculty/staff member’s main contributions and achievements over the last 12-18 months. (Include previous roles if less than 6 months in role.) |
| --- |
|  |

| **Gather evidence about current performance and behaviors**  Gather feedback from the faculty or staff member’s collaborators, students, and patients, as applicable. Remember, evidence should always be based on facts, so ask people for examples of performance and behavior. You might suggest a 360° appraisal if appropriate. (If the faculty or staff member has completed a 360° appraisal within the last year, ask them to come to the meeting ready to discuss conclusions and next steps.) |
| --- |
|  |

| **Review progress made in current development plan**   - Review development goals and progress made in attaining these goals. - Review training attended, implementation of new skills/knowledge, and any resulting increase in performance. |
| --- |
|  |

| **Summarize employee’s strengths and development needs** |
| --- |
|  |

| Section 2: Where Could This Individual Go? (Potential) |
| --- |
| **Is the faculty/staff member currently meeting or exceeding expectations?**  Is this individual fulfilling the expectations for the role? If not, why do you think this is? |
|  |

| **What motivates them?**  What does this individual enjoy? When do they seem happiest or most fulfilled? |
| --- |
|  |

| **What evidence of potential have you observed? (See examples below.)**   - Does this individual accomplish work easily? - Do they look to contribute elsewhere, see the bigger picture, or show leadership potential? - Are they a quick learner? - Has this individual developed and leveraged strong internal and/or external relationships? - Do they show commitment to the department and organization? - Do they show initiative or ambition? |
| --- |
|  |

| **How can this faculty/staff member leverage their strengths?**   - What are this individual’s key strengths? - How could these be leveraged to greater effect within your department and/or the wider organization? - What skills might the faculty/staff member have used in a previous job (current CV)? |
| --- |
|  |

| **What is a potential next role (if appropriate)?**  Consider the individual’s transferable skills and future potential (scope for development). What possible next role(s) have you identified that may be a suitable fit, either within or outside your department? |
| --- |
|  |

| Section 3: How Could This Individual Get There? (Opportunity) |
| --- |
| **Development opportunities to help this person grow into next role (or current role)**  Identify learning opportunities, stretch assignments, special projects, work shadowing, new/enriched responsibilities, or experiences that will aid the employee’s development for their next role. |
|  |

| **Building a network**  How will you help your faculty/staff member to build a network? Networks can help with increasing visibility, sharing knowledge, eliciting feedback, and coaching/mentoring. |
| --- |
|  |

| **Blockers**  What personal traits, attitudes, or behaviors might inhibit this employee’s development? Is the individual aware of them? If so, to what extent? (Self-awareness is an important factor in the development journey.) |
| --- |
|  |

| **Any other notes** |
| --- |
|  |

### Career Development Discussion Starters

Implementation Guidance: After you have completed the Career Development Discussion tool, choose any of the following questions that best fit your conversational needs.

## General Probing and Clarifying Questions:

- What do you hope to accomplish?
- What’s next?
- What do you have in mind?
- How can I help? What would be the best way for me to support you? How do you want me to be involved?
- What are the potential outcomes? Can you live with those outcomes?
- What is your role in solving the current problem?
- What strengths do you bring to the conversation?
- What obstacles stand in your way?
- What else can you try?
- May I offer a suggestion or tell you what I’ve observed?
- Do you need more time to think this over?
- Why do you think…?
- How do you plan to…?
- What’s stopping you from…?
- Tell me more....

## Questions to Help Identify Aspirations:

- What is the most rewarding part of your job or of jobs you’ve had in the past?
- What are some things you enjoy doing that you are not doing now?
- What would you like to look back on and say you were most proud of in your career?
- What do you NOT like doing?
- What career goals have you considered?
- Where do you see yourself in five years?
- What obstacles have you identified that may impede your success (geography, work life, skill sets, fears, too much accountability, etc.)? Which of them are you willing to change?

## Questions to Help Check Progress:

- What challenges or barriers are you encountering?
- What assistance or resources do you need?
- What feedback have you been able to apply to your work so far?
- How close are you to reaching your goal?
- What feedback have you received?
- What accomplishments do you feel especially good about?

## Questions to Create Organizational Alignment:

- How might this fill an important organizational need?
- How might this deepen expertise in an area that is critical to our ongoing success?

## Questions to Help Prioritize:

- What are some strengths or skills you would like to use more in your current job?
- Are there any personal values you would like to see reflected more through your work?
- What activities in your job do you find most rewarding?
- Given the changes/challenges that are taking place in the department or organization, where do you see yourself providing the most value?
- How might this help bring about needed change in the department/school/university/ academic health system?
- How might this improve service to those we serve?
- How does this help us create a high-performance organization and culture?
- How does this help us improve our productivity, processes, or quality?

## Questions to Help Debrief a Development Experience:

- What happened? How did it go?
- What worked and why?
- What didn’t work and why?
- What would you have done differently?
- What can you apply this to in your day-to-day work?
- What did you learn?
- How will you practice what you learned? What might be an upcoming opportunity?

**References:**

1. Becoming an Effective Leader. Association of American Medical Colleges. Accessed February 27, 2024. <https://www.aamc.org/career-development/leadership-development/leading>
