# Supplementary material for: Implementing a Holistic Review Toolkit for Faculty Recruitment and Retention
Source: MedEdPORTAL. 2024 Dec 4;20:11472. doi: 10.15766/mep_2374-8265.11472 (PMC11615027; doi:10.15766/mep_2374-8265.11472)
Supplement: Supplementary file 1 — Faculty Pilot Overview.docxOverview Equity-Minded Hiring_Step 1.docxAssess Readiness for Equity-Minded Hiring_Step 1.docxStaff Composition Inventory_Step 2.xlsxHolistic Search Committee Phases and Steps_Step 2.docxFaculty Workshop Facilitators Guide_Step 3.docxFaculty Workshop Presentation_Step 3.pptxFaculty Workshop Evaluation_Step 3.docxFaculty Workshop Activities_Step 3.docxJob Description Posting Tools and Resources_Step 4.docxInterview Questions Tools and Resources_Step 4.docxSubmission Requirements and Rating Tools_Step 4.docx360-Degree (Multisource) Reference Checking_Step 4.docxSearch Process Tools and Resources_Step 5.docxStanding Up a Search Committee_Step 5.docxMitigating Bias Resources_Step 5.docxOnboarding Tools and Resources_Step 6.docxCareer Development Discussion Guide_Step 6.docxU Colorado SOM Mentoring Resource Packet_Step 6.docxBaylor College of Medicine Exit Resources_Step 6.docxU Colorado SOM Equitable Hiring Tool_Step 7.docxHolistic Hiring and Retention Tracker_Step 8.docxEvaluation Materials Development Phase_Steps 4-6.docx [file mep_2374-8265.11472-s001.zip › W. Evaluation Materials Development Phase_Steps 4-6.docx]

Appendix W: Evaluation: Materials Development Phase (Steps 4-6)

The information you provide will be used to inform the evaluation of the tools from the Holistic Approach to Faculty Recruitment and Retention Pilot. This activity has been reviewed according to AAMC policies and procedures. Your information may also be used to inform other research activities in the future**.** Your responses will be securely stored by the AAMC with appropriate access controls to limit exposure of your data to those with a need to know. Participation in this activity is voluntary. You may choose to answer or not any question, and you may withdraw at any time without penalty by contacting the lead researchers.

**Steps 4, 5 and 6** (June 2021 Administration)

Please indicate whether your institution has completed the following Holistic Search Committee phases/steps:

|  | No | In Process | Yes |
| --- | --- | --- | --- |
| Developed tools (e.g., interview questions, candidate rating forms, etc.) to assist in implementing the search plan. |  |  |  |
| Created a *Holistic Review Applicant Evaluation Tool*, based on the identified E-A-C-Ms and the job description, to review applications. |  |  |  |

**If yes, to “Developed tools”** (question in yellow):

|  | Not At All | Only Slightly | Somewhat | For the Most Part | Very Much So |
| --- | --- | --- | --- | --- | --- |
| The relevant job boards, journals, publications, and web sites for the open position have been identified. |  |  |  |  |  |
| The job description was based on the E-A-C-M’s. |  |  |  |  |  |
| The job posting used language that is inclusive and reflective of the school’s mission and diversity goals. |  |  |  |  |  |
| The application included a short-answer essay prompt for applicant’s E-A-C-M’s. |  |  |  |  |  |
| A candidate evaluation form and score sheet was developed. |  |  |  |  |  |
| Structured or semi-structured interview questions (based on the identified E-A-C-M’s) were created. |  |  |  |  |  |
| Legal FAQs were written and made available. |  |  |  |  |  |

**If yes, to “Created a *Holistic Review Applicant Evaluation Tool*”** (question in blue):

|  | Not At All | Only Slightly | Somewhat | For the Most Part | Very Much So |
| --- | --- | --- | --- | --- | --- |
| Interview questions were developed by each search committee member. |  |  |  |  |  |
| The search committee has agreement on the content of the written evaluations of the candidates’ performance in the interview. |  |  |  |  |  |
| A format and specific questions for reference calls for candidates was developed by the search committee. |  |  |  |  |  |
| The criteria for presentation of finalist materials, including completed EACM candidate evaluation forms, was presented to the dean, CEO, or hiring authority. |  |  |  |  |  |

How helpful were the following Holistic Faculty Recruitment tools?

|  | Not At All helpful | Only Slightly helpful | Somewhat helpful | Helpful | Very Helpful | Did Not Use this Tool |
| --- | --- | --- | --- | --- | --- | --- |
| Job Description Tools |  |  |  |  |  |  |
| Sample Interview Questions |  |  |  |  |  |  |
| Sample Interview Rating Tools |  |  |  |  |  |  |
| Search Process Tools |  |  |  |  |  |  |
| Job Posting Resources |  |  |  |  |  |  |
| Applicant Screening Tools |  |  |  |  |  |  |
| Onboarding and Retention Tools |  |  |  |  |  |  |
| Career Development Tools |  |  |  |  |  |  |
| Exit Interview Tools |  |  |  |  |  |  |
| Holistic Review Applicant Evaluation Tool |  |  |  |  |  |  |

How easy was it to adapt each of these tools for your particular use?

|  | Very Difficult | Somewhat Difficult | Neutral | Somewhat Easy | Very Easy |
| --- | --- | --- | --- | --- | --- |
| Job Description Tools |  |  |  |  |  |
| Sample Interview Questions |  |  |  |  |  |
| Sample Interview Rating Tools |  |  |  |  |  |
| Search Process Tools |  |  |  |  |  |
| Job Posting Resources |  |  |  |  |  |
| Applicant Screening Tools |  |  |  |  |  |
| Onboarding and Retention Tools |  |  |  |  |  |
| Career Development Tools |  |  |  |  |  |
| Exit Interview Tools |  |  |  |  |  |
| Holistic Review Applicant Evaluation Tool |  |  |  |  |  |

How long did it take for you to adapt each of these tools for your use (total elapsed time)?

|  | ½ day  (up to four hours) | A day  (no more than 8 hours) | 2 days  (no more than 16 hours) | 3 days  (no more than 24 hours) | 4 or more days (more than 24 hours) |
| --- | --- | --- | --- | --- | --- |
| Job Description Tools |  |  |  |  |  |
| Sample Interview Questions |  |  |  |  |  |
| Sample Interview Rating Tools |  |  |  |  |  |
| Search Process Tools |  |  |  |  |  |
| Job Posting Resources |  |  |  |  |  |
| Applicant Screening Tools |  |  |  |  |  |
| Onboarding and Retention Tools |  |  |  |  |  |
| Career Development Tools |  |  |  |  |  |
| Exit Interview Tools |  |  |  |  |  |
| Holistic Review Applicant Evaluation Tool |  |  |  |  |  |

Feedback: Holistic Approach to Faculty Recruitment and Retention Tools

***Steps 4-6:*** *(Summer 2022 Administration)*

***Step 4: Rewrite Job Descriptions and Interview Questions***

* Indicates required question

1. **How helpful were the job description tools in the completion of step 4? ***

*Mark only one oval.*

- *Not at all helpful*
- *Only slightly helpful*
- *Somewhat helpful*
- *Helpful*
- *Very helpful*
- *Did not use this tool 🡪 Skip to question 5*

1. Thinking about the job description tools, please describe any barriers to adaptation.
2. Thinking about the job description tools, please describe your strategies for adaptation.
3. Thinking about the job description tools, please describe how these tools were used in your search process.
4. **How helpful were the interview questions tools in the completion of step 4?***

*Mark only one oval.*

- *Not at all helpful*
- *Only slightly helpful*
- *Somewhat helpful*
- *Helpful*
- *Very helpful*
- *Did not use this tool 🡪 Skip to question 9*

1. Thinking about the interview question tools, please describe any barriers to adaptation.
2. Thinking about the interview question tools, please describe your strategies for adaptation.
3. Thinking about the interview question tools, please describe how these tools were used in your search process.
4. **How helpful were the interview rating tools in the completion of step 4?***

*Mark only one oval.*

- *Not at all helpful*
- *Only slightly helpful*
- *Somewhat helpful*
- *Helpful*
- *Very helpful*
- *Did not use this tool 🡪 Skip to question 13*

1. Thinking about the interview rating tools, please describe any barriers to adaptation.
2. Thinking about the interview rating tools, please describe your strategies for adaptation.
3. Thinking about the interview rating tools, please describe how these tools were used in your search process.

***Step 5: Update Search Plan and Applicant Evaluation Materials***

1. **How helpful were the search process tools in the completion of step 5?***

*Mark only one oval.*

- *Not at all helpful*
- *Only slightly helpful*
- *Somewhat helpful*
- *Helpful*
- *Very helpful*
- *Did not use this tool 🡪 Skip to question 17*

1. Thinking about the search process tools, please describe any barriers to adaptation.
2. Thinking about the search process tools, please describe your strategies for adaptation.
3. Thinking about the search process tools, please describe how these tools were used in your search process.
4. **How helpful were the job posting resources in the completion of step 5?***

*Mark only one oval.*

- *Not at all helpful*
- *Only slightly helpful*
- *Somewhat helpful*
- *Helpful*
- *Very helpful*
- *Did not use this tool 🡪 Skip to question 21*

1. Thinking about the job posting resources, please describe any barriers to adaptation.
2. Thinking about the job posting resources, please describe your strategies for adaptation.
3. Thinking about the job posting resources, please describe how these tools were used in your search process.
4. **How helpful were the applicant screening tools in the completion of step 5?***

*Mark only one oval.*

- *Not at all helpful*
- *Only slightly helpful*
- *Somewhat helpful*
- *Helpful*
- *Very helpful*
- *Did not use this tool 🡪 Skip to question 25*

1. Thinking about the applicant screening tools, please describe any barriers to adaptation.
2. Thinking about the applicant screening tools, please describe your strategies for adaptation.
3. Thinking about the applicant screening tools, please describe how these tools were used in your search process.

***Step 6: Review Retention Materials***

1. **How helpful were the onboarding and retention tools in the completion of step 6?***

*Mark only one oval.*

- *Not at all helpful*
- *Only slightly helpful*
- *Somewhat helpful*
- *Helpful*
- *Very helpful*
- *Did not use this tool 🡪 Skip to question 29*

1. Thinking about the onboarding and retention tools, please describe any barriers to adaptation.
2. Thinking about the onboarding and retention tools, please describe your strategies for adaptation.
3. Thinking about the onboarding and retention tools, please describe how these tools were used in your search process.
4. **How helpful were the career development tools in the completion of step 6?***

*Mark only one oval.*

- *Not at all helpful*
- *Only slightly helpful*
- *Somewhat helpful*
- *Helpful*
- *Very helpful*
- *Did not use this tool 🡪 Skip to question 33*

1. Thinking about the career development tools, please describe any barriers to adaptation.
2. Thinking about the career development tools, please describe your strategies for adaptation.
3. Thinking about the career development tools, please describe how these tools were used in your search process.
4. **How helpful were the exit interview tools in the completion of step 6?***

*Mark only one oval.*

- *Not at all helpful*
- *Only slightly helpful*
- *Somewhat helpful*
- *Helpful*
- *Very helpful*
- *Did not use this tool 🡪 End survey*

1. Thinking about the exit interview tools, please describe any barriers to adaptation.
2. Thinking about the exit interview tools, please describe your strategies for adaptation.
3. Thinking about the exit interview tools, please describe how these tools were used in your search process.
