# Supplementary material for: Implementing a Holistic Review Toolkit for Faculty Recruitment and Retention
Source: MedEdPORTAL. 2024 Dec 4;20:11472. doi: 10.15766/mep_2374-8265.11472 (PMC11615027; doi:10.15766/mep_2374-8265.11472)
Supplement: Supplementary file 1 — Faculty Pilot Overview.docxOverview Equity-Minded Hiring_Step 1.docxAssess Readiness for Equity-Minded Hiring_Step 1.docxStaff Composition Inventory_Step 2.xlsxHolistic Search Committee Phases and Steps_Step 2.docxFaculty Workshop Facilitators Guide_Step 3.docxFaculty Workshop Presentation_Step 3.pptxFaculty Workshop Evaluation_Step 3.docxFaculty Workshop Activities_Step 3.docxJob Description Posting Tools and Resources_Step 4.docxInterview Questions Tools and Resources_Step 4.docxSubmission Requirements and Rating Tools_Step 4.docx360-Degree (Multisource) Reference Checking_Step 4.docxSearch Process Tools and Resources_Step 5.docxStanding Up a Search Committee_Step 5.docxMitigating Bias Resources_Step 5.docxOnboarding Tools and Resources_Step 6.docxCareer Development Discussion Guide_Step 6.docxU Colorado SOM Mentoring Resource Packet_Step 6.docxBaylor College of Medicine Exit Resources_Step 6.docxU Colorado SOM Equitable Hiring Tool_Step 7.docxHolistic Hiring and Retention Tracker_Step 8.docxEvaluation Materials Development Phase_Steps 4-6.docx [file mep_2374-8265.11472-s001.zip › V. Holistic Hiring and Retention Tracker_Step 8.docx]

# Appendix V: Holistic Hiring and Retention Practices Tracker

**Implementation Guidance:** Before utilizing this tracker, your institution should review federal and local laws to ensure it aligns with organizational policies and procedures.

**Instructions:** Track your department’s or unit’s progress with respect to each of the components that follow. Check the most appropriate box for each element using the following Likert scale:

**1** = *Not at all* | **2** = *Starting to think about it* | **3** = *In the beginning stages of development* |

**4** = *Developed but have not yet implemented fully* | **5** = *Completely developed and implemented*

| **Recruitment** | **1** | **2** | **3** | **4** | **5** |
| --- | --- | --- | --- | --- | --- |
| The department has developed a faculty profile of success using the holistic review ECAM (experiences, attributes, competencies, metrics) approach, and this profile is in alignment with the mission and values of the department. |  |  |  |  |  |
| Targeted recruitment practices that include institutions with high percentage of underrepresented in medicine (URiM) residents, fellows, and faculty are the norm for the department for all searches, e.g., includes historically black colleges or universities, minority-serving institutions, Hispanic-serving institutions, centers of excellence. |  |  |  |  |  |
| The department actively studies attrition rates of URiM faculty. |  |  |  |  |  |
| The department routinely performs exit interviews of URiM faculty. |  |  |  |  |  |
| The department has an advisory committee made up of URiM faculty presently in the department to advise the chair and division chiefs on recruitment and retention best practices. |  |  |  |  |  |
| The department has developed and implemented a URiM faculty recruitment and retention toolkit to assist the chair and division chiefs in their recruitment and retention planning. |  |  |  |  |  |
| **Retention** | **1** | **2** | **3** | **4** | **5** |
| The department chair and/or division chiefs perform stay interviews with URiM faculty. |  |  |  |  |  |
| The department has a structured mentoring academy for URiM faculty. |  |  |  |  |  |
| The department leadership regularly discuss potential sponsorship opportunities for their URiM faculty. |  |  |  |  |  |
| The department demonstrates investment in its URiM faculty by promoting and providing professional and leadership development opportunities to enhance their careers, e.g., encouraging attending AAMC Minority Faculty Leadership Seminars: Early-Career and Mid-Career; AAMC Grant Writing Workshops; and/or AAMC Group on Diversity and Inclusion (GDI) spring meeting. |  |  |  |  |  |
| The department has a designated representative that belongs to the AAMC GDI with the goal of providing the department with information about promising/effective practices in diversity and inclusion matters and opportunities. |  |  |  |  |  |

**1** = *Not at all* | **2** = *Starting to think about it* | **3** = *In the beginning stages of development* |

**4** = *Developed but have not yet implemented fully* | **5** = *Completely developed and implemented*

| **Search Process** | **1** | **2** | **3** | **4** | **5** |
| --- | --- | --- | --- | --- | --- |
| The department has a designated equity advisor that is involved in all searches and advises search committees. |  |  |  |  |  |
| Balanced representation of membership on every search committee is the norm. |  |  |  |  |  |
| All members of departmental search committees must participate in required unconscious bias training in order to serve. |  |  |  |  |  |
| The department has documented specific criteria for the screening of applications describing the necessary characteristics and attributes that are in alignment with the department’s mission and faculty profile of success (EACM). |  |  |  |  |  |
| All applicants are expected to submit a diversity statement that has equal weight with their personal statement, teaching statement, and research statement. |  |  |  |  |  |
| The department chair has granted responsibility and authority to the equity advisor, diversity liaison, or the institution’s chief diversity officer (vice dean of diversity and inclusion) to review and approve all applicant pools before moving forward with the search and selection process to ensure that there is an appropriate pool of diverse candidates being considered. |  |  |  |  |  |
| Balanced representation of participants on all interview panels is the norm. |  |  |  |  |  |
| All interviews have standardized interview questions that are asked of all interviewees. |  |  |  |  |  |
| Standardized interview questions include questions on diversity and inclusion, e.g., “How will you contribute to the diversity of our department?” |  |  |  |  |  |
| All search committee members are evaluated by the equity advisor regarding their performance, noted trends in screening, and scoring, and these evaluations are submitted to the department chair (this performance evaluation will be considered in the future regarding whether or not a member will be asked to serve on future search committees). |  |  |  |  |  |
| **All searches track the following information:** | **1** | **2** | **3** | **4** | **5** |
| - Number of URiM applications |  |  |  |  |  |
| - Number of URiM applications that make it into the selected pool |  |  |  |  |  |
| - Number of URiM applicants that are offered an interview |  |  |  |  |  |
| - Number of URiM applicants that complete an interview |  |  |  |  |  |
| - Number of URiM applicants that withdraw their application |  |  |  |  |  |
| - Number of URiM applicants that are offered a position |  |  |  |  |  |
| - Details of the hiring package offered to URiM applicants |  |  |  |  |  |
| - Number of URiM applicants that accept the offer |  |  |  |  |  |
| - Number of URiM applicants that turn down the offer |  |  |  |  |  |

*Developed by David Acosta, MD, March 22, 2019.*
