# Supplementary material for: Implementing a Holistic Review Toolkit for Faculty Recruitment and Retention
Source: MedEdPORTAL. 2024 Dec 4;20:11472. doi: 10.15766/mep_2374-8265.11472 (PMC11615027; doi:10.15766/mep_2374-8265.11472)
Supplement: Supplementary file 1 — Faculty Pilot Overview.docxOverview Equity-Minded Hiring_Step 1.docxAssess Readiness for Equity-Minded Hiring_Step 1.docxStaff Composition Inventory_Step 2.xlsxHolistic Search Committee Phases and Steps_Step 2.docxFaculty Workshop Facilitators Guide_Step 3.docxFaculty Workshop Presentation_Step 3.pptxFaculty Workshop Evaluation_Step 3.docxFaculty Workshop Activities_Step 3.docxJob Description Posting Tools and Resources_Step 4.docxInterview Questions Tools and Resources_Step 4.docxSubmission Requirements and Rating Tools_Step 4.docx360-Degree (Multisource) Reference Checking_Step 4.docxSearch Process Tools and Resources_Step 5.docxStanding Up a Search Committee_Step 5.docxMitigating Bias Resources_Step 5.docxOnboarding Tools and Resources_Step 6.docxCareer Development Discussion Guide_Step 6.docxU Colorado SOM Mentoring Resource Packet_Step 6.docxBaylor College of Medicine Exit Resources_Step 6.docxU Colorado SOM Equitable Hiring Tool_Step 7.docxHolistic Hiring and Retention Tracker_Step 8.docxEvaluation Materials Development Phase_Steps 4-6.docx [file mep_2374-8265.11472-s001.zip › _Educational Summary Report_11472.pdf]

MedEdPORTAL® The AAMC Journal of Teaching and Learning Resources

Original Publication Open Access

Implementing a Holistic Review Toolkit for Faculty Recruitment and Retention

Toi Blakley Harris, MD*, Michelle Shader, MA, Patrick O. Smith, PhD, Amelia Challender, MA, Cleveland Piggott, MD, MPH, Norma I. Poll, PhD, Ryan Henyard, MEd, Jeffrey M. Lyness, MD, Erik D. Malmberg, PhD, JD, Eva K. Pressman, MD, Alicia D. Monroe, MD, Negar N. Jacobs, PhD

*Corresponding author: toi.harris@memorialhermann.org

Abstract

Introduction: A diverse workforce improves health care, educational outcomes, and research agendas. Currently, faculty from historically excluded groups remain underrepresented in academic medicine. Resources are needed in academic medical centers for those charged with recruiting and retaining a diverse and talented workforce. **Methods:** Informed by the successful use of holistic review in medical student admissions, the Association of American Medical Colleges (AAMC) developed and piloted a toolkit for use in faculty recruitment and retention at five academic medical centers. Institutions led toolkit-guided holistic review workshops ranging from 2.5 to 3 hours in duration for leaders and faculty to identify and prioritize selection criteria used to modify faculty recruitment and retention materials and processes. AAMC surveys at pilot initiation and at two time points during implementation assessed satisfaction with and perceptions of the toolkit. **Results:** At the first survey time point, the average toolkit rating across five institutions ranged from 3.2 to 4.4 (out of a maximum of 5.0) for helpfulness and from 3.5 to 4.9 for ease of use. At the second survey time point, the helpfulness of the tools received average ratings of 3.0-4.8. In respondents' qualitative comments, we identified varying strategies for implementation and few barriers to implementation other than reluctance of some faculty to use scripted tools and concerns about anonymity in the exit interviews. **Discussion:** The toolkit was well received and easy to implement. It will be important to study the use of these materials across other institutions, with attention to their impact on faculty diversity.

Keywords
Faculty, Retention, Diversity, Equity, Inclusion, Faculty Affairs, Faculty Development, Promotions & Tenure, Recruitment

Educational Objectives

By the end of this session, learners will be able to:

1. Assess their institutional readiness for incorporating holistic review strategies for faculty recruitment, selection, and advancement.
2. Describe how holistic recruitment practices can expand their institution's search criteria to help the institution achieve its mission and goals.
3. Apply the holistic review framework to their institution's faculty recruitment, selection, and advancement processes.

Citation:
Harris TB, Shader M, Smith PO, et al. Implementing a holistic review toolkit for faculty recruitment and retention. *MedEdPORTAL*. 2024;20:11472. https://doi.org/10.15766/mep_2374-8265.11472

Introduction

A diverse workforce improves health care, educational outcomes, and research agendas.[1] In addition, workforce diversity has frequently been cited as a strategy to improve health across populations, including historically marginalized groups. Elevating health equity as the fifth component of the quintuple aim highlights the disproportionate impacts of health inequities and underscores the relevance of diversity, equity-mindedness, and conscious inclusion to leverage the full benefits of a diverse workforce.[2,3]

Despite efforts to improve and sustain faculty diversity, a 4-decade analysis of US medical faculty showed that while female representation has improved, other groups that have been historically underrepresented in medicine (URiM) remain underrepresented in clinical faculty; even greater disparities persist at the leadership level.[4] Our definition of URiM, meaning underrepresented relative to the numbers in the general

Copyright © 2024 Harris et al. This is an open-access publication distributed under the terms of the Creative Commons Attribution license.

population and by setting, has been based on the National Institutes of Health's definition of underrepresented populations in research and includes individuals from racial and ethnic groups that have been shown to be underrepresented, individuals with disabilities, individuals from disadvantaged backgrounds, and other aspects of diversity.[5,6] Compounding efforts to retain diverse faculty, promotion rates for racially and ethnically URiM faculty are lower than their White and Asian colleagues.[7] The literature about faculty diversity primarily focuses on the impact of implicit bias training for faculty search and recruitment committees; however, these resources do not include toolkits to increase diversity at every stage of the recruitment process, and they do not extend beyond the recruitment phase to include advancement and retention.[8-10] Because holistic review has been shown to be effective in increasing student diversity, we have adapted its principles for use in recruiting and retaining faculty.[11,12]

In 2007, the Association of American Medical Colleges (AAMC) launched an advisory committee to formalize the concepts of holistic review for application in medical school admissions. The resulting approach is designed to align with an organization's mission and aims to promote diversity in the medical workforce to address societal needs.[11] Since its introduction in 2010, medical school admissions committees have used the holistic review framework to evaluate applicants comprehensively, considering their experiences, attributes, and academic metrics in relation to the institution's mission. In 2016, Baylor College of Medicine (BCM) partnered with the AAMC to pilot application of the holistic review framework in faculty recruitment and selection.[13] A key adaptation of the faculty recruitment framework was the addition of competencies. Expanding on this collaboration and recognizing the significance of addressing faculty retention, a national working group was created to investigate the framework's broader application across the faculty life cycle.[14] In July 2019, an AAMC-sponsored webinar was held to socialize the need for and premise of the pilot and to invite webinar participants to apply. The webinar participants represented 10 medical schools and one teaching hospital, seven of which applied. The pilot required leadership and human resources endorsement; all applicants had institutional support and were accepted into the pilot. The multisite pilot launched in December 2019. This pilot aimed to adapt and implement the framework for faculty recruitment and retention, addressing a critical gap: the need for processes that increase diversity within the faculty. Due to pandemic challenges, two institutions did not complete the pilot. This left five sites, two from the south, one from the northeast, and two from the west, to fully implement the faculty recruitment and retention pilot.

The final pilot, involving five institutions, has demonstrated promise as a strategy for advancing faculty diversity goals.[14] This project aimed to assess the feasibility and perceptions of applying the tools provided in implementing a holistic approach to faculty recruitment and retention. We assessed readiness for holistic review and satisfaction with applying the model and tools at each institution.

The comprehensive toolkit is designed to provide educational resources and tools for leaders and teams in academic medical centers who focus on faculty recruitment, advancement, and retention. This publication provides information about our pilot's unique application of the toolkit, perceptions of the toolkit, and lessons learned during its implementation.

Methods

Phase 1: Education and Investigation (Steps 1-3)
Five academic medical centers were selected by the AAMC to participate in this pilot based on their organizational commitment to make changes in their recruitment and retention practices.[14] These institutions were diverse in size, location, and type (i.e., public or private) and included BCM, University of Colorado School of Medicine, University of Mississippi Medical Center School of Medicine, University of Nevada, Reno, School of Medicine, and University of Rochester School of Medicine and Dentistry. As the leads of this pilot project, we each assembled institutional implementation teams in late 2019 to build on the work initially piloted by BCM and the AAMC's holistic review team.[15] Before proceeding, it was determined that institutional review board review was not needed for participation.

We worked closely with AAMC staff to create a comprehensive toolkit of resources informed by best practices across the faculty life cycle.[15] The resources were organized to lead institutions stepwise through the change process and included evidence-based tools originally designed by the second author for the AAMC's *Leading: Top Skills, Attributes, and Behaviors Critical for Success*[16] and *Recruiting: Proven Search and Hiring Practices for the Best Talent*[17] publications. The AAMC's legal counsel was also engaged to advise on the structure and process of the pilot. Of note, although employment law has not changed based on the June 2023 Supreme Court decision on race-conscious admissions (the law governing employment practice and decisions for hiring is covered by Title VII of the Civil Rights Act, which has long prohibited the consideration of an applicant's race),[18] the AAMC encourages institutions to consult their legal

Copyright © 2024 Harris et al. This is an open-access publication distributed under the terms of the Creative Commons Attribution license.

counsel on federal and state laws when restructuring existing strategies to improve the recruitment, retention, promotion, and evaluation of leaders, faculty, staff, and learners representing groups that have been historically marginalized. Additionally, students, faculty, staff, and the local community should all be engaged, and their input considered when adopting and applying these strategies.

Creating implementation teams was a critical preliminary step because this group of people was responsible for shepherding change at each of our institutions. Implementation team members were stakeholders with a deep understanding of institutional context and culture; team members with experience in facilitation, data analysis, project management, and diversity strategic planning; and representatives who could drive change. An overview of and implementation guidance for the entire AAMC Holistic Faculty Recruitment and Retention Initiative can be found in Appendix A. In step 1, implementation teams, drawing on the framework outlined in Appendix B, sought buy-in from key institutional leaders, such as deans, leaders in faculty affairs, department chairs, and human resource professionals. Once the key stakeholders were oriented to equity-minded hiring practices, we reviewed our institutions' readiness to engage in institutional change using a standardized assessment developed by the AAMC (Appendix C).

During the next step, we collected baseline faculty demographic data (Appendix D), reviewed our current search practices against the AAMC's description of Holistic Search Committee Phases and Steps (Appendix E), identified gaps in our current processes, and updated our search plans based on holistic principles.

Step 3 required each pilot institution to deliver the holistic principles workshop. Workshops ranged in length from 2.5 to 3 hours and were delivered in early 2020. The workshop had several objectives: (1) to learn about the holistic review framework's application for faculty hiring, (2) to select expanded and holistic search criteria aligned with institutional mission and goals, and (3) to identify opportunities to change recruitment and hiring practices. The criteria selected and actions taken in these workshops laid the foundation for the remainder of the pilot process. Tools available for the completion of step 3 included the Holistic Principles Facilitator Guide (Appendix F), which provided an outline of in-person and virtual setup, materials needed, delivery instructions, and a content outline. Each school identified a skilled facilitator in advance to deliver the workshops. A complete slide deck with editable speaker notes was also provided (Appendix G). Each site evaluated its own workshops, but we here provide a standard evaluation based on the workshop goals (Appendix H).

Three activities guided discussion and decision-making among recruiting teams (Appendix I). In activity 1, participants selected Experience, Attribute, Competency, and Metric (EACM) criteria that are best aligned with their institutional mission to prioritize during the faculty and staff selection process. By the end of activity 1, each group had identified their top two to three selection criteria within each domain of the EACM framework. Once the EACMs had been identified, activity 2 helped each group define and operationalize each prioritized criterion. They also developed, using activity 3, an action plan to move the work forward within their implementation unit. Some institutions completed these activities in one sitting, while other institutions divided content delivery and assigned the activities as intersession work.

Phase 2: Recruitment, Selection, and Retention Materials and Process Updates (Steps 4-6)

After the workshop, members of each institution's implementation team used the toolkit materials to align their existing recruitment, selection, onboarding, and retention materials and processes with holistic review principles and the priorities identified. Each pilot site worked with the appropriate stakeholders at their own institution to update their hiring processes and practices. The development and update of recruitment and selection materials constituted a highly integrated process, and although materials were presented as separate areas of focus, changes in one process often affected another. For example, as job descriptions changed, those changes affected interview questions and how candidates were being evaluated. We outline the tools in each area and describe how the holistic principles were incorporated into existing institutional resources below.

In step 4, we began by updating job descriptions, interview questions, submission requirements, and applicant evaluation materials based on the EACMs identified by each implementation team. Tools included Job Description Tools and Resources (Appendix J), which provided sample job description guidelines and an example of a job description showing how BCM incorporated their priority EACMs into a search as well as a curated list of recommended job-posting sites. Interview Questions Tools and Resources (Appendix K) included sample holistic interview questions to identify priority EACMs and competence in diversity, equity, and inclusion. These tools and resources were adapted by each team. Additionally, guidance on interview questions that could pose legal risk to organizations were summarized (Appendix K). Lastly, Interview Rating Tools

Copyright © 2024 Harris et al. This is an open-access publication distributed under the terms of the Creative Commons Attribution license.

and Resources sample submissions requirements and a sample candidate rating tool used by BCM were provided as models that could be adopted by each team (Appendix L). A 360-degree reference-checking best-practice guide was also provided (Appendix M).

Simultaneously, utilizing the EACM framework in step 5, each team reviewed their search process, updated their search plans, and conducted hiring package analyses for equity purposes. Search Process Tools (Appendix N) included an overview of the characteristics of a good search, a good-practice guide for the application process, and a template to get feedback on how the candidate experienced the overall search process. Resources on how to form a search committee (Appendix O) and help mitigate bias throughout the recruitment and selection process (Appendix P) were provided.

In step 6, retention materials at each pilot site were reviewed, including onboarding materials, career development tools, and exit interview processes. A modifiable department chair onboarding guide was provided for reference (Appendix Q). Career development tools included a series of tools for holding career development discussions (Appendix R). Additionally, a comprehensive mentoring resource guide was developed by the University of Colorado Anschutz Medical Campus Department of Family Medicine because of pilot participation (Appendix S). Lastly, a sample exit survey and exit interview questions were developed by BCM (Appendix T). These materials were adapted for use at each institution. The phase of updating materials and processes ranged from 6 to 18 months depending on the institution.

Phase 3: Implementation and Continuous Improvement (Steps 7-8)

After the materials had been updated, we presented and socialized the updated forms and interview questions to hiring managers and search committees (step 7). To operationalize new hiring processes, the University of Colorado Anschutz Medical Campus Department of Family Medicine developed an Equitable Hiring Tool to help guide and track their new hiring processes (Appendix U). Finally, in step 8, we continually monitored and improved our processes. We reviewed how the process was going with our pilot team and tracked changes in hiring and retention over time. Tools to help with this tracking can be found in Appendix V. As with all new policies and interventions, it was important to communicate this information broadly. We developed communication strategies recommending that all pilot institutions share their work and outcomes broadly at their institution.

Evaluation of the Toolkit

We assessed the impact of our pilot by participating in a multisite evaluation led by the AAMC, including surveys throughout the implementation process designed to measure each institution's satisfaction with and perceptions of the toolkit. Each institution provided its feedback on the faculty holistic review process and rated the helpfulness of each domain of tools at two points during the implementation. In June of 2021, pilot institutions completed an initial evaluation survey about the utility of the resources, ease of adoption, and estimated amount of time to adapt the tools. It is important to note that at the time of this survey administration, some institutions had not yet had the opportunity to fully adopt the toolkit, as some of our recruitment efforts were halted by the pandemic, budget cuts, and hiring freezes. As the implementation of the pilot progressed, we completed a second survey in the summer of 2022 to assess the helpfulness of the tools used in postworkshop implementation and asked open-ended questions to assess strategies for adaptation, how we used the tools, and barriers to adoption. At this point in the implementation, most institutions had implemented the tools for recruitment, and two out of five had implemented the tools for retention. Every institution completed both surveys (see both surveys in Appendix W).

Results

In June of 2021, participants evaluated the holistic faculty recruitment tools created for this pilot. The tools in the toolkit were assessed on how helpful each was, how easy it was to adapt each one for respondents' particular use, and how long it took to adapt them. Each institution identified one individual to complete the survey on behalf of the pilot team ($n = 5$). Table 1 shows the average ratings of the five pilot institutions. Overall, the toolkit was found to be helpful, with ratings of helpfulness ranging from 3.2 to 4.4 on a 5-point scale (1 = *not at all helpful,* 5 = *very helpful*). Institutions also evaluated the tools as easy to adapt for their particular use, with ratings ranging from 3.5 to 4.9 on a 5-point scale (1 = *very difficult,* 5 = *very easy*). Participants were also asked how many hours it took for them to adapt the tools, and responses ranged from less than 4 hours to 4-8 hours.

The Sample Interview Questions and the Holistic Review Applicant Evaluation Tool were viewed as the most useful recruitment tools (average of 4.4). The Sample Interview Questions had the easiest reported adaptation of use (4.9) and were adapted on average in less than 4 hours. Similarly, the Holistic Review Applicant Evaluation Tool was considered easy (3.7) to quickly adapt (4-8 hours). Onboarding and Retention

Copyright © 2024 Harris et al. This is an open-access publication distributed under the terms of the Creative Commons Attribution license.

Table 1. Pilot Participant Evaluation Feedback: Utility, Ease of Use, and Time to Adapt Tools (June 2021 Administration)

Faculty Recruitment Tool	How Helpful Were the Following Holistic Faculty Recruitment Tools?[a]	How Easy Was It to Adapt Each of These Tools for Your Particular Use?[b]	How Long Did It Take for You to Adapt Each of These Tools for Your Use (Total Elapsed Time)?[c]
Job Description Tools	4.0	3.9	4-8 hours
Sample Interview Questions	4.4	4.9	<4 hours
Sample Interview Rating Tools	4.2	3.9	<4 hours
Search Process Tools	3.9	3.7	4-8 hours
Job Posting Resources	3.5	3.5	4-8 hours
Applicant Screening Tools	3.9	3.7	<4 hours
Onboarding and Retention Tools	3.2	4.1	4-8 hours
Career Development Tools	3.6	4.1	4-8 hours
Exit Interview Tools	3.6	4.1	4-8 hours
Holistic Review Applicant Evaluation Tool	4.4	3.7	4-8 hours

[a]Rated on a 5-point scale (1 = *not at all helpful*, 2 = *only slightly helpful*, 3 = *somewhat helpful*, 4 = *helpful*, 5 = *very helpful*).
[b]Rated on a 5-point scale (1 = *very difficult*, 2 = *somewhat difficult*, 3 = *neutral*, 4 = *somewhat easy*, 5 = *very easy*).
[c]Rated on a 5-point scale (1 = *½ day*, 2 = *a day*, 3 = *2 days*, 4 = *3 days*, 5 = *4 or more days*).

Tools received the lowest rating (3.2) but were considered easy to adapt (4.1), with adaptation taking 4-8 hours.

In the summer of 2022, pilot institutions were again asked to provide feedback on the helpfulness of the tools as well as answer open-ended questions assessing barriers to implementation, strategies for adaptation, and how the tools were used in their search process. Table 2 summarizes the evaluation data and provides representative quotes of themes found in the qualitative responses about strategies for implementation, application of the tools, and barriers to implementation. During this administration, participants were not asked to evaluate the Holistic Review Applicant Evaluation Tool.

At this administration, participants continued to rate the tools as helpful, with scores ranging from 3.0 to 4.8 on a 5-point scale (1 = *not at all helpful,* 5 = *very helpful*). The Sample Interview Questions were again rated as the most useful (4.8). Illustrative comments on strategies for implementation showed that questions based on the EACMs that teams had selected and agreed upon were used to screen candidates during interviews and to replace less structured processes for screening applicants. When asked about barriers to implementation, most participants identified none, and one institution noted that faculty can be resistant to scripted interviews. The Exit Interview Tools were the lowest rated (3.0), and institutions used them as a template to create their own interview or script. One school mentioned the barrier of threats to anonymity when using the Exit Interview Tools, as demographic data were included in the questionnaire and this could compromise anonymity in a small school.

Discussion

Across the two survey points, our results show overall favorable impressions of the educational activity with respect to helpfulness of the tools as well as their ease of adaption and adoption. This pilot project monitored the application of a holistic faculty recruitment systems model in five dissimilar institutions and revealed favorable reception to the process and toolkit resources. We found that the toolkit was well received and easy to implement and that barriers to adoption were documented. Survey results focused on the helpfulness of tools, time to adapt the recruitment tools, and barriers to implementation. At the 2021 administration, helpfulness of faculty recruitment tools was high (range: 3.2-4.4), as was recruitment tools' ease of use (3.5-4.9), suggesting that the tools were easy to use and adapt. The perceived time to adapt recruitment tools for use was less than 8 hours across all nine tools. The 2022 administration also revealed high satisfaction with the tools (3.0-4.8), with varying strategies for implementation and specific barriers to adoption noted. Thus, satisfaction with the toolkit was high, and ease of use and time to adapt the tools for use across the five institutions were reasonable and feasible.

We developed a process including a workshop activity and learned that application and implementation of a faculty-focused holistic recruitment systems model required flexibility to accommodate variations across the five institutions. This project began in 2019, and all institutions experienced challenges related to the global pandemic. In addition, the racial awakening incited by the murder of George Floyd and others contributed to the climate of our nation. Institutionally, there were direct effects on the workforce including lifestyle changes, various forms of discrimination and prejudice, environmental implications, health and mental health concerns, and delivery of educational programming.

This multisite pilot of the faculty holistic review toolkit included numerous strengths: (1) The framework drew upon the evidence-based holistic review process for medical student selection;

Copyright © 2024 Harris et al. This is an open-access publication distributed under the terms of the Creative Commons Attribution license.

Table 2. Pilot Participant Feedback: Tool Implementation, Application, and Implementation Barriers (Summer 2022 Administration)

Tool/Resource	Mean Rating[a]	Illustrative Comments: Strategies for Implementation	Illustrative Comments: Application	Illustrative Comments: Barriers to Implementation
Job Description Tools	3.8	"We used the template provided and completed the generic information that would be used department wide, leaving only the specifics for completion from the chiefs."	"We used them to replace some existing tools."	"We have our own way of doing job descriptions here."<br>"Our university has its own policies."
Sample Interview Questions	4.8	"Teams were provided sample questions to approve or disapprove based on the EACMs they identified."	"We used them in screening phone interviews and for first interviews with candidates."<br>"We have replaced less structured processes."	"Faculty can be resistant to scripted interviews."<br>"No barriers."
Sample Interview Rating Tools	4.4	"We created online surveys based on them."<br>"The rating tools were most helpful as our prior ones had inherent biases."	"The tools were used as a guide to help us create our own surveys online."<br>"They are offered as a tool to division chiefs to use with their candidate recruitment."	"Internal SOM policies requiring ratings to be put online and that rating system being different."<br>"None."
Search Process Tools	4.2	"Using the list of places to advertise."	"The tools were helpful in keeping the framework focused on inclusion."<br>"We review best practices and materials at a kickoff meeting and enforce use."	"Lack of budget and institutional support to expand recruitment using external resources to draw potential faculty."<br>"No barriers."
Job Posting Resources	4.3	"We added places to advertise to our usual list."	"The tools were provided to the department business administrator, division chiefs, and HR to share opportunities hey may wish to pursue."	"Cost is prohibitive on many sites and we find that a lot of our candidates are still using the bigger job posting sites anyway."
Applicant Screening Tools	4.5	"Continue to keep inclusion front and center."<br>"We adopted these to our institution and the EACMs of our positions."	"Added to our screening process."<br>"It was given to search committee members along with the applications."	"No barriers."
Onboarding and Retention Tools	3.5	"Documents were given to Dean, Associate Dean for Faculty, and Chairs."	"Adopted suggestions to our existing process."	"We haven't gotten this far yet with many searches."<br>"We already had a robust onboarding process."
Career Development Tools	4.0	"We incorporated some aspects into regularly scheduled meetings."<br>"Documents have been given to the onboarding team."	"Dean for Faculty is using the documents and they will be given to Chairs."	"We haven't gotten this far yet."<br>"Convincing faculty to set aside time needed for faculty development."
Exit Interview Tools	3.0	"These were used as a template to create our own interview."	"We used these as a template to create our own exit interview script."	"We have such a small school that anonymity is threatened when you ask any demographic questions."

Abbreviation: EACM, Experience, Attribute, Competency, and Metric; SOM, School of Medicine.
[a]Rated on a 5-point scale (1 = *not at all helpful*, 2 = *only slightly helpful*, 3 = *somewhat helpful*, 4 = *helpful*, 5 = *very helpful*).

(2) it filled a gap in the literature regarding resources to recruit a diverse faculty workforce by providing a toolkit that all institutions rated highly; (3) the institutions in the pilot were highly diverse, suggesting that the toolkit could be successfully implemented across a range of academic health centers with faculty and staff; (4) the model ranges beyond recruitment to address advancement and retention; and (5) the model provides a promising practice to increase equity in the hiring process.

The limitations of our work varied across a small number of sites, and each institution's operational team independently created implementation priorities, which limits the generalizability of our findings. Additionally, since each institution completed its own workshop evaluation, we could not report on the workshop results as a whole. To better standardize the workshop evaluation for this publication, we have created an evaluation (Appendix H) that aligns with the learning objectives for the workshop (slide 2 in Appendix G). The timing of the pilot with the COVID pandemic limited our ability to evaluate the effectiveness of the toolkit with respect to faculty retention, as only two of the five pilot institutions were able to implement retention tools. The small sample size and the short timeline of the pilot project compared with the long-term nature of this assessment were also limitations. Although the pilot primarily focused on faculty, the framework has applicability to staff. Additional limitations include a lack of assessment of the outcomes of the toolkit, especially the ability of the processes and tools to increase the diversity of faculty who are recruited and retained in academic medicine. Data collection on these outcomes is underway at our pilot institutions, and next steps include analysis and dissemination of these data.

Copyright © 2024 Harris et al. This is an open-access publication distributed under the terms of the Creative Commons Attribution license.

This holistic faculty recruitment pilot demonstrated a found pilot approach, where best practices from holistic admissions were applied to faculty recruitment and retention across five institutions, receiving favorable feedback. Creating a diverse workforce is an ongoing process with significant long-term implications for institutions. Using this model, as detailed in Appendix A, is the first step. The next steps involve expanding the toolkit's implementation beyond the pilot institutions to assess its short- and long-term impacts on a broader scale while continuing to monitor outcomes within the pilot group. Additionally, the incorporation of the latest legislative rulings into our processes may need to occur, depending on location.

Appendices

A. Faculty Pilot Overview.docx
B. Overview Equity-Minded Hiring_Step 1.docx
C. Assess Readiness for Equity-Minded Hiring_Step 1.docx
D. Staff Composition Inventory_Step 2.xlsx
E. Holistic Search Committee Phases and Steps_Step 2.docx
F. Faculty Workshop Facilitators Guide_Step 3.docx
G. Faculty Workshop Presentation_Step 3.pptx
H. Faculty Workshop Evaluation_Step 3.docx
I. Faculty Workshop Activities_Step 3.docx
J. Job Description Posting Tools and Resources_Step 4.docx
K. Interview Questions Tools and Resources_Step 4.docx
L. Submission Requirements and Rating Tools_Step 4.docx
M. 360-Degree (Multisource) Reference Checking_Step 4.docx
N. Search Process Tools and Resources_Step 5.docx
O. Standing Up a Search Committee_Step 5.docx
P. Mitigating Bias Resources_Step 5.docx
Q. Onboarding Tools and Resources_Step 6.docx
R. Career Development Discussion Guide_Step 6.docx
S. U Colorado SOM Mentoring Resource Packet_Step 6.docx
T. Baylor College of Medicine Exit Resources_Step 6.docx
U. U Colorado SOM Equitable Hiring Tool_Step 7.docx
V. Holistic Hiring and Retention Tracker_Step 8.docx
W. Evaluation Materials Development Phase_Steps 4-6.docx

All appendices are peer reviewed as integral parts of the Original Publication.

Toi Blakley Harris, MD: Senior Vice President, Chief Equity, Diversity and Inclusion Officer, and Executive Director, Institute for the Advancement of Health Equity, Memorial Hermann Health System; ORCID: https://orcid.org/0000-0003-1976-5321

Michelle Shader, MA: Director, Holistic Initiatives and Learning, Academic Affairs, Association of American Medical Colleges; ORCID: https://orcid.org/0000-0003-2008-7601

Patrick O. Smith, PhD: Chief Faculty Affairs Officer, Division of Academic Affairs, University of Mississippi Medical Center; Associate Dean of Faculty Affairs and Professor, Department of Family Medicine, University of Mississippi School of Medicine

Amelia Challender, MA: Associate Program Director of Education, University of Colorado Family Medicine Residency, and Senior Instructor, Department of Family Medicine, University of Colorado School of Medicine at the Anschutz Medical Campus

Cleveland Piggott, MD, MPH: Vice Chair for Diversity, Equity, and Inclusion and Associate Professor, Department of Family Medicine, University of Colorado School of Medicine at the Anschutz Medical Campus

Norma I. Poll, PhD: Senior Director, Human Capital Portfolio, Equity, Diversity & Inclusion, Association of American Medical Colleges

Ryan Henyard, MEd: Director of Faculty and Education Initiatives, Equity, Diversity & Inclusion, Association of American Medical Colleges

Jeffrey M. Lyness, MD: President and CEO, American Board of Psychiatry and Neurology; Professor Emeritus, Department of Psychiatry, University of Rochester School of Medicine and Dentistry

Erik D. Malmberg, PhD, JD: Assistant Dean, Equity and Medical Student Engagement, and Associate Teaching Professor, Department of Family Medicine, University of Washington School of Medicine

Eva K. Pressman, MD: Henry A. Thiede Professor and Chair, Department of Obstetrics and Gynecology, University of Rochester School of Medicine and Dentistry

Alicia D. Monroe, MD: Chief Integration Officer and Senior Advisor to the President, Office of the President, Old Dominion University

Negar N. Jacobs, PhD: Associate Dean for Diversity and Inclusion and Professor, Department of Psychiatry and Behavioral Sciences, University of Nevada, Reno, School of Medicine

Acknowledgments
The authors thank the Association of American Medical Colleges' Advancing Holistic Principles Advisory Committee and the Executive Leadership teams in Academic Affairs and Equity, Diversity, and Inclusion for their invaluable feedback, guidance, and support. We are especially grateful to Dr. David Acosta for his partnership and for allowing us to feature his original work in this toolkit. Special thanks to our AAMC colleagues, Hannah Turner and Charles Roads, for their editorial expertise and tireless efforts in preparing our submission and toolkit. Finally, we acknowledge the pilot institutions for their sustained commitment, insights, and innovations, which have enriched this submission and enhanced its value for the broader community.

Disclosures
None to report.

Copyright © 2024 Harris et al. This is an open-access publication distributed under the terms of the Creative Commons Attribution license.

Funding/Support
None to report.

Ethical Approval
Reported as not applicable.

References

1. Nivet MA. A diversity 3.0 update: are we moving the needle enough? *Acad Med*. 2015;90(12):1591-1593. https://doi.org/10.1097/acm.0000000000000950
2. Nundy S, Cooper LA, Mate KS. The quintuple aim for health care improvement: a new imperative to advance health equity. *JAMA*. 2022;327(6):521-522. https://doi.org/10.1001/jama.2021.25181
3. Farrell TW, Greer AG, Bennie S, Hageman H, Pfeifle A. Academic health centers and the quintuple aim of health care. *Acad Med*. 2003;98(5):563-568. https://doi.org/10.1097/acm.0000000000005031
4. Kamran SC, Winkfield KM, Reede JY, Vapiwala N. Intersectional analysis of U.S. medical faculty diversity over four decades. *N Engl J Med*. 2022;386(14):1363-1371. https://doi.org/10.1056/NEJMsr2114909
5. Notice of NIH's interest in diversity. National Institutes of Health Office of Extramural Research. November 22, 2019. Accessed November 14, 2024. https://grants.nih.gov/grants/guide/notice-files/NOT-OD-20-031.html
6. Get the facts. National Institutes of Health Diversity in Extramural Programs. Accessed November 14, 2024. https://extramural-diversity.nih.gov/diversity-matters/get-the-facts
7. Xierali IM, Nivet MA, Syed ZA, Shakil A, Schneider FD. Recent trends in faculty promotion in U.S. medical schools: implications for recruitment, retention, and diversity and inclusion. *Acad Med*. 2021;96(10):1441-1448. https://doi.org/10.1097/acm.0000000000004188
8. Jacobs NN, Esquierdo-Leal J, Smith GS, Piasecki M, Houmanfar RA. Diversifying academic medicine: one search committee at a time. *Front Public Health*. 2022;10:854450. https://doi.org/10.3389/fpubh.2022.854450
9. Cahn PS. Recognizing and reckoning with unconscious bias: a workshop for health professions faculty search committees. *MedEdPORTAL*. 2017;13:10544. https://doi.org/10.15766/mep_2374-8265.10544
10. Nakae S, Palermo AG, Sun M, Byakod R, La T. Bias Breakers: continuous practice for admissions and selection committees. *MedEdPORTAL*. 2022;18:11285. https://doi.org/10.15766/mep_2374-8265.11285
11. Conrad SS, Addams AN, Young GH. Holistic review in medical school admissions and selection: a strategic, mission-driven response to shifting societal needs. *Acad Med*. 2016;91(11): 1472-1474. https://doi.org/10.1097/acm.0000000000001403
12. Glazer G, Danek J, Michaels J, et al. *Holistic Admissions in the Health Professions: Findings From a National Survey*. Urban Universities for HEALTH; 2014. Accessed November 14, 2024. https://www.aplu.org/wp-content/uploads/holistic-admissions-in-the-health-professions.pdf
13. Harris TB, Thomson WA, Moreno NP, et al. Advancing holistic review for faculty recruitment and advancement. *Acad Med*. 2018;93(11):1658-1662. https://doi.org/10.1097/ACM.0000000000002364
14. Harris TB, Jacobs NN, Fuqua CF, et al. Advancing equity in academic medicine through holistic review for faculty recruitment and retention. *Acad Med*. 2022;97(5):631-634. https://doi.org/10.1097/acm.0000000000004568
15. Viggiano TR, Strobel HW. The career management life cycle: a model for supporting and sustaining faculty vitality and wellness. In: Cole TR, Goodrich TJ, Gritz ER, eds. *Faculty Health in Academic Medicine*. Humana Press; 2009. Accessed November 14, 2024. https://doi.org/10.1007/978-1-60327-451-7_6
16. Mallon WT, Grigsby RK. *Leading: Top Skills, Attributes, and Behaviors Critical for Success*. Association of American Medical Colleges; 2016.
17. Mallon WT, Grigsby RK. *Recruiting: Proven Search and Hiring Practices for the Best Talent*. Association of American Medical Colleges; 2017.
18. *Frequently Asked Questions: What Does the Harvard and UNC Decision Mean for Medical Education?* Association of American Medical Colleges; 2023. Accessed November 14, 2024. https://www.aamc.org/media/68771/download?attachment%3Fattachment

Received: April 16, 2024
Accepted: September 5, 2024
Published: December 4, 2024

Copyright © 2024 Harris et al. This is an open-access publication distributed under the terms of the Creative Commons Attribution license.
